# Molecular genotype-phenotype correlation in *ACTB-* and *ACTG1-*related non-muscle actinopathies

**DOI:** 10.1016/j.ajhg.2025.12.007

**Published:** 2026-01-12

**Authors:** Nataliya Di Donato, NMA Consortium, Andrew Thom, Andreas Rump, Johannes N. Greve, Juan Cadiñanos, Rocco Salvatore Calabrò, Sara Cathey, Brian Chung, Heidi Cope, Maria Costales, Sara Cuvertino, Philine Dinkel, Kalliopi Erripi, Andrew E. Fry, Livia Garavelli, Sabine Hoffjan, Wibke G. Janzarik, Insa Kreimer, Grazia Mancini, Purificacion Marin-Reina, Andrea Meinhardt, Indra Niehaus, Daniela Pilz, Ivana Ricca, Fernando Santos Simarro, Evelin Schrock, Anja Marquardt, Manuel H. Taft, Kamer Tezcan, Sofia Thunström, Judith Verhagen, Alain Verloes, Bernd Wollnik, Peter Krawitz, Tzung-Chien Hsieh, Michael Seifert, Michael Heide, Catherine B. Lawrence, Neil A. Roberts, Dietmar J. Manstein, Adrian S. Woolf, Siddharth Banka

**Affiliations:** 1 Institute for Clinical Genetics, Medical Faculty and University Hospital Carl Gustav Carus, TUD Dresden University of Technology, Fetscherstraße 74, 01307 Dresden, Germany; 2 Department of Human Genetics, Hannover Medical School, Carl Neuberg Str. 1, 30625 Hannover, Germany; 3 Division of Evolution, Infection and Genomics, School of Biological Sciences, Faculty of Biology, Medicine and Health, University of Manchester, Manchester, UK; 4 University Institute for Medical Genetics, Klinikum Oldenburg, Oldenburg, Germany; 5 Institute for Biophysical Chemistry and Structural Biochemistry, Hannover Medical School, Carl-Neuberg-Str. 1, 30625 Hannover, Germany; 6 Instituto de Medicina Oncológica y Molecular de Asturias IMOMA, Oviedo, Spain; 7 Fundación Centro Médico de Asturias, Oviedo, Spain; 8 IRCCS Centro Neurolesi Bonino-Pulejo, Messina, Italy; 9 Greenwood Genetic Center, Greenwood, IN, USA; 10 Department of Paediatrics and Adolescent Medicine, University of Hong Kong, Hong Kong, Hong Kong; 11 Department of Pediatrics, Division of Medical Genetics, Duke University Medical Center, Durham, NC, USA; 12 Otorhinolaryngoly Department, Hospital Universitario Central de Asturias, Hospital Central de Asturias, Oviedo, Spain; 13 Division of Evolution and Genomic Sciences, Faculty of Biology, Medicine, and Health, School of Biological Sciences, University of Manchester, Manchester, UK; 14 Ophthalmology Department, University Hospital of Gothenburg, Sahlgrenska, Sweden; 15 Institute of Medical Genetics, University Hospital of Wales, Cardiff, UK; 16 Division of Cancer and Genetics, School of Medicine, Cardiff University, Cardiff, UK; 17 Medical Genetics Unit, Azienda USL-IRCCS di Reggio Emilia, 42123 Reggio Emilia, Italy; 18 Department of Human Genetics, Ruhr-University Bochum, Bochum, Germany; 19 Department of Neuropediatrics and Muscle Disorders, Center for Pediatrics and Adolescent Medicine, Medical Center, Faculty of Medicine, University of Freiburg, Freiburg, Germany; 20 Department of Clinical Genetics, Erasmus MC University Medical Center Rotterdam, 3015 GD Rotterdam, the Netherlands; 21 Dysmorphology and Clinical Genetics, Department of Neonatology, Hospital Universitari i Politècnic La Fe. Valencia, Valencia, Spain; 22 West of Scotland Clinical Genetics Service, Queen Elizabeth University Hospital, Glasgow, UK; 23 Molecular Medicine for Neurodegenerative and Neuromuscular Diseases Unit, IRCCS Stella Maris Foundation, Pisa, Italy; 24 Institute of Medical and Molecular Genetics, Hospital La Paz Institute for Health Research, Madrid, Spain; 25 Department of Genetics, Kaiser Permanente, Sacramento, CA, USA; 26 Department of Clinical Genetics and Genomics, Sahlgrenska University Hospital, Gothenburg, Sweden; 27 Department of Genetics, APHP-Robert DEBRE University Hospital, Denis Diderot School of Medicine, Paris University, Paris, France; 28 Institute of Human Genetics, University Medical Center Göttingen, Göttingen, Germany; 29 DZHK German Center for Cardiovascular Research, Partner Site Göttingen, Göttingen, Germany; 30 Cluster of Excellence “Multiscale Bioimaging: from Molecular Machines to Networks of Excitable Cells” MBExC, University of Göttingen, Göttingen, Germany; 31 Institute for Genomic Statistics and Bioinformatics, University Hospital Bonn, Rheinische Friedrich-Wilhelms-Universität Bonn, Bonn, Germany; 32 Institute for Medical Informatics and Biometry IMB, Carl Gustav Carus Faculty of Medicine, TU Dresden, Fetscherstraße 74, 01307 Dresden, Germany; 33 German Primate Center, Leibniz Institute for Primate Research, Göttingen, Germany; 34 Division of Neuroscience and Experimental Psychology, and Geoffrey Jefferson Brain Research Centre, Faculty of Biology, Medicine and Health, Manchester Academic Health Science Centre, University of Manchester, Manchester, UK; 35 Geoffrey Jefferson Brain Research Centre, Northern Care Alliance NHS Foundation Trust, Manchester Academic Health Science Centre, University of Manchester, Manchester, UK; 36 Division of Cell Matrix Biology and Regenerative Medicine, School of Biological Sciences, Faculty of Biology, Medicine and Health, University of Manchester, Manchester, UK; 37 Division for Structural Biochemistry, Hannover Medical School, Carl Neuberg Str. 1, 30625 Hannover, Germany; 38 Manchester Centre for Genomic Medicine, St. Mary’s Hospital, Central Manchester University Hospitals NHS Foundation Trust, Manchester Academic Health Science Centre, Manchester, UK

**Keywords:** non-muscle actinopathies, cytoskeleton, cytoskeletal actin, ACTB, ACTG1, Baraitser-Winter cerebrofrontofacial syndrome, ACTB-related dystonia-deafness syndrome, hearing loss

## Abstract

Recent advances in Mendelian genomics reveal the importance of variant-level characterization of allelic disorders. Non-muscle actin isoforms, encoded by the genes *ACTB* and *ACTG1*, are the most abundant intracellular proteins, but historically, they are often regarded as merely being “housekeeping” molecules. Here, we illuminate the extraordinary clinical heterogeneity and complex pathobiology of genetic non-muscle actinopathies. To do this, we combine human genomics studies with molecular biology. Strikingly, variants in *ACTB* and *ACTG1* isoforms generate at least eight distinct clinical disorders. A subset of disease-associated missense variants causes dysregulated actin polymerization-depolymerization and neuronal migration defects. In contrast, nonsense, frameshift, and missense variants enhancing protein degradation cause milder phenotypes or are benign. These results emphasize the essential functional aspects of the non-muscle actin isoforms. Critically, they additionally constitute a template for the personalized genetic variant-level-driven management of the pleiotropic allelic single-gene disorders.

## Teaser

Through systematic evaluation of 290 individuals with *ACTB* or *ACTG1* variants, we delineate eight non-muscle actinopathies with distinct clinical profiles. Clear genotype-phenotype correlations, characteristic facial gestalts, and organ involvement patterns support improved diagnostic classification, prognostication, and counseling, providing clinicians with a practical framework for managing these rare disorders.

## Introduction

The ability to make accurate diagnoses, reliably estimate prognoses, and having a deep understanding of underpinning molecular and disease mechanisms are prerequisites to developing precision medicine approaches for genetic disorders. Recent advances in Mendelian genomics have dismantled the “one-gene one-disorder paradigm.”^1^ This means that many genetic disorders will likely require characterization at the variant level, not just at the gene level. We attempted dissection of a complex set of allelic disorders caused by constitutional variants in two genes encoding for actin isoforms, which we here refer to collectively as non-muscle actinopathies (NMAs).

Actin is the most abundant intracellular protein, highly versatile and essential for numerous cellular processes. The mammalian actin gene family consists of four muscle-specific isoforms (*ACTA1* [MIM: 102610], *ACTA2* [MIM: 102620], *ACTC1* [MIM: 102540], and *ACTG2* [MIM: 102545]) and two ubiquitously expressed genes, *ACTB* (MIM: 102630) and *ACTG1* (MIM: 102560), coding for the highly conserved β- and γ-cytoplasmic actins (CYAs), respectively. Human βCYA and γCYA differ only in four out of 375 amino acids but possess different polymerization properties, localize in different parts of the cell, and display preferred interactions with different subsets of actin-binding proteins.^2^

NMAs listed in OMIM include *ACTB*-related Baraitser-Winter cerebrofrontofacial syndrome 1 (BWCFF1 [MIM: 243310]), dystonia-deafness syndrome 1 (DDS1 [MIM: 607371]),^3^ thrombocytopenia 8 with dysmorphic features and developmental delay (MIM: 620475),^4^ *ACTG1*-related BWCFF2 (MIM: 614583),^5^^,^^6^ and dominant deafness 20/26 (MIM: 604717).^7^^,^^8^ An additional documented NMA, *ACTB*-associated isolated ocular coloboma,^9^ is not listed in OMIM. A neurodevelopmental-congenital malformation disorder due to loss-of-function *ACTB* variants^10^ is included within BWCFF1. Furthermore, postzygotic somatic mosaic *ACTB* variants have been detected in Becker’s nevus, segmental odontomaxillary dysplasia, and congenital smooth muscle hamartoma with or without hemihypertrophy.^11^^,^^12^^,^^13^ Nevertheless, the full mutational and clinical spectrum of human monogenic disorders caused by *ACTB* or *ACTG1* variants has yet to be systematically explored. Challenges in assigning pathogenicity and in linking individual variants to specific NMAs hamper accurate diagnosis. Even after a specific NMA is diagnosed, clinical management and providing prognosis is challenging due to limited data. Moreover, the pathobiology of NMAs is poorly understood. For example, it is not known whether disease-causing missense variants (MVs) in NMAs are hypomorphic, dominant-negative, or gain-of-function. Because of these limitations, no specific treatments for these disorders exist.

Here, we undertake detailed studies of disease-causing variants in *ACTB* and *ACTG1*. We describe a cohort of 290 individuals with pathogenic or likely pathogenic (P/LP) variants and propose a variant- and phenotype-based classification system that delineates NMAs and defines their clinical spectrum. Using *in vitro* cellular and biochemical studies, we provide variant-level insights into the molecular mechanisms of NMAs.

## Material and methods

### Simulation of all possible nucleotide substitutions within genomic regions of *ACTB* and *ACTG1*

For each of the 1,128 reference alleles in *ACTB* transcript chr7:5527148–5530601 (hg38) (GenBank: NM_001101.5) and 1,128 in *ACTG1* transcript chr17:81509971–81512799 (hg38) (GenBank: NM_001614.5), all three possible single-nucleotide alterations were simulated *in silico*, resulting in a list of 1,128 × 3 × 2 = 6,768 alternate alleles (ALT) for both transcripts (Table S1). The functional consequences of all possible single-nucleotide alterations were annotated according to Ensembl Variant Effect predictor.^14^

### Compilation of *ACTB* and *ACTG1* population variants and public databases

We retrieved gnomAD v.3.1.1 (hg38) genomic variants from genomic regions chr7:5527148–5530601 (*ACTB*) and chr17:81509971–81512799 (*ACTG1*).^15^ To prevent redundancy with v.3.1.1., the gnomAD v.2.1.1 (hg19) variants were downloaded for exomes only; exons were defined by gnomAD standard transcripts ENSG00000075624.9 (*ACTB*) and ENSG00000184009 (*ACTG1*), including gnomAD’s default padding of 75 nt. The Ensembl tool “assembly converter” converted the genomic positions of gnomAD v.2 hg19 variants to hg38. We exploited each of the different gnomAD subsets separately: controls (2,148 variants), non-TOPMed (3,107 variants), non-neuro (3,476 variants), and non-cancer (3,605 variants). We retrieved 2,184 genomic variants passing TOPMed filter (i.e., flagged as PASS in VCF “FILTER” column) (Freeze8 on GRCh38, accessed on November 1, 2021) within the genomic regions chr7:5527148–5530601 (ACTB) and chr17:81509971–81512799 (ACTG1).^16^ We retrieved 2,280 variants from https://grch38.pggsnv.org/index.html (accessed on July 31, 2021) within the genomic regions chr7:5527148–5530601 (*ACTB*) and chr17:81509971–81512799 (*ACTG1*).^17^ 752 variants were retrieved from the COSMIC catalog using gene names *ACTB* and *ACTG1* (441 and 311 variants, respectively). The query for cBioPortal mutations (not including structural variants and copy-number alterations) was performed using a user-defined list containing HUGO gene symbols *ACTB* and *ACTG1* (accessed on July 31, 2021 with the query of 46,305 individuals, representing 48,834 samples in 188 studies).^18^^,^^19^ This query identified 389 unique and a total number of 508 variants. ClinVar variants were retrieved with the NCBI search term using hg38 coordinates of both genes (accessed on November 3, 2021)^20^: (7[CHR] AND 5527148[CHRPOS]:5530601[CHRPOS]) and (17[CHR] AND 81509971[CHRPOS]:81512799[CHRPOS]). From LOVD the search for all variants in *ACTB* (GenBank: NM_001101.3 transcript reference sequence) and *ACTG1* (GenBank: NM_001614.3 transcript reference sequence) resulted in 110 and 195 variants, respectively (accessed on November 3, 2021).^21^

### Clinical and genomic analysis of the cohort

#### Study approval

The study was approved by the Institutional Review Board (IRB) of TU Dresden (EK-127032017 and BO-EK-341062021) and the Central Manchester (02/CM/238) and local IRBs from referring physicians. All individuals consented to participation prior to participating. Written informed consent was also received for all photographs of individuals included. The record of informed consent has been retained.

#### Compilation of the clinical cohort

Patients were recruited primarily at the Institute for Clinical Genetics, University Hospital Dresden (by N.D.D.) as part of the active registry within the EJPRD-funded PredACTINg project as well as at the Manchester Center for Genomic Medicine within the NHS sequencing projects (by S.B.) after the rare variant in *ACTB* or *ACTG1* was discovered in the clinical genetic testing. Additionally, affected individuals were referred to N.D.D. and S.B. by multiple clinicians for the second opinion regarding variant interpretation, management advice, or specifically for this study. Clinical and molecular data were collected with the standardized proforma (supplemental information). Clinical photographs were available for 160 persons and were independently assessed by four authors experienced in dysmorphology and specifically familiar with BWCFF (N.D.D., S.B., G.V.M., and D.P.). Additionally, PubMed was searched for publications using terms “ACTB” OR “ACTG1” OR “Baraitser-Winter syndrome” OR “Baraitser-Winter-Cerebrofrontofacial syndrome” OR “Dystonia-deafness syndrome” OR “ACTG1 hearing loss” OR “ACTB neurodevelopmental disorder” OR “ACTG1 neurodevelopmental disorder.” We extracted reported data from identified publications and added follow-up information where available. In addition, we screened the ClinVar database for (likely) pathogenic variants and variants of uncertain significance and contacted responsible laboratories requesting clinical information.

#### Genomic- and phenotypic-led approach for classification of the clinical cohort

Available clinical data, facial photos, and MR images of all individuals were systematically reassessed regarding their phenotypical assignment by one of the corresponding authors. For classification of the cohort into relevant NMA groups, first all individuals were separated according to the gene in which their variant was located. Predicted loss-of function (pLoF) variants and protein-altering variants (PAVs) (missense or in-frame) were then separated. Clinical features of persons with pLoF variants were manually analyzed. Facial images were available for 166 of 275 individuals with small single-nucleotide variants (SNVs). Definition of the facial gestalt is described separately in the next section. The identified clinical features identified from these individuals were used to identify individuals with PAVs whose phenotype overlapped with those with pLoF variants. Next, individuals with recurrent PAVs were identified, their clinical features were manually analyzed, and genotype-phenotype relationships were extracted. Clinical data from these individuals were used to generate diagnostic criteria for each NMA. These diagnostic criteria were applied to individuals with non-recurrent variants, and additional individuals were identified for each subgroup. The clinical and mutation spectrum of each NMA was compiled using the combined data. The approach is summarized in Figure S1.

#### Definition of the syndrome specific facial gestalt

Four medical geneticists experienced in dysmorphology who have seen multiple (>10) individuals with BWCFF (G.M., D.P., S.B., and N.D.D.) independently evaluated clinical pictures of 89 individuals with the pathogenic variants in *ACTB* and *ACTG1*. Images were copied into one file without mentioning the genotype or additional clinical data. Frontal facial photographs of different quality were available for all individuals. Lateral images were available for more than two-thirds of the individuals. Body images as well as hand and feet photographs were provided for less than the half of the individuals. Twelve additional individuals with the microdeletions encompassing the entire *ACTB* gene^10^ were shown with the genotype. Individuals were grouped as having a typical BWCFF face, typical *ACTB* pLoF face (guided by the facial features associated with deletions), or not having typical features of any of both disorders. The phenotypes were discussed in several online meetings.

#### GestaltMatcher facial analysis

To assess similarity between cohorts, we utilized GestaltMatcher^22^^,^^23^ to analyze 75 images spanning BWCFF (*n* = 38; including ACTB_BWCFF *n* = 23 and ACTG1_BWCFF *n* = 15), ACTB LoF (*n* = 19), unNMA (*n* = 15; ACTG1_unNMA *n* = 6 and ACTB_unNMA *n* = 9), and BWCFF_unNMA (*n* = 3). The dataset comprises affected individuals reported in this work and images previously published in the GestaltMatcher database^24^ (GMDB; https://db.gestaltmatcher.org). We first encoded each image to a 512-dimensional feature vector by GestaltMatcher and further utilized t-distributed stochastic neighbor embedding (t-SNE)^25^ to visualize the distribution of the four groups of subjects in two-dimensional space.

Moreover, we wanted to compare the similarities between each group and the control distribution sampled from GMDB. Because the facial phenotypic similarity between two persons was quantified by the cosine distance, when the distance was smaller, the two individuals were more similar as they are closer in the space. We then examined the mean pairwise cosine distance between individuals among the three groups. We sampled control distributions from 1,499 images with 321 different disorders in the GMDB that were not included in the training of GestaltMatcher and calculated the mean pairwise distances between two cohorts stemming (1) from the same syndrome and (2) from two different syndromes. We further derived a threshold to decide whether two cohorts stem from the same or different syndromes by receiver-operating characteristic analysis, resulting in a final threshold of *c* = 0.896. To assess the similarity between the two cohorts C_1_ and C_2_, we computed their mean pairwise cosine distance*d*(C_1_, C_2_), and compared it to the threshold *c*. Additionally, we conducted 100 subsampling iterations from each cohort to generate subcohorts, calculating the mean pairwise cosine distance for each iteration. If at least 50% of these 100 subsampled comparisons yielded values above the threshold *c*, it would provide evidence suggesting that the two cohorts stem from different syndromes.

To quantify how distinct are the two cohorts (C_1_ and C_2_), we computed the positive predictive value (PPV) for an observed intercohort distance *d* that falls within the range of distances between sampled subgroups of C_1_ and C_2_: *d* ∈ (min *d*(C_1_, C_2_), max *d*(C_1_, C_2_)).^23^ PPV was estimated from pooled control distributions built on the validation folds using PPV = (sensitivity × *p*)/(sensitivity × *p* + (1 − specificity) × (1 − *p*)), where sensitivity = *P*(distance in range | different syndromes), specificity = *P*(distance outside range | same syndrome), and *p* is the pre-test probability that two cohorts are from different syndromes. We set *p* = 0.5 to reflect no prior information (equal prior odds of “same” vs. “different”). In this framework, a higher PPV indicates greater evidence that the two cohorts are truly distinct.

To assess whether each subgroup (unNMA, ACTB_unNMA, ACTG1_unNMA, BWCFF, and ACTB LoF) exhibits phenotypic cohesion, we computed the mean pairwise distance among images within each subgroup. As a reference, we simulated a control distribution by repeatedly sampling equally sized batches of individuals from the GMDB drawn across different syndromes and computing their mean pairwise distances. Each subgroup’s within-group mean distance was then located on this control distribution to derive an empirical left-tail percentile, which reflects how unusually similar the subgroup is relative to random batches of subjects. Lower mean distance and lower percentile indicate stronger intragroup similarity.

### Structural modeling

The structures of human βCYA and γCYA were homology modeled using the Schrödinger Prime 4.0 and BioLuminate applications (Schrödinger, New York, NY). The sequences were retrieved from the UniProt database (accession numbers UniProt: P60709 and P63261). The crystal structure of *Bos taurus* β-actin (PDB: 2btf) was used as a template. Mismatched residues in the template were replaced with those in the target structures, and the strontium ion was replaced with a magnesium ion. Model optimization of the resulting structures was performed by a series of iterative rotamer prediction and energy minimization steps.

### Fibroblast experiments

#### Establishment of the subject-derived and control fibroblast cultures

Primary dermal fibroblasts were obtained following 3-mm cutaneous punch biopsies and cultured in BIO-AMF-2 medium (Biological Industries USA, Cromwell, CT, USA). For subculturing, primary fibroblasts were washed twice with 1× Dulbecco’s PBS (dPBS) and detached at 37°C for at least 3 min with 0.05% trypsin/EDTA (Gibco; Thermo Scientific, Waltham, MA, USA). Cells were resuspended in BIO-AMF-2 medium, seeded onto Corning plasticware (Corning, NY, USA), and maintained in BIO-AMF-2 medium at 37°C in the presence of 5% CO_2_. Cultures were continued for a maximum of three passages and, thereafter, the cultures were cryopreserved in multiple cryovials for long-term storage at −150°C. 90% FBS + 10% dimethyl sulfoxide was used as a freezing medium. Thawing of the frozen cells was performed rapidly in a 37°C water bath. Thawed cells were centrifuged at 240 × *g* for 5 min and resuspended in fresh BIO-AMF-2 medium in a new flask. Only cultures in early passages (maximum seven passages) have been used in the experiments. Cultures were labeled with the actin amino acid change and a subject’s ID corresponding to the ID in Table S2 (e.g., G343S 61-B).

Primary dermal fibroblasts from nine healthy adult individuals were obtained and stored following the same procedure described above. These cell lines are labeled as Control_1 and Control_2, as well as Control_6 through Control_12. Only cultures in early passages (maximum seven passages) were used in the experiments. Additionally, three control cell lines were acquired from the Coriell Biobank—GM00013 (passage 13), GM04390 (passage 11), and GM05294 (passage 8)—and labeled Control_3, Control_4, and Control_5, respectively. Coriell cultures were only used for the transcriptome analysis.

#### Cell lysis and western blot analysis

Cells grown to approximately 70% confluence were washed with room-temperature dPBS twice. 0.6 mL of RIPA buffer (Santa Cruz, sc-24948) was added to the monolayer cells in a T25 flask and gently rocked for 15 min at 4°C. Adherent cells were removed with a cell scraper, and the lysate was transferred into a microcentrifuge tube. Lysate was incubated for 5 min on ice before centrifugation at 14,000 × *g* for 10 min at 4°C, and the supernatant was collected. Total protein concentration was measured with the Qubit fluorometer and Qubit protein assay kit (Thermo Fischer Scientific). Approximately 7 μg of protein was separated on 4%–12% NuPAGE Bis-Tris gels (Thermo Fischer Scientific) by electrophoresis (90 V for approximately 2.5 h) under reducing conditions with 6 μL of ProSieve QuadColor pre-stained protein marker loaded. Proteins were blotted onto a 0.2-μm pore nitrocellulose membrane with the iBlot2 system (Thermo Fischer Scientific). Revert 700 Total Protein stain (LI-COR, 926-11011) was performed for normalization of target protein signal intensities, and images of wet membranes were acquired with the Odyssey SA LI-COR Infrared Imaging system in the 700-nm channel (LI-COR). Subsequently, membranes were blocked overnight at 4°C in 5% (w/v) skim milk in TBS-Tween 20 (TBS-T). On the next day, membranes were incubated with primary antibodies diluted in 0.5% (w/v) skim milk in TBS-T for 2 h at room temperature (see Table S4 for antibodies and dilutions used). After three washing steps with 0.5% (w/v) skim milk in TBS-T, membranes were incubated with secondary antibodies for 1 h at room temperature with three subsequent washes with TBS-T followed by three washes with TBS. Next, images of dried membranes were acquired again with the Odyssey Imaging system in the 800-nm channel. Analysis of western blot signals was performed with CLIQS Gel Image Analysis software (TotalLab). Target protein signals were normalized to corresponding total lane signals of total protein stain, and lastly the samples from affected individuals were normalized to the respective control cell line. Results were obtained from three different lysates of independent cell passages with up to three replicates for each sample. GraphPad Prism v.9.3.1 for Windows (GraphPad Software, La Jolla, CA, USA; http://www.graphpad.com/) was used for graphical illustration and statistical analysis. Data were tested for normal distribution using the Shapiro-Wilk test and subsequently analyzed using ordinary one-way ANOVA. Outlier analysis was performed using the ROUT method.

#### Transcriptome analysis of primary fibroblast cultures

##### Library preparation and sequencing of biological and technical replicates

Transcriptome analysis was done using primary fibroblast cultures from 15 individuals with missense variants in *ACTB*, three individuals with variants in *ACTG1*, and seven control cultures from healthy adult individuals (Control_1, Control_2, and Control_6 through Control_10) as well as three fibroblast cultures from Coriell Biobank (Control_3 through Control_5). All cultures were harvested at early passages of a maximum of seven, except for cultures from Coriell, which were available in the later passages (up to passage 15).

Fibroblasts were seeded in T75 flasks and harvested at ∼70% confluence. The culture was performed twice to produce biological replicates. RNA was extracted using the miRNeasy Mini Kit (Qiagen) according to the manufacturer’s instructions. On-column DNA digestion was included to remove residual contaminating genomic DNA. All experiments were performed in triplicate, meaning that RNA was independently extracted three times from each culture. In total, we performed six library preparations per individual (affected individuals and control subjects). For library preparations, the TruSeq Stranded mRNA Library Prep Kit (Illumina) was used according to the manufacturer’s protocol, starting with 1 μg of total RNA. All barcoded libraries were pooled and sequenced 2 × 75-bp paired-end on an Illumina NextSeq500 platform to obtain a minimum of 10 million reads per sample. Raw reads from Illumina sequencers were converted from bcl to fastq format using bcl2fastq (v.2.20) allowing for one barcode mismatch.

##### Bioinformatics pipeline

The quality of the obtained fastq files was initially checked by FastQC v.0.11.4 (https://www.bioinformatics.babraham.ac.uk/projects/fastqc/) followed by adapter removal and quality trimming using Trim Galore v0.4.2 (http://www.bioinformatics.babraham.ac.uk/projects/trim_galore/). Mapping of reads to the human reference genome (GRCh38 Ensembl release 95) was done using STAR v.2.5.3a with standard settings,^26^ and duplicates were marked and removed using Picard tools v.1.141 (http://broadinstitute.github.io/picard/). Quality analysis of mapped reads was done using RSeQC v.3.0.0^27^ to analyze read distributions across gene bodies. Raw read counts per gene were determined by counting gene-specific reads in exons of protein-coding genes using FeatureCounts v.1.5.3.^28^ Finally, a gene-expression data matrix was created by removing genes without any reads and lowly expressed genes (less than 1 read per million in more than 50% of samples) followed by cyclic loess normalization,^29^ resulting in normalized log_2_ counts per million for 12,772 protein-coding genes that were measured in each sample. The average gene-expression levels per affected individual or control individual are provided in Table S3.

##### Similarity of gene-expression profiles and differential gene-expression analysis

Principal-component analysis of average genome-wide gene-expression profiles of study subject and control samples was done to analyze whether the different disease and control groups form separate clusters (R function prcomp). Further, similarity of genome-wide gene-expression profiles of samples from affected individuals and control samples was determined by computing the Pearson correlation coefficient for each pair of samples utilizing the average expression profile of each subject and control sample (R function cor). The corresponding correlation matrix was visualized as a heatmap (Figure S12).

### Protein production and characterization

#### Generation of plasmid and baculovirus

The pFastBac vectors carrying the sequence of interest were constructed and generated as described previously.^30^ Mutations in the sequence were introduced via site-directed mutagenesis with oligonucleotides encoding the desired mutation. Baculovirus was generated as described in the Bac-to-Bac Baculovirus Expression System manual (Thermo, Waltham, MA, USA). In short, pFastBac vectors carrying the sequence of interest were transformed into DH10EMBacY *Escherichia coli* to generate the recombinant bacmid. Sf-9 insect cells were then transfected with the recombinant bacmid to generate the recombinant baculovirus. Production of the recombinant actin wild type (WT) or mutant was started by infecting 1.8 × 10^6^ cells/mL with 1:50 of the corresponding virus stock. Cells containing the protein of interest were harvested 3 days post infection and stored at −80°C until used for purification.

#### Purification of recombinant cytoskeletal actin WT and mutants

Recombinant human cytoskeletal actin WT and mutants were purified from Sf-9 insect cells using an actin-thymosin-β4-His_8_ fusion construct as described by Noguchi et al.^31^ In short, Sf-9 cells were resuspended in lysis buffer (10 mM Tris [pH 7.8], 5 mM CaCl_2_, 1.25% Triton X-100, 1 mM ATP, 100 mM KCl, 7 mM β-mercaptoethanol, 1 mM phenylmethylsulfonyl fluoride [PMSF], 100 μg/mL *N*_α_-*p*-tosyl-L-arginine methyl ester, 80 μg/mL *N*-tosyl-L-phenylalanine chloromethyl ketone, 2 μg/mL pepstatin, and 5 μg/mL leupeptin) and sonicated to disrupt the cells. The lysate was cleared by centrifugation and the supernatant incubated with 2 mL of lysis-buffer-equilibrated Pure Cube NiNTA column material (Cube Biotech, Monheim am Rhein, Germany) per liter of expression culture for 2 h, rotating at 4°C. The material was washed with 25 column volumes of wash buffer 1 (10 mM Tris [pH 7.8], 5 mM CaCl_2_, 10 mM imidazole, 200 mM KCl, and 1 mM ATP) followed by 25 column volumes of wash buffer 2 (10 mM Tris [pH 7.8], 5 mM CaCl_2_, 10 mM imidazole, 50 mM KCl, and 1 mM ATP). The protein was eluted with 250 mM imidazole, and the purity of the eluate was verified via SDS-PAGE. Fractions that contained the majority of the protein were pooled and dialyzed against G-buffer (10 mM Tris [pH 7.8], 0.2 mM CaCl_2_, 0.1 mM dithiothreitol [DTT], and 0.1 mM ATP) overnight to remove imidazole. The dialyzed sample was then digested with 1:300 weight/weight of α-chymotrypsin from bovine pancreas (Merck, Darmstadt, Germany) to remove the thymosin-β4-His_8_ moiety including the linker to yield the pure actin with native N and C termini. The reaction was quenched after a minimum of 45 min by the addition of 1 mM PMSF. The exact time of digest strongly depends on the age of the used batch of α-chymotrypsin. The sample was concentrated to 10–15 mg/mL, and polymerization of actin was induced by the addition of 100 mM KCl and 5 mM MgCl_2_. The polymerization reaction was incubated for at least 3 h at room temperature and then moved to 4°C overnight. On the following day, the F-actin was sedimented by centrifugation at 130,000 × *g* for 1 h at 4°C. The pellet was washed with G-buffer and finally resuspended in 0.5–1 mL of G-buffer using a Dounce homogenizer. The sample was dialyzed against a total of 5 L of G-buffer supplemented with 0.1 mM PMSF over 4 days. The buffer was changed at least three times over that period. After dialysis, the protein sample was centrifuged at 15,000 × *g* for 15 min to remove precipitate. The pure protein was flash frozen in liquid nitrogen and stored at −80°C.

#### Assays probing polymerization and depolymerization of actin

Pyrene-actin-based assays to monitor polymerization and depolymerization of actin filaments were performed as previously described^32^^,^^33^ with some slight modifications. To determine the rate of actin polymerization, Mg^2+^-ATP-G-actin was supplemented with 5% pyrene-labeled Mg^2+^-ATP-G-actin (WT) as a tracer to a final concentration of 10 μM. 20 μL of this solution was placed in a black flat-bottom 96-well plate (BrandTech Scientific, USA). The polymerization reaction was monitored as a function of increasing pyrenyl fluorescence in a Synergy 4 microplate reader (BioTek Instruments, Winooski, USA) using the built-in filter set (excitation 340/30 nm, emission 400/30 nm). Polymerization was induced by applying 80 μL of 1.25× polymerization buffer to a final concentration of 10 mM Tris (pH 7.8), 100 mM KCl, 5 mM MgCl_2_, 0.5 mM EGTA, 0.1 mM DTT, and 0.1 mM ATP using the built-in pipetting function. To determine the rate of depolymerization, Mg^2+^-ATP-G-actin was polymerized at 20 μM in the presence of 5% pyrene-labeled Mg^2+^-ATP-G-actin (WT) overnight at 4°C. 3 μL of the F-actin solution was placed in a black flat-bottom 96-well plate and rapidly diluted by applying 297 μL of G-buffer (10 mM Tris [pH 7.8], 0.2 mM CaCl_2_, 0.1 mM DTT, and 0.1 mM ATP). The dilution-induced depolymerization of the actin filaments was monitored using the settings mentioned above. The apparent half-time of the polymerization and depolymerization reaction was calculated by applying a single-exponential fit to the kinetic traces.

#### Thermofluor assay

Thermal stability of WT and mutant proteins was assessed using the Thermofluor assay. The assay utilizes the fluorescent dye Sypro Orange, which shows an increase in fluorescent intensity upon binding to hydrophobic core regions of proteins that become exposed during thermal denaturation. Ca^2+^-ATP-G-actin was converted to Mg^2+^-ATP-G-actin by incubation with magnesium-exchange buffer (10×, 10 mM EGTA and 1 mM MgCl_2_) for 2 min on ice prior to the experiment. 0.2 mg/mL Mg^2+^-ATP-G-actin and 5× Sypro Orange (Life Technologies, Carlsbad, USA, stock: 5000×) were mixed in assay buffer (10 mM Tris [pH 7.8], 0.1 mM MgCl_2_, 1 mM EGTA, 0.1 mM DTT, and 0.1 mM ATP) to a final volume of 25 μL. The samples were placed in a MicroAmp 48-well plate (Applied Biosystems, Waltham, MA, USA). The change in fluorescence intensity over a linear temperature gradient (1°C/min) was measured in a StepOne Real-Time PCR System (Applied Biosystems). The melting temperature was derived from the peak value of the first derivative of the melting function.

#### Nucleotide exchange assay

The rate of nucleotide exchange of Mg^2+^-ATP-G-actin was determined as previously described^34^ using the fluorescent ATP analog ε-ATP (Jena Bioscience, Jena, Germany).

## Results

### *ACTB* and *ACTG1* variants have dissimilar population profiles

**Figure 1:**
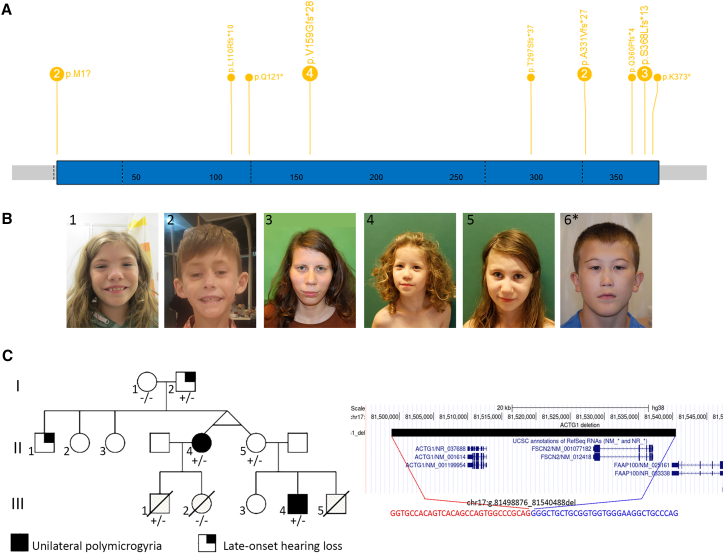
Definition of the haploinsufficiency of *ACTB* and *ACTG1* (A) Number and distribution of truncating variants (start loss, stop gain, frameshift) in *ACTB*. (B) Facial gestalt in individuals with the *ACTB* whole-gene deletions (1 and 2) and familial presentation of the *ACTB* stop gain (3–5). Note the resemblance of the facial gestalt of the last individual (6), although he carries a missense variant in *ACTB*. (C) Segregation and intrafamilial variability of microdeletion of *ACTG1* with the pedigree with the affected aunt (II.4) and nephew (III.4) with unilateral polymicrogyria on the left; +/− indicates the carrier status. Right: the UCSC genome hg38 browser view demonstrating a microdeletion encompassing *ACTG1* and *FSCB2*; coordinates of the last normal probes are shown in red with the precise breakpoint mapping in blue (first and last missing bases), including junction sequence shown below.

To study the profile and consequences of the variants in the NMA genes, we simulated all possible *ACTB* and *ACTG1* single-nucleotide changes and compared them with known variants from population databases, representing ∼431,130 individuals.^15^^,^^16^^,^^17^ Simulated variant counts per gene were similar overall, but we observed several differences between the non-synonymous, synonymous, and non-coding population variant counts of the two genes, which are unlikely to be the result of differences in mutation acquisition potential (Figures S2 and S3; Table S1). For example, the population dataset included only one individual with pLoF *ACTB* variant (probability of LoF intolerance [pLI] = 0.99) but >20 individuals with truncating *ACTG1* variants (pLI = 0)^35^ (Figures 1A and S2A). Both genes are highly constrained for population MVs (Table S1A), but *ACTB* is more intolerant (43 distinct MVs, 0.02% population frequency) than *ACTG1* (149 MVs, 0.05% population frequency) (Table S1).^35^

Next, we compiled clinical information from 290 individuals (125 new) with *ACTB* or *ACTG1* variants classified as P/LP^36^ in various diagnostic laboratories in multiple countries. This “NMA cohort” comprised 275 individuals with SNVs in *ACTB* (*n* = 145) or *ACTG1* (*n* = 130) and 15 individuals with small deletions removing the entire *ACTB* or *ACTG1* gene (Table S2). The cohort’s age range spanned 20 weeks gestation to 60 years, with 54% males and 46% females. The phenotype profiles of individuals in the NMA cohort, as expected, were highly variable.

*ACTB* and *ACTG1* variants, therefore, have remarkably different population profiles. These observations support their different biological roles in humans. Our initial examination of the clinical data revealed impressive heterogeneity, indicating the need for systematic in-depth analysis.

### *ACTB* and *ACTG1* LoF alleles have distinct clinical consequences

We devised a variant and phenotypic-led approach to study the clinical consequences of variations in the two genes (Figure S1). We first analyzed pLoF variants in our NMA cohort (Figure 1A). Previously, two conditions have been described to result from *ACTB* pLoF variants, i.e., a “pleiotropic developmental disorder” due to 7p22.1 chromosomal microdeletions or point variants,^10^ which is included within BWCFF1 (#243310) in OMIM; and syndromic thrombocytopenia caused by point variants in the last exon, which has a separate OMIM entry (#620475).^4^ We compared the phenotypes of individuals either with microdeletions encompassing *ACTB* or with individuals’ *ACTB* pLoF variants expected to undergo or escape nonsense-mediated decay (NMD) (Figure 1B and Table S2). Individuals with LoF variants predicted to escape NMD were initially analyzed separately but exhibited a phenotype comparable to those with alleles subject to NMD. Across all three groups, the clinical presentations were highly overlapping and included mild to moderate neurodevelopmental delay or borderline to mild intellectual disability, behavioral abnormalities, mild microcephaly, short stature, and brain anomalies (such as heterotopia but not pachygyria) as well as various congenital malformations and variable thrombocytopenia (Note S1).

In contrast, the NMA cohort did not have any P/LP *ACTG1* pLoF point variants. However, we identified seven individuals with chromosome 17q25 deletions shorter than 1 Mb that encompassed *ACTG1*. Deletion carriers showed variable phenotypes ranging from normal intelligence to borderline intellectual disability with additional structural anomalies (Table S2). This included a 41.6-kb deletion including *ACTG1* and *FSCN2* segregating through three generations in mildly affected and healthy relatives (Figure 1C). A unilateral polymicrogyria was observed in two affected family members, both of whom are high-functioning and show only mild neurodevelopmental involvement. *FSCN2* encodes the actin-bundling protein fascin-2 that crosslinks actin filaments into bundles within dynamic membrane protrusions, and its pLI score is 0, indicating that its haploinsufficiency alone is unlikely to cause a monogenic disorder.^15^

*ACTG1* shows a loss-of-function observed/expected upper bound fraction (LOEUF) of ∼0.62, indicating no marked depletion of pLoF. Considering the prevalence of the *ACTG1* pLoF variants in the population datasets, *ACTG1* pLoF point variants are unlikely to be pathogenic. Chromosome 17q25.3 deletions involving *ACTG1* and flanking genes could cause a contiguous gene deletion syndrome with variable penetrance, which requires further studies.

### *ACTB* or *ACTG1* missense and in-frame variants result in multiple NMAs

**Figure 2:**
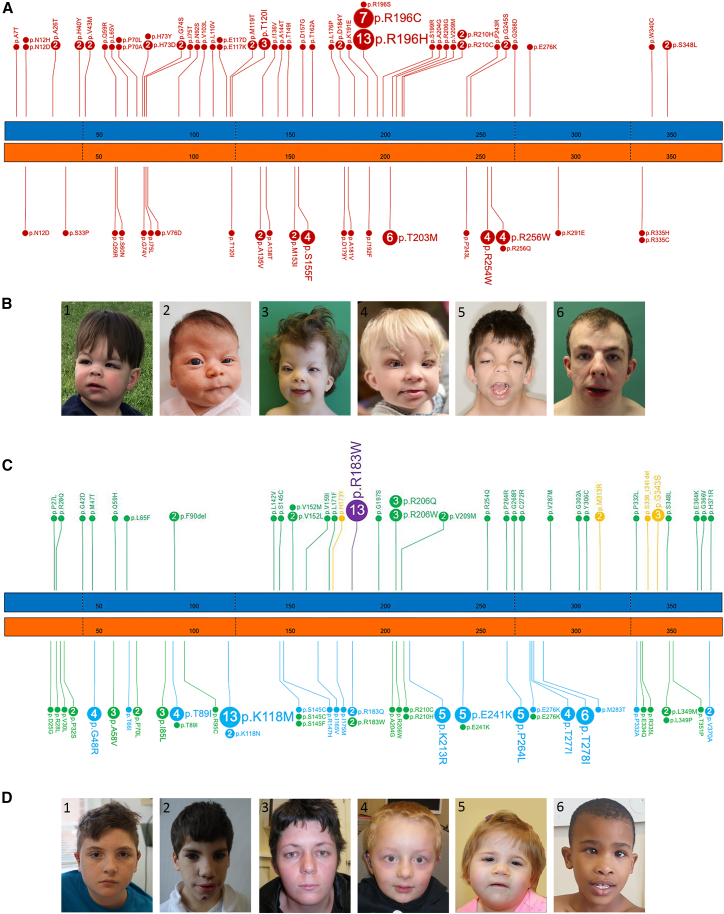
Missense variants in *ACTB* and *ACTG1* result in several distinct disorders (A) Number and distribution of missense variants in *ACTB* and *ACTG1* resulting in the Baraitser-Winter cerebrofrontofacial syndrome (BWCFF); *ACTB* (blue bar) and *ACTG1* (orange bar) with every 50th amino acid numbered in both gene models. The borders of coding exons are marked with dashed lines. (B) Representative facial gestalt in individuals with the BWCFF syndrome. Shown are individuals #1 (180-G), #2 (194-G), #3 (23-B), #4 (ID_09), #5 (47-B), and #6 (45-B), with subjects’ IDs corresponding to those listed in Table S2. Note that individuals #5 and #6 carry the same *ACTB* hotspot variant at position Arg196, and that individuals in images 5 and 6 carry the same *ACTB* hotspot variant at position Arg196. (C) Number, distribution, and clinical consequence of the missense variants in *ACTB* and *ACTG1* not resulting in BWCFF; gene models and amino acid numbering are as described above. (D) Representative facial gestalt in individuals with unspecified non-muscle actinopathies (unNMA) due to variants in *ACTG1* (1–4) and *ACTB* (5 and 6). Note that images 3 and 4 show affected individuals from the same family. Shown are individuals #1 (35-G), #2 (119-B), #3 (47-G), #4 (48-G), #5 (20-B), and #6 (193-G), with subjects’ IDs corresponding to those listed in Table S2.

Next, applying a structured classification strategy (Figure S1) on individuals with missense or in-frame variants in the NMA cohort, we identified 73 individuals whom we categorized into *ACTB-*BWCFF1 and 40 with *ACTG1-*BWCFF2 (Figures 2A and 2B). BWCFF can be diagnosed in an individual with a (likely) pathogenic MV in *ACTB* or *ACTG1* if this individual presents with (1) the specific facial dysmorphism (typically including hypertelorism, high-arched eyebrows, ptosis, long palpebral fissures with everted lower lid, broad nasal tip, long smooth philtrum, large mouth with thin upper lip, grooved chin, and large vertically oriented ears) and/or (2) frontal predominant pachygyria. Expert consensus description of the specific features is summarized in Note S2.

Sixty individuals presented with *ACTG1-*isolated hearing loss (HL) and 13 with *ACTB-*DDS1. Detailed inspection of the clinical data of the remainder showed that eight individuals with five *ACTB* MVs/in-frame indels presented with features similar to those of individuals with *ACTB* pLoF variants (Figure 1B1–1B6). Here, the main criterion was the overlap of the subjects’ facial gestalt with that observed in individuals carrying deletions encompassing *ACTB* or variants predicted to result in NMD. These defining features are detailed in Note S1. Importantly, none of these individuals presented with the frontal predominant pachygyria, a clinical symptom specific for BWCFF.

The remaining 65 individuals exhibited heterogeneous phenotypes that were not compatible with any of the previously mentioned actinopathies and did not show a recognizable facial gestalt even if some individuals presented with minor facial anomalies (grouped as unspecified non-muscle actinopathy or unNMA) (Figures 2C and 2D). We suggest that more distinct entities will be identified within this cohort in the future.

This classification revealed detailed clinical characteristics and variant spectrum for each NMA subtype (Notes S1–S5).

Note that multiple congenital malformations; e.g., heart, urinary tract, skeletal, and gastrointestinal anomalies, were more prevalent in the BWCFF group but were also observed in individuals with *ACTB* LoF and unNMA. Apart from rare exceptions, we found that individuals with the same variants generally had highly overlapping clinical features and were classified into the same NMA subtype. Notably, however, we detected substantial differences in severity within the same subtype. We found the variant spectra to be much larger than previously recognized for all NMAs, apart from *ACTB-*DDS1, which seems to be caused only by *ACTB*:p.Arg183Trp. Interestingly, we observed that for certain positions, the same substitution in *ACTB* or in *ACTG1* can cause different NMAs.

These data show that *ACTB* or *ACTG1* protein-altering variants (PAVs) result in multiple NMAs. While specific variants exhibit reproducible genotype-phenotype correlations, we recognize that not all pathogenic variants in *ACTB* or *ACTG1* will necessarily act with similar predictability, and that phenotypic outcome may in some cases depend on variation at other loci and/or environmental influences.

Notably, the broad spread of PAVs across most NMAs suggests that these variants (with a possible exception of *ACTB*:p.Arg183Trp [c.547C>T]) are unlikely to be gain-of-function.^5^ The differences of phenotypes of most *ACTB*/*ACTG1* PAVs versus LoF variants indicate that these missense/in-frame variants are unlikely to result in simple LoF. Hence, most of these variants possibly act in dominant-negative manner or result in loss of different subfunctions.^37^ The heterogeneity of associated clinical features suggests that their cellular consequences could be context dependent.

### Genetic and clinically led classification of NMAs is accurate and has important clinical implications

**Figure 3:**
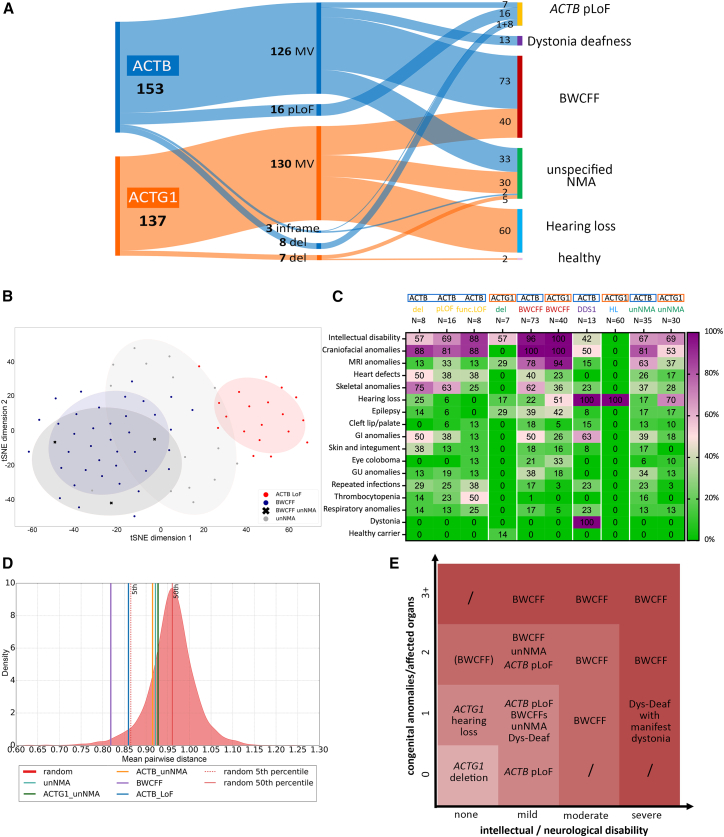
Broad clinical spectrum of non-muscle actinopathies (A) Distribution of 290 pathogenic variants in *ACTB* and *ACTG1* within different disorders of NMA spectrum. BWCFF, Baraitser-Winter cerebrofrontofacial syndrome; del, whole-gene deletion; MV, missense variant; pLoF, putative loss of function. (B) t-SNE visualization of three major NMA phenotypes in GestaltMatcher analysis. NMA-BWCFF indicates three individuals who were not reliably classified as BWCFF or unNMA based on the facial gestalt. Note that GestaltMatcher analysis allocated all three individuals within the BWCFF group. (C) Phenogrid demonstrating frequencies of selected clinical features in individuals with pLoF in *ACTB*, microdeletions encompassing *ACTB* (*ACTB* del) or *ACTG1* (*ACTG1* del), and non-truncating *ACTB* variants resulting in unstable protein (*ACTB* func.LoF), BWCFF, dystonia-deafness syndrome (DD), hearing loss (HL), and unspecified non-muscle actinopathy (unNMA). Individuals with incomplete clinical information were counted as “clinical feature not present” except for gastrointestinal (GI) anomalies. For GI anomalies, individuals with unknown status were excluded from the calculation. The presence of dystonia was analyzed in individuals older than 18 years. (D) Phenotypic similarity between subgroups of subjects. To assess whether individuals within each subgroup are phenotypically coherent, we simulated a control distribution by repeatedly computing the mean pairwise distance in batches of randomly sampled individuals from the GestaltMatcher database (i.e., individuals with different syndromes). Each subgroup’s mean within-group distance *d*(*C*) was then compared to this control distribution. The BWCFF subgroup showed the strongest intra-group similarity (*d* = 0.82, 1.61st percentile), followed by *ACTB* LoF (*d* = 0.86, 4.35th percentile). The unNMA-based subsets were less extreme but still below random expectation: *ACTB*_unNMA *d* = 0.91 (15.90th percentile), unNMA *d* = 0.92 (18.95th percentile), and *ACTG1*_unNMA *d* = 0.93 (22.20th percentile). These results indicate that BWCFF and *ACTB* LoF groups exhibit pronounced within-group similarity, whereas the unNMA subsets show modest yet non-random clustering relative to random controls. (E) Severity grading of the disorders within the non-muscle actinopathy spectrum based on the number of congenital anomalies and/or affected organs and the severity of intellectual impairment.

To test our clinically led classification (Figure 3A), we subjected our conclusions to objective validation. First, objective analysis of the craniofacial features using the photographs available to us using GestaltMatcher^22^^,^^38^ confirmed our classification orthogonally (Figures 3B, 3D, S4, and S5; Table S5). Next, we compared the clinical features of all the NMAs (Figure 3C) and mapped the summarized trends in the severity of intellectual/neurological and the number of congenital anomalies or affected organs (Figure 3E). This revealed an extraordinarily broad clinical spectrum, ranging from individuals who appear to be unaffected (e.g., small chromosomal deletions across *ACTG1*) or relatively mildly affected (e.g., *ACTB* pLoF variants) to individuals who die *in utero*.

These results show that our genetic and clinically led classification of NMAs is reasonably accurate, and that the clinical differences between NMAs have implications for their accurate diagnosis, management, prognosis, and surveillance (expert opinion recommendations are summarized in Notes S1–S5).

### *ACTB* MVs associated with LoF phenotype result in abnormal folding or impact actin thermal stability

**Figure 4:**
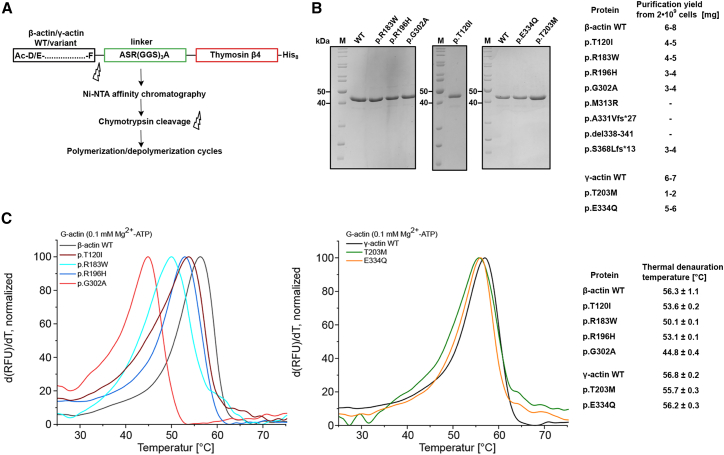
*ACTB* MVs associated with LoF phenotype result in abnormal folding or impact actin thermal stability (A) Schematic of the purification strategy for recombinant human βCYA and γCYA (adapted from Greve and Manstein^40^). (B) SDS gels show purified WT and variant actin isoforms with the exact purification yield listed in the right panel; note three recombinant βCYA mutant variants could not be purified. Examples of the immunoblot of Sf9 insect cell lysate are presented in Figure S4. (C) Differential scanning fluorimetry was used to assess the thermal stability of Mg^2+^-ATP-G-actin. Representative traces of experiments with βCYA WT and variants (left) and γCYA (right) in the presence of 0.1 μM Mg^2+^-ATP. Data are shown as the first derivative of the obtained protein melting traces. The point of thermal denaturation (*T*_M_) is determined from the peak of the function. Final concentration of actin is 0.2 mg mL^−1^. Four independent experiments were performed. Means and SD are shown in the right panel.

Eight individuals with five *ACTB* MVs/in-frame indels presented with clinical features similar to those of individuals with *ACTB* pLoF variants. According to SpliceAI^39^ predictions, none of these MVs are expected to affect *ACTB* splicing. Therefore, we hypothesized that some *ACTB* MVs might result in protein instability and degradation. We generated recombinant mutant βCYA proteins for *in vitro* characterization of a selection of these variants (Figure 4A). Protein purification could not be achieved for βCYA with two variants (p.Met313Arg [c.938T>G] or p.Ser338_Ile341del [c.1012_1023del]; Figure S6), indicating a possible defect with protein folding and/or stability. In contrast, other tested variants could be purified in sufficient quantity (Figure 4B). Among these, one variant (p.Gly302Ala [c.905G>C]) showed a significantly reduced stability (Figure 4C). We confirmed the previously observed mild reduction in stability for p.Arg183Trp^34^ and detected no further differences in thermal stability between the wild-type and the remaining four MVs analyzed in this study (Figure 4D).

Using dermal fibroblasts from individuals with *ACTB*-pLoF disorder, we quantified the amount of βCYA and γCYA produced in the affected individuals’ cells by western blot. Intriguingly, we did not observe a significant reduction of βCYA production in cells with *ACTB* deletion and LoF variant, nor with the variants with reduced thermal stability (Figure S7).

Summarizing our data, we suggest that the *ACTB*-related “pleiotropic developmental disorder”^10^ is distinct from BWCFF1. The phenotype previously described as “syndromic thrombocytopenia”^4^ substantially overlaps with the *ACTB*-related pleiotropic developmental disorder. In the expanded cohort, we observed a shared facial gestalt and confirmed thrombocytopenia in individuals with *ACTB* deletions, supporting that these entities represent a single clinical spectrum that should be merged under the term “*ACTB* pLoF disorder.” The *ACTB* pLoF-associated disorder may involve three molecular mechanisms: (1) loss of *ACTB* transcription due to deletion of one allele, (2) NMD of mRNA containing a premature stop codon, and (3) production of unstable βCYA resulting from selected missense or in-frame *ACTB* variants.

### Actin polymerization and depolymerization dynamics are significantly altered in BWCFF syndrome variants

**Figure 5:**
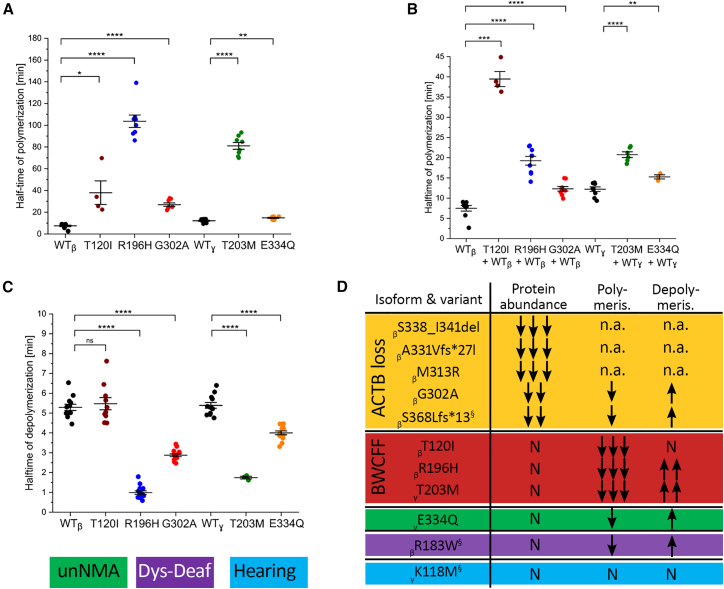
NMA variants result in diverse polymerization anomalies (A) Salt-induced actin polymerization assessed by pyrene assay. Scatterplot demonstrates distribution of the measured half-times from the individual experiments with 2 μM βCYA WT, γCYA WT, and mutant isoforms. Means and SD are indicated; representative traces are shown in Figure S14. (B) Same experiment as in (A) repeated with a 1:1 mixture of the WT and mutant proteins. (C) Dilution-induced actin filament depolymerization assessed by pyrene assay. Scatterplot demonstrates distribution of the measured half-times from the individual experiments with 20 μM F-actin that is rapidly diluted to 0.2 μM to induce depolymerization. Representative traces are shown in Figure S14. (D) Summary of the actin mutants characterized in the current and previous studies. Previously published mutants are marked with a silcrow with the following references: βS368Lfs^∗^13,^33^ βR183W,^34^ and γK118M.^42^ n.a., not assessable.

Next, we set out to understand the mechanistic basis of BWCFF. Using selected recombinant proteins, we investigated the potential impacts of CYA variants on actin dynamics. *ACTB*:T120I (BWCFF1), *ACTB*:R196H (BWCFF1), and *ACTG1*:T203M (BWCFF2) could be successfully purified with the thermal stability similar to the purified control isoforms (Figure 4). We observed a striking decrease in filament polymerization rates and faster depolymerization in the cases of *ACTB*:R196H and *ACTG1*:T203M (Figures 5A and S11). All tested mutants formed co-filaments with WT protein. The presence of 50% WT protein in the polymerization experiments attenuated the observed defect in all but one mutant (Figure 5B). Actin filaments containing both *ACTB*:T120I and WT proteins showed the same polymerization rate as the pure *ACTB*:T120I mutant. In comparison, we observed only minor alterations in the polymerization and depolymerization kinetics of one unNMA-associated variant *ACTG1*:E334Q.^41^ Similarly, nearly normal dynamics were demonstrated for the ACTB:R183W, ACTB:E364K,^34^ and ACTG1:K118.^42^

Collectively, we observed significant changes in the polymerization and depolymerization dynamics of the selected BWCFF variants, a biochemical property specifically associated with BWCFF and not observed in other NMA phenotypes (Figure 5D).

The amount of βCYA and γCYA in the primary fibroblasts from individuals with BWCFF did not differ from the actin amount in the healthy control cell lines (Figures S7–S10). Interestingly, we observed no significant differences in the transcriptional profiles of the several BWCFF1/2, DDS1, and unNMA fibroblast cell lines (Figures S12 and S13).

### *In silico* prediction tools require caution in clinical applications with novel actin variants

The identification of novel variants in *ACTB* or *ACTG1* classified as (likely) pathogenic according to ACMG criteria still necessitates a thorough evaluation of their clinical association. This can be particularly challenging in individuals with limited clinical data, such as those undergoing prenatal or newborn testing. A clear correlation between the location of variants in the actin molecule and their associated phenotypic outcomes is crucial for predicting the phenotypic outcome of novel variants. Therefore, we mapped all identified disease-associated single amino acid substitutions in βCYA and γCYA onto the corresponding structures to analyze possible correlations between the variant’s location within the three-dimensional structure of actin and the phenotype. We observed no clear phenotype-specific accumulation of variants in specific regions of the actin molecule (Figure S15). Next, we analyzed the location of the variants in correlation with regions located on the filament surface (1), involved in actin-actin interaction (2), interaction with specific actin-binding proteins (profilin [3], cofilin [4], myosin [5], and tropomyosin [6]), phosphate, and Mg^2+,^as well as nucleotide coordination (7) (Figure S15). We found that DD-associated R183W affects one of the key residues of the nucleotide-binding site. Actin-cofilin, actin-profilin, and actin-myosin interaction sites were enriched for PVAs associated with unNMA and HL. However, this is a preliminary observation that is worth further exploration, but it is insufficient to be applied as an ACMG criterion for variant pathogenicity.^36^ Multiple *in silico* tools generally classify most rare actin variants as pathogenic; however, they do not allow any allocation to a specific phenotype, emphasizing the importance of the experimental characterization of the mutant proteins.

## Discussion

The combination of large datasets, clinical studies, and complementary biochemical studies led to a coherent functional classification of the NMA spectrum. It finally delineated eight distinct NMAs, demonstrating extraordinary pleiotropy and a spectrum of disease severity associated with *ACTB* and *ACTG1* variants. Based on our data, NMAs can be categorized into five clinical entities and three major functional groups (Figure S16). We hypothesize that this remarkable number of conditions associated with the two genes reflects the large number of cellular interactions of CYAs, with downstream effects being context dependent and clinical consequences being highly variant specific. This delineation of disorders has direct implications for the diagnosis and treatment of affected individuals, showing the power of large, systematically collected cohorts in rare diseases.

**Figure 6:**
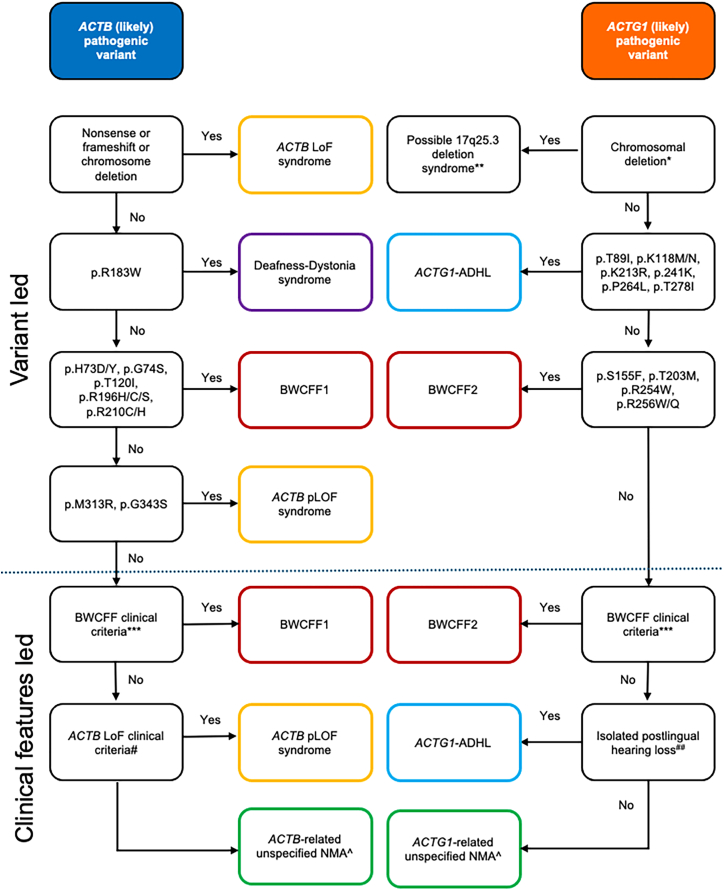
Diagnostic workflow supporting clinical interpretation of ACTB/ACTG1 variants ^∗^ACTG1 nonsense or frameshift variants are likely to be classified as benign by ACMG criteria. ^∗∗^Further studies are required to prove the existence of this disorder; deletions usually involve additional flanking genes and likely have variable penetrance. *^∗∗∗^*Specific facial dysmorphism (typically including hypertelorism, high-arched eyebrows, ptosis, long palpebral fissures with everted lower lid, broad nasal tip, long smooth philtrum, large mouth with thin upper lip, grooved chin, and large vertically oriented ears) and/or frontal predominant pachygyria with or without other brain and inner organ malformations or minor anomalies. ^*#*^Specific facial dysmorphism typical for *ACTB* LoF (long face, straight eyebrows, deep-set eyes, epicanthus, narrow or flat nasal bridge with broad nasal tip, and large mouth) without pachygyria; ∧Potential revision of the clinical diagnosis during follow-up.

The clinical association of the novel variant in *ACTB* or *ACTG1* might be challenging and requires an accurate assessment of the complete clinical data (expert opinion in Notes S1–S5). Figure 6 shows a diagnostic workflow that guides the clinical classification of individuals with variants in *ACTB* and *ACTG1*. The clinical decision about the type of NMA is heavily based on the evaluation of the facial gestalt and requires previous expertise. Figures 1 and 2 show representative images for ACTB LoF disorder, BWCFF, and the variability of unNMA phenotypes. We recommend complementing the clinical evaluation with an assessment of the individual’s facial gestalt using AI-based phenotyping tools.

While the application of the clinical diagnosis “unspecified NMA” may not fully meet the needs of affected individuals due to its lack of precision, we consider its use valuable for distinguishing this group from other phenotypes. It is essential, however, that individuals and families are informed about the provisional nature of this designation and are offered regular follow-up, as more refined clinical definitions may become available in the future.

Detailed biochemical characterization of disease-associated variants in βCYA and γCYA is currently limited to a few reports.^33^^,^^34^^,^^41^^,^^42^^,^^43^^,^^44^^,^^45^ A recent study investigated the substitutions at the position R196 in βCYA, confirming the reduced polymerization rate and faster depolymerization not only in the presence of R196H but also for R196C and R196S variants.^40^ Moreover, this work demonstrates a significant reduction of the actin-Arp2/3 interaction, as well as isoform-specific defects in the actin-myosin interaction. As BWCFF-associated variants are scattered throughout the whole length of both actin isoforms, we expect that nearly every variant might have its own additional variant-specific, either more or less pronounced impact on actin-ABP interaction in addition to the perturbed actin dynamics that we suggest being a common molecular mechanism in BWCFF. This might be one of the factors contributing to the broad clinical variability of BWCFF.

In contrast to the striking biochemical abnormalities, we observed no significant changes in either actin abundance or transcriptional profiles of the cell lines derived from affected individuals. Patient-derived skin fibroblasts provide a readily accessible and genetically matched cellular model widely used for functional studies in rare genetic diseases.^46^^,^^47^ However, this is not a primarily affected tissue in individuals with any type of NMAs and represents a very robust cellular system that might require specific stimuli to unmask the impact of *ACTB* and *ACTG1* variants. Actin has multiple tissue-specific functions, and it is possible that expression profiles in different cell types of the same individuals might be significantly abnormal. These results emphasize that *in vitro* modeling and functional validation of actin variants require a careful choice of the cellular system.

Although both βCYA and γCYA are among the most abundant cytoplasmic proteins, they also function in the nucleus.^48^^,^^49^ Though not addressed in this work, the dysfunction of the nuclear actin might be an additional disease mechanism at least for a part of the NMA spectrum. What is even more intriguing is that some actin functions might be regulated not at the protein level but at the level of the nucleotide sequence.^50^^,^^51^ Biallelic knockout of Actb in mice is lethal.^52^ In contrast, mice engineered to express γCYA from the Actb gene were viable, fertile, and largely unremarkable, except for degenerative changes in the inner ear and retina during aging.^53^^,^^54^ Coding sequences of the *ACTB* and *ACTG1* may have independent functional significance, regulating the transcription patterns and translation dynamics of both actin isoforms, which results in different ribosomal densities specific to each actin isoform.^55^ Our cohort comprises 259 individuals with variants that alter the coding sequences of *ACTB* or *ACTG1*. Although we could demonstrate the impact of selected PAVs at the protein level, we did not address the potential impact of these and other variants on the translational dynamics of actin mRNA. This aspect, addressed in future studies, might shed light on the nucleotide-dependent functionalities of actin and explore an additional facet in the molecular pathogenesis of NMAs.

Neurodevelopmental and neurological features are the predominant characteristics of many NMAs. This is in line with the known importance of CYAs for neurodevelopment, neural function, and memory.^56^^,^^57^ Variants in several other genes that control cytoskeletal dynamics, such as *DIAPH1* (MIM: 602121), *RAC1* (MIM: 602048), and *CYFIP* (MIM: 617978), have been shown to result in neurodevelopmental phenotypes similar to those of NMAs. Our findings underscore the growing appreciation of the significance of fine-tuned cytoskeletal control and dynamics in normal development. Furthermore, understanding of actin dynamics in the neurodevelopmental context is of greater importance, as several other monogenic neurodevelopmental disorders are caused by variants in genes encoding actin regulators and interactors.^58^^,^^59^^,^^60^^,^^61^^,^^62^^,^^63^ The actin-ABP network appears particularly susceptible to variant-specific effects, as several of these genes result in allelic disorders.^64^^,^^65^ Studies in patient-derived fibroblasts are inherently limited by their inability to capture the organ-specific actin dynamics critical to the pathogenesis of NMA-related NDDs. Neuronal cells derived from patient-specific pluripotent stem cells provide a more physiologically relevant model for studying these mechanisms.^66^ Moving forward, such systems should be systematically employed to investigate neuronal function across distinct NMA phenotypes, enabling a deeper understanding of genotype-phenotype correlations and disease-specific pathomechanisms.

In conclusion, the present work improves the diagnosis and management of individuals affected by NMA and raises caution regarding the clinical interpretation of novel coding and non-coding variants in *ACTB* and *ACTG1*. These results also reflect the considerable amount of variant-level collaborative clinical trials and multi-modal mechanistic studies that may be required to realize the promise of precision medicine for rare allelic disorders. While multiplexed assays for variant effect (MAVEs) may provide valuable functional insights, the complexity of NMA phenotypes is unlikely to be resolved by a single experimental approach. Assays measuring β-actin protein stability are particularly useful for identifying likely *ACTB* LoF variants, and polymerization/depolymerization assays have proven informative for variants causing BWCFF. Other phenotypes, however, remain mechanistically unresolved and will require further molecular characterization to define suitable functional assays. Beyond MAVEs, simple model organisms such as *Caenorhabditis elegans* offer an attractive and scalable system to dissect the molecular mechanisms of actinopathies and to validate variant effects in a physiological context. An initial proof of concept showed that expression of human actin variants associated with severe or lethal phenotypes in individuals results in comparable phenotypic severity in *C. elegans*, underscoring the potential of this model to functionally stratify actinopathies.^67^ Together, these complementary strategies highlight the progress toward integrating molecular and clinical data to refine variant interpretation in non-muscle actinopathies.

## Data and code availability

Datasets supporting the results of this article are available in the supplemental information or can be requested from the corresponding authors.

## Consortia

Members of the NMA clinical consortium are Andrea Accogli, Maria Albers, Fowzan Alkuraya, Neophytos Apeshiotis, Diana Baralle, Carmen Barba, Allan Bayat, Andreas Benneche, Laura Bernardini, Saskia Biskup, Nina Bögershausen, Knut Brockmann, Nicola Brunetti-Pierri, Peter Burfeind, Ruben Cabanillas, Patricia Corriols-Noval, Elke de Boer, Iris de Lange, Charulata Deshpande, Marta Diñeiro, Emily Doherty, Julia Doll, Sofia Douzgou, Tracy Dudding-Byth, Nadja Ehmke, Katherine Fawcett, Carlos R. Ferreira, Jan Fischer, Joel Fluss, Rocío González-Aguado, Luitgard Graul-Neumann, Andrew Green, Renzo Guerrini, Asya Gusina, Ute Hehr, Maja Hempel, Michaela A.H. Hofrichter, Ivan Ivanovski, Diana Johnson, Marieke Joosten, Silke Kaulfub, Tjitske Kleefstra, Eva Klopocki, Karla Krause, Alma Kuechler, Maria Kuzyakova, Martin W. Laass, Augusta Lachmeijer, Wayne Lam, Cha Gon Lee, Yun Li, Vanesa López-González, Karen Low, Michael Lyons, Carlo Marcelis, Francisco Martinez-Castellano, Maarten Massink, Kay Metcalfe, Donatella Milani, Shahida Moosa, Manuela Morleo, Teresa Neuhann, Thomas Neumann, Huu Nguyen, Vincenzo Nigro, Nuha Nimeri, Ewa Obersztyn, Anne O’Donnell, Carmen Orellana, Estrella Pallas, Hans-Jürgen Pander, Elena Parrini, Silke Pauli, Michele Pinelli, Lina Quteineh, Julia Rankin, Monica Rosello, Tamanna Roshan Lal, Vincenzo Salpietro, Jens Schallner, Gregor Schlüter, Julia Schmidt, Mariasavina Severino, Vandana Shashi, Corinna Siegel, Margie Sinnema, Anne Slavotinek, Sarah Smithson, Siddharth Srivastava, Rikke Steensbjerre Møller, Maja Svrakic, Lindsay Swanson, Hannah Thomson, Eduardo Tizzano Ferrari, Annalaura Torella, Irene Valenzuela Palafoll, Yolande van Bever, Ellen van Binsbergen, Marjon van Slegtenhorst, Nienke Verbeek, Virginie Verhoeven, Barbara Vona, Dagmar Wahl, Luisa Weiss, Gökhan Yigit, Maha Zaki, Telethon Undiagnosed Diseases Program, and Undiagnosed Diseases Network.

## Acknowledgments

N.D.D., P.D., M.H., and I.N. gratefully acknowledge support of the Core Facility at the NCT/UCC-CMTD Dresden and Stem Cell Engineering Core Facility of the CMCB Technology Platform at TU Dresden. D.J.M. and his lab gratefully acknowledge support provided by the Research Core Unit for Structural Biochemistry. Computing time was provided on supercomputers Lise and Emmy at NHR@ZIB and NHR@Göttingen as part of the Alliance for National High-Performance Computing (NHR) infrastructure. The calculations for this research were conducted with computing resources under the project ID nib00018. N.D.D. received grant support from the Deutsche Forschungsgemeischaft (DI 2170/3-1 and DI 2170/5-1) and 10.13039/501100003042Else Kröner-Fresenius-Stiftung (2020_EKES.04). N.D.D. and D.J.M. are supported through the European Union’s Horizon 2020 research and innovation program under the EJP RD COFUND-EJP no. 825575 with support from the 10.13039/501100002347German Federal Ministry of Education and Research under grant agreements 01GM1922A and 01GM1922B, respectively. D.J.M. acknowledges grant support from the 10.13039/501100001659Deutsche Forschungsgemeinschaft (MA1081/28-1). J.N.G. acknowledges the support provided by the PREPARE program for medical scientists from 10.13039/501100005624Hannover Medical School. A.S.W. and N.A.R. acknowledge grant support from 10.13039/501100000265Medical Research Council project grant MR/T016809/1; Medical Research Council - National Institute for Health and Care Research rare disease research platform MR/Y008340/1; and Kidneys for Life pump priming grant 2017. S.B. acknowledges grant support from the 10.13039/100014653NIHR Manchester Biomedical Research Centre (NIHR203308). S.B., A.S.W., and A.T. acknowledge support from the Davies family for grant support in the form of Marsh Studentship to the 10.13039/501100000770University of Manchester. S.B. and S. Cuvertino acknowledge support from 10.13039/501100001279Great Ormond Street Hospital Charity research grant V4621.

## Author contributions

Conceptualization, N.D.D., D.J.M., A.S.W., and S.B.; methodology, N.D.D., D.J.M., A.S.W., S.B., A.R., G.M., D.P., E.S., J.N.G., P.K., and M.H.; investigation, A.T., J.N.G., J.C., S. Calabro, S. Cathey, B.C., H.C., M.C., S. Cuvertino, P.D., K.E., A.E.F., L.G., S.H., W.G.J., I.K., P.M.-R., A. Meinhardt, I.N., I.R., F.S.S., A. Marquardt, K.T., S.T., J.V., A.V., B.W., T.-C.H., and M.S.; supervision, N.D.D., A.R., E.S., M.H.T., P.K., M.H., C.B.L., N.A.R., D.J.M., A.S.W., and S.B.; writing – original draft, N.D.D., D.J.M., A.S.W., and S.B.; writing – review & editing, all co-authors.

## Declaration of interests

The authors declare no competing interests.

## Web resources

FastQC, https://www.bioinformatics.babraham.ac.uk/projects/fastqc/
GestaltMatcher database, https://db.gestaltmatcher.org/
GraphPad, http://www.graphpad.com/
OMIM, https://www.omim.org/
PGG.SNV, https://pog.fudan.edu.cn/pggsnv/
Picard tools, http://broadinstitute.github.io/picard/
Trim Galore, http://www.bioinformatics.babraham.ac.uk/projects/trim_galore/

## Supplemental information

Document S1. Figures S1–S16, Tables S4 and S5, and Notes S1–S7

Table S1. Actin variant database

Table S2. Clinical registry

Table S3. Average gene-expression profiles

Document S2. Article plus supplemental information
