# Supplementary material for: Molecular genotype-phenotype correlation in ACTB- and ACTG1-related non-muscle actinopathies
Source: Am J Hum Genet. 2026 Jan 12;113(2):324–41. doi: 10.1016/j.ajhg.2025.12.007 (PMC13087417; doi:10.1016/j.ajhg.2025.12.007)
Supplement: Document S1. Figures S1–S16, Tables S4 and S5, and Notes S1–S7 [file mmc1.pdf]

The American Journal of Human Genetics, Volume *113*

Supplemental information

Molecular genotype-phenotype correlation in *ACTB*- and *ACTG1*-related non-muscle actinopathies

Nataliya Di Donato, NMA Consortium, Andrew Thom, Andreas Rump, Johannes N. Greve, Juan Cadiñanos, Rocco Salvatore Calabrò, Sara Cathey, Brian Chung, Heidi Cope, Maria Costales, Sara Cuvertino, Philine Dinkel, Kalliopi Erripi, Andrew E. Fry, Livia Garavelli, Sabine Hoffjan, Wibke G. Janzarik, Insa Kreimer, Grazia Mancini, Purificacion Marin-Reina, Andrea Meinhardt, Indra Niehaus, Daniela Pilz, Ivana Ricca, Fernando Santos Simarro, Evelin Schrock, Anja Marquardt, Manuel H. Taft, Kamer Tezcan, Sofia Thunström, Judith Verhagen, Alain Verloes, Bernd Wollnik, Peter Krawitz, Tzung-Chien Hsieh, Michael Seifert, Michael Heide, Catherine B. Lawrence, Neil A. Roberts, Dietmar J. Manstein, Adrian S. Woolf, and Siddharth Banka

Supplemental Materials

Contents

Note S1. Syndrome caused by *ACTB* pLoF variants 2
Note S2. Baraitser-Winter-Cerebrofrontofacial Syndrome 4
Note S3. *ACTB*:p.Arg183Trp-related dystonia-deafness syndrome 8
Note S4. *ACTG1*-associated isolated hearing loss (*ACTG1*-ADHL) 10
Note S5. Unspecified non-muscle actinopathies including *ACTG1*-associated isolated coloboma 11
Note S6. GestaltMatcher facial analysis.. 15
Note S7. Transcriptome sequencing 15
Supplemental Figures **17**
Figure S1. Classification of the NMA patient cohort applying genomic and phenotypic-led approach. 17
Figure S2. Differences in population genetic variability of actin loci. 18
Figure S3. Compatible number of cancer-associated somatic variants in CYA genes 19
Figure S4. GestaltMatcher analysis of the NMA spectrum.. 20
Figure S5. Size-matched inter-group separation after downsampling. 22
Figure S6. Immunoblot of Sf9 insect cell lysate revealed only small amounts of mutated actin-thymosin β4 fusion constructs in the cell lysate 23
Figure S7. Expression of CYA isoforms in patient-derived and control fibroblasts. 24
Figure S8. Western blots of ßCYA in patient-derived and control fibroblasts. 25
Figure S9. Western blots of γCYA in patient-derived and control fibroblasts.................... 26
Figure S10. Western blots of panactin in patient-derived and control fibroblasts. 27
Figure S11. Pyrene–based bulk-polymerization and depolymerization experiments of CYA isoforms (5% pyrene–labeled)... 28
Figure S12. Expression profiles of the patient-derived and control fibroblasts. 29
Figure S13. Principle component analysis of the average expression profile per patient. . 30
Figure S14. MRI images with cortical malformations typical for BWCFF 31
Figure S15. Spatial enrichment of variants by phenotype across the actin structure. 32
Figure S16. Functional classification of non-muscle actinopathies. 34
Supplemental Tables **35**
Table S4. List of antibodies 35
Table S5. GestaltMatcher analysis - positive predictive values for all pairwise contrasts presented in Figure S4. 36
NMA clinical consortium **37**
References.. **45**

Supplemental Notes

Note S1. Syndrome caused by *ACTB* pLoF variants

This note includes details about the syndrome cause by heterozygous germline predicted loss of function (pLOF) variants (nonsense and frameshift), and missense variants (MVs) resulting in instability of cytoplasmic β-actin (β-CYA).

0% Absent, <10% Rare, 10-25% Sometimes, 25-75% Frequent, >75% Very frequent 100% Always

Tabular summary of clinical features of individuals with *ACTB* pLOF variants (N=31*)

**Intellectual development and behaviour problems** (Frequent)	21 had intellectual difficulties (ID), mostly borderline/mild and moderate (in two). Where information was available, individuals without ID had either normal or low normal IQ (75-80). Generally, individuals had open and pleasant personalities. Behaviour anomalies reported in 14/31 (45%) individuals (with or without ID), and included attention deficit hyperactivity disorder (ADHD), temper tantrums, autism and difficulty in socialising.
**Craniofacial Anomalies** (Very frequent)	Recognisable facial features with long face, straight eyebrows, deep set eyes, epicanthus, narrow or flat nasal bridge with broad nasal tip, and large mouth. Microcephaly was present in 13.
**Eye coloboma** (Rare)	Iris coloboma and cataract in one individual (116-B).
**MRI anomalies** (Frequent)	Reported in 5/14 (36%) and included periventricular nodular heterotopia (PNVH) in three, hypoplastic corpus callosum (CC) and hypoplastic cerebellar vermis in one, and unspecific areas of high T2 signal in one. Pachygyria was not reported in anyone. MRI was not performed in 17/31.
**Growth problems** (Frequent)	Short stature was documented in 11 (up to -4 SD).
**Epilepsy** (Absent) **or Seizures** (Rare)	No individual was reported to have epilepsy. Seizures were reported in two and included as single episodes of seizures during the early childhood with spontaneous remission later (in individual 168-B with additional variant in *MID2* and individual 107-B).
**Dystonia** (Rare or absent)	Reported in one previously published individual (63-B, patient XXIV[1]) who was lost for follow-up. No neurological features noted in other individuals (included five older than 35y of age).
**Hearing loss** (Rare)	Reported in three and included bilateral sensorineural (173-B, XXVI[1]), bilateral mixed (153-B, XXII[1]), or conductive that resolved after the first few years of life (111-B).
**Skeletal anomalies** (Frequent)	Reported in nine and included pectus deformities, scapula winging, leg deformities, craniosynostosis, congenital parietal foramina, and scoliosis.
**Heart Defects** (Frequent)	Congenital heart defects reported in eight individuals and included atrial septal defect (ASD), ventricular septal defect (VSD), pulmonary stenosis, and patent ductus arteriosus. One individual reported with cardiomyopathy (remission at 10y). One individual reported with left ventricular dilatation at 2y without functional consequences (last assessment at 12y).

**Respiratory problems** (Sometimes)	Reported in five and included severe and prolonged respiratory infections and pneumonias in three and asthma in the remaining two
**Gastro-intestinal problems** (Frequent)	Reported in 12 and included feeding difficulties, failure to thrive in early childhood, constipation, gastro-oesophageal reflux, esophageal atresia with trachea-oesophageal fistula (in one) and gallstones (in one).
**Genito-urinary anomalies** (Sometimes)	Reported in five and included horseshoe kidney in three, hypospadias and renal cortical cysts.
**Skin and integument** (Rare)	Atopic reactions (in three), sparse scalp hair, generalised hirsutism, facial haemangioma, extra skin folds on abdomen and back.
**Repeated infections** (Frequent)	Reported in 8 and included recurrent respiratory infections including pneumonias, and multiple acute otitis media, chronic ear infections. Tests of immune function were performed in one and no abnormality was detected.
**Haematological anomalies** (Sometimes)	Thrombocytopenia was documented in 6 individuals from the original report of the *ACTB*-associated syndromic thrombocytopenia[2], one individual with *ACTB* gene deletion (111-B) and one patient with an *ACTB* MV (61-B). Blood counts were not available for 4/31.
**Healthy carriers** (Rare)	All carries demonstrated typical features that however might have been limited to mild craniofacial dysmorphism and learning difficulties mentioned only on enquiry after the molecular diagnosis

*Some features could not be assessed in all individuals.

Diagnostic and follow-up recommendations

- Individuals with larger deletions can have more severe presentation, perhaps due to loss of other genes.
- 6 out of 31 patients inherited the *ACTB* pLOF variant from a parent who was similarly affected, or mildly affected, or apparently unaffected. pLOF *ACTB* variants should, therefore, be considered as pathogenic variants even if inherited from apparently unaffected parent.
- Clinical follow-up should consider appropriate nutritional status in infancy and early childhood; screening for heart and renal defects; regular hearing test in early childhood in individuals with recurrent otitis; singular blood count with occasional follow-up if necessary (transitory thrombocytopenia with spontaneous remission during first decade was reported); GI function monitoring; developmental and behaviour assessment with appropriate intervention (individuals with severe behaviour anomalies benefit from symptomatic medication).

Note S2. Baraitser-Winter-Cerebrofrontofacial Syndrome

The first report[3] of the syndromic condition later named as Baraitser-Winter syndrome described three children with ID and a unique combination of clinical features including iris coloboma, bilateral ptosis, telecanthus, hypertelorism and short stature, the gestalt resembling Noonan syndrome[4]. The major clinical features were further extended with trigonocephaly and/or prominent metopica suture and lissencephaly mostly in form of frontal predominant pachygyria with or without posterior subcortical band heterotopia[5,6]. Delineation of the genetic cause[7] demonstrated that two other syndromes originally described as separate conditions (Fryns-Aftimos and cerebrofrontofacial syndromes) were part of the same spectrum with a unifying name of the Baraitser-Winter-Cerebrofrontofacial syndrome (BWCFFS) [8,9].

Tabular summary of clinical features in individuals with BWCFFS (N=113)

**Intellectual development and behaviour** (Nearly always)	101/104 individuals whose developmental level could be assessed, presented with developmental delay (DD) (N=25, age 9mo-3y) or ID, that was mild (in 24), moderate (in 33), severe (in 21) and profound in 6 individuals. For 17 individuals ID grade was not specified. Remaining 5 individuals were younger than 9 months; 4 fetal cases were also excluded from this evaluation. One of three individuals without ID had IQ>130. 14 individuals with ID showed behaviour anomalies that included ADHD, hyperactivity, temper tantrums with aggression and autism; however, majority of the individuals had open and pleasant personalities.
**Craniofacial Anomalies** (Nearly always)	Craniofacial anomalies were very consistent resulting in distinct and recognizable facial gestalt. Typical features included prominent metopic ridge, hypertelorism, high-arched eyebrows, ptosis, long palpebral fissures with everted lower lid, broad nasal tip, long smooth philtrum, large mouth with thin upper lip and everted lower lip, grooved chin, large vertically oriented ears, low posterior hair line. Face becomes coarser in the 2nd decade, so gestalt is easier to recognize. Microcephaly was reported in 52% (59/113) individuals with HC in range of -2 to -5.6 SD. Craniosynostosis requiring surgical correction was documented in 5 individuals.
**Eye coloboma** (Frequent)	Iris and/or chorioretinal colobomas were reported in 28. However, vision problems and other eye anomalies were seen in 43 individuals represented by reduced vision, refractive errors and in some individuals microphthalmia/microcornea, nystagmus, cataract and bilateral congenital fibrosis of the rectus medial and inferior extraocular muscles seen in one patient.
**MRI anomalies** (Very frequent)	MRI anomalies were documented in 81/97 (84%) individuals and were represented by lissencephaly/pachygyria in 52. Other cortical malformations included dysgyria/polymicrogyria in five, and periventricular nodular heterotopia (PNVH) in two individuals. Other anomalies included agenesis or hypoplastic CC, leukomalacia, Chiari I anomaly, hypoplastic cerebellum, and ventriculomegaly. MRI was not performed in 16 individuals.
**Growth** (Frequent)	Short stature was documented in 39 (height -6,4 to 3,4 SD) One individual had significantly delayed bone age and received growth hormone therapy with some catch up growth (23-B). Significant failure to thrive in early childhood was reported in at least 6 individuals

**Epilepsy** (Frequent)	Epilepsy was present in 41, with age of onset ranging from neonatal period to 24y. Cortical malformations were reported in 28 individuals with epilepsy, five were reported not to have structural brain anomaly, and in four individuals brain imaging was not performed.
**Dystonia** (Absent)	Not reported.
**Hearing loss** (Frequent)	Reported in 35 individuals and included bilateral sensorineural (in 19), conductive (in two) or unspecified in the remaining individuals.
**Skeletal anomalies** (Frequent)	Reported in 55 and included spine anomalies in 22, pectus anomalies, scapula winging, hip dysplasia, Polydactyly (in seven) and feet deformities.
**Heart Defects** (Frequent)	Reported in 39 and included ASD, VSD, pulmonary stenosis, patent ductus arteriosus, aortic coarctation, and valve anomalies.
**Respiratory problems** (Sometimes)	Reported in 14 and included severe and prolong respiratory infections and pneumonias in six individuals, sleep apnoea and asthma in two individuals as well as laryngomalacia and narrow nasal passage in one patient, respectively.
**GI problems** (Frequent)	Reported documented in 27 and included constipation, feeding difficulties (four required gastrostomy feeding), structural anomalies (in four individuals including duodenal atresia, jejunal atresia, intestinal malrotation and partial bowel obstruction). One individual had progressive liver cirrhosis, and another presented with chronic diarrhoea. Interestingly, GI complains were not documented in the first BWCFF cohort[8] and were often reported only on enquiry and no detailed information was available for 44 of 109 individuals (4 fetal cases excluded).
**Genito-urinary anomalies** (Sometimes)	Structural renal anomalies were reported in 23 individuals and included 10 with duplicated kidneys and/or collecting system, 7 with severe hydronephrosis, and two individuals each with ectopic kidneys, renal fusion or hypoplastic kidneys. Abnormal external genitalia were described predominantly in males including cryptorchidism, inguinal hernia and small penis. Hypoplastic external genitalia were also reported in one female individual.
**Skin and integument** (Sometimes)	Reported in 19 individuals, included dysplastic skin derivatives in 7 individuals (sparse hair, hypoplastic nails, hypodontia, small teeth and delayed tooth eruption), cutis hyperelastica, vascular anomalies (cutis marmorata, teleangiaectasia, hemangiomas) and skin hyperpigmentation (café au lait marks and Mongolian sacral spot), dermatitis, pterygia and interdigital webbing reported in one or two individuals each.
**Repeated infections** (Sometimes)	Repeated and/or excessive infections were documented in 13 individuals mostly as recurrent respiratory infections including pneumonias, multiple acute otitis media as well as chronic ear infections, urinary tract infections and necrotising enterocolitis.
**Haematological anomalies** (Absent)	Not reported (including thrombocytopenia).
**Other**	We could confirm previous observation about BWCFFs typical body posture than becomes apparent in the second or third life decade[8]. This includes anteverted shoulders with generally narrow shoulder gridle, scoliosis and semiflexed knees. Children are often

	present with excessive nuchal skinfolds or pterygium colli, low posterior hairline, pectus excavatum and mild diastasis recti resulting in prominent navel or umbilical hernia.
**Healthy carrier** (Almost absent)	All carries demonstrated typical features and vast majority of the individuals had *de novo* variants. Only one patient inherited the pathogenic variant from affected mother[9], who presented mild but typical gestalt and had low normal intelligence.

*Some features could not be assessed in all individuals.

BWCFF specific brain anomalies

BWCFF is associated with a specific MCD pattern: frontal-predominant pachygyria, frontal pachygyria accompanied with a thin occipital band heterotopia and PVNH (Figure S12). As bilateral PVNH were also observed in individuals with *ACTB* pLOF disorder, we did not consider the later MCD to be BWCFF specific. Enlarged (prominent) perivascular spaces in the centrum semiovale (but not in basal ganglia) were noted in several BWCFF individuals with and without cortical malformations (N=18). As this is a quite common non-specific finding, we suspect that enlargement of perivascular spaces might have been overlooked or not mentioned in the final radiological report in individuals where no MRI images were available for the evaluation.

Prenatal manifestation of BWCFFs

43 of 76 BWCFFs individuals with available pregnancy data had abnormal prenatal history. For the remaining 37 individuals, early clinical information was not available. 3 pregnancies were terminated between 26th and 35th gestational weeks. The most common manifestation was increased nuchal translucency (N=22) either transient or persisting and reaching the form of cystic hygroma (N=7). 14 individuals presented with hydrops fetalis. Other recurrent features were microcephaly, agenesis of the CC, ventriculomegaly or hydrocephalus, polyhydramnios, cleft lip/palate, cortical anomalies, structural renal and heart anomalies. Reduced foetal movements, oligohydramnios and duodenal atresia were reported in a single patient respectively. Although common, prenatal manifestation is not specific and does not allow for clinical suspicion of BWCFF in absence of family history. Prenatal diagnosis is only possible through exome/genome-wide genetic testing. We recommend a very careful consideration of the clinical diagnosis in every patient with a novel MV in *ACTB* and *ACTG1* as well as MVs that were previously observed in less than three individuals and/or MVs with insufficient clinical information.

Adult complications and reduced life expectancy in BWCFFs

Our BWCFFs cohort includes 19 individuals at the age 18-45 years. The oldest known patient was 62y old at the time of the last follow-up (personal experience of Allan Bayat, limited clinical data were available). 16/19 individuals had epilepsy with AO from 2-24y and all 19 individuals showed ID ranging from mild (2 individuals), moderate (N=7) to severe (N=10). 10/19 individuals demonstrated progressive spinal deformity, limited extension of large joints and slow decline in overall motor activity. Two previously reported individuals died at the age of 26y (P1[10]) and 30y (B34[8]) from complications of the acute ileus and progressive feeding difficulties resulting in recurrent respiratory pneumonias. Both individuals carried the same MV p.Thr120Ile in *ACTB*. The whole BWCFFs cohort encompasses two other deceased individuals with presumable cause of death being adverse reaction to codeine administration at the age of 20y[10] and progressive sepsis in a 8m boy with untreated decompensated heart defect[11]. The

current adult cohort might not be representative for milder affected individuals that were currently diagnosed via genotype-first approach.

Diagnostic and follow-up recommendations

- BWCFF diagnostic criteria delineated in this work - (1) specific facial dysmorphism, and/or (2) frontal predominant pachygyria in a patient with (3) (likely) pathogenic MV in *ACTB* or *ACTG1*.
- Clinical follow-up includes initial organ screening (brain MRI, EEG, heart ultrasound, abdominal and renal ultrasound, assess of the nutritional status, ophthalmologic evaluation including fundoscopy, audiologic evaluation, developmental assessment and genetic counseling) and annual surveillance (surveillance might be more frequent depending on the individual situation)[12]

Note S3. *ACTB*:p.Arg183Trp-related dystonia-deafness syndrome

Our cohort included 9 individuals with well documented progressive generalized dystonia; all of them carried an identical pathogenic variant in *ACTB*:p.Arg183Trp. All individuals had a history of the profound prelingual sensorineural hearing loss with significant improvement with the cochlear implants in individuals who received them. Four remaining individuals were ascertained following detection of *ACTB*:p.Arg183Trp and presence of the congenital deafness without BWCFF specific features. Dystonia was a fully penetrant feature in all adults. However, this might represent an ascertainment bias as all adult individuals underwent genetic testing because of dystonia, whereas younger individuals received exome sequencing because of congenital hearing loss. As the cohort remains small, the exact penetrance of dystonia cannot be estimated.

First individuals reported with *ACTB*-DDs were monozygotic twins[13,14] that were subsequently discussed as a part of BWCFF spectrum[8].

It remains currently unknown whether other MV within the same *ACTB* codon would also result in *ACTB*-DDs. In ClinVar we identified one individual with a de novo MV NM_001101.5(*ACTB*):c.547C>G (p.Arg183Gly). This variant was evaluated by a single submitter as a likely pathogenic for *ACTB*-related disorder. No clinical data could be provided after our active enquiry.

Tabular summary of clinical features in individuals with deafness-dystonia syndrome (N=13)

**Intellectual development and behaviour**	Borderline/mild ID was present in 5 out of 13 individuals, however two individuals with normal intelligence had delayed motor and/or speech development. Three individuals demonstrated abnormal behaviour with anxiety and insecurity in two individuals and psychotic episodes with paranoid delusions in another one.
**Craniofacial anomalies**	No specific gestalt has been documented, however 6 individuals had hypertelorism with arched eyebrows and/or mild ptosis. None of the individuals had BWCFF facial gestalt.
**Eye coloboma**	No coloboma was reported, one patient had cataract diagnosed at 3y[13]
**MRI anomalies**	MRI anomalies were present in two individuals and included arterial ischemic stroke at 5y in one subject (26-B) and bilateral symmetrical FLAIR T2 hyperintensity in the basal ganglia[15] in the other patient with manifest dystonia.
**Epilepsy**	Focal seizures with AO at 5y were reported in one patient[16].
**Dystonia**	Dystonia manifested in 9 of 13 individuals, AO varied from 11 till 24 years and the disease progression from focal into severe generalised dystonia, 5 individuals received deep brain stimulation with positive effect, 3 individuals died. Dystonia did not respond to conventional drug treatment such as LDopa, biperiden and clonazepam[16]. Three individuals without dystonia were 7months, 2y and 12y at the last follow-up.
**Hearing loss**	Profound hearing loss was documented in all individuals, 7 individuals received cochlear implant, one as early as 11months.
**Skeletal anomalies**	Skeletal anomalies (scoliosis) were documented in 3 individuals[13,16] and most probably represented secondary complications of progressive uncontrolled dystonia.
**Heart Defects**	Structural heart defects were not documented.
**Respiratory anomalies**	Respiratory features were present in 3 individuals and included aspiration pneumonia, asthma, and impeded breathing during

	cold most probably representing secondary complication of progressive uncontrolled dystonia.
**GI anomalies**	Gastro-intestinal concerns were documented in 5 individuals and included constipations reported in three individuals as well as achalasia reported in monozygotic twins[13].
**Genito-urinary anomalies**	No GU anomalies were documented.
**Skin and integument**	One patient presented with dermatitis.
**Repeated infections**	Repeated and/or excessive infections were not documented
**Thrombocytopenia and other haematological anomalies**	Thrombocytopenia was not observed.
**Healthy carrier**	All carries presented with congenital deafness; as dystonia is an age-related manifestation, its penetrance in the youngest individuals remains unknown.

Diagnostic and follow-up recommendations

- *ACTB*-DDs is diagnosed in individuals with early onset severe hearing loss carrying MV *ACTB*:p.Arg183Trp.
- Hearing loss is severe and rapidly progressive suggesting that early cochlear implants should be considered to maintain adequate language development.
- The incidence of dystonia remains unknown but current data suggests that it may be as high as 100%.
- Regular monitoring of motor and language development with appropriate early intervention program if necessary.
- Early connection to the neurologist specialized in movement disorders is highly recommended to facilitate future treatment.
- Bilateral globus pallidus interna deep brain stimulation currently represents the only treatment option resulting in substantial clinical improvement.
- Supportive therapy such as early initiation of physiotherapy and application of the adaptive aids after onset of dystonia.

Note S4. *ACTG1*-associated isolated hearing loss (*ACTG1*-ADHL)

Non-syndromic hearing loss was the first disorder associated with the cytoplasmic actin genes[17-19]. Heterozygous variants in *ACTG1* were reported segregating in six families with multiple affected individuals including a large Norwegian family with more than 40 affected family members presenting with post lingual progressive sensorineural hearing loss, clinically defined as DFNA20/26[19,20]. The typical characteristics include a bilateral slowly progressive sensorineural hearing loss with the age of onset between the first and the third decades of life. The hearing loss begins at the highest frequencies and steadily progresses into profound deafness across all frequencies. The majority of the affected individuals would demonstrate the sloping configuration audiogram at the early age while hearing threshold remains intact at the lower frequencies. The hearing loss is progressing into deafness by the 6th decade[21]. Tinnitus, vertigo, and other vestibular symptoms were occasionally reported in individuals with the *ACTG1*-associated hearing loss. The recent review summarized 36 *ACTG1* variants reported in individuals with hearing loss. However, several individuals presented with additional symptoms including other malformations and neurodevelopmental disorder[21] indicating that these individuals should be classified as unspecified non-muscle actinopathies. Considering the highly variable symptomatic even within the same family[22], we recommend careful consideration of the clinical assignment in individuals with the novel *ACTG1* variants especially when the molecular diagnosis was done early in life. Several missense variants such as T89I[17], K118M/N[17,23,24], K213R[25], E241K[23,24], T278I[18], and V370A[19] were recurrently observed in large well characterized families with non-syndromic hearing loss and some of these variants were also studied *in-vitro* and *in vivo*[23,26,27]. These variants can be reliably associated with the non-syndromic hearing loss.

The penetrance was reported as complete; however, the age of onset, progression and severity differ greatly even within the same family[19,21].

Tabular summary of clinical features in individuals with non-syndromic hearing loss (N=60)

**Intellectual development and behaviour**	Normal development.
**Craniofacial anomalies**	No specific gestalt has been documented.
**Hearing loss**	Bilateral progressive sensorineural hearing loss with the typical begin at the higher frequencies and characteristic audiogram with the sloping configuration that may maintain even at the advanced stage. The progression rate varies from 1 dB/year to 6 dB/year[24].
**Vestibular symptoms**	Vestibular dysfunction, manifested as some equilibristic instability, was claimed occasionally by some of the elderly, profoundly hearing-impaired individuals but was formally assessed[19]. Tinnitus is occasionally reported.
**Healthy carrier**	Not reported; hearing loss is an age-related phenotype with the variable onset within the same family.

Note S5. Unspecified non-muscle actinopathies including *ACTG1*-associated isolated coloboma

This cohort encompassed individuals with missense variants in either *ACTB* or *ACTG1* whose clinical features did not fit any of the disorders described above. It is possible that future work might define novel distinct entities within this group, one of which could be an *ACTG1*-associated isolated coloboma[28]. unNMA is diagnosed in a patient without BWCCF typical facial gestalt and/or brain malformation with a (likely) pathogenic missense variant in *ACTB* or *ACTG1* (except *ACTB* R183W) presenting with any phenotype other than post-lingual non-syndromic hearing loss.

In line with the previous section, we want to point out the high phenotypic heterogeneity in this group observed even within the same family. Although most of the individuals presented with the neurodevelopmental disorder, the severity of the intellectual impairment is usually mild with good developmental progress under intensive speech and occupational therapy. Speech was usually more severely impaired in comparison with motor skills. Speech delay was more prominent in children with the congenital or early onset hearing loss and remained a significant health issue even after the administration of the adequate hearing aids or cochlear implants.

The available data on adult individuals in unNMA (10 individuals older than 20y) indicates the stable course with no additional neurological or other health issues being developed. However, this statement would need to be confirmed in a larger patient cohort.

Tabular summary of clinical features in individuals with unspecified NMA (N=66)

	***ACTB* N=36**	***ACTG1* N=30**
**Intellectual development and behaviour**	21 individuals, borderline/mild in 13 and moderate in 6, 7 individuals had normal mental development; 8 individuals presented prenatally or during neonatal period; 10 individuals with and without ID had behaviour anomalies, presented with ADHS, hyperactive and aggressive behaviour and temper tantrums; single individuals were reported to have sleep disorder, Tourette syndrome and auto aggression.	20 out of 30, mild in 7, moderate in 6 but also severe and profound in 3 individuals; 8 individuals had behaviour anomalies with ADHS and temper tantrums, as well as autism with obsessions described in 1 patient.
**Craniofacial anomalies**	Craniofacial anomalies were present in 27 individuals and were mild in the majority of the individuals. Microcephaly was documented in 11 individuals. Interestingly, microcephaly was a consistent feature in three individuals with MV within the codon 152.	Mild craniofacial anomalies were described in 16 individuals presented with an unspecific pattern. However, 4 individuals had ptosis accompanied by epicanthus in 2. Only two individuals presented with microcephaly.
**Eye coloboma**	Iris coloboma was reported in two individuals.	Iris coloboma was reported in three individuals.
**MRI anomalies**	MRI anomalies were documented in 15 individuals but nobody	MRI anomalies were present in 7 individuals, thereof 4

	presented with cortical malformations except one patient with single PVNH. Structural abnormalities included abnormal corpus callosum in 3, enlarged ventricles in 3 and hydrocephalus in 1, posterior fossa anomalies in 2, as well as Chiari I anomaly, multiple calcifications and abnormal white matter signal in one patient, respectively.	individuals had cortical malformations including PMG in 2, dysgyria in 1 and PVNH in 1; the remaining 3 individuals had either agenesis or hypoplastic corpus callosum.
**Epilepsy**	Epilepsy was present in 5 individuals, two of them had abnormal MRI such as Chiari I anomaly and multiple calcifications. Another patient was diagnosed with Doose syndrome.	Epilepsy manifested in 5 individuals, two of them had cortical malformations (PVNH and PMG).
**Dystonia**	Dystonia was not documented.	Dystonia was not documented.
**Hearing loss**	Hearing loss was documented in 6 individuals as bilateral sensorineural in three individuals, mixed in one patient and conductive in another two individuals.	Hearing loss was present in 21 individuals, all individuals had bilateral sensorineural hearing loss with AO from birth/first year till 3rd and 4th decades. However, adult onset was observed only in one multigenerational family with MV p.Ile85Leu. Other individuals had the onset in early childhood.
**Skeletal anomalies**	Skeletal anomalies were documented in 11 and included vertebral anomalies (N=4) as well as pectus deformity, joint hypermobility, feet deformities, brachydactyly and long and slender fingers described in individual individuals. Eight individuals had short stature (till -3,4 z).	Skeletal anomalies were present in 9 individuals, 5 had scoliosis, two had short stature (-4,7 z) and two bilateral feet deformities, respectively.
**Heart Defects**	Structural heart defects were present in 9 individuals and included ASD, VSD, aortic coarctation, and PFO. Two individuals had transposition of the great arteries and one had dextrocardia. Two individuals had mitral valve prolapse.	Heart anomalies were seen in 5 individuals as ASD/VSD, PDA, pulmonary stenosis and right descending aortic arch with aberrant left subclavian artery and diverticle of Kommerell, respectively.
**Respiratory anomalies**	Respiratory features were present in 4 individuals and included severe and prolonged respiratory infections and	One patient had asthma; another patient presented with laryngomalacia and two individuals had documented

	pneumonias in three and respiratory distress in the remaining patient.	tracheomalacia in early months.
**GI anomalies**	Gastro-intestinal concerns were documented in 12 individuals and required operative treatment in 4 individuals.<br>Incomplete data in 6 individuals and not assessed in 4 fetuses.	Gastro-intestinal concerns were documented in 4 individuals and presented as duodenal atresia in 1, intestinal pseudo-obstruction and TNT dependency in 1, and constipations in the other two individuals. In 8 individuals GI data was incomplete.
**Genito-urinary anomalies**	GU anomalies included renal anomalies in 5 (pyelectasis/hydronephrosis, cystic dysplasia, and pyelonephritis) and abnormal genitalia in other 7 individuals.	One patient presented with hydronephrosis, two with inguinal hernias and one with cryptorchidism.
**Skin and integument**	Diverse dermatological concerns were recorded in 5 individuals: skin laxity, mild angiomas, photosensitivity and cutaneous infections with impetigo.	CALFs were documented in a single patient.
**Repeated infections**	Repeated and/or excessive infections were documented in 7 individuals presented as recurrent respiratory infections including pneumonias in and multiple acute otitis media as well as chronic ear infections. However, 3 individuals demonstrated systemic disorder with recurrent abscesses and cutaneous infections (158-B, 62-B and 119-B). One of these individuals had thymus atrophy. One patient had periodic fever.	Repeated and/or excessive infections were documented in three individuals.
**Thrombocytopenia and other haematological anomalies**	Three individuals had thrombocytopenia presented as borderline or mildly diminished platelet count without manifesting bleeding disorder. All 3 individuals had MV in the last exon.	Thrombocytopenia was not documented.
**Healthy carrier**	All carries demonstrated either ID or structural/morphological anomalies.	All carries demonstrated either ID or structural/morphological anomalies.

Prenatal manifestation in unNMA

Abnormal prenatal history was documented in 15 individuals (N=9 with variants in *ACTB* and N=6 in *ACTG1*). Whereas increased nuchal translucency was the most common prenatal feature in individuals with BWCFF, it was reported in only two pregnancies in the unNMA cohort. Other features included ventriculomegaly, heart defects, cleft lip/palate, duodenal atresia, omphalocele, and fetal arrhythmia. Prenatal molecular diagnosis was made in four cases and led to the termination between 16 and 28 GWs. Three of four foetuses presented with ventriculomegaly or hydrocephalus, one had IUGR, transposition of the great arteries, renal cysts and omphalocele. Detailed neuropathological examination of the cerebral was available in two cases and reported normal cortical structure.

Diagnostic and follow-up recommendations

- Considering the high clinical heterogeneity within the unNMA patient cohort and still limited information about the natural history, developing general recommendations regarding the clinical management remains difficult.
- Referral to an early intervention program is strongly recommended for the detailed developmental and behaviour evaluation and intervention.
- Medical surveillance should be focused on individual presentation of the individuals and may include the control of the growth parameters, cardiac evaluation, hearing test, ophthalmological surveillance and other evaluations depending on individual concerns. Young individuals with uncertain clinical classification should have annual follow-up and their families should be informed that clinical diagnosis is ambiguous and so remains developmental and neurological long-term prognosis; families should be offered the maximal BWCFFs-oriented management that can become less intensive or lifted completely if BWCFFs can be prospectively excluded.

Note S6. GestaltMatcher facial analysis

Figure S4 and Table S5 summarize the quantitative evidence for cohort distinctiveness from the pairwise PPV analysis and the intra-group percentile results against the random baseline. The PPV values (probability that two cohorts are truly different within the decision interval, neutral prior) show strong separation between BWCFF and *ACTB* LoF (PPV ≈ 93%), indicating these cohorts are highly distinct. Comparisons against unNMA also support inter-group distinctiveness: *ACTB* LoF vs unNMA (PPV ≈ 76.5%) and BWCFF vs unNMA (PPV ≈ 63.9%) both favor difference. Within the unNMA framework, ACTB_unNMA vs ACTG1_unNMA yields a moderate signal (PPV ≈ 71.7%), suggesting gene-specific separation inside unNMA. By contrast, BWCFF vs BWCFF_unNMA (PPV ≈ 18.8%) and ACTB_BWCFF vs ACTG1_BWCFF (PPV ≈ 8.7%) show limited evidence of distinctiveness, consistent with substantial phenotypic overlap.

To make sample-size dependence explicit, Figure S5 presents a size-matched downsampling analysis. For each pair, both cohorts are repeatedly downsampled to the same size k (from 1 up to the smaller cohort), and the inter-group mean pairwise distance is summarized at each k relative to the threshold c. As expected, variability widens and apparent separation can attenuate at very small k, whereas robust pairings (e.g., BWCFF vs ACTB LoF) remain consistently above c across a broad range of k. This sensitivity analysis complements the PPV/percentile results and clarifies how limited n in recurrent-variant cohorts influences confidence in inter-group differences.

The intra-group percentile analysis (Figure 3D) against the resampled random baseline (random KDE figure) independently supports these conclusions. BWCFF and *ACTB* LoF have unusually low mean within-group distances—approximately the 1.6th and 4.4th percentiles of the random control distribution, respectively—indicating pronounced intra-group cohesion far beyond chance. By comparison, unNMA and its gene-specific subsets (ACTB_unNMA and ACTG1_unNMA) fall around the 16th–22nd percentiles, which does not indicate a recognizable, tight gestalt as a group. Together, the PPV results (between-group) and percentile findings (within-group) provide convergent, quantitative evidence that BWCFF and ACTB LoF are both internally cohesive and externally distinct from other cohorts, whereas unNMA lacks strong intra-group similarity yet can still be distinct from other cohorts in inter-group comparisons.

Note S7. Transcriptome sequencing

Missense variants in CYA genes do not have major impact on overall gene expression.

In line with the overlapping expression profiles, we observed only few differentially expressed genes between individuals derived and control cell cultures. Only one gene (*OLFM1*) was differentially expressed at an FDR-adjusted p-value cutoff of 0.01 in comparison with BWCFF patient cell cultures to control 2 cell cultures, whereas all other disease-specific cell cultures did not show any differentially expressed genes in comparison to control 2 cell cultures (Supplementary Table 3 Differential Gene Expression Analyses). Of note, seven fibroblast cultures of control 2 group were established under identical conditions like most of the patient derived cultures whereas control 1 consisted of three cultures acquired from Coriell. Some more genes were differentially expressed in comparison to control 1 cell cultures, but only for BWCFF vs. control 1 (90 genes) and *ACTB*-BWCFF vs. control 1 (43 genes).
Analysing the expression of the genes encoding for actin isoforms and actin-binding proteins(ABP) (as in Latham et al.[2]) we observed one cluster that mainly contained BWCFF samples (Figure S8) together with another larger cluster with three subclusters

including three control 2 samples in a subcluster, control 1 samples form a subcluster control samples, Dystonia Deafness, non-BWCFF and BWCFF samples that were more wide-spread across the subclusters. Therefore, analysed actin variants have only minor systemic impact even on the expression of ABP-encoding genes.

Supplemental Figures

Figure S1. Classification of the NMA patient cohort applying genomic and phenotypic-led approach.

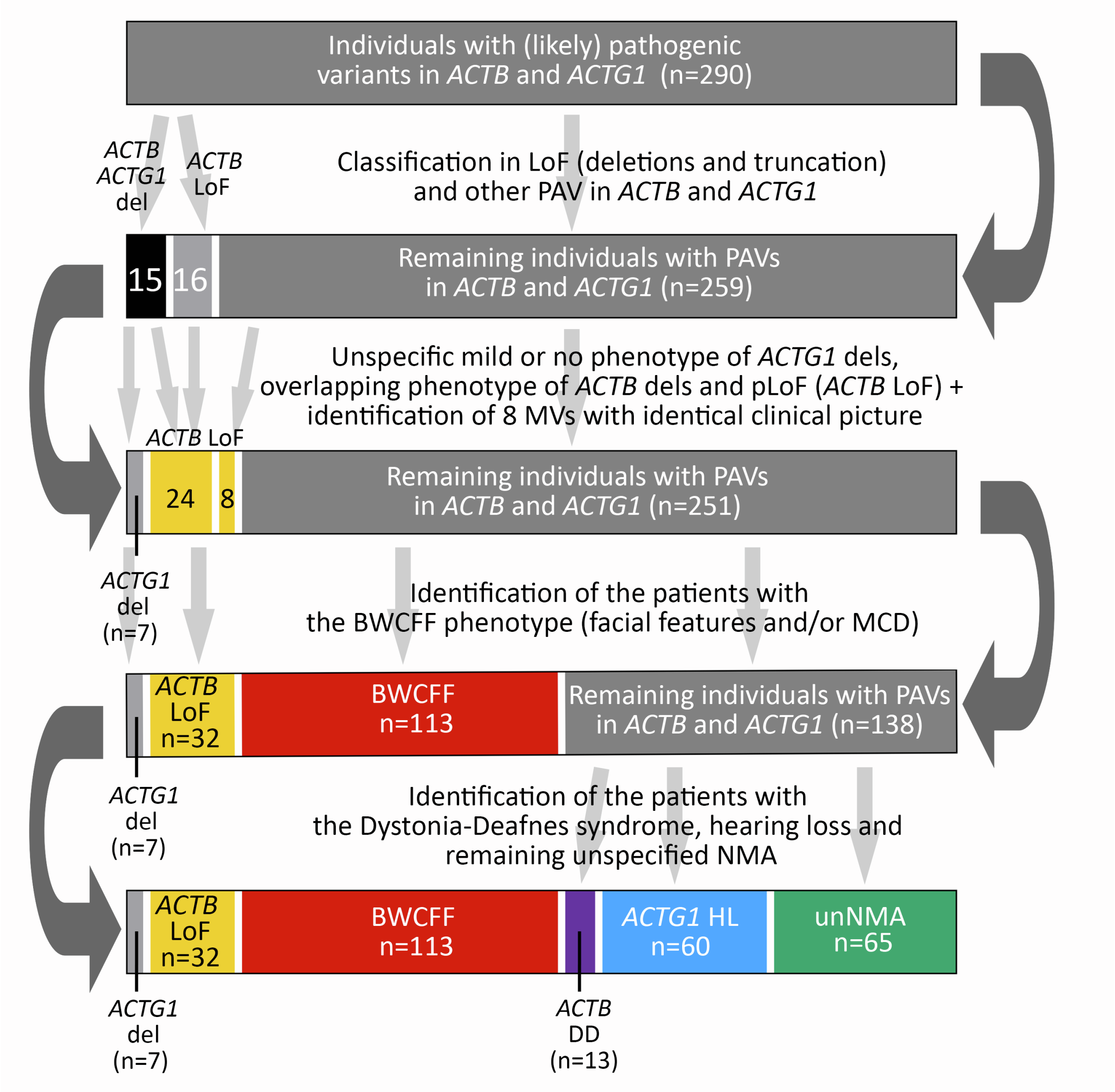

Figure S2. Differences in population genetic variability of actin loci.

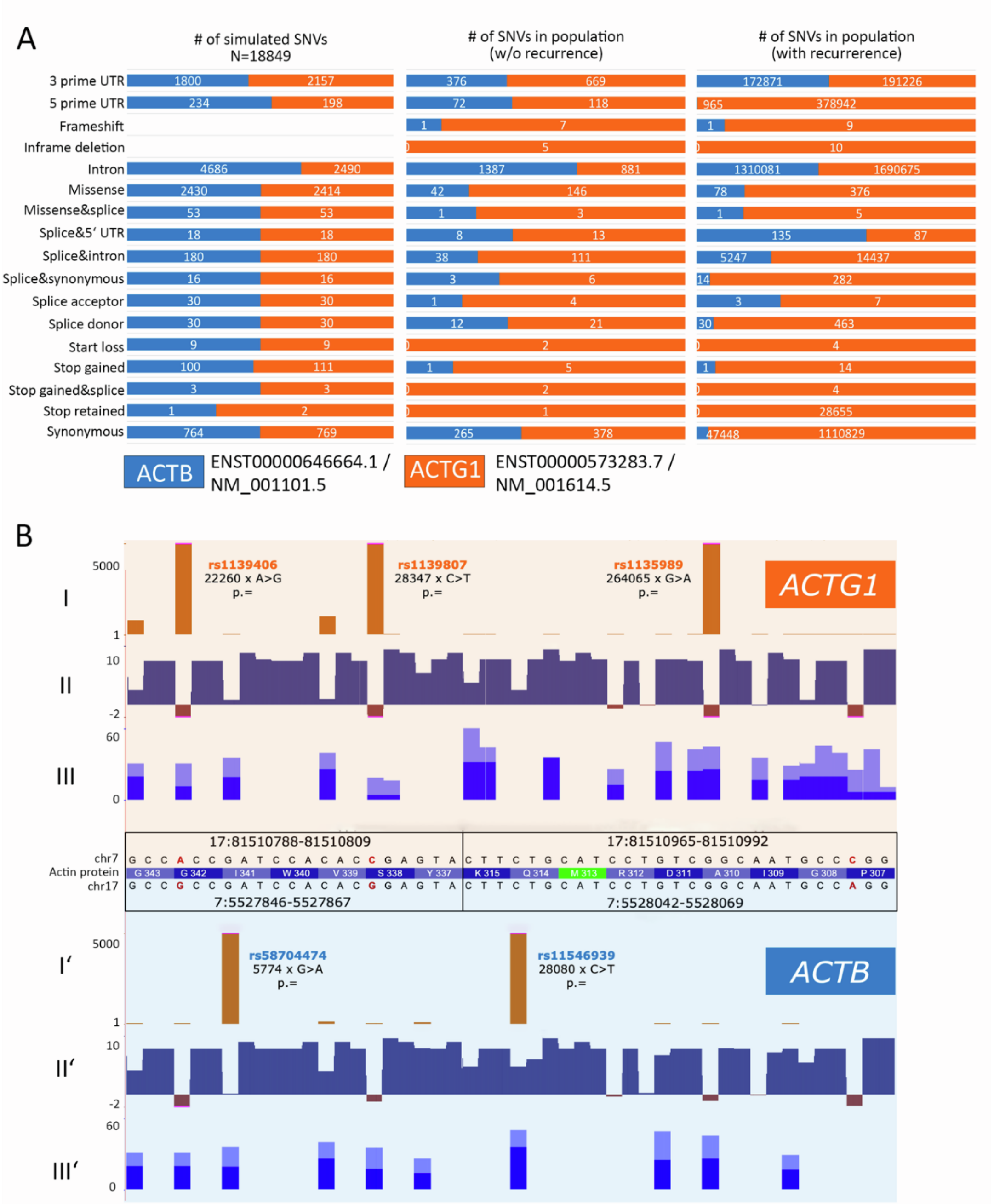

(A) Stacked column charts demonstrating functional consequences of all possible singe nucleotide substitutions simulated within the genomic regions of *ACTB* (NM_001101.5; hg38 7:5527148-5530601) and *ACTG1* (NM_001614.5; hg38 17:81509971-81512799) annotated with the Ensembl Variant Effect predictor (VEP) compared to the small nucleotide variants observed in public databases with (with recurrences) and without (w/o recurrence) population frequencies of the variants; variant types are listed in alphabetical order **(B)** integrated ucsc genome browser view demonstrating two paralogous regions within coding regions of *ACTG1* and *ACTB* (sequence differences are highlighted in red), I and I' relative usage of reference (dark blue) and alternative codon (light blue), II and II' PhyloP basewise conservation score (100 vertebrates), III and III' number of recurrent variants in public databases, pink top on bars indicates the data exceeding the display limit of 5,000.

Figure S3. Compatible number of cancer-associated somatic variants in CYA genes

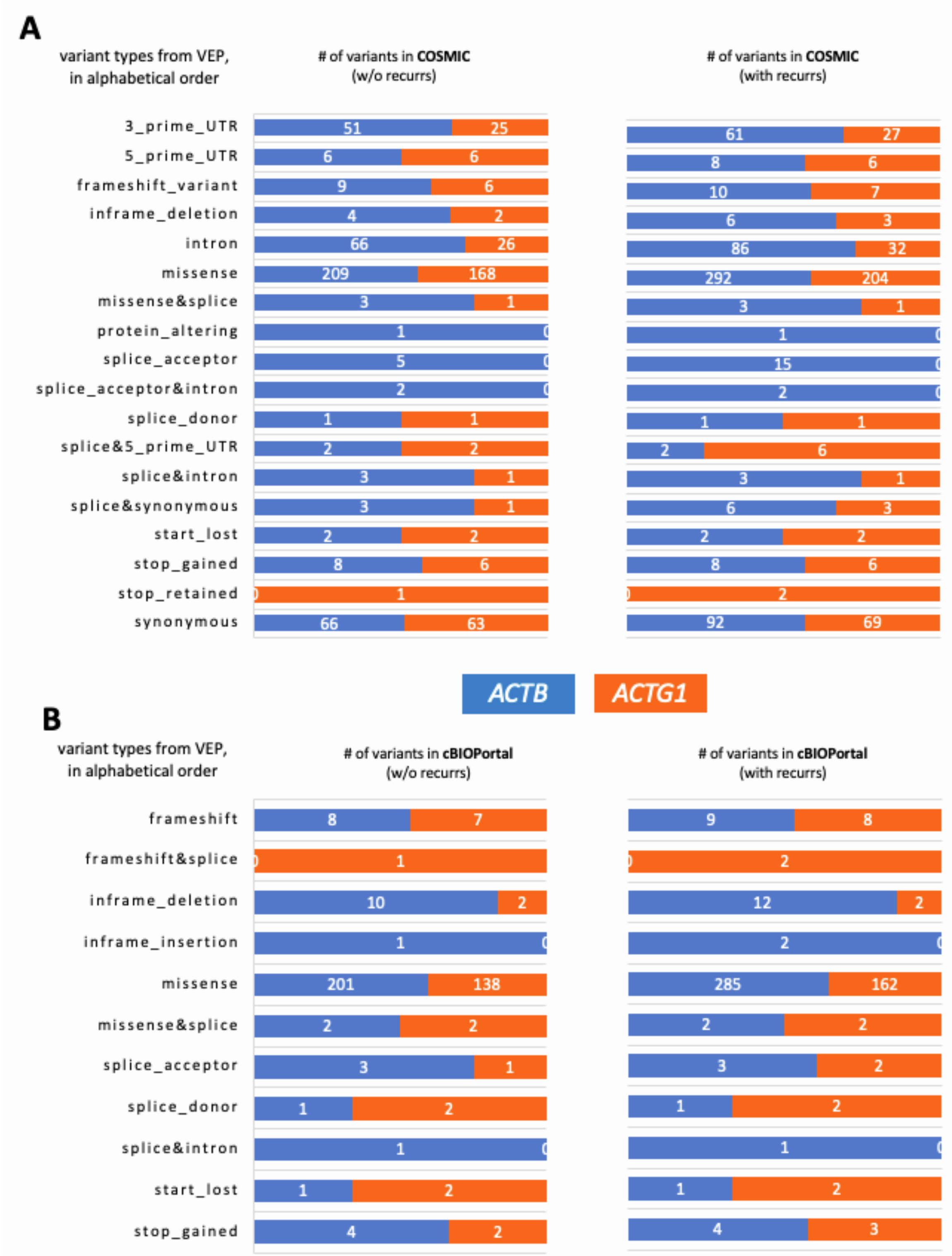

(A) Stacked column charts demonstrating the spectrum and frequencies of somatic small nucleotide variations in *ACTB* and *ACTG1* observed in COSMIC and **(B)** cBIOPortal databases; note that cBIOPortal supports only non-synonymous and coding region small nucleotide variants; colour code and annotations correspond to the Figure S2. Note that the data is included to illustrate the spectrum and relative frequencies of reported somatic variants with respect to the mutagenic potential of the affected regions; no functional or clinical conclusions were drawn.

Figure S4. GestaltMatcher analysis of the NMA spectrum.

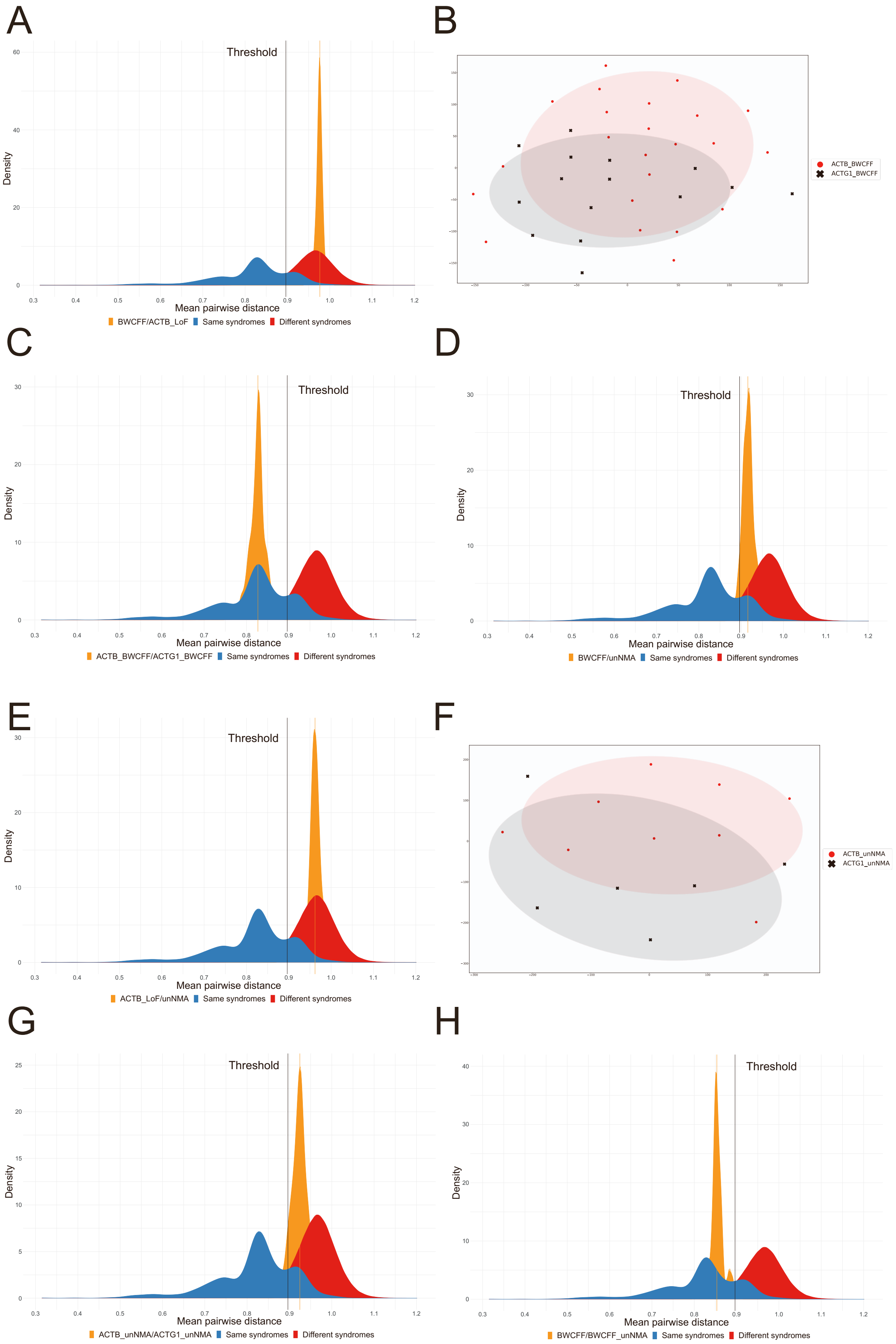

tSNE visualization of different groups in GestaltMatcher analysis and the mean pairwise distance distribution of cohorts sampled from (blue) same syndrome, (red)

different syndrome, and the orange distribution of the target comparisons. The threshold (c) is 0.896. When more than 50% of the orange region fall above the threshold, it indicates the two disorders are not similar. **(A)** The mean pairwise distance between BWCFF and *ACTB*_LoF individuals is 0.977, and 100% of the sampling is above the threshold (the region falling on the right of the threshold) PPV (positive predictive value) ≈ 93%, indicating strong distinctiveness. **(B)** The tSNE visualization between *ACTB*_BWCFF and *ACTG1*_BWCFF. **(C)** The mean pairwise distance between BWCFF_*ACTB* and BWCFF_*ACTG1* individuals is 0.827, and 0% of the sampling is above the threshold. **(D)** The mean pairwise distance between unNMA and BWCFF individuals is 0.916, and 92% of the sampling is above the threshold; PPV ≈ 63.9%, supporting inter-group difference with partial overlap; **(E)** The mean pairwise distance between unNMA and *ACTB*_LoF individuals is 0.962, and 98% of the sampling is above the threshold; PPV ≈ 76.5%, indicating separation. F) The tSNE visualization between *ACTB*_unNMA and *ACTG1*_unNMA. **(G)** The mean pairwise distance between *ACTB*_unNMA and *ACTG1*_unNMA individuals is 0.924, and 92% of the sampling is above the threshold; suggesting gene-specific separation within unNMA. **(H)** The mean pairwise distance between BWCFF and BWCFF_unNMA individuals is 0.853, and 1% of the sampling is above the threshold; PPV ≈ 18.8%, indicating considerable overlap. PPVs for all pairwise contrasts are summarized in Table S5.

Figure S5. Size-matched inter-group separation after downsampling.

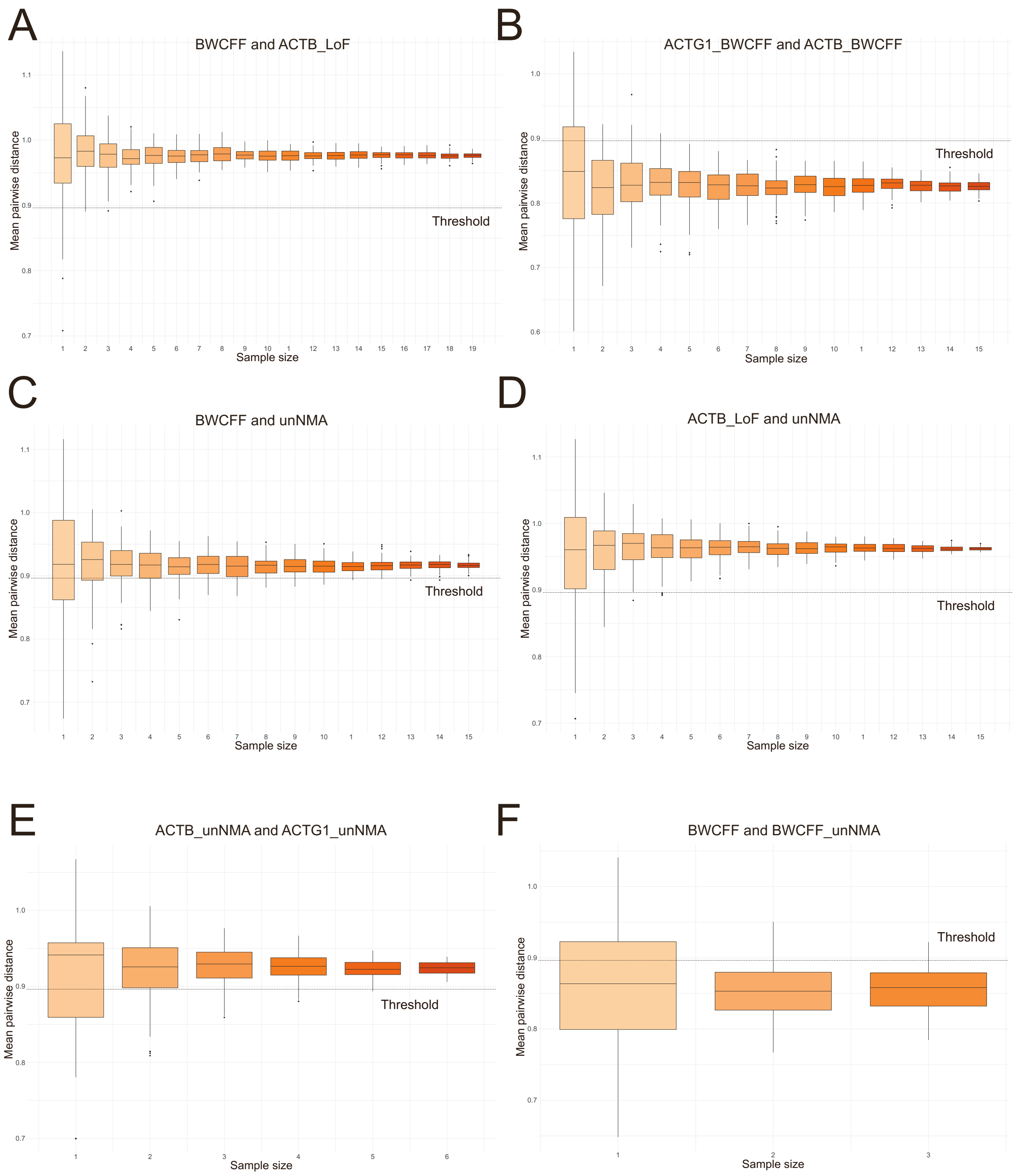

Panels A–F show pairwise cohort comparisons. For each panel, both cohorts are downsampled to the same size k (x-axis) and repeatedly resampled; the boxplots summarize the distribution of the mean pairwise distance between cohorts at that k. The dashed line marks the historical threshold $c$for "different." **A)** BWCFF vs ACTB LoF: distances remain stably above c from small $k$upward. **B)** ACTG1_BWCFF vs ACTB_BWCFF: distances are lower and closer to c, reflecting greater overlap. **C)** BWCFF vs unNMA and **D)** ACTB LoF vs unNMA: distances trend above c as k increases. **E)** ACTB_unNMA vs ACTG1_unNMA: moderate separation with wider dispersion at small k. **F)** BWCFF vs BWCFF_unNMA: small n limits precision; distances hover near c. Overall, smaller k yields wider variability due to limited sampling, while consistently separated pairs remain above c across k, illustrating how recurrent-variant cohorts with small $n$influence confidence in inter-group differences.

Figure S6. Immunoblot of Sf9 insect cell lysate revealed only small amounts of mutated actin-thymosin β4 fusion constructs in the cell lysate

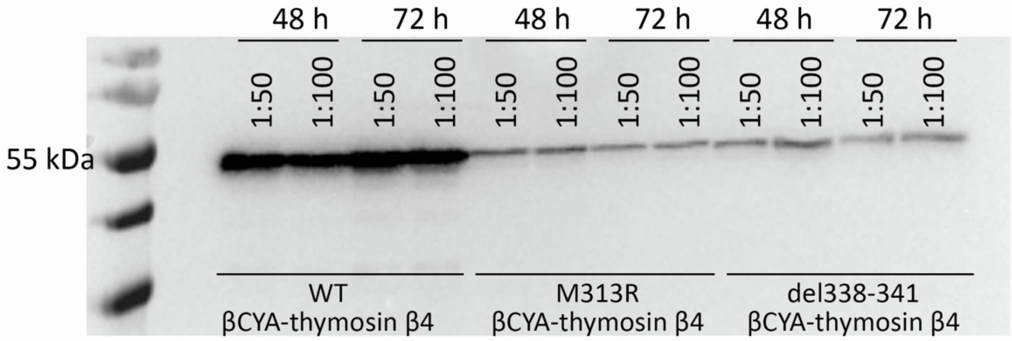

Immunoblot of Sf9 insect cell lysate revealing only small amounts of mutated actin-thymosin β4 fusion constructs in the cell lysate. This amount was not sufficient to purify mutant contracts as they could not be eluted of the NiNTA column. Cells were transfected with different titres of baculovirus encoding for the respective actin-thymosin β4 construct (1:50, 1:100). Samples were taken 48 hours and 72 hours after transfection. Blot was developed using the anti-Penta-His antibody (Qiagen, Hilden, Germany) and the goat anti-mouse IgG-HRP secondary antibody (Thermo, Waltham, USA)

Figure S7. Expression of CYA isoforms in patient-derived and control fibroblasts.

Analysis of bCYA, gCYA, and panactin protein abundance in patient-derived fibroblasts by western blot; data is presented using the box-and-whiskers plot where box contains the 25th to 75th percentiles of the data set and central line indicate the median signal intensity in immunoblots normalized to the total protein.

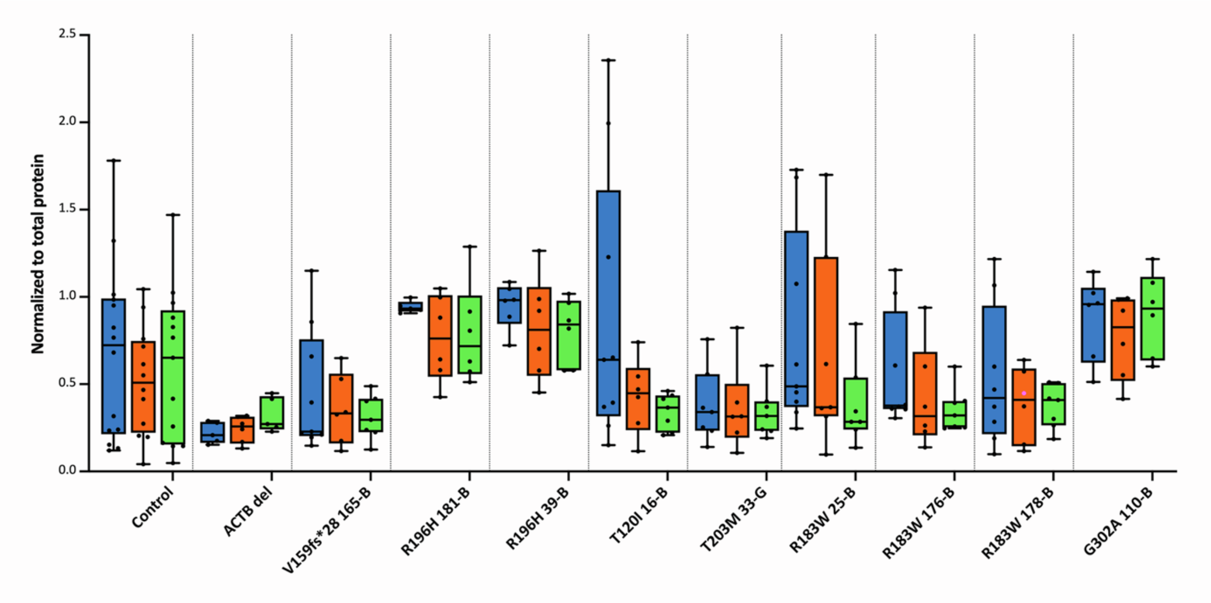

Figure S8. Western blots of ßCYA in patient-derived and control fibroblasts.

(A), (C) Fluorescent total protein membrane staining (Revert™ 700 Total protein stain), gray scale image; samples are labeled corresponding to sample identifiers in Figure S6, R183W corresponds to 25-B and R183W' to 176-B, R196H corresponds to 181-B, R196H' to 39-B (**B), (D)** Fluorescent ßCYA protein detection using IRDye800CW.

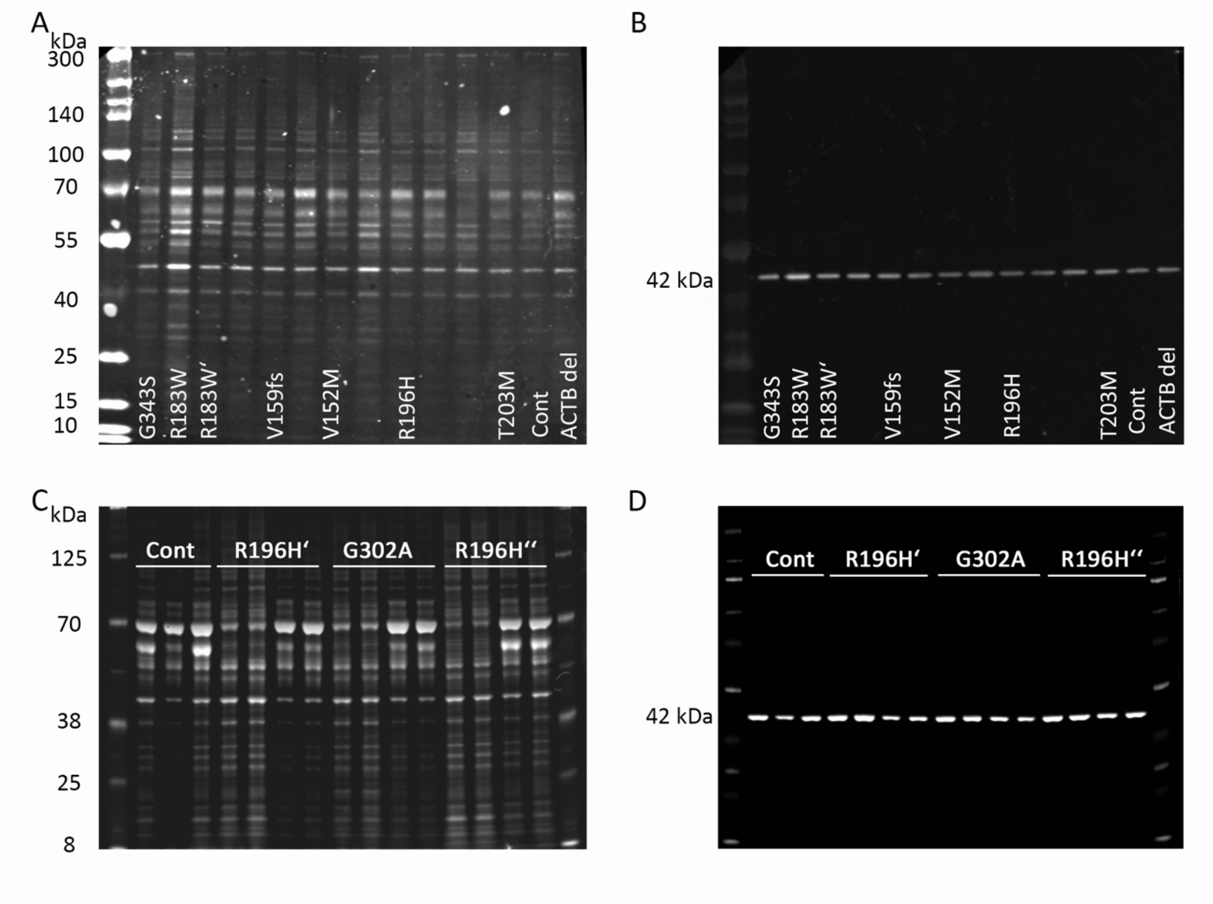

Figure S9. Western blots of γCYA in patient-derived and control fibroblasts.

(A), (C) Fluorescent total protein membrane staining (Revert^{TM} 700 Total protein stain), gray scale image; samples are labeled corresponding to sample identifiers in Figure S6, R183W corresponds to 25-B and R183W' to 176-B, R196H corresponds to 181-B, R196H' to 39-B **(B), (D)** Fluorescent ßCYA protein detection using IRDye800CW.

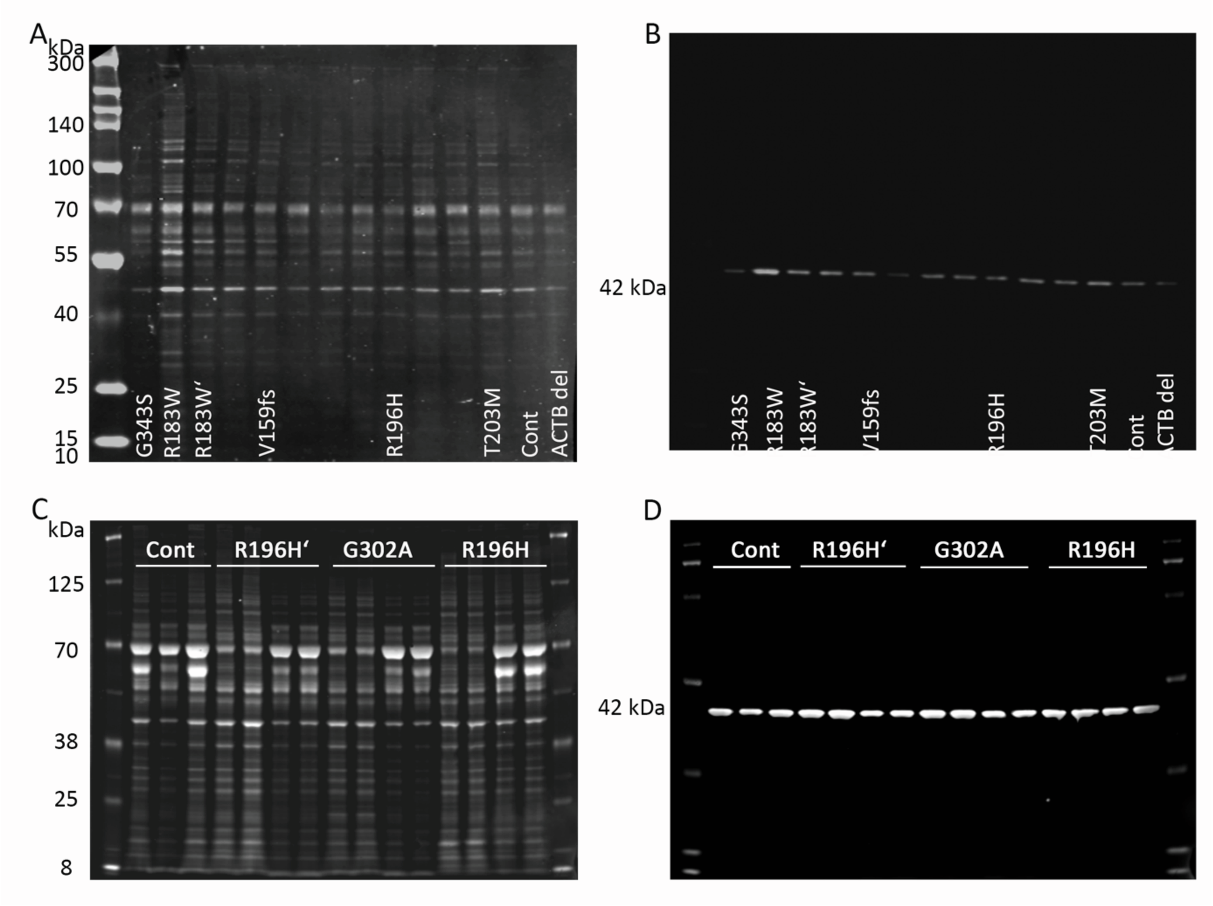

Figure S10. Western blots of panactin in patient-derived and control fibroblasts.

((A), (C) Fluorescent total protein membrane staining (Revert^{TM} 700 Total protein stain), gray scale image; samples are labeled corresponding to sample identifiers in Figure S6, R183W corresponds to 25-B and R183W' to 176-B, R196H corresponds to 181-B, R196H' to 39-B (**B), (D)** Fluorescent ßCYA protein detection using IRDye800CW.

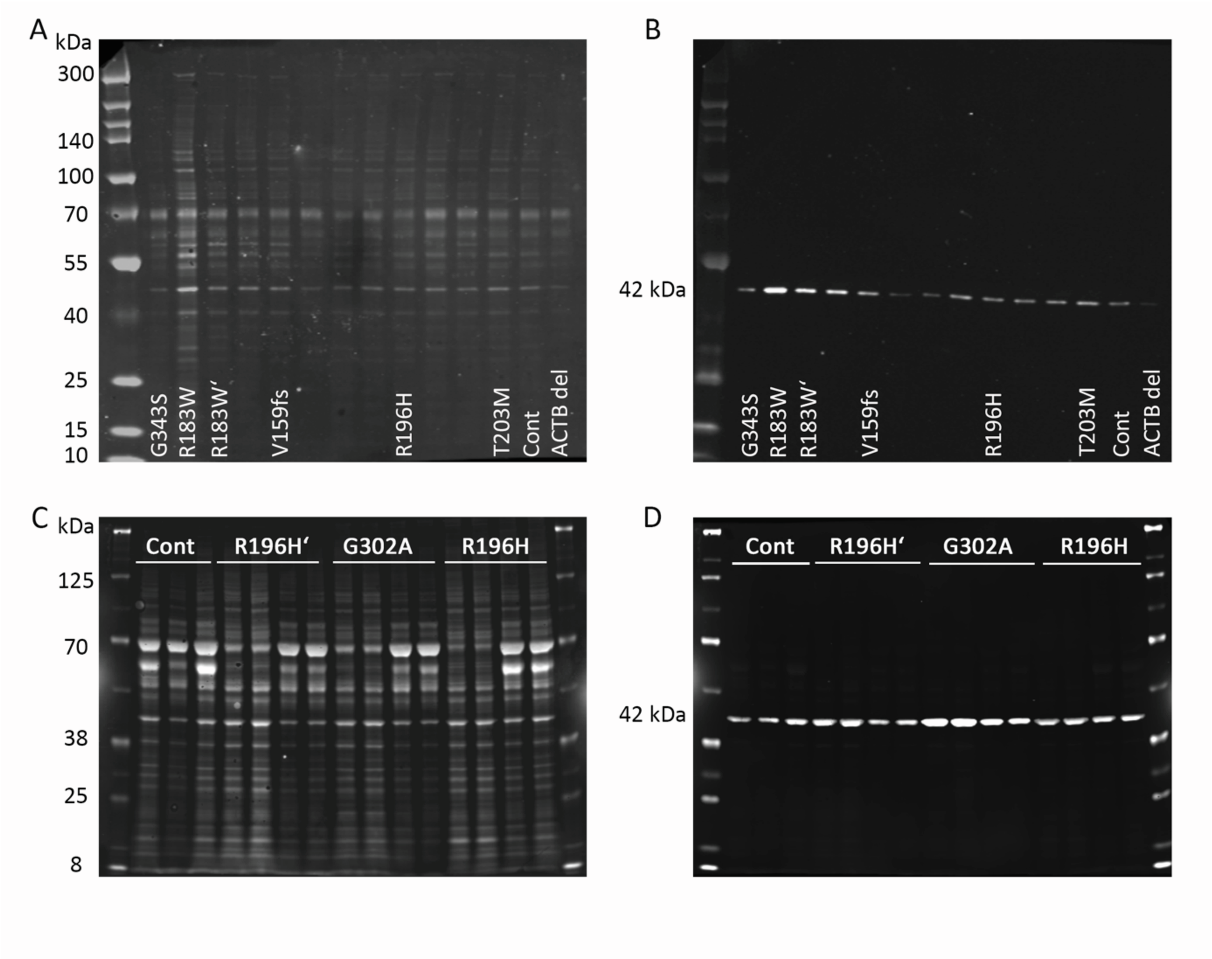

Figure S11. Pyrene–based bulk-polymerization and depolymerization experiments of CYA isoforms (5% pyrene–labeled).

(A, B) Representative traces of pyrene-polymerization experiments with wild type b-actin and mutants. Experiments were performed with pure actin mutants (A) or a 1:1 mixture of wild type and mutant actin (B) **(C)** Representative traces of pyrene-based dilution-induced depolymerization experiments performed with pure wild type b-actin and mutant proteins. (**D, E**) Representative traces of seeded pyrene-polymerization experiments with g-actin wild type and mutants. Experiments were performed with pure actin mutants (D) or a 1:1 mixture of wild type and mutant actin (E). **(F)** Representative traces of pyrene-based dilution-induced depolymerization experiments performed with pure wild type g-actin and mutant proteins.

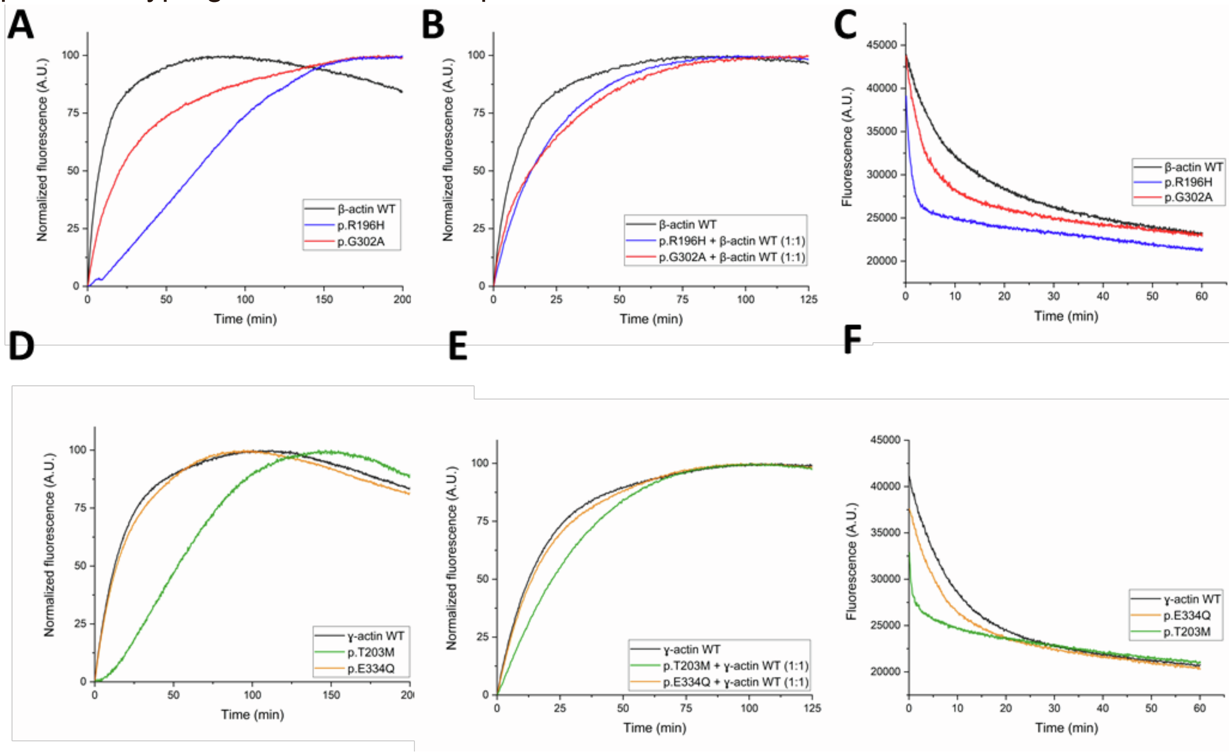

Figure S12. Expression profiles of the patient-derived and control fibroblasts.

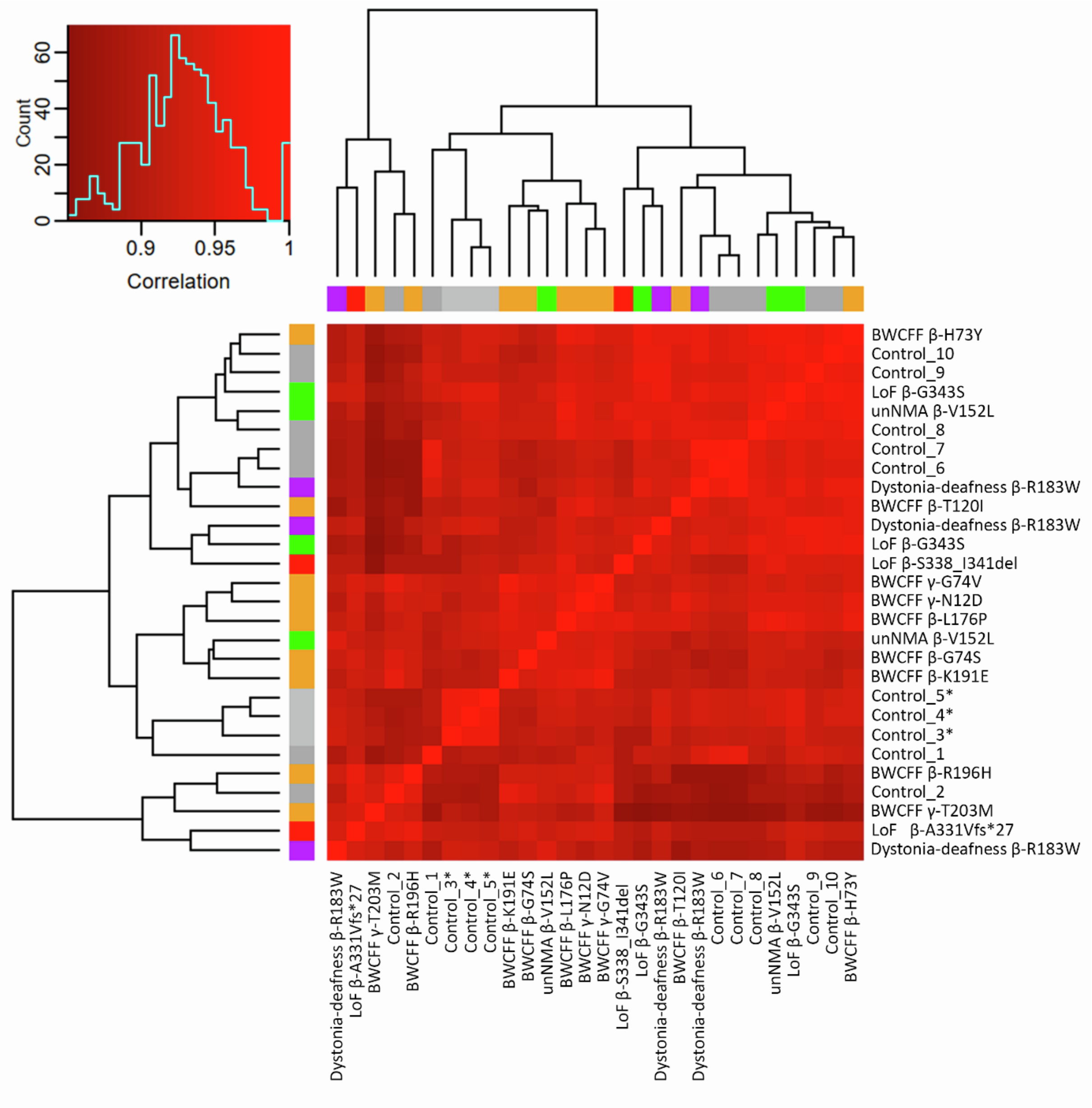

Figure S13. Principle component analysis of the average expression profile per patient.

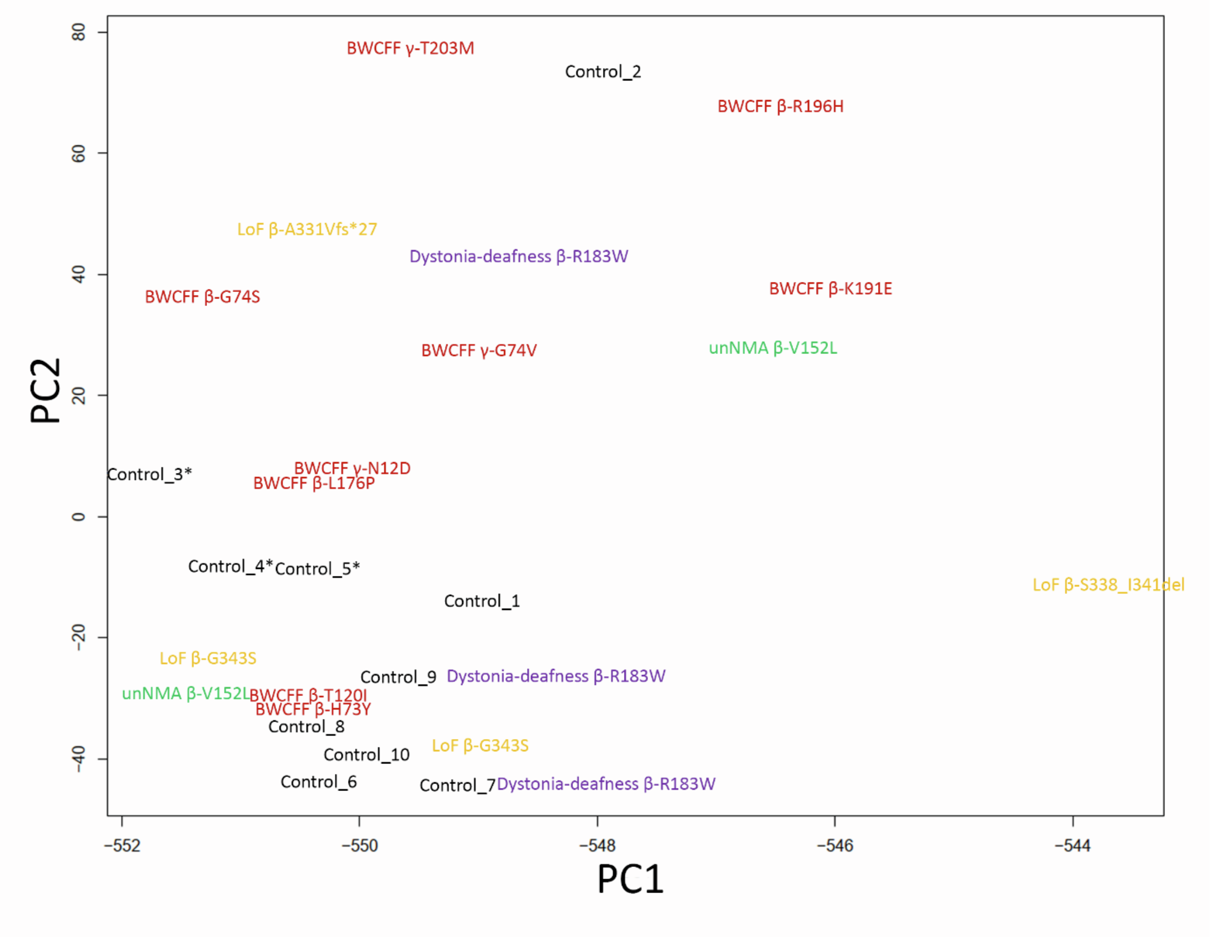

Figure S14. MRI images with cortical malformations typical for BWCFF

(A) T2 weighted axial image demonstrating fronto-temporal pachygyria and prominent perivascular spaces (age 10y); **(B)** T1 weighted axial image shows anterio predominant pachygyria and a thin band heterotopia in the occipital lobes with prominent perivascular spaces (age 1,5y); **(C)** T2 weighted coronal image with the bilateral single periventricular nodules (age 4m); scale bar 1cm.

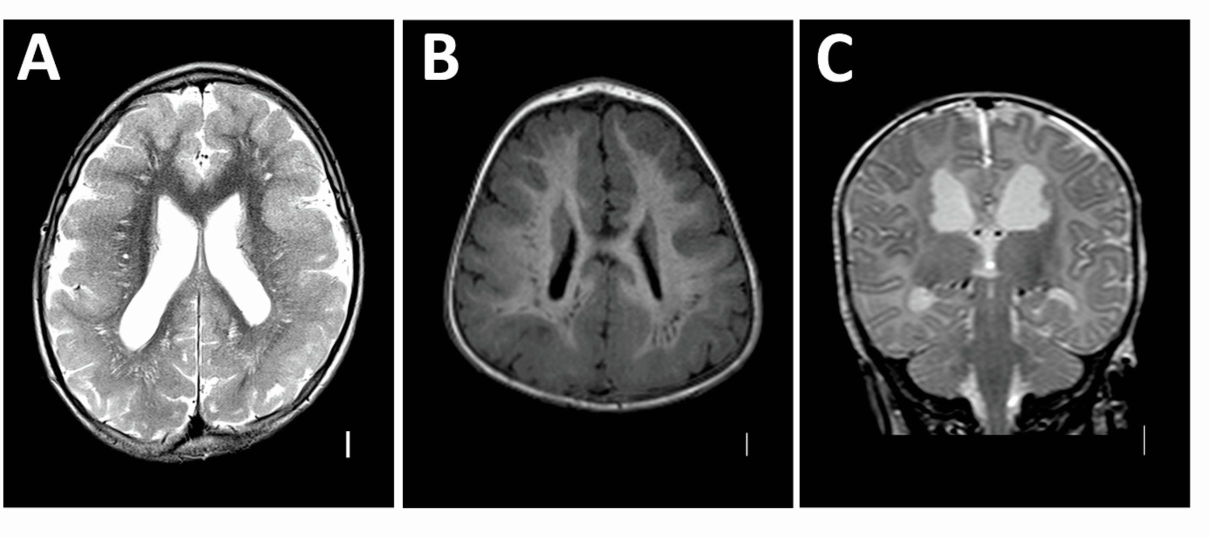

Figure S15. Spatial enrichment of variants by phenotype across the actin structure.

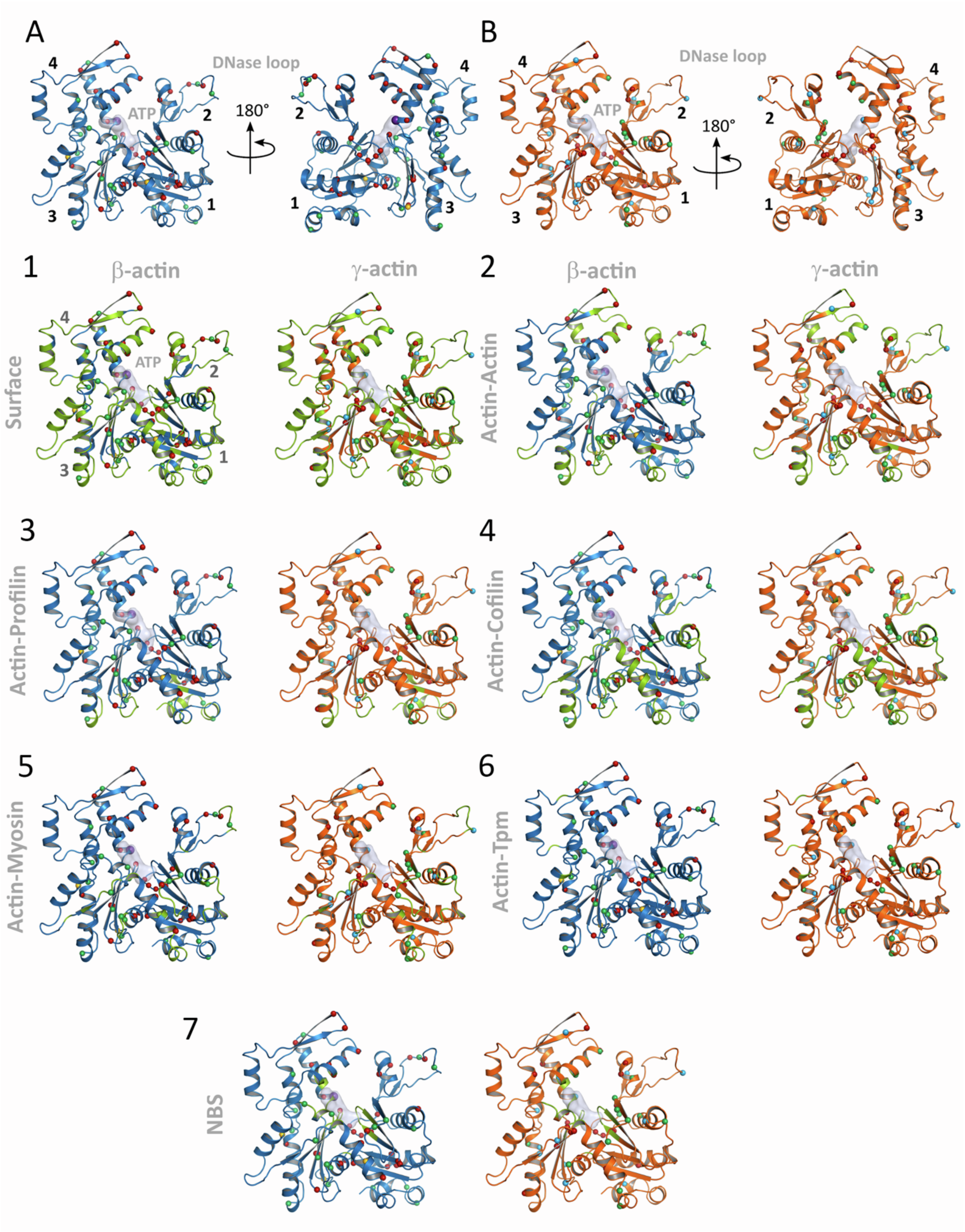

The structures of human βCYA **(A)** γCYA **(B)** were homology modeled using the Schrödinger Prime 4.0 and BioLuminate® applications (Schrödinger Inc., New York, NY). The sequences were retrieved from the Uniprot database (accession numbers: P60709 and P6326). The C-α atoms at mutation sites are shown as spheres. The different disease phenotypes associated with the site of mutation are indicated according to the color code used in Figure 2. Residues located on the surface are colored in

green (1); an interaction site is defined as the region within 5 Å of the binding partner and is colored in green: actin-actin interaction (2) profilin (3) cofilin (4), myosin (5), tropomyosin (6), phosphate and Mg^{2+}, as well as nucleotide coordination at the nucleotide binding site, NBS. (7)

Figure S16. Functional classification of non-muscle actinopathies.

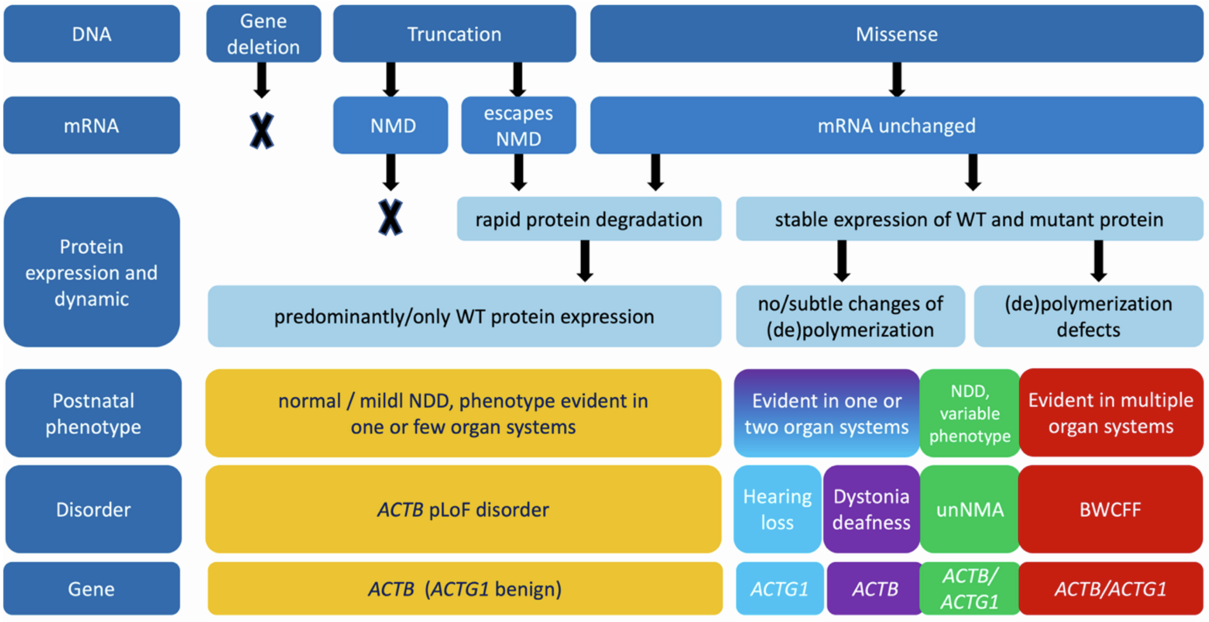

Based on our data, NMAs can be categorized into five clinical entities and three major functional groups First, genomic variants that result in decreased or absent expression of mRNA or production of unstable mutant actin. Such variants in *ACTB* are clinically associated with *ACTB* pLoF. ACTG1 variants of the first group are either benign or result with not-fully penetrant unNMA. Second, MVs that result in stable actin expression with severely impaired poly-/depolymerization dynamics. These variants in both *ACTB* and *ACTG1* are associated with BWCFF, pointing out the correlation with the abnormal postnatal presentation affecting in multiple organ systems. Third, MVs that result in stable expression of actin with normal or slightly abnormal polymerization dynamics. One of these variants in *ACTB* (R183W) leads to dystonia-deafness syndrome, and the others are associated with unNMA. Such variants in *ACTG1* can also cause unNMA. Still, several of these MVs result in progressive hearing loss, suggesting that these group three variants are likely associated with the limited expression of the phenotype referable to only one organ system.

Supplemental Tables

Table S4. List of antibodies

Primary antibodies

Target	Specificity	Company	Catalog no. #	RRID	Dilution factor
β-CYA	Mouse monoclonal IgG_1 clone 4C2	bio-rad	CMCA5775GA	AB_2571580	1:50 (IF) 1:7500 (WB)
g-CYA	Mouse monoclonal IgG_{2b} clone 2A3	bio-rad	MCA5776GA	AB_2571583	1:100 (IF) 1:7500 (WB)
Ki-67	Rabbit polyclonal	abcam	ab15580	AB_443209	1:300 (IF)
Tuj1	Mouse monoclonal	BioLegend	#801201	AB_2313773	1:300 (IF)
Pan-Actin	Mouse monoclonal	Novus Biologicals	NB600-535	AB_2222881	1:200 (IF) 1:2500 (WB)
SOX2	Goat polyclonal	R+D Systems	AF2018	AB_355110	1:300 (IF)
DAPI		Roche	10236276001		1:1000
IRDye® 800CW		Li-COR	926-32210		1:15000

Secondary antibodies

Host/ Target	Isotype	Conjugate	Company	Catalog no. #	RRID	Dilution factor
Goat anti Mouse	Mouse IgG, Fcg Subclass 1 Specific	AlexaFluor 488	Jackson Immunoresearch	115-545-205	AB_2338854	1:200
Goat anti Mouse	Mouse IgG, Fcg Subclass 2b Specific	CY5	Jackson Immunoresearch	115-175-207	AB_2338717	1:50
Donkey anti Mouse	Donkey IgG	AlexaFluor 488	Thermo Fisher	A-21202	AB_141607	1:500
Donkey anti Rabbit	Donkey IgG	AlexaFluor 555	Thermo Fisher	A-31572	AB_162543	1:500
Donkey anti Goat	Donkey IgG	AlexaFluor 647	Thermo Fisher	A-21447	AB_2535864	1:500
Donkey anti Rat	Donkey IgG	AlexaFluor 488	Thermo Fisher	A-21208	AB_2535794	1:500

Table S5. GestaltMatcher analysis - positive predictive values for all pairwise contrasts presented in Figure S4.

Group 1	Group 2	Mean pairwise distance	% above threshold	PPV in interval (%)
BWCFF	ACTB_LoF	0.977	100	92.99
ACTB_BWCFF	ACTG1_BWCFF	0.827	0	8.74
ACTB_unNMA	ACTG1_unNMA	0.924	92	71.68
BWCFF	BWCFF_unNMA	0.853	1.01	18.77
BWCFF	unNMA	0.916	92	63.91

NMA clinical consortium

Name	Affiliation 1	Affiliation 2
Andrea Accogli	Department of Specialized Medicine, Division of Medical Genetics, McGill University Health Centre, Montreal, Canada	Department of Human Genetics, McGill University, Montreal, Canada
Maria Albers	Department of Genetics, University Medical Center Utrecht, Utrecht, Netherlands	
Fowzan Alkuraya	Department of Genetics, King Faisal Specialist Hospital and Research Center, Riyadh, Saudi Arabia	
Neophytos Apeshiotis	Praxis für Genetik, Eckert-Str. 12, Braunschweig, Germany	
Diana Baralle	Faculty of Medicine, University of Southampton, University of Southampton, Southampton, United Kingdom	
Carmen Barba	Neuroscience Department, Meyer Children's Hospital IRCCS, viale Pieraccini 24, 50139, Florence, Italy	Department of NEUROFARBA, University of Florence, viale Pieraccini 6, 50139, Florence, Italy
Allan Bayat	Department of Epilepsy Genetics and Personalized Medicine, Danish Epilepsy Centre, Dianalund, Denmark	Department of Clinical Genetics, Copenhagen University Hospital, Rigshospitalet, Copenhagen, Denmark
Andreas Benneche	Department of Medical Genetics, Haukeland University Hospital, Bergen, Norway	
Laura Bernardini	Medical Genetics Unit, IRCCS Casa Sollievo della Sofferenza Foundation, San Giovanni Rotondo (FG), Italy	
Saskia Biskup	Zentrum für Humangenetik Tübingen, Tübingen, Germany	
Nina Bögershausen	Institute of Human Genetics, University Medical Center Göttingen, Göttingen, Germany	
Knut Brockmann	Department of Pediatrics and Adolescent Medicine, University Medical Center Göttingen, Göttingen, Germany	
Nicola Brunetti-Pierri	Telethon Institute of Genetics and Medicine (TIGEM), Pozzuoli, Naples, Italy	Department of Translational Medicine, Federico II University, Naples, Italy
Peter Burfeind	Institute of Human Genetics, University Medical Center Göttingen, Göttingen, Germany	

Ruben Cabanillas	Cabanillas Precision Consulting, Zurich, Switzerland	Translational Medicine, T-Therapeutics, Cambridge, United Kingdom
Patricia Corriols-Noval	Department of Otorhinolaryngology, Hospital Universitario Marqués de Valdecilla, Santander, Spain	
Elke de Boer	Department of Human Genetics, Radboudumc, 6500 HB, Nijmegen, Netherlands	
Iris de Lange	Department of Genetics, University Medical Center Utrecht, Utrecht, Netherlands	
Charulata Deshpande	Manchester Centre for Genomic Medicine, St Mary's Hospital, Manchester University NHS Foundation Trust, Manchester, United Kingdom	
Marta Diñeiro	Instituto de Medicina Oncológica y Molecular de Asturias (IMOMA), Oviedo, Spain	
Emily Doherty	Carilion Clinic Children's Hospital, Roanoke, United States	
Julia Doll	Institut für Humangenetik, Biozentrum, Universität Würzburg, Würzburg, Germany	
Sofia Douzgou	Department of Medical Genetics, Haukeland University Hospital, Bergen, Norway	
Tracy Dudding-Byth	University of Newcastle, The NSW Genetics of Learning Disability Newcastle, Newcastle, Australia	
Nadja Ehmke	Institute of Medical Genetics and Human Genetics, Charité-Universitätsmedizin Berlin, Corporate member of Freie Universität Berlin and Humboldt-Universität zu Berlin, Berlin, Germany	
Katherine Fawcett	MRC Computational Genomics Analysis and Training Programme (CGAT), MRC Centre for Computational Biology, MRC Weatherall Institute of Molecular Medicine, John Radcliffe Hospital, Oxford, United Kingdom	Department of Population Health Sciences, University of Leicester, LE1 7RH, Leicester, United Kingdom
Carlos R. Ferreira	National Human Genome Research Institute, National Institutes of Health, 20892, Bethesda, United States	
Jan Fischer	Institute for Clinical Genetics, Medical Faculty and University Hospital Carl Gustav Carus, TUD Dresden University of Technology, Fetscherstrabe 78, 01311, Dresden, Germany	

Joel Fluss	Pediatric Neurology Unit, Paediatrics Subspecialties Service, Geneva Children's Hospital, Geneva, Switzerland	
Rocío González-Aguado	Department of Otorhinolaryngology, Hospital Universitario Marqués de Valdecilla, Santander, Spain	
Luitgard Graul-Neumann	Institute of Medical Genetics and Human Genetics, Charité-Universitätsmedizin Berlin, Corporate member of Freie Universität Berlin and Humboldt-Universität zu Berlin, Berlin, Germany	
Andrew Green	UCD School of Medicine and Medical Science, Children's Health Ireland (CHI) at Crumlin, Dublin, Ireland	
Renzo Guerrini	Neuroscience Department, Meyer Children's Hospital IRCCS, viale Pieraccini 24, 50139, Florence, Italy	Department of NEUROFARBA, University of Florence, viale Pieraccini 6, 50139, Florence, Italy
Asya Gusina	Laboratory of Cytogenetic, Molecular Genetic and Morphological Studies, National Research and Applied Medicine Centre 'Mother and Child'', Minsk, Belarus	
Ute Hehr	Center for Human Genetics, Regensburg, Germany	
Maja Hempel	Institute of Human Genetics, Heidelberg University, Heidelberg, Germany	
Michaela AH Hofrichter	Institut für Humangenetik, Biozentrum, Universität Würzburg, Würzburg, Germany	
Ivan Ivanovski	Medical Genetics Unit, Azienda USL-IRCCS di Reggio Emilia, Reggio Emilia, Italy	Institute of Medical Genetics, University of Zurich, Zürich, Switzerland
Wibke G. Janzarik	Department of Neuropediatrics and Muscle Disorders, Center for Pediatrics and Adolescent Medicine, Medical Center, Faculty of Medicine, University of Freiburg, Freiburg, Germany	
Diana Johnson	Department of Medical Genetics, National Health Service, NHS, Leeds, United Kingdom	
Marieke Joosten	Department of Clinical Genetics, Erasmus MC, Rotterdam, Netherlands	
Silke Kaulfub	Institute of Human Genetics, University Medical Center Göttingen, Göttingen, Germany	
Hyun Jung Kim	Department of Pediatrics, Eulji General Hospital, College of Medicine, Eulji University, Seoul, Republic of Korea	

Tjitske Kleefstra	Department of Human Genetics, Radboudumc, 6500 HB, Nijmegen, Netherlands	Donders Institute for Brain, Cognition and Behaviour, Radboud University, 6500 GL, Nijmegen, Netherlands
Eva Klopocki	Institut für Humangenetik, Biozentrum, Universität Würzburg, Würzburg, Germany	
Karla Krause	Institute for Clinical Genetics, Medical Faculty and University Hospital Carl Gustav Carus, TUD Dresden University of Technology, Fetscherstrabe 77, 01310, Dresden, Germany	
Alma Kuechler	Institute of Human Genetics, University Hospital Essen, University Duisburg-Essen, 45122, Essen, Germany	
Maria Kuzyakova	Institute of Human Genetics, University Medical Center Göttingen, Göttingen, Germany	
Martin W. Laass	Department of Pediatrics, Medizinische Fakultät Carl Gustav Carus, TUD Dresden University of Technology, Dresden, Germany	
Augusta Lachmeijer	Department of Genetics, University Medical Center Utrecht, Utrecht, Netherlands	
Wayne Lam	South East of Scotland Clinical Genetics Service, Edinburgh, United Kingdom	
Cha Gon Lee	Department of Pediatrics, Nowon Eulji Medical Center, Eulji University School of Medicine, Seoul, Republic of Korea	
Yun Li	Institute of Human Genetics, University Medical Center Göttingen, Göttingen, Germany	
Vanesa López-González	Sección de Genética Médica, Servicio de Pediatría, Hospital Clínico Universitario Virgen de la Arrixaca, Murcia, Spain	
Karen Low	Department of Clinical Genetics, University Hospitals Bristol NHS Foundation Trust, Bristol, United Kingdom	Centre for Academic Child Health, Bristol Medical School, University of Bristol, Bristol, United Kingdom,
Michael Lyons	Greenwood Genetic Center, Greenwood, United States	
Carlo Marcelis	Department of Clinical Genetics, Radboud University Medical Center, Nijmegen, Netherlands	
Francisco Martinez-Castellano	Unit of Genetics, Hospital Universitari i Politècnic La Fe. Valencia, Valencia, Spain	Genomics Unit, Instituto de Investigación Sanitaria La Fe, 46026, Valencia, Spain

Maarten Massink	Department of Genetics, University Medical Center Utrecht, Utrecht, Netherlands	
Kay Metcalfe	Manchester Centre for Genomic Medicine, St Mary's Hospital, Manchester University NHS Foundation Trust, Manchester, United Kingdom	
Donatella Milani	Fondazione IRCCS Ca' Granda Ospedale Maggiore Policlinico, Milan, Italy	
Shahida Moosa	Division of Molecular Biology and Human Genetics, Faculty of Medicine and Health Sciences, Stellenbosch University, Tygerberg, South Africa	Medical Genetics, Tygerberg Hospital, South Africa
Manuela Morleo	Telethon Institute of Genetics and Medicine (TIGEM), Pozzuoli, Naples, Italy	Department of Precision Medicine, University of Campania “Luigi Vanvitelli”, Naples, Italy
Teresa Neuhann	Institute of Human Genetics, University Medical Center Göttingen, Göttingen, Germany	
Thomas Neumann	Mitteldeutscher Praxisverbund Humangenetik, Halle, Germany	
Huu Nguyen	Department of Human Genetics, Ruhr-University Bochum, Bochum, Germany	
Vincenzo Nigro	Telethon Institute of Genetics and Medicine (TIGEM), Pozzuoli, Naples, Italy	Department of Precision Medicine, University of Campania “Luigi Vanvitelli”, Naples, Italy
Nuha Nimeri	Women’s Wellness and Research Center, NICU, Hamad Medical Corporation, Doha, Qatar	
Ewa Obersztyn	Department of Medical Genetics, Institute of Mother and Child, Warsaw, Poland	
Anne O'Donnell	Division of Genetics and Genomics, Boston Children's Hospital, Boston, United States	
Carmen Orellana	Unit of Genetics, Hospital Universitari i Politècnic La Fe. Valencia, Valencia, Spain	
Estrella Pallas	Department of Otorhinolaryngology, Hospital Álvaro Cunqueiro, Vigo, Spain	
Hans-Jürgen Pander	Institute of Clinical Genetics, Klinikum Stuttgart, Stuttgart, Germany	
Elena Parrini	Neuroscience Department, Meyer Children's Hospital IRCCS, viale Pieraccini 24, 50139, Florence, Italy	
Silke Pauli	Institute of Human Genetics, University Medical Center Göttingen, Göttingen, Germany	

Michele Pinelli	Department of Molecular Medicine and Medical Biotechnologies, University Federico II, Naples, Italy	Telethon Institute of Genetics and Medicine (TIGEM), Pozzuoli, Naples, Italy
Lina Quteineh	Division of Genetic Medicine, Geneva University Hospitals, Geneva, Switzerland	
Julia Rankin	Peninsula Clinical Genetics Service, Royal Devon and Exeter NHS Trust, Exeter, United Kingdom	
Monica Rosello	Unit of Genetics, Hospital Universitari i Politècnic La Fe. Valencia, Valencia, Spain	
Tamanna Roshan Lal	Genetics and Metabolism, Children's National Hospital, Washington, United States	
Vincenzo Salpietro	Department of Neuromuscular Disorders, Queen Square Institute of Neurology, University College London, WC1N 3BG, London, United Kingdom	Department of Biotechnological and Applied Clinical Sciences, University of L'Aquila, 67100, L'Aquila, Italy
Jens Schallner	Department of Neuropediatrics, TUD Dresden University of Technology, Dresden, Germany	
Gregor Schlüter	PRAENATAL, Nürnberg, Germany	
Julia Schmidt	Institute of Human Genetics, University Medical Center Göttingen, Göttingen, Germany	
Mariasavina Severino	Neuroradiology Unit, IRCCS Istituto Giannina Gaslini, Genoa, Italy	
Vandana Shashi	Department of Pediatrics, Division of Medical Genetics, Duke University Medical Center, Durham, United States	
Corinna Siegel	Institute of Human Genetics, Klinikum rechts der Isar, Technical University of Munich, Munich, Germany	Department of Clinical Genetics, MVZ Martinsried, Munich,
Margie Sinnema	Department of Clinical Genetics, Maastricht University Medical Center, Maastricht, Netherlands	
Anne Slavotinek	Division of Genetics, Department of Pediatrics, University of California, San Francisco, United States	Division of Human Genetics, Cincinnati Children's Hospital, 3333 Burnet Ave, Cincinnati OH 45229, United States
Sarah Smithson	Department of Clinical Genetics, University Hospitals Bristol NHS Foundation Trust, Bristol, United Kingdom	
Siddharth Srivastava	Department of Neurology, Boston Children's Hospital, Boston, United States	
Maja Svrakic	Northwell Health Department of Otolaryngology, New York, United States	

Lindsay Swanson	Department of Neurology, Boston Children's Hospital, Boston, United States	
Hannah Thomson	Hunter Genetics, The NSW Genetics of Learning Disability Newcastle, Newcastle, Australia	
Eduardo Tizzano Ferrari	Àrea de Genètica Clínica i Molecular, Hospital Vall d'Hebrón, Barcelona, Spain	
Annalaura Torella	Telethon Institute of Genetics and Medicine (TIGEM), Pozzuoli, Naples, Italy	Department of Precision Medicine, University of Campania “Luigi Vanvitelli”, Naples, Italy
Undiagnosed Diseases Network		
Irene Valenzuela Palafoll	Àrea de Genètica Clínica i Molecular, Hospital Vall d'Hebrón, Barcelona, Spain	
Yolande van Bever	Department of Clinical Genetics, ErasmusMC University Medical Center Rotterdam, 3015 GD, Rotterdam, Netherlands	
Ellen van Binsbergen	Department of Genetics, University Medical Center Utrecht, Utrecht, Netherlands	
Marjon van Slegtenhorst	Department of Clinical Genetics, ErasmusMC University Medical Center Rotterdam, 3015 GD, Rotterdam, Netherlands	
Nienke Verbeek	Department of Genetics, University Medical Center Utrecht, Utrecht, Netherlands	
Virginie Verhoeven	Department of Clinical Genetics, ErasmusMC University Medical Center Rotterdam, Rotterdam, Netherlands	
Barbara Vona	Institute of Human Genetics, University Medical Center Göttingen, Heinrich-Düker-Weg 12, 37073, Göttingen, Germany	Institute for Auditory Neuroscience and InnerEarLab, University Medical Center Göttingen, Robert-Koch-Str. 40, 37075, Göttingen, Germany
Dagmar Wahl	Medical Practice for Genetic Counselling, Center for Human Genetics and Laboratory Diagnostics Martinsried, Augsburg, Germany	
Luisa Weiss	Center for Human Genetics, Regensburg, Germany	

Gökhan Yigit	Institute of Human Genetics, University Medical Center Göttingen, Göttingen, Germany	DZHK (German Center for Cardiovascular Research), partner site Göttingen, Göttingen, Germany
Maha Zaki	Clinical Genetics Department, Human Genetics and Genome Research Institute, National Research Centre, Cairo, Egypt	

References

1. Cuvertino, S., Stuart, H.M., Chandler, K.E., Roberts, N.A., Armstrong, R., Bernardini, L., Bhaskar, S., Callewaert, B., Clayton-Smith, J., Davalillo, C.H., et al. (2017). ACTB Loss-of-Function Mutations Result in a Pleiotropic Developmental Disorder. Am J Hum Genet *101*, 1021-1033. 10.1016/j.ajhg.2017.11.006.
2. Latham, S.L., Ehmke, N., Reinke, P.Y.A., Taft, M.H., Eicke, D., Reindl, T., Stenzel, W., Lyons, M.J., Friez, M.J., Lee, J.A., et al. (2018). Variants in exons 5 and 6 of ACTB cause syndromic thrombocytopenia. Nature communications *9*, 4250. 10.1038/s41467-018-06713-0.
3. Baraitser, M., and Winter, R.M. (1988). Iris coloboma, ptosis, hypertelorism, and mental retardation: a new syndrome. J Med Genet *25*, 41-43.
4. Verloes, A. (1993). Iris coloboma, ptosis, hypertelorism, and mental retardation: Baraitser-Winter syndrome or Noonan syndrome? J Med Genet *30*, 425-426.
5. Ramer, J.C., Lin, A.E., Dobyns, W.B., Winter, R., Ayme, S., Pallotta, R., and Ladda, R.L. (1995). Previously apparently undescribed syndrome: shallow orbits, ptosis, coloboma, trigonocephaly, gyral malformations, and mental and growth retardation. Am J Med Genet *57*, 403-409. 10.1002/ajmg.1320570308.
6. Rossi, M., Guerrini, R., Dobyns, W.B., Andria, G., and Winter, R.M. (2003). Characterization of brain malformations in the Baraitser-Winter syndrome and review of the literature. Neuropediatrics *34*, 287-292. 10.1055/s-2003-44666.
7. Riviere, J.B., van Bon, B.W., Hoischen, A., Kholmanskikh, S.S., O'Roak, B.J., Gilissen, C., Gijsen, S., Sullivan, C.T., Christian, S.L., Abdul-Rahman, O.A., et al. (2012). De novo mutations in the actin genes ACTB and ACTG1 cause Baraitser-Winter syndrome. Nat Genet *44*, 440-444, S441-442. 10.1038/ng.1091.
8. Verloes, A., Di Donato, N., Masliah-Planchon, J., Jongmans, M., Abdul-Raman, O.A., Albrecht, B., Allanson, J., Brunner, H., Bertola, D., Chassaing, N., et al. (2015). Baraitser-Winter cerebrofrontofacial syndrome: delineation of the spectrum in 42 cases. Eur J Hum Genet *23*, 292-301. 10.1038/ejhg.2014.95.
9. Yates, T.M., Turner, C.L., Firth, H.V., Berg, J., and Pilz, D.T. (2017). Baraitser-Winter cerebrofrontofacial syndrome. Clin Genet *92*, 3-9. 10.1111/cge.12864.
10. Di Donato, N., Rump, A., Koenig, R., Der Kaloustian, V.M., Halal, F., Sonntag, K., Krause, C., Hackmann, K., Hahn, G., Schrock, E., and Verloes, A. (2014). Severe forms of Baraitser-Winter syndrome are caused by ACTB mutations rather than ACTG1 mutations. Eur J Hum Genet *22*, 179-183. 10.1038/ejhg.2013.130.
11. Eker, H.K., Derinkuyu, B.E., Unal, S., Masliah-Planchon, J., Drunat, S., and Verloes, A. (2013). Cerebro-Fronto-Facial Syndrome Type 3 With Polymicrogyria: a Clinical Presentation of Baraitser-Winter Syndrome. European journal of medical genetics. 10.1016/j.ejmg.2013.10.005.
12. Verloes, A., Drunat, S., Pilz, D., and Di Donato, N. (1993). Baraitser-Winter Cerebrofrontofacial Syndrome. In GeneReviews((R)), M.P. Adam, H.H. Ardinger, R.A. Pagon, S.E. Wallace, L.J.H. Bean, K. Stephens, and A. Amemiya, eds.
13. Gearing, M., Juncos, J.L., Procaccio, V., Gutekunst, C.A., Marino-Rodriguez, E.M., Gyure, K.A., Ono, S., Santoianni, R., Krawiecki, N.S., Wallace, D.C., and Wainer, B.H. (2002). Aggregation of actin and cofilin in identical twins with juvenile-onset dystonia. Ann Neurol *52*, 465-476. 10.1002/ana.10319.
14. Procaccio, V., Salazar, G., Ono, S., Styers, M.L., Gearing, M., Davila, A., Jimenez, R., Juncos, J., Gutekunst, C.A., Meroni, G., et al. (2006). A mutation of beta -actin that alters depolymerization dynamics is associated with autosomal dominant developmental malformations, deafness, and dystonia. Am J Hum Genet *78*, 947-960. 10.1086/504271.
15. Conboy, E., Vairo, F., Waggoner, D., Ober, C., Das, S., Dhamija, R., Klee, E.W., and Pichurin, P. (2017). Pathogenic Variant in ACTB, p.Arg183Trp, Causes Juvenile-Onset Dystonia, Hearing Loss, and Developmental Delay without Midline Malformation. Case Rep Genet *2017*, 9184265. 10.1155/2017/9184265.
16. Freitas, J.L., Vale, T.C., Barsottini, O.G.P., and Pedroso, J.L. (2020). Expanding the Phenotype of Dystonia-Deafness Syndrome Caused by ACTB Gene Mutation. Mov Disord Clin Pract *7*, 86-87. 10.1002/mdc3.12854.

17. Zhu, M., Yang, T., Wei, S., DeWan, A.T., Morell, R.J., Elfenbein, J.L., Fisher, R.A., Leal, S.M., Smith, R.J., and Friderici, K.H. (2003). Mutations in the gamma-actin gene (ACTG1) are associated with dominant progressive deafness (DFNA20/26). Am J Hum Genet *73*, 1082-1091. S0002-9297(07)61971-510.1086/379286.

18. van Wijk, E., Krieger, E., Kemperman, M.H., De Leenheer, E.M., Huygen, P.L., Cremers, C.W., Cremers, F.P., and Kremer, H. (2003). A mutation in the gamma actin 1 (ACTG1) gene causes autosomal dominant hearing loss (DFNA20/26). J Med Genet *40*, 879-884.

19. Rendtorff, N.D., Zhu, M., Fagerheim, T., Antal, T.L., Jones, M., Teslovich, T.M., Gillanders, E.M., Barmada, M., Teig, E., Trent, J.M., et al. (2006). A novel missense mutation in ACTG1 causes dominant deafness in a Norwegian DFNA20/26 family, but ACTG1 mutations are not frequent among families with hereditary hearing impairment. Eur J Hum Genet *14*, 1097-1105. 10.1038/sj.ejhg.5201670.

20. Teig, E. (1968). Hereditary progressive perceptive deafness in a family of 72 patients. Acta Otolaryngol *65*, 365-372. 10.3109/00016486809120977.

21. Sorrentino, U., Piccolo, C., Rigon, C., Brasson, V., Trevisson, E., Boaretto, F., Martini, A., and Cassina, M. (2021). DFNA20/26 and Other ACTG1-Associated Phenotypes: A Case Report and Review of the Literature. Audiol Res *11*, 582-593. 10.3390/audiolres11040052.

22. Kemerley, A., Sloan, C., Pfeifer, W., Smith, R., and Drack, A. (2016). A novel mutation in ACTG1 causing Baraitser-Winter syndrome with extremely variable expressivity in three generations. Ophthalmic Genet, 1-5. 10.3109/13816810.2016.1164196.

23. Morin, M., Bryan, K.E., Mayo-Merino, F., Goodyear, R., Mencia, A., Modamio-Hoybjor, S., del Castillo, I., Cabalka, J.M., Richardson, G., Moreno, F., et al. (2009). In vivo and in vitro effects of two novel gamma-actin (ACTG1) mutations that cause DFNA20/26 hearing impairment. Hum Mol Genet *18*, 3075-3089. 10.1093/hmg/ddp249.

24. Miyagawa, M., Nishio, S.Y., Ichinose, A., Iwasaki, S., Murata, T., Kitajiri, S., and Usami, S. (2015). Mutational spectrum and clinical features of patients with ACTG1 mutations identified by massively parallel DNA sequencing. Ann Otol Rhinol Laryngol *124 Suppl 1*, 84S-93S. 10.1177/0003489415575057.

25. Yuan, Y., Gao, X., Huang, B., Lu, J., Wang, G., Lin, X., Qu, Y., and Dai, P. (2016). Phenotypic Heterogeneity in a DFNA20/26 family segregating a novel ACTG1 mutation. BMC Genet *17*, 33. 10.1186/s12863-016-0333-1.

26. Bryan, K.E., Wen, K.K., Zhu, M., Rendtorff, N.D., Feldkamp, M., Tranebjaerg, L., Friderici, K.H., and Rubenstein, P.A. (2006). Effects of human deafness gamma-actin mutations (DFNA20/26) on actin function. J Biol Chem *281*, 20129-20139. 10.1074/jbc.M601514200.

27. Drummond, M.C., Belyantseva, I.A., Friderici, K.H., and Friedman, T.B. (2012). Actin in hair cells and hearing loss. Hear Res *288*, 89-99. 10.1016/j.heares.2011.12.003.

28. Rainger, J., Williamson, K.A., Soares, D.C., Truch, J., Kurian, D., Gillessen-Kaesbach, G., Seawright, A., Prendergast, J., Halachev, M., Wheeler, A., et al. (2017). A recurrent de novo mutation in ACTG1 causes isolated ocular coloboma. Hum Mutat *38*, 942-946. 10.1002/humu.23246.
