# Supplementary material for: Molecular genotype-phenotype correlation in ACTB- and ACTG1-related non-muscle actinopathies
Source: Am J Hum Genet. 2026 Jan 12;113(2):324–41. doi: 10.1016/j.ajhg.2025.12.007 (PMC13087417; doi:10.1016/j.ajhg.2025.12.007)
Supplement: Document S2. Article plus supplemental information [file mmc5.pdf]

Molecular genotype-phenotype correlation in *ACTB*- and *ACTG1*-related non-muscle actinopathies

Authors

Nataliya Di Donato, NMA Consortium,
Andrew Thom, ..., Dietmar J. Manstein,
Adrian S. Woolf, Siddharth Banka

Correspondence

didonato.nataliya@mh-hannover.de (N.D.D.),
siddharth.banka@manchester.ac.uk (S.B.)

Through systematic evaluation of 290 individuals with *ACTB* or *ACTG1* variants, we delineate eight non-muscle actinopathies with distinct clinical profiles. Clear genotype-phenotype correlations, characteristic facial gestalts, and organ involvement patterns support improved diagnostic classification, prognostication, and counseling, providing clinicians with a practical framework for managing these rare disorders.

Di Donato et al., 2026, The American Journal of Human Genetics *113*, 324–341
February 5, 2026 © 2025 The Authors. Published by Elsevier Inc. on behalf of American Society of Human Genetics.
https://doi.org/10.1016/j.ajhg.2025.12.007

CellPress

Molecular genotype-phenotype correlation in *ACTB*- and *ACTG1*-related non-muscle actinopathies

Nataliya Di Donato,[1,2,*] NMA Consortium, Andrew Thom,[3] Andreas Rump,[1,4] Johannes N. Greve,[5] Juan Cadiñanos,[6,7] Rocco Salvatore Calabrò,[8] Sara Cathey,[9] Brian Chung,[10] Heidi Cope,[11] Maria Costales,[12] Sara Cuvertino,[13] Philine Dinkel,[1] Kalliopi Erripi,[14] Andrew E. Fry,[15,16] Livia Garavelli,[17] Sabine Hoffjan,[18] Wibke G. Janzarik,[19] Insa Kreimer,[1] Grazia Mancini,[20] Purificacion Marin-Reina,[21] Andrea Meinhardt,[1] Indra Niehaus,[1] Daniela Pilz,[22] Ivana Ricca,[23] Fernando Santos Simarro,[24] Evelin Schrock,[1] Anja Marquardt,[5] Manuel H. Taft,[5] Kamer Tezcan,[25] Sofia Thunström,[26] Judith Verhagen,[20] Alain Verloes,[27] Bernd Wollnik,[28,29,30] Peter Krawitz,[31] Tzung-Chien Hsieh,[31] Michael Seifert,[32] Michael Heide,[33] Catherine B. Lawrence,[34,35] Neil A. Roberts,[36] Dietmar J. Manstein,[5,37] Adrian S. Woolf,[36] and Siddharth Banka[13,38,*]

Summary

Recent advances in Mendelian genomics reveal the importance of variant-level characterization of allelic disorders. Non-muscle actin isoforms, encoded by the genes *ACTB* and *ACTG1*, are the most abundant intracellular proteins, but historically, they are often regarded as merely being "housekeeping" molecules. Here, we illuminate the extraordinary clinical heterogeneity and complex pathobiology of genetic non-muscle actinopathies. To do this, we combine human genomics studies with molecular biology. Strikingly, variants in *ACTB* and *ACTG1* isoforms generate at least eight distinct clinical disorders. A subset of disease-associated missense variants causes dysregulated actin polymerization-depolymerization and neuronal migration defects. In contrast, nonsense, frameshift, and missense variants enhancing protein degradation cause milder phenotypes or are benign. These results emphasize the essential functional aspects of the non-muscle actin isoforms. Critically, they additionally constitute a template for the personalized genetic variant-level-driven management of the pleiotropic allelic single-gene disorders.

Introduction

The ability to make accurate diagnoses, reliably estimate prognoses, and having a deep understanding of underpinning molecular and disease mechanisms are prerequisites to developing precision medicine approaches for genetic disorders. Recent advances in Mendelian genomics have dismantled the "one-gene one-disorder

[1]Institute for Clinical Genetics, Medical Faculty and University Hospital Carl Gustav Carus, TUD Dresden University of Technology, Fetscherstraße 74, 01307 Dresden, Germany; [2]Department of Human Genetics, Hannover Medical School, Carl Neuberg Str. 1, 30625 Hannover, Germany; [3]Division of Evolution, Infection and Genomics, School of Biological Sciences, Faculty of Biology, Medicine and Health, University of Manchester, Manchester, UK; [4]University Institute for Medical Genetics, Klinikum Oldenburg, Oldenburg, Germany; [5]Institute for Biophysical Chemistry and Structural Biochemistry, Hannover Medical School, Carl-Neuberg-Str. 1, 30625 Hannover, Germany; [6]Instituto de Medicina Oncológica y Molecular de Asturias IMOMA, Oviedo, Spain; [7]Fundación Centro Médico de Asturias, Oviedo, Spain; [8]IRCCS Centro Neurolesi Bonino-Pulejo, Messina, Italy; [9]Greenwood Genetic Center, Greenwood, IN, USA; [10]Department of Paediatrics and Adolescent Medicine, University of Hong Kong, Hong Kong, Hong Kong; [11]Department of Pediatrics, Division of Medical Genetics, Duke University Medical Center, Durham, NC, USA; [12]Otorhinolaryngoly Department, Hospital Universitario Central de Asturias, Hospital Central de Asturias, Oviedo, Spain; [13]Division of Evolution and Genomic Sciences, Faculty of Biology, Medicine, and Health, School of Biological Sciences, University of Manchester, Manchester, UK; [14]Ophthalmology Department, University Hospital of Gothenburg, Sahlgrenska, Sweden; [15]Institute of Medical Genetics, University Hospital of Wales, Cardiff, UK; [16]Division of Cancer and Genetics, School of Medicine, Cardiff University, Cardiff, UK; [17]Medical Genetics Unit, Azienda USL-IRCCS di Reggio Emilia, 42123 Reggio Emilia, Italy; [18]Department of Human Genetics, Ruhr-University Bochum, Bochum, Germany; [19]Department of Neuropediatrics and Muscle Disorders, Center for Pediatrics and Adolescent Medicine, Medical Center, Faculty of Medicine, University of Freiburg, Freiburg, Germany; [20]Department of Clinical Genetics, Erasmus MC University Medical Center Rotterdam, 3015 GD Rotterdam, the Netherlands; [21]Dysmorphology and Clinical Genetics, Department of Neonatology, Hospital Universitari i Politècnic La Fe. Valencia, Valencia, Spain; [22]West of Scotland Clinical Genetics Service, Queen Elizabeth University Hospital, Glasgow, UK; [23]Molecular Medicine for Neurodegenerative and Neuromuscular Diseases Unit, IRCCS Stella Maris Foundation, Pisa, Italy; [24]Institute of Medical and Molecular Genetics, Hospital La Paz Institute for Health Research, Madrid, Spain; [25]Department of Genetics, Kaiser Permanente, Sacramento, CA, USA; [26]Department of Clinical Genetics and Genomics, Sahlgrenska University Hospital, Gothenburg, Sweden; [27]Department of Genetics, APHP-Robert DEBRE University Hospital, Denis Diderot School of Medicine, Paris University, Paris, France; [28]Institute of Human Genetics, University Medical Center Göttingen, Göttingen, Germany; [29]DZHK German Center for Cardiovascular Research, Partner Site Göttingen, Göttingen, Germany; [30]Cluster of Excellence "Multiscale Bioimaging: from Molecular Machines to Networks of Excitable Cells" MBExC, University of Göttingen, Göttingen, Germany; [31]Institute for Genomic Statistics and Bioinformatics, University Hospital Bonn, Rheinische Friedrich-Wilhelms-Universität Bonn, Bonn, Germany; [32]Institute for Medical Informatics and Biometry IMB, Carl Gustav Carus Faculty of Medicine, TU Dresden, Fetscherstraße 74, 01307 Dresden, Germany; [33]German Primate Center, Leibniz Institute for Primate Research, Göttingen, Germany; [34]Division of Neuroscience and Experimental Psychology, and Geoffrey Jefferson Brain Research Centre, Faculty of Biology, Medicine and Health, Manchester Academic Health Science Centre, University of Manchester, Manchester, UK; [35]Geoffrey Jefferson Brain Research Centre, Northern Care Alliance NHS Foundation Trust, Manchester Academic Health Science Centre, University of Manchester, Manchester, UK; [36]Division of Cell Matrix Biology and Regenerative Medicine, School of Biological Sciences, Faculty of Biology, Medicine and Health, University of Manchester, Manchester, UK; [37]Division for Structural Biochemistry, Hannover Medical School, Carl Neuberg Str. 1, 30625 Hannover, Germany; [38]Manchester Centre for Genomic Medicine, St. Mary's Hospital, Central Manchester University Hospitals NHS Foundation Trust, Manchester Academic Health Science Centre, Manchester, UK

*Correspondence: didonato.nataliya@mh-hannover.de (N.D.D.), siddharth.banka@manchester.ac.uk (S.B.)

https://doi.org/10.1016/j.ajhg.2025.12.007.

© 2025 The Authors. Published by Elsevier Inc. on behalf of American Society of Human Genetics.
This is an open access article under the CC BY license (http://creativecommons.org/licenses/by/4.0/).

paradigm."[1] This means that many genetic disorders will likely require characterization at the variant level, not just at the gene level. We attempted dissection of a complex set of allelic disorders caused by constitutional variants in two genes encoding for actin isoforms, which we here refer to collectively as non-muscle actinopathies (NMAs).

Actin is the most abundant intracellular protein, highly versatile and essential for numerous cellular processes. The mammalian actin gene family consists of four muscle-specific isoforms (*ACTA1* [MIM: 102610], *ACTA2* [MIM: 102620], *ACTC1* [MIM: 102540], and *ACTG2* [MIM: 102545]) and two ubiquitously expressed genes, *ACTB* (MIM: 102630) and *ACTG1* (MIM: 102560), coding for the highly conserved β- and γ-cytoplasmic actins (CYAs), respectively. Human βCYA and γCYA differ only in four out of 375 amino acids but possess different polymerization properties, localize in different parts of the cell, and display preferred interactions with different subsets of actin-binding proteins.[2]

NMAs listed in OMIM include *ACTB*-related Baraitser-Winter cerebrofrontofacial syndrome 1 (BWCFF1 [MIM: 243310]), dystonia-deafness syndrome 1 (DDS1 [MIM: 607371]),[3] thrombocytopenia 8 with dysmorphic features and developmental delay (MIM: 620475),[4] *ACTG1*-related BWCFF2 (MIM: 614583),[5,6] and dominant deafness 20/26 (MIM: 604717).[7,8] An additional documented NMA, *ACTB*-associated isolated ocular coloboma,[9] is not listed in OMIM. A neurodevelopmental-congenital malformation disorder due to loss-of-function *ACTB* variants[10] is included within BWCFF1. Furthermore, postzygotic somatic mosaic *ACTB* variants have been detected in Becker's nevus, segmental odontomaxillary dysplasia, and congenital smooth muscle hamartoma with or without hemihypertrophy.[11–13] Nevertheless, the full mutational and clinical spectrum of human monogenic disorders caused by *ACTB* or *ACTG1* variants has yet to be systematically explored. Challenges in assigning pathogenicity and in linking individual variants to specific NMAs hamper accurate diagnosis. Even after a specific NMA is diagnosed, clinical management and providing prognosis is challenging due to limited data. Moreover, the pathobiology of NMAs is poorly understood. For example, it is not known whether disease-causing missense variants (MVs) in NMAs are hypomorphic, dominant-negative, or gain-of-function. Because of these limitations, no specific treatments for these disorders exist.

Here, we undertake detailed studies of disease-causing variants in *ACTB* and *ACTG1*. We describe a cohort of 290 individuals with pathogenic or likely pathogenic (P/LP) variants and propose a variant- and phenotype-based classification system that delineates NMAs and defines their clinical spectrum. Using *in vitro* cellular and biochemical studies, we provide variant-level insights into the molecular mechanisms of NMAs.

Material and methods

Simulation of all possible nucleotide substitutions within genomic regions of *ACTB* and *ACTG1*

For each of the 1,128 reference alleles in *ACTB* transcript chr7:5527148–5530601 (hg38) (GenBank: NM_001101.5) and 1,128 in *ACTG1* transcript chr17:81509971–81512799 (hg38) (GenBank: NM_001614.5), all three possible single-nucleotide alterations were simulated *in silico*, resulting in a list of $1{,}128 \times 3 \times 2 = 6{,}768$ alternate alleles (ALT) for both transcripts (Table S1). The functional consequences of all possible single-nucleotide alterations were annotated according to Ensembl Variant Effect predictor.[14]

Compilation of *ACTB* and *ACTG1* population variants and public databases

We retrieved gnomAD v.3.1.1 (hg38) genomic variants from genomic regions chr7:5527148–5530601 (*ACTB*) and chr17:81509971–81512799 (*ACTG1*).[15] To prevent redundancy with v.3.1.1., the gnomAD v.2.1.1 (hg19) variants were downloaded for exomes only; exons were defined by gnomAD standard transcripts ENSG00000075624.9 (*ACTB*) and ENSG00000184009 (*ACTG1*), including gnomAD's default padding of 75 nt. The Ensembl tool "assembly converter" converted the genomic positions of gnomAD v.2 hg19 variants to hg38. We exploited each of the different gnomAD subsets separately: controls (2,148 variants), non-TOPMed (3,107 variants), non-neuro (3,476 variants), and non-cancer (3,605 variants). We retrieved 2,184 genomic variants passing TOPMed filter (i.e., flagged as PASS in VCF "FILTER" column) (Freeze8 on GRCh38, accessed on November 1, 2021) within the genomic regions chr7:5527148–5530601 (ACTB) and chr17:81509971–81512799 (ACTG1).[16] We retrieved 2,280 variants from https://grch38.pggsnv.org/index.html (accessed on July 31, 2021) within the genomic regions chr7:5527148–5530601 (*ACTB*) and chr17:81509971–81512799 (*ACTG1*).[17] 752 variants were retrieved from the COSMIC catalog using gene names *ACTB* and *ACTG1* (441 and 311 variants, respectively). The query for cBioPortal mutations (not including structural variants and copy-number alterations) was performed using a user-defined list containing HUGO gene symbols *ACTB* and *ACTG1* (accessed on July 31, 2021 with the query of 46,305 individuals, representing 48,834 samples in 188 studies).[18,19] This query identified 389 unique and a total number of 508 variants. ClinVar variants were retrieved with the NCBI search term using hg38 coordinates of both genes (accessed on November 3, 2021)[20]: (7[CHR] AND 5527148[CHRPOS]: 5530601[CHRPOS]) and (17[CHR] AND 81509971[CHRPOS]: 81512799[CHRPOS]). From LOVD the search for all variants in *ACTB* (GenBank: NM_001101.3 transcript reference sequence) and *ACTG1* (GenBank: NM_001614.3 transcript reference sequence) resulted in 110 and 195 variants, respectively (accessed on November 3, 2021).[21]

Clinical and genomic analysis of the cohort

Study approval

The study was approved by the Institutional Review Board (IRB) of TU Dresden (EK-127032017 and BO-EK-341062021) and the Central Manchester (02/CM/238) and local IRBs from referring physicians. All individuals consented to participation prior to participating. Written informed consent was also received for

all photographs of individuals included. The record of informed consent has been retained.

Compilation of the clinical cohort

Patients were recruited primarily at the Institute for Clinical Genetics, University Hospital Dresden (by N.D.D.) as part of the active registry within the EJPRD-funded PredACTINg project as well as at the Manchester Center for Genomic Medicine within the NHS sequencing projects (by S.B.) after the rare variant in *ACTB* or *ACTG1* was discovered in the clinical genetic testing. Additionally, affected individuals were referred to N.D.D. and S.B. by multiple clinicians for the second opinion regarding variant interpretation, management advice, or specifically for this study. Clinical and molecular data were collected with the standardized proforma (supplemental information). Clinical photographs were available for 160 persons and were independently assessed by four authors experienced in dysmorphology and specifically familiar with BWCFF (N.D.D., S.B., G.V.M., and D.P.). Additionally, PubMed was searched for publications using terms “ACTB” OR “ACTG1” OR “Baraitser-Winter syndrome” OR “Baraitser-Winter-Cerebrofrontofacial syndrome” OR “Dystonia-deafness syndrome” OR “ACTG1 hearing loss” OR “ACTB neurodevelopmental disorder” OR “ACTG1 neurodevelopmental disorder.” We extracted reported data from identified publications and added follow-up information where available. In addition, we screened the ClinVar database for (likely) pathogenic variants and variants of uncertain significance and contacted responsible laboratories requesting clinical information.

Genomic- and phenotypic-led approach for classification of the clinical cohort

Available clinical data, facial photos, and MR images of all individuals were systematically reassessed regarding their phenotypical assignment by one of the corresponding authors. For classification of the cohort into relevant NMA groups, first all individuals were separated according to the gene in which their variant was located. Predicted loss-of function (pLoF) variants and protein-altering variants (PAVs) (missense or in-frame) were then separated. Clinical features of persons with pLoF variants were manually analyzed. Facial images were available for 166 of 275 individuals with small single-nucleotide variants (SNVs). Definition of the facial gestalt is described separately in the next section. The identified clinical features identified from these individuals were used to identify individuals with PAVs whose phenotype overlapped with those with pLoF variants. Next, individuals with recurrent PAVs were identified, their clinical features were manually analyzed, and genotype-phenotype relationships were extracted. Clinical data from these individuals were used to generate diagnostic criteria for each NMA. These diagnostic criteria were applied to individuals with non-recurrent variants, and additional individuals were identified for each subgroup. The clinical and mutation spectrum of each NMA was compiled using the combined data. The approach is summarized in Figure S1.

Definition of the syndrome specific facial gestalt

Four medical geneticists experienced in dysmorphology who have seen multiple (>10) individuals with BWCFF (G.M., D.P., S.B., and N.D.D.) independently evaluated clinical pictures of 89 individuals with the pathogenic variants in *ACTB* and *ACTG1*. Images were copied into one file without mentioning the genotype or additional clinical data. Frontal facial photographs of different quality were available for all individuals. Lateral images were available for more than two-thirds of the individuals. Body images as well as hand and feet photographs were provided for less than the half of the individuals. Twelve additional individuals with the microdeletions encompassing the entire *ACTB* gene[10] were shown with the genotype. Individuals were grouped as having a typical BWCFF face, typical *ACTB* pLoF face (guided by the facial features associated with deletions), or not having typical features of any of both disorders. The phenotypes were discussed in several online meetings.

GestaltMatcher facial analysis

To assess similarity between cohorts, we utilized GestaltMatcher[22,23] to analyze 75 images spanning BWCFF ($n = 38$; including ACTB_BWCFF $n = 23$ and ACTG1_BWCFF $n = 15$), ACTB LoF ($n = 19$), unNMA ($n = 15$; ACTG1_unNMA $n = 6$ and ACTB_unNMA $n = 9$), and BWCFF_unNMA ($n = 3$). The dataset comprises affected individuals reported in this work and images previously published in the GestaltMatcher database[24] (GMDB; https://db.gestaltmatcher.org). We first encoded each image to a 512-dimensional feature vector by GestaltMatcher and further utilized t-distributed stochastic neighbor embedding (t-SNE)[25] to visualize the distribution of the four groups of subjects in two-dimensional space.

Moreover, we wanted to compare the similarities between each group and the control distribution sampled from GMDB. Because the facial phenotypic similarity between two persons was quantified by the cosine distance, when the distance was smaller, the two individuals were more similar as they are closer in the space. We then examined the mean pairwise cosine distance between individuals among the three groups. We sampled control distributions from 1,499 images with 321 different disorders in the GMDB that were not included in the training of GestaltMatcher and calculated the mean pairwise distances between two cohorts stemming (1) from the same syndrome and (2) from two different syndromes. We further derived a threshold to decide whether two cohorts stem from the same or different syndromes by receiver-operating characteristic analysis, resulting in a final threshold of $c = 0.896$. To assess the similarity between the two cohorts C_1 and C_2, we computed their mean pairwise cosine distance$d(C_1, C_2)$, and compared it to the threshold c. Additionally, we conducted 100 subsampling iterations from each cohort to generate subcohorts, calculating the mean pairwise cosine distance for each iteration. If at least 50% of these 100 subsampled comparisons yielded values above the threshold c, it would provide evidence suggesting that the two cohorts stem from different syndromes.

To quantify how distinct are the two cohorts (C_1 and C_2), we computed the positive predictive value (PPV) for an observed intercohort distance d that falls within the range of distances between sampled subgroups of C_1 and C_2: $d \in (\min d(C_1, C_2), \max d(C_1, C_2))$.[23] PPV was estimated from pooled control distributions built on the validation folds using $\text{PPV} = (\text{sensitivity} \times p)/(\text{sensitivity} \times p + (1 - \text{specificity}) \times (1 - p))$, where $\text{sensitivity} = P(\text{distance in range} \mid \text{different syndromes})$, $\text{specificity} = P(\text{distance outside range} \mid \text{same syndrome})$, and p is the pre-test probability that two cohorts are from different syndromes. We set $p = 0.5$ to reflect no prior information (equal prior odds of “same” vs. “different”). In this framework, a higher PPV indicates greater evidence that the two cohorts are truly distinct.

To assess whether each subgroup (unNMA, ACTB_unNMA, ACTG1_unNMA, BWCFF, and ACTB LoF) exhibits phenotypic cohesion, we computed the mean pairwise distance among images within each subgroup. As a reference, we simulated a control distribution by repeatedly sampling equally sized batches of

individuals from the GMDB drawn across different syndromes and computing their mean pairwise distances. Each subgroup's within-group mean distance was then located on this control distribution to derive an empirical left-tail percentile, which reflects how unusually similar the subgroup is relative to random batches of subjects. Lower mean distance and lower percentile indicate stronger intragroup similarity.

Structural modeling

The structures of human βCYA and γCYA were homology modeled using the Schrödinger Prime 4.0 and BioLuminate applications (Schrödinger, New York, NY). The sequences were retrieved from the UniProt database (accession numbers UniProt: P60709 and P63261). The crystal structure of *Bos taurus* β-actin (PDB: 2btf) was used as a template. Mismatched residues in the template were replaced with those in the target structures, and the strontium ion was replaced with a magnesium ion. Model optimization of the resulting structures was performed by a series of iterative rotamer prediction and energy minimization steps.

Fibroblast experiments

Establishment of the subject-derived and control fibroblast cultures

Primary dermal fibroblasts were obtained following 3-mm cutaneous punch biopsies and cultured in BIO-AMF-2 medium (Biological Industries USA, Cromwell, CT, USA). For subculturing, primary fibroblasts were washed twice with 1× Dulbecco's PBS (dPBS) and detached at 37°C for at least 3 min with 0.05% trypsin/EDTA (Gibco; Thermo Scientific, Waltham, MA, USA). Cells were resuspended in BIO-AMF-2 medium, seeded onto Corning plasticware (Corning, NY, USA), and maintained in BIO-AMF-2 medium at 37°C in the presence of 5% CO_2. Cultures were continued for a maximum of three passages and, thereafter, the cultures were cryopreserved in multiple cryovials for long-term storage at −150°C. 90% FBS + 10% dimethyl sulfoxide was used as a freezing medium. Thawing of the frozen cells was performed rapidly in a 37°C water bath. Thawed cells were centrifuged at 240 × *g* for 5 min and resuspended in fresh BIO-AMF-2 medium in a new flask. Only cultures in early passages (maximum seven passages) have been used in the experiments. Cultures were labeled with the actin amino acid change and a subject's ID corresponding to the ID in Table S2 (e.g., G343S 61-B).

Primary dermal fibroblasts from nine healthy adult individuals were obtained and stored following the same procedure described above. These cell lines are labeled as Control_1 and Control_2, as well as Control_6 through Control_12. Only cultures in early passages (maximum seven passages) were used in the experiments. Additionally, three control cell lines were acquired from the Coriell Biobank—GM00013 (passage 13), GM04390 (passage 11), and GM05294 (passage 8)—and labeled Control_3, Control_4, and Control_5, respectively. Coriell cultures were only used for the transcriptome analysis.

Cell lysis and western blot analysis

Cells grown to approximately 70% confluence were washed with room-temperature dPBS twice. 0.6 mL of RIPA buffer (Santa Cruz, sc-24948) was added to the monolayer cells in a T25 flask and gently rocked for 15 min at 4°C. Adherent cells were removed with a cell scraper, and the lysate was transferred into a microcentrifuge tube. Lysate was incubated for 5 min on ice before centrifugation at 14,000 × *g* for 10 min at 4°C, and the supernatant was collected. Total protein concentration was measured with the Qubit fluorometer and Qubit protein assay kit (Thermo Fischer Scientific). Approximately 7 μg of protein was separated on 4%–12% NuPAGE Bis-Tris gels (Thermo Fischer Scientific) by electrophoresis (90 V for approximately 2.5 h) under reducing conditions with 6 μL of ProSieve QuadColor pre-stained protein marker loaded. Proteins were blotted onto a 0.2-μm pore nitrocellulose membrane with the iBlot2 system (Thermo Fischer Scientific). Revert 700 Total Protein stain (LI-COR, 926-11011) was performed for normalization of target protein signal intensities, and images of wet membranes were acquired with the Odyssey SA LI-COR Infrared Imaging system in the 700-nm channel (LI-COR). Subsequently, membranes were blocked overnight at 4°C in 5% (w/v) skim milk in TBS-Tween 20 (TBS-T). On the next day, membranes were incubated with primary antibodies diluted in 0.5% (w/v) skim milk in TBS-T for 2 h at room temperature (see Table S4 for antibodies and dilutions used). After three washing steps with 0.5% (w/v) skim milk in TBS-T, membranes were incubated with secondary antibodies for 1 h at room temperature with three subsequent washes with TBS-T followed by three washes with TBS. Next, images of dried membranes were acquired again with the Odyssey Imaging system in the 800-nm channel. Analysis of western blot signals was performed with CLIQS Gel Image Analysis software (TotalLab). Target protein signals were normalized to corresponding total lane signals of total protein stain, and lastly the samples from affected individuals were normalized to the respective control cell line. Results were obtained from three different lysates of independent cell passages with up to three replicates for each sample. GraphPad Prism v.9.3.1 for Windows (GraphPad Software, La Jolla, CA, USA; http://www.graphpad.com/) was used for graphical illustration and statistical analysis. Data were tested for normal distribution using the Shapiro-Wilk test and subsequently analyzed using ordinary one-way ANOVA. Outlier analysis was performed using the ROUT method.

Transcriptome analysis of primary fibroblast cultures

Library preparation and sequencing of biological and technical replicates. Transcriptome analysis was done using primary fibroblast cultures from 15 individuals with missense variants in *ACTB*, three individuals with variants in *ACTG1*, and seven control cultures from healthy adult individuals (Control_1, Control_2, and Control_6 through Control_10) as well as three fibroblast cultures from Coriell Biobank (Control_3 through Control_5). All cultures were harvested at early passages of a maximum of seven, except for cultures from Coriell, which were available in the later passages (up to passage 15).

Fibroblasts were seeded in T75 flasks and harvested at ∼70% confluence. The culture was performed twice to produce biological replicates. RNA was extracted using the miRNeasy Mini Kit (Qiagen) according to the manufacturer's instructions. On-column DNA digestion was included to remove residual contaminating genomic DNA. All experiments were performed in triplicate, meaning that RNA was independently extracted three times from each culture. In total, we performed six library preparations per individual (affected individuals and control subjects). For library preparations, the TruSeq Stranded mRNA Library Prep Kit (Illumina) was used according to the manufacturer's protocol, starting with 1 μg of total RNA. All barcoded libraries were pooled and sequenced 2 × 75-bp paired-end on an Illumina NextSeq500 platform to obtain a minimum of 10

million reads per sample. Raw reads from Illumina sequencers were converted from bcl to fastq format using bcl2fastq (v.2.20) allowing for one barcode mismatch.

Bioinformatics pipeline. The quality of the obtained fastq files was initially checked by FastQC v.0.11.4 (https://www.bioinformatics.babraham.ac.uk/projects/fastqc/) followed by adapter removal and quality trimming using Trim Galore v0.4.2 (http://www.bioinformatics.babraham.ac.uk/projects/trim_galore/). Mapping of reads to the human reference genome (GRCh38 Ensembl release 95) was done using STAR v.2.5.3a with standard settings,[26] and duplicates were marked and removed using Picard tools v.1.141 (http://broadinstitute.github.io/picard/). Quality analysis of mapped reads was done using RSeQC v.3.0.0[27] to analyze read distributions across gene bodies. Raw read counts per gene were determined by counting gene-specific reads in exons of protein-coding genes using FeatureCounts v.1.5.3.[28] Finally, a gene-expression data matrix was created by removing genes without any reads and lowly expressed genes (less than 1 read per million in more than 50% of samples) followed by cyclic loess normalization,[29] resulting in normalized $\log_2$ counts per million for 12,772 protein-coding genes that were measured in each sample. The average gene-expression levels per affected individual or control individual are provided in Table S3.

Similarity of gene-expression profiles and differential gene-expression analysis. Principal-component analysis of average genome-wide gene-expression profiles of study subject and control samples was done to analyze whether the different disease and control groups form separate clusters (R function prcomp). Further, similarity of genome-wide gene-expression profiles of samples from affected individuals and control samples was determined by computing the Pearson correlation coefficient for each pair of samples utilizing the average expression profile of each subject and control sample (R function cor). The corresponding correlation matrix was visualized as a heatmap (Figure S12).

Protein production and characterization

Generation of plasmid and baculovirus

The pFastBac vectors carrying the sequence of interest were constructed and generated as described previously.[30] Mutations in the sequence were introduced via site-directed mutagenesis with oligonucleotides encoding the desired mutation. Baculovirus was generated as described in the Bac-to-Bac Baculovirus Expression System manual (Thermo, Waltham, MA, USA). In short, pFastBac vectors carrying the sequence of interest were transformed into DH10EMBacY *Escherichia coli* to generate the recombinant bacmid. Sf-9 insect cells were then transfected with the recombinant bacmid to generate the recombinant baculovirus. Production of the recombinant actin wild type (WT) or mutant was started by infecting 1.8×10^6 cells/mL with 1:50 of the corresponding virus stock. Cells containing the protein of interest were harvested 3 days post infection and stored at −80°C until used for purification.

Purification of recombinant cytoskeletal actin WT and mutants

Recombinant human cytoskeletal actin WT and mutants were purified from Sf-9 insect cells using an actin-thymosin-β4-His_8 fusion construct as described by Noguchi et al.[31] In short, Sf-9 cells were resuspended in lysis buffer (10 mM Tris [pH 7.8], 5 mM $CaCl_2$, 1.25% Triton X-100, 1 mM ATP, 100 mM KCl, 7 mM β-mercaptoethanol, 1 mM phenylmethylsulfonyl fluoride [PMSF], 100 μg/mL N_α-*p*-tosyl-L-arginine methyl ester, 80 μg/mL *N*-tosyl-L-phenylalanine chloromethyl ketone, 2 μg/mL pepstatin, and 5 μg/mL leupeptin) and sonicated to disrupt the cells. The lysate was cleared by centrifugation and the supernatant incubated with 2 mL of lysis-buffer-equilibrated Pure Cube NiNTA column material (Cube Biotech, Monheim am Rhein, Germany) per liter of expression culture for 2 h, rotating at 4°C. The material was washed with 25 column volumes of wash buffer 1 (10 mM Tris [pH 7.8], 5 mM $CaCl_2$, 10 mM imidazole, 200 mM KCl, and 1 mM ATP) followed by 25 column volumes of wash buffer 2 (10 mM Tris [pH 7.8], 5 mM $CaCl_2$, 10 mM imidazole, 50 mM KCl, and 1 mM ATP). The protein was eluted with 250 mM imidazole, and the purity of the eluate was verified via SDS-PAGE. Fractions that contained the majority of the protein were pooled and dialyzed against G-buffer (10 mM Tris [pH 7.8], 0.2 mM $CaCl_2$, 0.1 mM dithiothreitol [DTT], and 0.1 mM ATP) overnight to remove imidazole. The dialyzed sample was then digested with 1:300 weight/weight of α-chymotrypsin from bovine pancreas (Merck, Darmstadt, Germany) to remove the thymosin-β4-His_8 moiety including the linker to yield the pure actin with native N and C termini. The reaction was quenched after a minimum of 45 min by the addition of 1 mM PMSF. The exact time of digest strongly depends on the age of the used batch of α-chymotrypsin. The sample was concentrated to 10–15 mg/mL, and polymerization of actin was induced by the addition of 100 mM KCl and 5 mM $MgCl_2$. The polymerization reaction was incubated for at least 3 h at room temperature and then moved to 4°C overnight. On the following day, the F-actin was sedimented by centrifugation at 130,000 × g for 1 h at 4°C. The pellet was washed with G-buffer and finally resuspended in 0.5–1 mL of G-buffer using a Dounce homogenizer. The sample was dialyzed against a total of 5 L of G-buffer supplemented with 0.1 mM PMSF over 4 days. The buffer was changed at least three times over that period. After dialysis, the protein sample was centrifuged at 15,000 × g for 15 min to remove precipitate. The pure protein was flash frozen in liquid nitrogen and stored at −80°C.

Assays probing polymerization and depolymerization of actin

Pyrene-actin-based assays to monitor polymerization and depolymerization of actin filaments were performed as previously described[32,33] with some slight modifications. To determine the rate of actin polymerization, Mg^{2+}-ATP-G-actin was supplemented with 5% pyrene-labeled Mg^{2+}-ATP-G-actin (WT) as a tracer to a final concentration of 10 μM. 20 μL of this solution was placed in a black flat-bottom 96-well plate (BrandTech Scientific, USA). The polymerization reaction was monitored as a function of increasing pyrenyl fluorescence in a Synergy 4 microplate reader (BioTek Instruments, Winooski, USA) using the built-in filter set (excitation 340/30 nm, emission 400/30 nm). Polymerization was induced by applying 80 μL of 1.25× polymerization buffer to a final concentration of 10 mM Tris (pH 7.8), 100 mM KCl, 5 mM $MgCl_2$, 0.5 mM EGTA, 0.1 mM DTT, and 0.1 mM ATP using the built-in pipetting function. To determine the rate of depolymerization, Mg^{2+}-ATP-G-actin was polymerized at 20 μM in the presence of 5% pyrene-labeled Mg^{2+}-ATP-G-actin (WT) overnight at 4°C. 3 μL of the F-actin solution was placed in a black flat-bottom 96-well plate and rapidly diluted by applying 297 μL of G-buffer (10 mM Tris [pH 7.8], 0.2 mM $CaCl_2$, 0.1 mM DTT, and 0.1 mM ATP). The dilution-induced depolymerization of the actin filaments was monitored using the settings mentioned above. The apparent half-time of the polymerization and depolymerization reaction was calculated by applying a single-exponential fit to the kinetic traces.

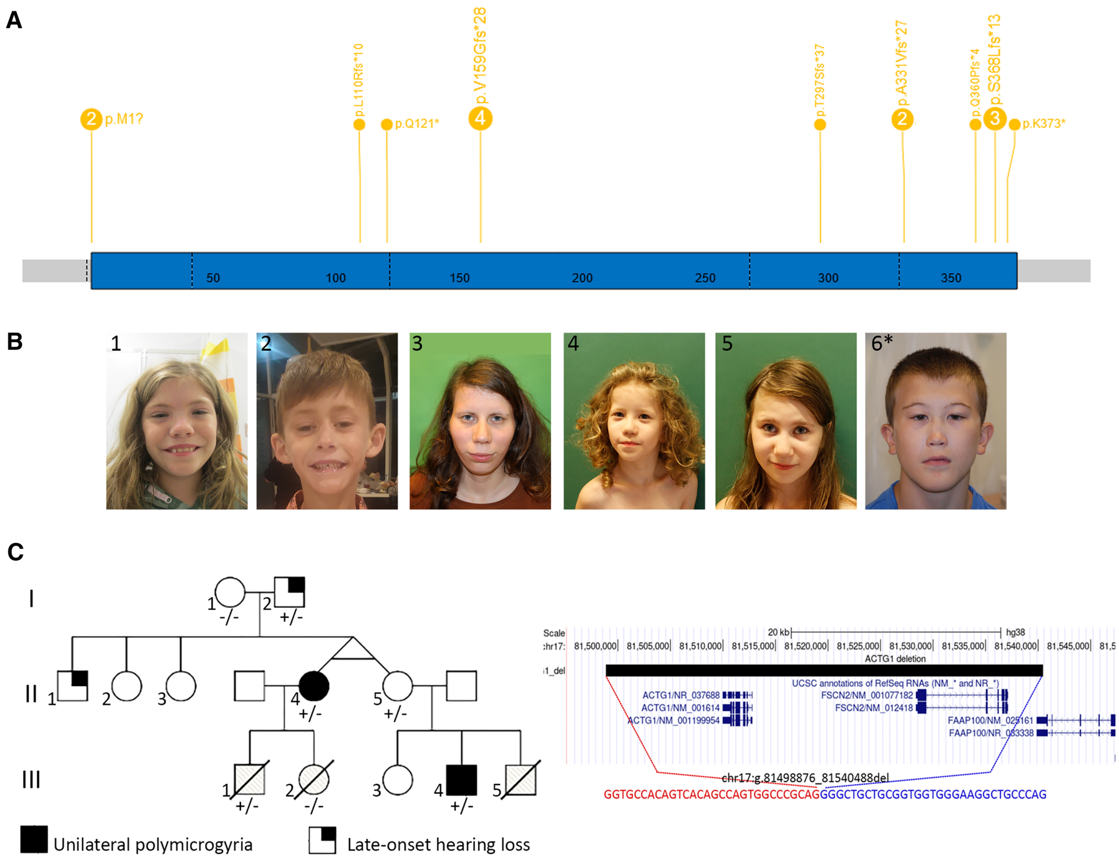

Figure 1. Definition of the haploinsufficiency of *ACTB* and *ACTG1*
(A) Number and distribution of truncating variants (start loss, stop gain, frameshift) in *ACTB*.
(B) Facial gestalt in individuals with the *ACTB* whole-gene deletions (1 and 2) and familial presentation of the *ACTB* stop gain (3–5). Note the resemblance of the facial gestalt of the last individual (6), although he carries a missense variant in *ACTB*.
(C) Segregation and intrafamilial variability of microdeletion of *ACTG1* with the pedigree with the affected aunt (II.4) and nephew (III.4) with unilateral polymicrogyria on the left; +/− indicates the carrier status. Right: the UCSC genome hg38 browser view demonstrating a microdeletion encompassing *ACTG1* and *FSCB2*; coordinates of the last normal probes are shown in red with the precise breakpoint mapping in blue (first and last missing bases), including junction sequence shown below.

Thermofluor assay

Thermal stability of WT and mutant proteins was assessed using the Thermofluor assay. The assay utilizes the fluorescent dye Sypro Orange, which shows an increase in fluorescent intensity upon binding to hydrophobic core regions of proteins that become exposed during thermal denaturation. Ca^{2+}-ATP-G-actin was converted to Mg^{2+}-ATP-G-actin by incubation with magnesium-exchange buffer (10×, 10 mM EGTA and 1 mM $MgCl_2$) for 2 min on ice prior to the experiment. 0.2 mg/mL Mg^{2+}-ATP-G-actin and 5× Sypro Orange (Life Technologies, Carlsbad, USA, stock: 5000×) were mixed in assay buffer (10 mM Tris [pH 7.8], 0.1 mM $MgCl_2$, 1 mM EGTA, 0.1 mM DTT, and 0.1 mM ATP) to a final volume of 25 μL. The samples were placed in a MicroAmp 48-well plate (Applied Biosystems, Waltham, MA, USA). The change in fluorescence intensity over a linear temperature gradient (1°C/min) was measured in a StepOne Real-Time PCR System (Applied Biosystems). The melting temperature was derived from the peak value of the first derivative of the melting function.

Nucleotide exchange assay

The rate of nucleotide exchange of Mg^{2+}-ATP-G-actin was determined as previously described[34] using the fluorescent ATP analog ε-ATP (Jena Bioscience, Jena, Germany).

Results

ACTB and *ACTG1* variants have dissimilar population profiles

To study the profile and consequences of the variants in the NMA genes, we simulated all possible *ACTB* and *ACTG1* single-nucleotide changes and compared them with known variants from population databases, representing ∼431,130 individuals.[15–17] Simulated variant counts per gene were similar overall, but we observed several differences between the non-synonymous, synonymous, and non-coding population variant counts of the two genes, which are unlikely to be the result of differences in mutation acquisition potential (Figures S2 and S3; Table S1). For example, the population dataset included only one individual with pLoF *ACTB* variant (probability of LoF intolerance [pLI] = 0.99) but >20 individuals with truncating *ACTG1* variants (pLI = 0)[35] (Figures 1A and S2A). Both genes are highly constrained for population MVs (Table S1A), but *ACTB* is more intolerant (43 distinct MVs, 0.02% population

frequency) than *ACTG1* (149 MVs, 0.05% population frequency) (Table S1).[35]

Next, we compiled clinical information from 290 individuals (125 new) with *ACTB* or *ACTG1* variants classified as P/LP[36] in various diagnostic laboratories in multiple countries. This "NMA cohort" comprised 275 individuals with SNVs in *ACTB* ($n = 145$) or *ACTG1* ($n = 130$) and 15 individuals with small deletions removing the entire *ACTB* or *ACTG1* gene (Table S2). The cohort's age range spanned 20 weeks gestation to 60 years, with 54% males and 46% females. The phenotype profiles of individuals in the NMA cohort, as expected, were highly variable.

ACTB and *ACTG1* variants, therefore, have remarkably different population profiles. These observations support their different biological roles in humans. Our initial examination of the clinical data revealed impressive heterogeneity, indicating the need for systematic in-depth analysis.

ACTB and *ACTG1* LoF alleles have distinct clinical consequences

We devised a variant and phenotypic-led approach to study the clinical consequences of variations in the two genes (Figure S1). We first analyzed pLoF variants in our NMA cohort (Figure 1A). Previously, two conditions have been described to result from *ACTB* pLoF variants, i.e., a "pleiotropic developmental disorder" due to 7p22.1 chromosomal microdeletions or point variants,[10] which is included within BWCFF1 (#243310) in OMIM; and syndromic thrombocytopenia caused by point variants in the last exon, which has a separate OMIM entry (#620475).[4] We compared the phenotypes of individuals either with microdeletions encompassing *ACTB* or with individuals' *ACTB* pLoF variants expected to undergo or escape nonsense-mediated decay (NMD) (Figure 1B and Table S2). Individuals with LoF variants predicted to escape NMD were initially analyzed separately but exhibited a phenotype comparable to those with alleles subject to NMD. Across all three groups, the clinical presentations were highly overlapping and included mild to moderate neurodevelopmental delay or borderline to mild intellectual disability, behavioral abnormalities, mild microcephaly, short stature, and brain anomalies (such as heterotopia but not pachygyria) as well as various congenital malformations and variable thrombocytopenia (Note S1).

In contrast, the NMA cohort did not have any P/LP *ACTG1* pLoF point variants. However, we identified seven individuals with chromosome 17q25 deletions shorter than 1 Mb that encompassed *ACTG1*. Deletion carriers showed variable phenotypes ranging from normal intelligence to borderline intellectual disability with additional structural anomalies (Table S2). This included a 41.6-kb deletion including *ACTG1* and *FSCN2* segregating through three generations in mildly affected and healthy relatives (Figure 1C). A unilateral polymicrogyria was observed in two affected family members, both of whom are high-functioning and show only mild neurodevelopmental involvement. *FSCN2* encodes the actin-bundling protein fascin-2 that crosslinks actin filaments into bundles within dynamic membrane protrusions, and its pLI score is 0, indicating that its haploinsufficiency alone is unlikely to cause a monogenic disorder.[15]

ACTG1 shows a loss-of-function observed/expected upper bound fraction (LOEUF) of ~0.62, indicating no marked depletion of pLoF. Considering the prevalence of the *ACTG1* pLoF variants in the population datasets, *ACTG1* pLoF point variants are unlikely to be pathogenic. Chromosome 17q25.3 deletions involving *ACTG1* and flanking genes could cause a contiguous gene deletion syndrome with variable penetrance, which requires further studies.

ACTB or *ACTG1* missense and in-frame variants result in multiple NMAs

Next, applying a structured classification strategy (Figure S1) on individuals with missense or in-frame variants in the NMA cohort, we identified 73 individuals whom we categorized into *ACTB*-BWCFF1 and 40 with *ACTG1*-BWCFF2 (Figures 2A and 2B). BWCFF can be diagnosed in an individual with a (likely) pathogenic MV in *ACTB* or *ACTG1* if this individual presents with (1) the specific facial dysmorphism (typically including hypertelorism, high-arched eyebrows, ptosis, long palpebral fissures with everted lower lid, broad nasal tip, long smooth philtrum, large mouth with thin upper lip, grooved chin, and large vertically oriented ears) and/or (2) frontal predominant pachygyria. Expert consensus description of the specific features is summarized in Note S2.

Sixty individuals presented with *ACTG1*-isolated hearing loss (HL) and 13 with *ACTB*-DDS1. Detailed inspection of the clinical data of the remainder showed that eight individuals with five *ACTB* MVs/in-frame indels presented with features similar to those of individuals with *ACTB* pLoF variants (Figure 1B1–1B6). Here, the main criterion was the overlap of the subjects' facial gestalt with that observed in individuals carrying deletions encompassing *ACTB* or variants predicted to result in NMD. These defining features are detailed in Note S1. Importantly, none of these individuals presented with the frontal predominant pachygyria, a clinical symptom specific for BWCFF.

The remaining 65 individuals exhibited heterogeneous phenotypes that were not compatible with any of the previously mentioned actinopathies and did not show a recognizable facial gestalt even if some individuals presented with minor facial anomalies (grouped as unspecified non-muscle actinopathy or unNMA) (Figures 2C and 2D). We suggest that more distinct entities will be identified within this cohort in the future.

This classification revealed detailed clinical characteristics and variant spectrum for each NMA subtype (Notes S1–S5).

Note that multiple congenital malformations; e.g., heart, urinary tract, skeletal, and gastrointestinal anomalies, were more prevalent in the BWCFF group but were also observed in individuals with *ACTB* LoF and unNMA. Apart from

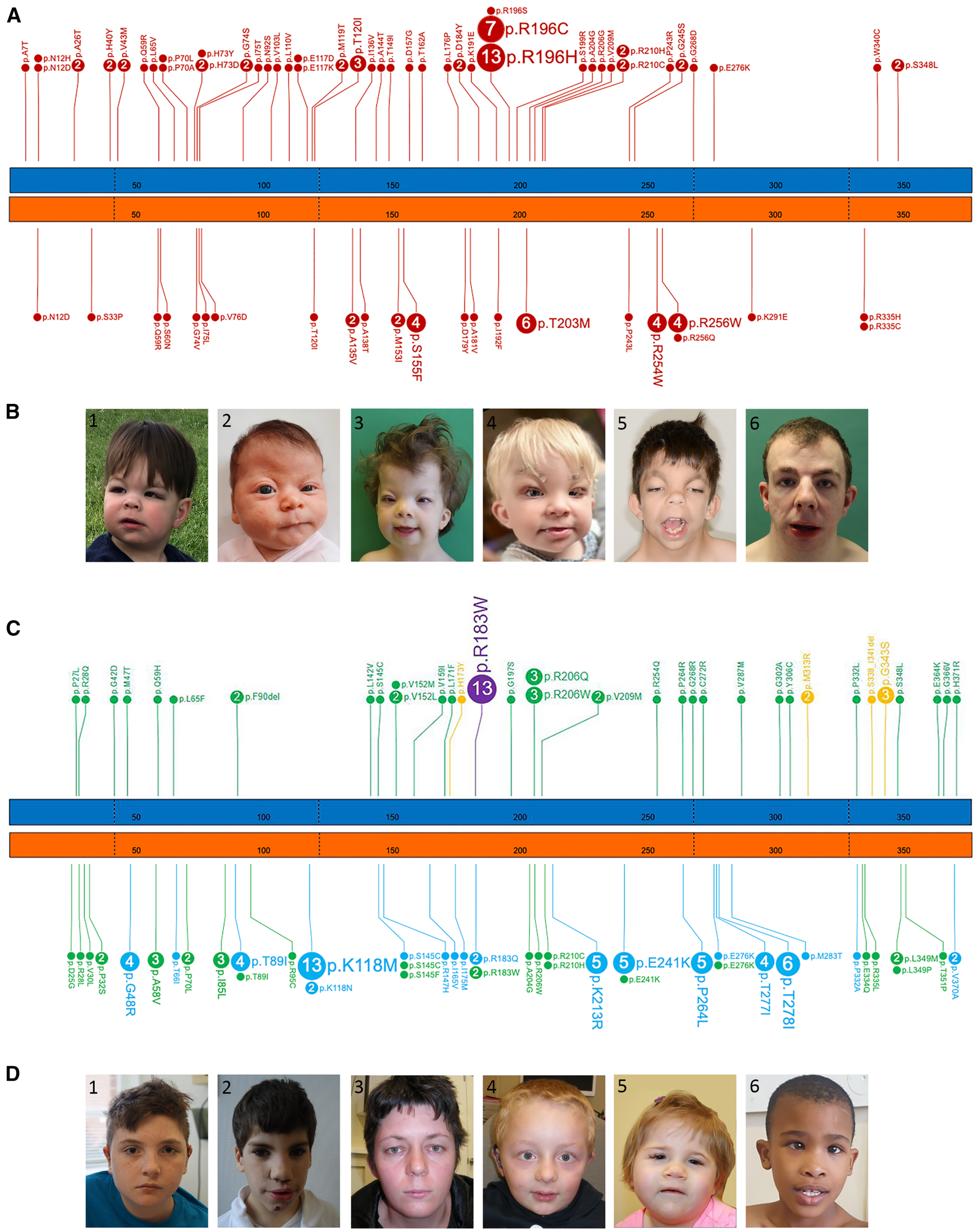

Figure 2. Missense variants in *ACTB* and *ACTG1* result in several distinct disorders

(A) Number and distribution of missense variants in *ACTB* and *ACTG1* resulting in the Baraitser-Winter cerebrofrontofacial syndrome (BWCFF); *ACTB* (blue bar) and *ACTG1* (orange bar) with every 50th amino acid numbered in both gene models. The borders of coding exons are marked with dashed lines.

(B) Representative facial gestalt in individuals with the BWCFF syndrome. Shown are individuals #1 (180-G), #2 (194-G), #3 (23-B), #4 (ID_09), #5 (47-B), and #6 (45-B), with subjects' IDs corresponding to those listed in Table S2. Note that individuals #5 and #6 carry the same *ACTB* hotspot variant at position Arg196, and that individuals in images 5 and 6 carry the same *ACTB* hotspot variant at position Arg196.

(C) Number, distribution, and clinical consequence of the missense variants in *ACTB* and *ACTG1* not resulting in BWCFF; gene models and amino acid numbering are as described above.

(D) Representative facial gestalt in individuals with unspecified non-muscle actinopathies (unNMA) due to variants in *ACTG1* (1–4) and *ACTB* (5 and 6). Note that images 3 and 4 show affected individuals from the same family. Shown are individuals #1 (35-G), #2 (119-B), #3 (47-G), #4 (48-G), #5 (20-B), and #6 (193-G), with subjects' IDs corresponding to those listed in Table S2.

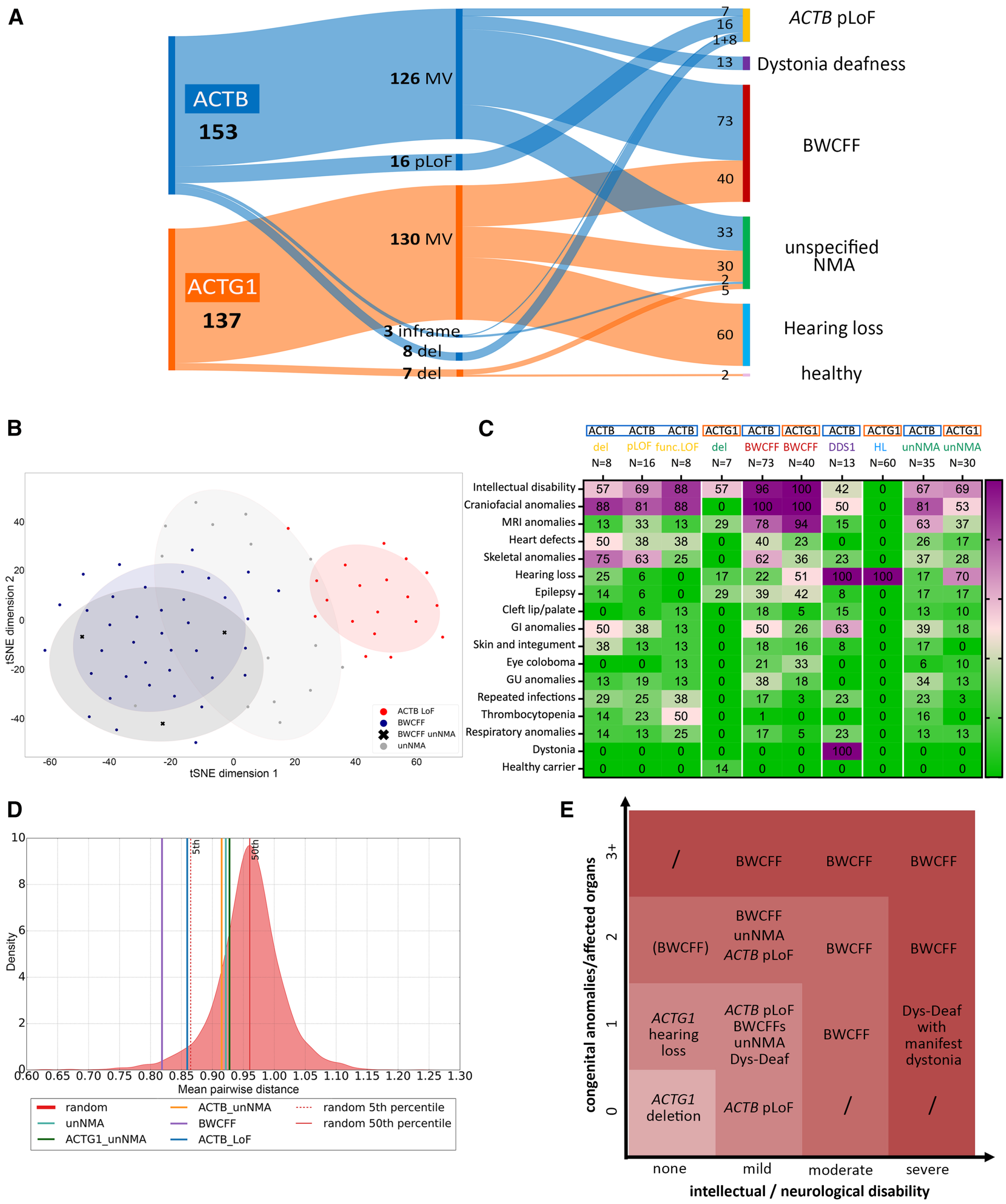

Figure 3. Broad clinical spectrum of non-muscle actinopathies

(A) Distribution of 290 pathogenic variants in *ACTB* and *ACTG1* within different disorders of NMA spectrum. BWCFF, Baraitser-Winter cerebrofrontofacial syndrome; del, whole-gene deletion; MV, missense variant; pLoF, putative loss of function.

(B) t-SNE visualization of three major NMA phenotypes in GestaltMatcher analysis. NMA-BWCFF indicates three individuals who were not reliably classified as BWCFF or unNMA based on the facial gestalt. Note that GestaltMatcher analysis allocated all three individuals within the BWCFF group.

(C) Phenogrid demonstrating frequencies of selected clinical features in individuals with pLoF in *ACTB*, microdeletions encompassing *ACTB* (*ACTB* del) or *ACTG1* (*ACTG1* del), and non-truncating *ACTB* variants resulting in unstable protein (*ACTB* func.LoF), BWCFF, dystonia-deafness syndrome (DD), hearing loss (HL), and unspecified non-muscle actinopathy (unNMA). Individuals with incomplete clinical information were counted as "clinical feature not present" except for gastrointestinal (GI) anomalies. For GI anomalies, individuals with unknown status were excluded from the calculation. The presence of dystonia was analyzed in individuals older than 18 years.

(D) Phenotypic similarity between subgroups of subjects. To assess whether individuals within each subgroup are phenotypically coherent, we simulated a control distribution by repeatedly computing the mean pairwise distance in batches of randomly sampled individuals from the GestaltMatcher database (i.e., individuals with different syndromes). Each subgroup's mean within-group distance $d(C)$ was then compared to this control distribution. The BWCFF subgroup showed the strongest intra-group similarity ($d = 0.82$, 1.61st percentile), followed by *ACTB* LoF ($d = 0.86$, 4.35th percentile). The unNMA-based subsets were less extreme but still below random expectation: *ACTB*_unNMA $d = 0.91$ (15.90th percentile), unNMA $d = 0.92$ (18.95th percentile), and *ACTG1*_unNMA

(legend continued on next page)

rare exceptions, we found that individuals with the same variants generally had highly overlapping clinical features and were classified into the same NMA subtype. Notably, however, we detected substantial differences in severity within the same subtype. We found the variant spectra to be much larger than previously recognized for all NMAs, apart from *ACTB*-DDS1, which seems to be caused only by *ACTB*:p.Arg183Trp. Interestingly, we observed that for certain positions, the same substitution in *ACTB* or in *ACTG1* can cause different NMAs.

These data show that *ACTB* or *ACTG1* protein-altering variants (PAVs) result in multiple NMAs. While specific variants exhibit reproducible genotype-phenotype correlations, we recognize that not all pathogenic variants in *ACTB* or *ACTG1* will necessarily act with similar predictability, and that phenotypic outcome may in some cases depend on variation at other loci and/or environmental influences.

Notably, the broad spread of PAVs across most NMAs suggests that these variants (with a possible exception of *ACTB*:p.Arg183Trp [c.547C>T]) are unlikely to be gain-of-function.[5] The differences of phenotypes of most *ACTB*/*ACTG1* PAVs versus LoF variants indicate that these missense/in-frame variants are unlikely to result in simple LoF. Hence, most of these variants possibly act in dominant-negative manner or result in loss of different subfunctions.[37] The heterogeneity of associated clinical features suggests that their cellular consequences could be context dependent.

Genetic and clinically led classification of NMAs is accurate and has important clinical implications

To test our clinically led classification (Figure 3A), we subjected our conclusions to objective validation. First, objective analysis of the craniofacial features using the photographs available to us using GestaltMatcher[22,38] confirmed our classification orthogonally (Figures 3B, 3D, S4, and S5; Table S5). Next, we compared the clinical features of all the NMAs (Figure 3C) and mapped the summarized trends in the severity of intellectual/neurological and the number of congenital anomalies or affected organs (Figure 3E). This revealed an extraordinarily broad clinical spectrum, ranging from individuals who appear to be unaffected (e.g., small chromosomal deletions across *ACTG1*) or relatively mildly affected (e.g., *ACTB* pLoF variants) to individuals who die *in utero*.

These results show that our genetic and clinically led classification of NMAs is reasonably accurate, and that the clinical differences between NMAs have implications for their accurate diagnosis, management, prognosis, and surveillance (expert opinion recommendations are summarized in Notes S1–S5).

ACTB MVs associated with LoF phenotype result in abnormal folding or impact actin thermal stability

Eight individuals with five *ACTB* MVs/in-frame indels presented with clinical features similar to those of individuals with *ACTB* pLoF variants. According to SpliceAI[39] predictions, none of these MVs are expected to affect *ACTB* splicing. Therefore, we hypothesized that some *ACTB* MVs might result in protein instability and degradation. We generated recombinant mutant βCYA proteins for *in vitro* characterization of a selection of these variants (Figure 4A). Protein purification could not be achieved for βCYA with two variants (p.Met313Arg [c.938T>G] or p.Ser338_Ile341del [c.1012_1023del]; Figure S6), indicating a possible defect with protein folding and/or stability. In contrast, other tested variants could be purified in sufficient quantity (Figure 4B). Among these, one variant (p.Gly302Ala [c.905G>C]) showed a significantly reduced stability (Figure 4C). We confirmed the previously observed mild reduction in stability for p.Arg183Trp[34] and detected no further differences in thermal stability between the wild-type and the remaining four MVs analyzed in this study (Figure 4D).

Using dermal fibroblasts from individuals with *ACTB*-pLoF disorder, we quantified the amount of βCYA and γCYA produced in the affected individuals' cells by western blot. Intriguingly, we did not observe a significant reduction of βCYA production in cells with *ACTB* deletion and LoF variant, nor with the variants with reduced thermal stability (Figure S7).

Summarizing our data, we suggest that the *ACTB*-related "pleiotropic developmental disorder"[10] is distinct from BWCFF1. The phenotype previously described as "syndromic thrombocytopenia"[4] substantially overlaps with the *ACTB*-related pleiotropic developmental disorder. In the expanded cohort, we observed a shared facial gestalt and confirmed thrombocytopenia in individuals with *ACTB* deletions, supporting that these entities represent a single clinical spectrum that should be merged under the term "*ACTB* pLoF disorder." The *ACTB* pLoF-associated disorder may involve three molecular mechanisms: (1) loss of *ACTB* transcription due to deletion of one allele, (2) NMD of mRNA containing a premature stop codon, and (3) production of unstable βCYA resulting from selected missense or in-frame *ACTB* variants.

Actin polymerization and depolymerization dynamics are significantly altered in BWCFF syndrome variants

Next, we set out to understand the mechanistic basis of BWCFF. Using selected recombinant proteins, we

$d = 0.93$ (22.20th percentile). These results indicate that BWCFF and *ACTB* LoF groups exhibit pronounced within-group similarity, whereas the unNMA subsets show modest yet non-random clustering relative to random controls.
(E) Severity grading of the disorders within the non-muscle actinopathy spectrum based on the number of congenital anomalies and/or affected organs and the severity of intellectual impairment.

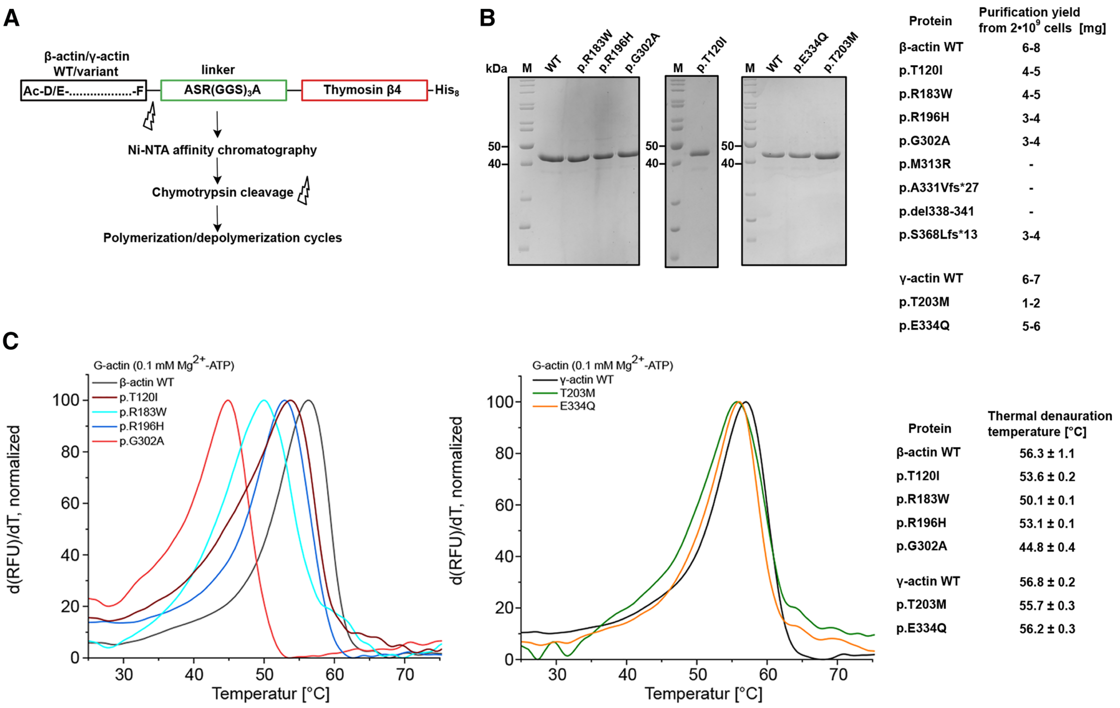

Figure 4. *ACTB* MVs associated with LoF phenotype result in abnormal folding or impact actin thermal stability
(A) Schematic of the purification strategy for recombinant human βCYA and γCYA (adapted from Greve and Manstein[40]).
(B) SDS gels show purified WT and variant actin isoforms with the exact purification yield listed in the right panel; note three recombinant βCYA mutant variants could not be purified. Examples of the immunoblot of Sf9 insect cell lysate are presented in Figure S4.
(C) Differential scanning fluorimetry was used to assess the thermal stability of Mg^{2+}-ATP-G-actin. Representative traces of experiments with βCYA WT and variants (left) and γCYA (right) in the presence of 0.1 μM Mg^{2+}-ATP. Data are shown as the first derivative of the obtained protein melting traces. The point of thermal denaturation (T_M) is determined from the peak of the function. Final concentration of actin is 0.2 mg mL^{-1}. Four independent experiments were performed. Means and SD are shown in the right panel.

investigated the potential impacts of CYA variants on actin dynamics. *ACTB*:T120I (BWCFF1), *ACTB*:R196H (BWCFF1), and *ACTG1*:T203M (BWCFF2) could be successfully purified with the thermal stability similar to the purified control isoforms (Figure 4). We observed a striking decrease in filament polymerization rates and faster depolymerization in the cases of *ACTB*: R196H and *ACTG1*:T203M (Figures 5A and S11). All tested mutants formed co-filaments with WT protein. The presence of 50% WT protein in the polymerization experiments attenuated the observed defect in all but one mutant (Figure 5B). Actin filaments containing both *ACTB*:T120I and WT proteins showed the same polymerization rate as the pure *ACTB*:T120I mutant. In comparison, we observed only minor alterations in the polymerization and depolymerization kinetics of one unNMA-associated variant *ACTG1*:E334Q.[41] Similarly, nearly normal dynamics were demonstrated for the ACTB:R183W, ACTB: E364K,[34] and ACTG1:K118.[42]

Collectively, we observed significant changes in the polymerization and depolymerization dynamics of the selected BWCFF variants, a biochemical property specifically associated with BWCFF and not observed in other NMA phenotypes (Figure 5D).

The amount of βCYA and γCYA in the primary fibroblasts from individuals with BWCFF did not differ from the actin amount in the healthy control cell lines (Figures S7–S10). Interestingly, we observed no significant differences in the transcriptional profiles of the several BWCFF1/2, DDS1, and unNMA fibroblast cell lines (Figures S12 and S13).

In silico prediction tools require caution in clinical applications with novel actin variants

The identification of novel variants in *ACTB* or *ACTG1* classified as (likely) pathogenic according to ACMG criteria still necessitates a thorough evaluation of their clinical association. This can be particularly challenging in individuals with limited clinical data, such as those undergoing prenatal or newborn testing. A clear correlation between the location of variants in the actin molecule and their associated phenotypic outcomes is crucial for predicting the phenotypic outcome of novel variants. Therefore, we mapped all identified disease-associated single amino acid substitutions in βCYA and γCYA onto the corresponding structures to analyze possible correlations between the variant's location within the three-dimensional structure of actin and the phenotype. We observed no clear phenotype-specific accumulation of variants in

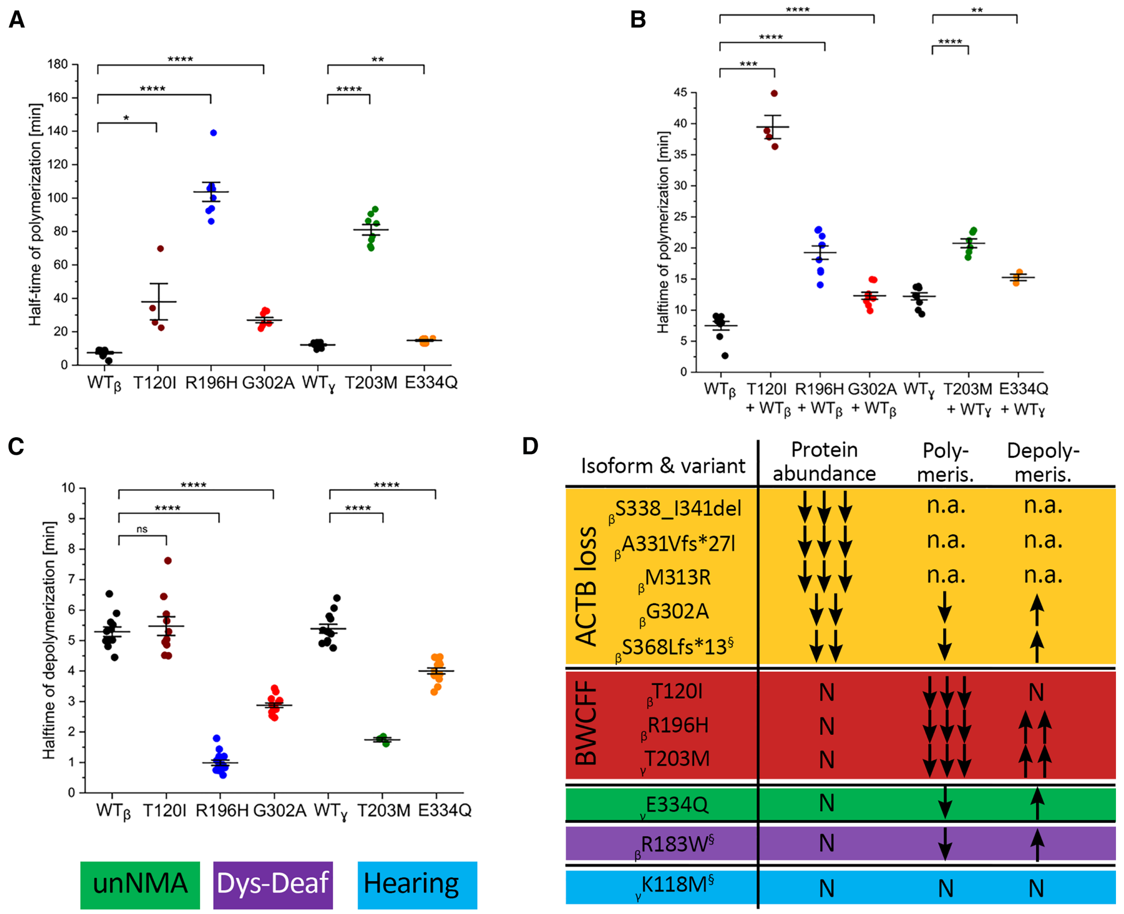

Figure 5. NMA variants result in diverse polymerization anomalies

(A) Salt-induced actin polymerization assessed by pyrene assay. Scatterplot demonstrates distribution of the measured half-times from the individual experiments with 2 μM βCYA WT, γCYA WT, and mutant isoforms. Means and SD are indicated; representative traces are shown in Figure S14.

(B) Same experiment as in (A) repeated with a 1:1 mixture of the WT and mutant proteins.

(C) Dilution-induced actin filament depolymerization assessed by pyrene assay. Scatterplot demonstrates distribution of the measured half-times from the individual experiments with 20 μM F-actin that is rapidly diluted to 0.2 μM to induce depolymerization. Representative traces are shown in Figure S14.

(D) Summary of the actin mutants characterized in the current and previous studies. Previously published mutants are marked with a silcrow with the following references: βS368Lfs*13,[33] βR183W,[34] and γK118M.[42] n.a., not assessable.

specific regions of the actin molecule (Figure S15). Next, we analyzed the location of the variants in correlation with regions located on the filament surface (1), involved in actin-actin interaction (2), interaction with specific actin-binding proteins (profilin [3], cofilin [4], myosin [5], and tropomyosin [6]), phosphate, and Mg^{2+},as well as nucleotide coordination (7) (Figure S15). We found that DD-associated R183W affects one of the key residues of the nucleotide-binding site. Actin-cofilin, actin-profilin, and actin-myosin interaction sites were enriched for PVAs associated with unNMA and HL. However, this is a preliminary observation that is worth further exploration, but it is insufficient to be applied as an ACMG criterion for variant pathogenicity.[36] Multiple *in silico* tools generally classify most rare actin variants as pathogenic; however, they do not allow any allocation to a specific phenotype, emphasizing the importance of the experimental characterization of the mutant proteins.

Discussion

The combination of large datasets, clinical studies, and complementary biochemical studies led to a coherent functional classification of the NMA spectrum. It finally delineated eight distinct NMAs, demonstrating extraordinary pleiotropy and a spectrum of disease severity associated with *ACTB* and *ACTG1* variants. Based on our data, NMAs can be categorized into five clinical entities and three major functional groups (Figure S16). We hypothesize that this remarkable number of conditions associated with the two genes reflects the large number of cellular interactions of CYAs, with downstream effects being context dependent and clinical consequences being highly variant specific. This delineation of disorders has direct implications for the diagnosis and treatment of affected individuals, showing the power of large, systematically collected cohorts in rare diseases.

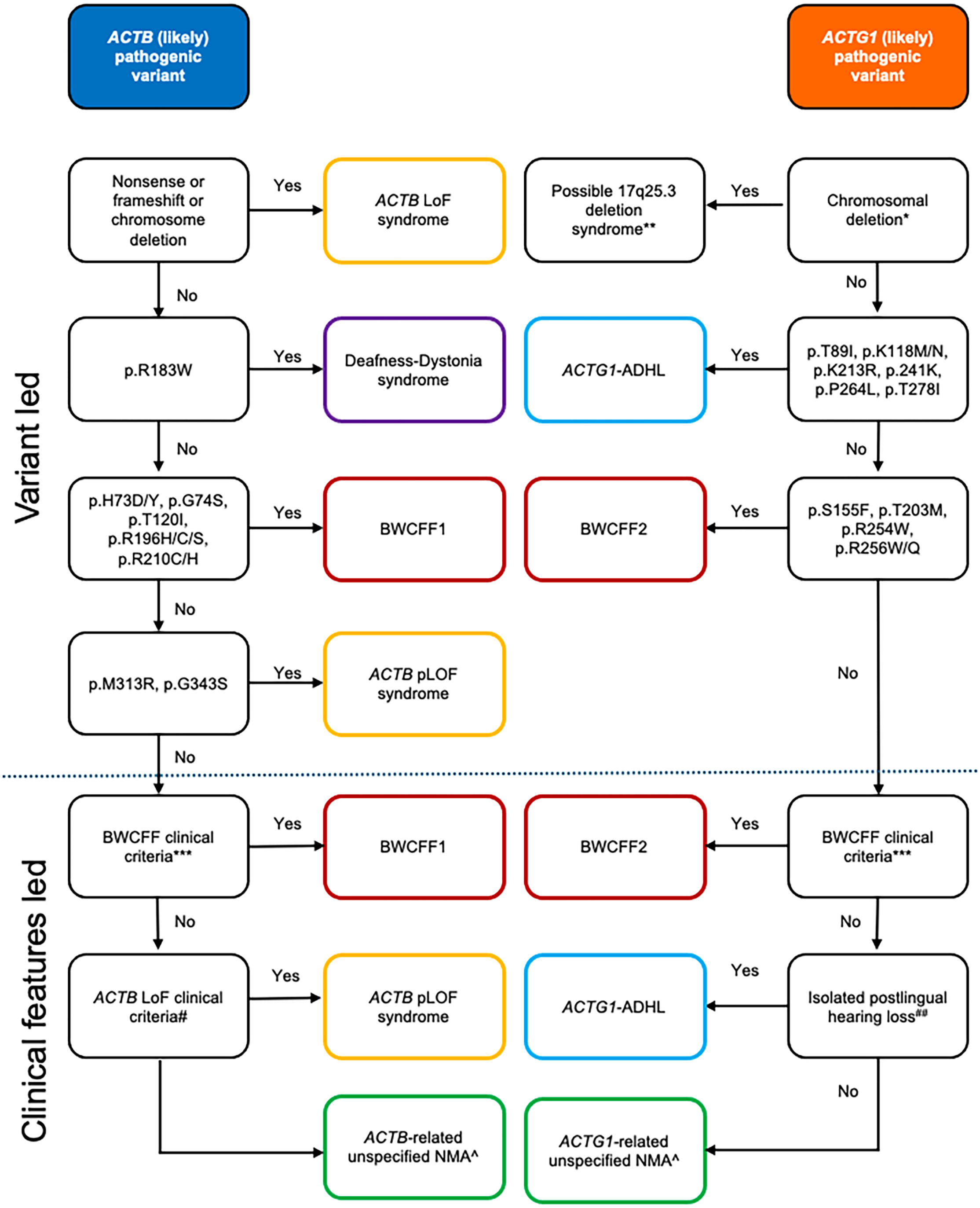

Figure 6. Diagnostic workflow supporting clinical interpretation of ACTB/ACTG1 variants
*ACTG1 nonsense or frameshift variants are likely to be classified as benign by ACMG criteria. **Further studies are required to prove the existence of this disorder; deletions usually involve additional flanking genes and likely have variable penetrance. ***Specific facial dysmorphism (typically including hypertelorism, high-arched eyebrows, ptosis, long palpebral fissures with everted lower lid, broad nasal tip, long smooth philtrum, large mouth with thin upper lip, grooved chin, and large vertically oriented ears) and/or frontal predominant pachygyria with or without other brain and inner organ malformations or minor anomalies. #Specific facial dysmorphism typical for *ACTB* LoF (long face, straight eyebrows, deep-set eyes, epicanthus, narrow or flat nasal bridge with broad nasal tip, and large mouth) without pachygyria; ^Potential revision of the clinical diagnosis during follow-up.

The clinical association of the novel variant in *ACTB* or *ACTG1* might be challenging and requires an accurate assessment of the complete clinical data (expert opinion in Notes S1–S5). Figure 6 shows a diagnostic workflow that guides the clinical classification of individuals with variants in *ACTB* and *ACTG1*. The clinical decision about the type of NMA is heavily based on the evaluation of the facial gestalt and requires previous expertise. Figures 1 and 2 show representative images for ACTB LoF disorder, BWCFF, and the variability of unNMA phenotypes. We

recommend complementing the clinical evaluation with an assessment of the individual's facial gestalt using AI-based phenotyping tools.

While the application of the clinical diagnosis "unspecified NMA" may not fully meet the needs of affected individuals due to its lack of precision, we consider its use valuable for distinguishing this group from other phenotypes. It is essential, however, that individuals and families are informed about the provisional nature of this designation and are offered regular follow-up, as more refined clinical definitions may become available in the future.

Detailed biochemical characterization of disease-associated variants in βCYA and γCYA is currently limited to a few reports.[33,34,41–45] A recent study investigated the substitutions at the position R196 in βCYA, confirming the reduced polymerization rate and faster depolymerization not only in the presence of R196H but also for R196C and R196S variants.[40] Moreover, this work demonstrates a significant reduction of the actin-Arp2/3 interaction, as well as isoform-specific defects in the actin-myosin interaction. As BWCFF-associated variants are scattered throughout the whole length of both actin isoforms, we expect that nearly every variant might have its own additional variant-specific, either more or less pronounced impact on actin-ABP interaction in addition to the perturbed actin dynamics that we suggest being a common molecular mechanism in BWCFF. This might be one of the factors contributing to the broad clinical variability of BWCFF.

In contrast to the striking biochemical abnormalities, we observed no significant changes in either actin abundance or transcriptional profiles of the cell lines derived from affected individuals. Patient-derived skin fibroblasts provide a readily accessible and genetically matched cellular model widely used for functional studies in rare genetic diseases.[46,47] However, this is not a primarily affected tissue in individuals with any type of NMAs and represents a very robust cellular system that might require specific stimuli to unmask the impact of *ACTB* and *ACTG1* variants. Actin has multiple tissue-specific functions, and it is possible that expression profiles in different cell types of the same individuals might be significantly abnormal. These results emphasize that *in vitro* modeling and functional validation of actin variants require a careful choice of the cellular system.

Although both βCYA and γCYA are among the most abundant cytoplasmic proteins, they also function in the nucleus.[48,49] Though not addressed in this work, the dysfunction of the nuclear actin might be an additional disease mechanism at least for a part of the NMA spectrum. What is even more intriguing is that some actin functions might be regulated not at the protein level but at the level of the nucleotide sequence.[50,51] Biallelic knockout of Actb in mice is lethal.[52] In contrast, mice engineered to express γCYA from the Actb gene were viable, fertile, and largely unremarkable, except for degenerative changes in the inner ear and retina during aging.[53,54] Coding sequences of the *ACTB* and *ACTG1* may have independent functional significance, regulating the transcription patterns and translation dynamics of both actin isoforms, which results in different ribosomal densities specific to each actin isoform.[55] Our cohort comprises 259 individuals with variants that alter the coding sequences of *ACTB* or *ACTG1*. Although we could demonstrate the impact of selected PAVs at the protein level, we did not address the potential impact of these and other variants on the translational dynamics of actin mRNA. This aspect, addressed in future studies, might shed light on the nucleotide-dependent functionalities of actin and explore an additional facet in the molecular pathogenesis of NMAs.

Neurodevelopmental and neurological features are the predominant characteristics of many NMAs. This is in line with the known importance of CYAs for neurodevelopment, neural function, and memory.[56,57] Variants in several other genes that control cytoskeletal dynamics, such as *DIAPH1* (MIM: 602121), *RAC1* (MIM: 602048), and *CYFIP* (MIM: 617978), have been shown to result in neurodevelopmental phenotypes similar to those of NMAs. Our findings underscore the growing appreciation of the significance of fine-tuned cytoskeletal control and dynamics in normal development. Furthermore, understanding of actin dynamics in the neurodevelopmental context is of greater importance, as several other monogenic neurodevelopmental disorders are caused by variants in genes encoding actin regulators and interactors.[58–63] The actin-ABP network appears particularly susceptible to variant-specific effects, as several of these genes result in allelic disorders.[64,65] Studies in patient-derived fibroblasts are inherently limited by their inability to capture the organ-specific actin dynamics critical to the pathogenesis of NMA-related NDDs. Neuronal cells derived from patient-specific pluripotent stem cells provide a more physiologically relevant model for studying these mechanisms.[66] Moving forward, such systems should be systematically employed to investigate neuronal function across distinct NMA phenotypes, enabling a deeper understanding of genotype-phenotype correlations and disease-specific pathomechanisms.

In conclusion, the present work improves the diagnosis and management of individuals affected by NMA and raises caution regarding the clinical interpretation of novel coding and non-coding variants in *ACTB* and *ACTG1*. These results also reflect the considerable amount of variant-level collaborative clinical trials and multi-modal mechanistic studies that may be required to realize the promise of precision medicine for rare allelic disorders. While multiplexed assays for variant effect (MAVEs) may provide valuable functional insights, the complexity of NMA phenotypes is unlikely to be resolved by a single experimental approach. Assays measuring β-actin protein stability are particularly useful for identifying likely *ACTB* LoF variants, and polymerization/depolymerization assays have proven informative

for variants causing BWCFF. Other phenotypes, however, remain mechanistically unresolved and will require further molecular characterization to define suitable functional assays. Beyond MAVEs, simple model organisms such as *Caenorhabditis elegans* offer an attractive and scalable system to dissect the molecular mechanisms of actinopathies and to validate variant effects in a physiological context. An initial proof of concept showed that expression of human actin variants associated with severe or lethal phenotypes in individuals results in comparable phenotypic severity in *C. elegans*, underscoring the potential of this model to functionally stratify actinopathies.[67] Together, these complementary strategies highlight the progress toward integrating molecular and clinical data to refine variant interpretation in non-muscle actinopathies.

Data and code availability

Datasets supporting the results of this article are available in the supplemental information or can be requested from the corresponding authors.

Consortia

Members of the NMA clinical consortium are Andrea Accogli, Maria Albers, Fowzan Alkuraya, Neophytos Apeshiotis, Diana Baralle, Carmen Barba, Allan Bayat, Andreas Benneche, Laura Bernardini, Saskia Biskup, Nina Bögershausen, Knut Brockmann, Nicola Brunetti-Pierri, Peter Burfeind, Ruben Cabanillas, Patricia Corriols-Noval, Elke de Boer, Iris de Lange, Charulata Deshpande, Marta Diñeiro, Emily Doherty, Julia Doll, Sofia Douzgou, Tracy Dudding-Byth, Nadja Ehmke, Katherine Fawcett, Carlos R. Ferreira, Jan Fischer, Joel Fluss, Rocío González-Aguado, Luitgard Graul-Neumann, Andrew Green, Renzo Guerrini, Asya Gusina, Ute Hehr, Maja Hempel, Michaela A.H. Hofrichter, Ivan Ivanovski, Diana Johnson, Marieke Joosten, Silke Kaulfub, Tjitske Kleefstra, Eva Klopocki, Karla Krause, Alma Kuechler, Maria Kuzyakova, Martin W. Laass, Augusta Lachmeijer, Wayne Lam, Cha Gon Lee, Yun Li, Vanesa López-González, Karen Low, Michael Lyons, Carlo Marcelis, Francisco Martinez-Castellano, Maarten Massink, Kay Metcalfe, Donatella Milani, Shahida Moosa, Manuela Morleo, Teresa Neuhann, Thomas Neumann, Huu Nguyen, Vincenzo Nigro, Nuha Nimeri, Ewa Obersztyn, Anne O'Donnell, Carmen Orellana, Estrella Pallas, Hans-Jürgen Pander, Elena Parrini, Silke Pauli, Michele Pinelli, Lina Quteineh, Julia Rankin, Monica Rosello, Tamanna Roshan Lal, Vincenzo Salpietro, Jens Schallner, Gregor Schlüter, Julia Schmidt, Mariasavina Severino, Vandana Shashi, Corinna Siegel, Margie Sinnema, Anne Slavotinek, Sarah Smithson, Siddharth Srivastava, Rikke Steensbjerre Møller, Maja Svrakic, Lindsay Swanson, Hannah Thomson, Eduardo Tizzano Ferrari, Annalaura Torella, Irene Valenzuela Palafoll, Yolande van Bever, Ellen van Binsbergen, Marjon van Slegtenhorst, Nienke Verbeek, Virginie Verhoeven, Barbara Vona, Dagmar Wahl, Luisa Weiss, Gökhan Yigit, Maha Zaki, Telethon Undiagnosed Diseases Program, and Undiagnosed Diseases Network.

Acknowledgments

N.D.D., P.D., M.H., and I.N. gratefully acknowledge support of the Core Facility at the NCT/UCC-CMTD Dresden and Stem Cell Engineering Core Facility of the CMCB Technology Platform at TU Dresden. D.J.M. and his lab gratefully acknowledge support provided by the Research Core Unit for Structural Biochemistry. Computing time was provided on supercomputers Lise and Emmy at NHR@ZIB and NHR@Göttingen as part of the Alliance for National High-Performance Computing (NHR) infrastructure. The calculations for this research were conducted with computing resources under the project ID nib00018. N.D.D. received grant support from the Deutsche Forschungsgemeischaft (DI 2170/3-1 and DI 2170/5-1) and Else Kröner-Fresenius-Stiftung (2020_EKES.04). N.D.D. and D.J.M. are supported through the European Union's Horizon 2020 research and innovation program under the EJP RD COFUND-EJP no. 825575 with support from the German Federal Ministry of Education and Research under grant agreements 01GM1922A and 01GM1922B, respectively. D.J.M. acknowledges grant support from the Deutsche Forschungsgemeinschaft (MA1081/28-1). J.N.G. acknowledges the support provided by the PREPARE program for medical scientists from Hannover Medical School. A.S.W. and N.A.R. acknowledge grant support from Medical Research Council project grant MR/T016809/1; Medical Research Council - National Institute for Health and Care Research rare disease research platform MR/Y008340/1; and Kidneys for Life pump priming grant 2017. S.B. acknowledges grant support from the NIHR Manchester Biomedical Research Centre (NIHR203308). S.B., A.S.W., and A.T. acknowledge support from the Davies family for grant support in the form of Marsh Studentship to the University of Manchester. S.B. and S. Cuvertino acknowledge support from Great Ormond Street Hospital Charity research grant V4621.

Author contributions

Conceptualization, N.D.D., D.J.M., A.S.W., and S.B.; methodology, N.D.D., D.J.M., A.S.W., S.B., A.R., G.M., D.P., E.S., J.N.G., P.K., and M.H.; investigation, A.T., J.N.G., J.C., S. Calabro, S. Cathey, B.C., H.C., M.C., S. Cuvertino, P.D., K.E., A.E.F., L.G., S.H., W.G.J., I.K., P.M.-R., A. Meinhardt, I.N., I.R., F.S.S., A. Marquardt, K.T., S.T., J.V., A.V., B.W., T.-C.H., and M.S.; supervision, N.D.D., A.R., E.S., M.H.T., P.K., M.H., C.B.L., N.A.R., D.J.M., A.S.W., and S.B.; writing – original draft, N.D.D., D.J.M., A.S.W., and S.B.; writing – review & editing, all coauthors.

Declaration of interests

The authors declare no competing interests.

Supplemental information

Supplemental information can be found online at https://doi.org/10.1016/j.ajhg.2025.12.007.

Web resources

FastQC, https://www.bioinformatics.babraham.ac.uk/projects/fastqc/
GestaltMatcher database, https://db.gestaltmatcher.org/
GraphPad, http://www.graphpad.com/
OMIM, https://www.omim.org/
PGG.SNV, https://pog.fudan.edu.cn/pggsnv/
Picard tools, http://broadinstitute.github.io/picard/
Trim Galore, http://www.bioinformatics.babraham.ac.uk/projects/trim_galore/

Received: July 17, 2025
Accepted: December 8, 2025
Published: January 12, 2026

References

1. Cerrone, M., Remme, C.A., Tadros, R., Bezzina, C.R., and Delmar, M. (2019). Beyond the One Gene–One Disease Paradigm. Circulation *140*, 595–610. https://doi.org/10.1161/CIRCULATIONAHA.118.035954.
2. Bergeron, S.E., Zhu, M., Thiem, S.M., Friderici, K.H., and Rubenstein, P.A. (2010). Ion-dependent polymerization differences between mammalian beta- and gamma-nonmuscle actin isoforms. J. Biol. Chem. *285*, 16087–16095. https://doi.org/10.1074/jbc.M110.110130.
3. Conboy, E., Vairo, F., Waggoner, D., Ober, C., Das, S., Dhamija, R., Klee, E.W., and Pichurin, P. (2017). Pathogenic Variant in ACTB, p.Arg183Trp, Causes Juvenile-Onset Dystonia, Hearing Loss, and Developmental Delay without Midline Malformation. Case Rep. Genet. *2017*, 9184265. https://doi.org/10.1155/2017/9184265.
4. Latham, S.L., Ehmke, N., Reinke, P.Y.A., Taft, M.H., Eicke, D., Reindl, T., Stenzel, W., Lyons, M.J., Friez, M.J., Lee, J.A., et al. (2018). Variants in exons 5 and 6 of ACTB cause syndromic thrombocytopenia. Nat. Commun. *9*, 4250. https://doi.org/10.1038/s41467-018-06713-0.
5. Riviere, J.B., van Bon, B.W., Hoischen, A., Kholmanskikh, S.S., O'Roak, B.J., Gilissen, C., Gijsen, S., Sullivan, C.T., Christian, S.L., Abdul-Rahman, O.A., et al. (2012). De novo mutations in the actin genes ACTB and ACTG1 cause Baraitser-Winter syndrome. Nat. Genet. *44*, 440–444. https://doi.org/10.1038/ng.1091.
6. Verloes, A., Di Donato, N., Masliah-Planchon, J., Jongmans, M., Abdul-Raman, O.A., Albrecht, B., Allanson, J., Brunner, H., Bertola, D., Chassaing, N., et al. (2015). Baraitser-Winter cerebrofrontofacial syndrome: delineation of the spectrum in 42 cases. Eur. J. Hum. Genet. *23*, 292–301. https://doi.org/10.1038/ejhg.2014.95.
7. van Wijk, E., Krieger, E., Kemperman, M.H., De Leenheer, E.M.R., Huygen, P.L.M., Cremers, C.W.R.J., Cremers, F.P.M., and Kremer, H. (2003). A mutation in the gamma actin 1 (ACTG1) gene causes autosomal dominant hearing loss (DFNA20/26). J. Med. Genet. *40*, 879–884.
8. Zhu, M., Yang, T., Wei, S., DeWan, A.T., Morell, R.J., Elfenbein, J.L., Fisher, R.A., Leal, S.M., Smith, R.J.H., and Friderici, K.H. (2003). Mutations in the gamma-actin gene (ACTG1) are associated with dominant progressive deafness (DFNA20/26). Am. J. Hum. Genet. *73*, 1082–1091. https://doi.org/10.1086/379286.
9. Rainger, J., Williamson, K.A., Soares, D.C., Truch, J., Kurian, D., Gillessen-Kaesbach, G., Seawright, A., Prendergast, J., Halachev, M., Wheeler, A., et al. (2017). A recurrent de novo mutation in ACTG1 causes isolated ocular coloboma. Hum. Mutat. *38*, 942–946. https://doi.org/10.1002/humu.23246.
10. Cuvertino, S., Stuart, H.M., Chandler, K.E., Roberts, N.A., Armstrong, R., Bernardini, L., Bhaskar, S., Callewaert, B., Clayton-Smith, J., Davalillo, C.H., et al. (2017). ACTB Loss-of-Function Mutations Result in a Pleiotropic Developmental Disorder. Am. J. Hum. Genet. *101*, 1021–1033. https://doi.org/10.1016/j.ajhg.2017.11.006.
11. Cai, E.D., Sun, B.K., Chiang, A., Rogers, A., Bernet, L., Cheng, B., Teng, J., Rieger, K.E., and Sarin, K.Y. (2017). Postzygotic Mutations in Beta-Actin Are Associated with Becker's Nevus and Becker's Nevus Syndrome. J. Invest. Dermatol. *137*, 1795–1798. https://doi.org/10.1016/j.jid.2017.03.017.
12. Polubothu, S., Abdin, D., Barysch, M., Thomas, A., Bulstrode, N., Evans, R., Solman, L., Obwegeser, J., Hennekam, R.C., Weibel, L., et al. (2020). Dermatological signs lead to discovery of mosaic ACTB variants in segmental odontomaxillary dysplasia. Br. J. Dermatol. *183*, 1128–1130. https://doi.org/10.1111/bjd.19339.
13. Atzmony, L., Ugwu, N., Zaki, T.D., Antaya, R.J., and Choate, K.A. (2020). Post-zygotic ACTB mutations underlie congenital smooth muscle hamartomas. J. Cutan. Pathol. *47*, 681–685. https://doi.org/10.1111/cup.13683.
14. McLaren, W., Gil, L., Hunt, S.E., Riat, H.S., Ritchie, G.R.S., Thormann, A., Flicek, P., and Cunningham, F. (2016). The Ensembl Variant Effect Predictor. Genome Biol. *17*, 122. https://doi.org/10.1186/s13059-016-0974-4.
15. Karczewski, K.J., Francioli, L.C., Tiao, G., Cummings, B.B., Alföldi, J., Wang, Q., Collins, R.L., Laricchia, K.M., Ganna, A., Birnbaum, D.P., et al. (2020). The mutational constraint spectrum quantified from variation in 141,456 humans. Nature *581*, 434–443. https://doi.org/10.1038/s41586-020-2308-7.
16. Taliun, D., Harris, D.N., Kessler, M.D., Carlson, J., Szpiech, Z.A., Torres, R., Taliun, S.A.G., Corvelo, A., Gogarten, S.M., Kang, H.M., et al. (2021). Sequencing of 53,831 diverse genomes from the NHLBI TOPMed Program. Nature *590*, 290–299. https://doi.org/10.1038/s41586-021-03205-y.
17. Zhang, C., Gao, Y., Ning, Z., Lu, Y., Zhang, X., Liu, J., Xie, B., Xue, Z., Wang, X., Yuan, K., et al. (2019). PGG.SNV: understanding the evolutionary and medical implications of human single nucleotide variations in diverse populations. Genome Biol. *20*, 215. https://doi.org/10.1186/s13059-019-1838-5.
18. Cerami, E., Gao, J., Dogrusoz, U., Gross, B.E., Sumer, S.O., Aksoy, B.A., Jacobsen, A., Byrne, C.J., Heuer, M.L., Larsson, E., et al. (2012). The cBio cancer genomics portal: an open platform for exploring multidimensional cancer genomics data. Cancer Discov. *2*, 401–404. https://doi.org/10.1158/2159-8290.CD-12-0095.
19. Gao, J., Aksoy, B.A., Dogrusoz, U., Dresdner, G., Gross, B., Sumer, S.O., Sun, Y., Jacobsen, A., Sinha, R., Larsson, E., et al. (2013). Integrative analysis of complex cancer genomics and clinical profiles using the cBioPortal. Sci. Signal. *6*, pl1. https://doi.org/10.1126/scisignal.2004088.
20. Landrum, M.J., Lee, J.M., Benson, M., Brown, G.R., Chao, C., Chitipiralla, S., Gu, B., Hart, J., Hoffman, D., Jang, W., et al. (2018). ClinVar: improving access to variant interpretations and supporting evidence. Nucleic Acids Res. *46*, D1062–D1067. https://doi.org/10.1093/nar/gkx1153.
21. Fokkema, I.F.A.C., Taschner, P.E.M., Schaafsma, G.C.P., Celli, J., Laros, J.F.J., and den Dunnen, J.T. (2011). LOVD v.2.0: the

next generation in gene variant databases. Hum. Mutat. *32*, 557–563. https://doi.org/10.1002/humu.21438.
22. Hsieh, T.C., Bar-Haim, A., Moosa, S., Ehmke, N., Gripp, K.W., Pantel, J.T., Danyel, M., Mensah, M.A., Horn, D., Rosnev, S., et al. (2022). GestaltMatcher facilitates rare disease matching using facial phenotype descriptors. Nat. Genet. *54*, 349–357. https://doi.org/10.1038/s41588-021-01010-x.
23. Mak, C.C.Y., Klinkhammer, H., Choufani, S., Reko, N., Christman, A.K., Pisan, E., Chui, M.M.C., Lee, M., Leduc, F., Dempsey, J.C., et al. (2025). Artificial intelligence-driven genotype-epigenotype-phenotype approaches to resolve challenges in syndrome diagnostics. EBioMedicine *115*, 105677. https://doi.org/10.1016/j.ebiom.2025.105677.
24. Lesmann, H., Lyon, G.J., Caro, P., Abdelrazek, I.M., Moosa, S., Pantel, J.T., Hagen, M.T., Rosnev, S., Kamphans, T., Meiswinkel, W., et al. (2023). GestaltMatcher Database - a FAIR database for medical imaging data of rare disorders. Preprint at medRxiv. https://doi.org/10.1101/2023.06.06.23290887.
25. van der Maaten, L., and Hinton, G. (2008). Visualizing Data using t-SNE. J. Mach. Learn. Res. *9*, 2579–2605.
26. Dobin, A., Davis, C.A., Schlesinger, F., Drenkow, J., Zaleski, C., Jha, S., Batut, P., Chaisson, M., and Gingeras, T.R. (2013). STAR: ultrafast universal RNA-seq aligner. Bioinformatics *29*, 15–21. https://doi.org/10.1093/bioinformatics/bts635.
27. Wang, L., Wang, S., and Li, W. (2012). RSeQC: quality control of RNA-seq experiments. Bioinformatics *28*, 2184–2185. https://doi.org/10.1093/bioinformatics/bts356.
28. Liao, Y., Smyth, G.K., and Shi, W. (2014). featureCounts: an efficient general purpose program for assigning sequence reads to genomic features. Bioinformatics *30*, 923–930. https://doi.org/10.1093/bioinformatics/btt656.
29. Ritchie, M.E., Phipson, B., Wu, D., Hu, Y., Law, C.W., Shi, W., and Smyth, G.K. (2015). limma powers differential expression analyses for RNA-sequencing and microarray studies. Nucleic Acids Res. *43*, e47. https://doi.org/10.1093/nar/gkv007.
30. Muller, M., Diensthuber, R.P., Chizhov, I., Claus, P., Heissler, S.M., Preller, M., Taft, M.H., and Manstein, D.J. (2013). Distinct functional interactions between actin isoforms and nonsarcomeric myosins. PLoS One *8*, e70636. https://doi.org/10.1371/journal.pone.0070636.
31. Noguchi, T.Q.P., Kanzaki, N., Ueno, H., Hirose, K., and Uyeda, T.Q.P. (2007). A novel system for expressing toxic actin mutants in Dictyostelium and purification and characterization of a dominant lethal yeast actin mutant. J. Biol. Chem. *282*, 27721–27727. https://doi.org/10.1074/jbc.M703165200.
32. Giese, S., Reindl, T., Reinke, P.Y.A., Zattelman, L., Fedorov, R., Henn, A., Taft, M.H., and Manstein, D.J. (2021). Mechanochemical properties of human myosin-1C are modulated by isoform-specific differences in the N-terminal extension. J. Biol. Chem. *296*, 100128. https://doi.org/10.1074/jbc.RA120.015187.
33. Greve, J.N., Schwäbe, F.V., Pokrant, T., Faix, J., Di Donato, N., Taft, M.H., and Manstein, D.J. (2022). Frameshift mutation S368fs in the gene encoding cytoskeletal beta-actin leads to ACTB-associated syndromic thrombocytopenia by impairing actin dynamics. Eur. J. Cell Biol. *101*, 151216. https://doi.org/10.1016/j.ejcb.2022.151216.
34. Hundt, N., Preller, M., Swolski, O., Ang, A.M., Mannherz, H.G., Manstein, D.J., and Müller, M. (2014). Molecular mechanisms of disease-related human beta-actin mutations p.R183W and p.E364K. FEBS J. *281*, 5279–5291. https://doi.org/10.1111/febs.13068.
35. Lek, M., Karczewski, K.J., Minikel, E.V., Samocha, K.E., Banks, E., Fennell, T., O'Donnell-Luria, A.H., Ware, J.S., Hill, A.J., Cummings, B.B., et al. (2016). Analysis of protein-coding genetic variation in 60,706 humans. Nature *536*, 285–291. https://doi.org/10.1038/nature19057.
36. Richards, S., Aziz, N., Bale, S., Bick, D., Das, S., Gastier-Foster, J., Grody, W.W., Hegde, M., Lyon, E., Spector, E., et al. (2015). Standards and guidelines for the interpretation of sequence variants: a joint consensus recommendation of the American College of Medical Genetics and Genomics and the Association for Molecular Pathology. Genet. Med. *17*, 405–424. https://doi.org/10.1038/gim.2015.30.
37. Zschocke, J., Byers, P.H., and Wilkie, A.O.M. (2023). Mendelian inheritance revisited: dominance and recessiveness in medical genetics. Nat. Rev. Genet. *24*, 442–463. https://doi.org/10.1038/s41576-023-00574-0.
38. Hustinx, A., Hellmann, F., Ö, S., Javanmardi, B., André, E., Krawitz, P., and Hsieh, T.C. (2023). Improving Deep Facial Phenotyping for Ultra-rare Disorder Verification Using Model Ensembles (IEEE), pp. 5007–5017.
39. Jaganathan, K., Kyriazopoulou Panagiotopoulou, S., McRae, J.F., Darbandi, S.F., Knowles, D., Li, Y.I., Kosmicki, J.A., Arbelaez, J., Cui, W., Schwartz, G.B., et al. (2019). Predicting Splicing from Primary Sequence with Deep Learning. Cell *176*, 535–548.e24. https://doi.org/10.1016/j.cell.2018.12.015.
40. Greve, J.N., and Manstein, D.J. (2025). Molecular mechanisms of hotspot variants in cytoskeletal beta-actin associated with Baraitser-Winter syndrome. FEBS J. *292*, 4898–4917. https://doi.org/10.1111/febs.70018.
41. Greve, J.N., Marquardt, A., Heiringhoff, R., Reindl, T., Thiel, C., Di Donato, N., Taft, M.H., and Manstein, D.J. (2023). The non–muscle actinopathy–associated mutation E334Q in cytoskeletal γ–actin perturbs interaction of actin filaments with myosin and ADF/cofilin family proteins. eLife *12*, RP93013. https://doi.org/10.7554/eLife.93013.1.
42. Jepsen, L., Kruth, K.A., Rubenstein, P.A., and Sept, D. (2016). Two Deafness-Causing Actin Mutations (DFNA20/26) Have Allosteric Effects on the Actin Structure. Biophys. J. *111*, 323–332. https://doi.org/10.1016/j.bpj.2016.06.012.
43. Morin, M., Bryan, K.E., Mayo-Merino, F., Goodyear, R., Mencia, A., Modamio-Hoybjor, S., del Castillo, I., Cabalka, J.M., Richardson, G., Moreno, F., et al. (2009). In vivo and in vitro effects of two novel gamma-actin (ACTG1) mutations that cause DFNA20/26 hearing impairment. Hum. Mol. Genet. *18*, 3075–3089. https://doi.org/10.1093/hmg/ddp249.
44. Machida, K., Miyawaki, S., Kanzawa, K., Hakushi, T., Nakai, T., and Imataka, H. (2021). An in Vitro Reconstitution System Defines the Defective Step in the Biogenesis of Mutated beta-Actin Proteins. ACS Synth. Biol. *10*, 3158–3166. https://doi.org/10.1021/acssynbio.1c00432.
45. Kruth, K.A., and Rubenstein, P.A. (2012). Two deafness-causing (DFNA20/26) actin mutations affect Arp2/3-dependent actin regulation. J. Biol. Chem. *287*, 27217–27226. https://doi.org/10.1074/jbc.M112.377283.
46. Hentschel, A., Czech, A., Münchberg, U., Freier, E., Schara-Schmidt, U., Sickmann, A., Reimann, J., and Roos, A. (2021). Protein signature of human skin fibroblasts allows the study of the molecular etiology of rare neurological

diseases. Orphanet J. Rare Dis. *16*, 73. https://doi.org/10.1186/s13023-020-01669-1.

47. Olesen, M.A., Villavicencio-Tejo, F., and Quintanilla, R.A. (2022). The use of fibroblasts as a valuable strategy for studying mitochondrial impairment in neurological disorders. Transl. Neurodegener. *11*, 36. https://doi.org/10.1186/s40035-022-00308-y.
48. Gunasekaran, S., Miyagawa, Y., and Miyamoto, K. (2022). Actin nucleoskeleton in embryonic development and cellular differentiation. Curr. Opin. Cell Biol. *76*, 102100. https://doi.org/10.1016/j.ceb.2022.102100.
49. Virtanen, J.A., and Vartiainen, M.K. (2017). Diverse functions for different forms of nuclear actin. Curr. Opin. Cell Biol. *46*, 33–38. https://doi.org/10.1016/j.ceb.2016.12.004.
50. Vedula, P., Kurosaka, S., Leu, N.A., Wolf, Y.I., Shabalina, S.A., Wang, J., Sterling, S., Dong, D.W., and Kashina, A. (2017). Diverse functions of homologous actin isoforms are defined by their nucleotide, rather than their amino acid sequence. eLife *6*, e31661. https://doi.org/10.7554/eLife.31661.
51. Patrinostro, X., Roy, P., Lindsay, A., Chamberlain, C.M., Sundby, L.J., Starker, C.G., Voytas, D.F., Ervasti, J.M., and Perrin, B.J. (2018). Essential nucleotide- and protein-dependent functions of Actb/beta-actin. Proc. Natl. Acad. Sci. USA *115*, 7973–7978. https://doi.org/10.1073/pnas.1807895115.
52. Bunnell, T.M., Burbach, B.J., Shimizu, Y., and Ervasti, J.M. (2011). beta-Actin specifically controls cell growth, migration, and the G-actin pool. Mol. Biol. Cell *22*, 4047–4058. https://doi.org/10.1091/mbc.E11-06-0582.
53. Sundby, L.J., Southern, W.M., Hawbaker, K.M., Trujillo, J.M., Perrin, B.J., and Ervasti, J.M. (2022). Nucleotide- and Protein-Dependent Functions of Actg1. Mol. Biol. Cell *33*, ar77. https://doi.org/10.1091/mbc.E22-02-0054.
54. Vedula, P., Fina, M.E., Bell, B.A., Nikonov, S.S., Kashina, A., and Dong, D.W. (2023). Beta -actin is essential for structural integrity and physiological function of the retina. Preprint at bioRxiv. https://doi.org/10.1101/2023.03.27.534392.
55. Vedula, P., and Kashina, A. (2018). The makings of the 'actin code': regulation of actin's biological function at the amino acid and nucleotide level. J. Cell Sci. *131*, jcs215509. https://doi.org/10.1242/jcs.215509.
56. Cingolani, L.A., and Goda, Y. (2008). Actin in action: the interplay between the actin cytoskeleton and synaptic efficacy. Nat. Rev. Neurosci. *9*, 344–356. https://doi.org/10.1038/nrn2373.
57. Schneider, F., Metz, I., and Rust, M.B. (2023). Regulation of actin filament assembly and disassembly in growth cone motility and axon guidance. Brain Res. Bull. *192*, 21–35. https://doi.org/10.1016/j.brainresbull.2022.10.019.
58. Reijnders, M.R.F., Ansor, N.M., Kousi, M., Yue, W.W., Tan, P.L., Clarkson, K., Clayton-Smith, J., Corning, K., Jones, J.R., Lam, W.W.K., et al. (2017). RAC1 Missense Mutations in Developmental Disorders with Diverse Phenotypes. Am. J. Hum. Genet. *101*, 466–477. https://doi.org/10.1016/j.ajhg.2017.08.007.
59. Harms, F.L., Kloth, K., Bley, A., Denecke, J., Santer, R., Lessel, D., Hempel, M., and Kutsche, K. (2018). Activating Mutations in PAK1, Encoding p21-Activated Kinase 1, Cause a Neurodevelopmental Disorder. Am. J. Hum. Genet. *103*, 579–591. https://doi.org/10.1016/j.ajhg.2018.09.005.
60. Zweier, M., Begemann, A., McWalter, K., Cho, M.T., Abela, L., Banka, S., Behring, B., Berger, A., Brown, C.W., Carneiro, M., et al. (2019). Spatially clustering de novo variants in CYFIP2, encoding the cytoplasmic FMRP interacting protein 2, cause intellectual disability and seizures. Eur. J. Hum. Genet. *27*, 747–759. https://doi.org/10.1038/s41431-018-0331-z.
61. Barbosa, S., Greville-Heygate, S., Bonnet, M., Godwin, A., Fagotto-Kaufmann, C., Kajava, A.V., Laouteouet, D., Mawby, R., Wai, H.A., Dingemans, A.J.M., et al. (2020). Opposite Modulation of RAC1 by Mutations in TRIO Is Associated with Distinct, Domain-Specific Neurodevelopmental Disorders. Am. J. Hum. Genet. *106*, 338–355. https://doi.org/10.1016/j.ajhg.2020.01.018.
62. Begemann, A., Sticht, H., Begtrup, A., Vitobello, A., Faivre, L., Banka, S., Alhaddad, B., Asadollahi, R., Becker, J., Bierhals, T., et al. (2021). New insights into the clinical and molecular spectrum of the novel CYFIP2-related neurodevelopmental disorder and impairment of the WRC-mediated actin dynamics. Genet. Med. *23*, 543–554. https://doi.org/10.1038/s41436-020-01011-x.
63. Scala, M., Nishikawa, M., Ito, H., Tabata, H., Khan, T., Accogli, A., Davids, L., Ruiz, A., Chiurazzi, P., Cericola, G., et al. (2022). Variant-specific changes in RAC3 function disrupt corticogenesis in neurodevelopmental phenotypes. Brain *145*, 3308–3327. https://doi.org/10.1093/brain/awac106.
64. Martinelli, S., Krumbach, O.H.F., Pantaleoni, F., Coppola, S., Amin, E., Pannone, L., Nouri, K., Farina, L., Dvorsky, R., Lepri, F., et al. (2018). Functional Dysregulation of CDC42 Causes Diverse Developmental Phenotypes. Am. J. Hum. Genet. *102*, 309–320. https://doi.org/10.1016/j.ajhg.2017.12.015.
65. Banka, S., Bennington, A., Baker, M.J., Rijckmans, E., Clemente, G.D., Ansor, N.M., Sito, H., Prasad, P., Anyane-Yeboa, K., Badalato, L., et al. (2022). Activating RAC1 variants in the switch II region cause a developmental syndrome and alter neuronal morphology. Brain *145*, 4232–4245. https://doi.org/10.1093/brain/awac049.
66. Niehaus, I., Wilsch-Bräuninger, M., Mora-Bermúdez, F., Bobic-Rasonja, M., Radosevic, V., Milkovic-Perisa, M., Wimberger, P., Severino, M., Haase, A., Martin, U., et al. (2024). Cerebral organoids expressing mutant actin genes reveal cellular mechanism underlying microcephaly. Preprint at bioRxiv. https://doi.org/10.1101/2022.12.07.519435.
67. Hecquet, T., Arbogast, N., Suhner, D., Goetz, A., Amann, G., Yürekli, S., Marangoni, F., Quintin, S., Greve, J.N., Di Donato, N., and Reymann, A.C. (2025). Multiscale characterization of Caenorhabditis elegans mutants to probe functional mechanisms of human actin pathological variants. iScience *28*, 113652. https://doi.org/10.1016/j.isci.2025.113652.

The American Journal of Human Genetics, Volume *113*

Supplemental information

Molecular genotype-phenotype correlation

in *ACTB*- and *ACTG1*-related non-muscle actinopathies

Nataliya Di Donato, NMA Consortium, Andrew Thom, Andreas Rump, Johannes N. Greve, Juan Cadiñanos, Rocco Salvatore Calabrò, Sara Cathey, Brian Chung, Heidi Cope, Maria Costales, Sara Cuvertino, Philine Dinkel, Kalliopi Erripi, Andrew E. Fry, Livia Garavelli, Sabine Hoffjan, Wibke G. Janzarik, Insa Kreimer, Grazia Mancini, Purificacion Marin-Reina, Andrea Meinhardt, Indra Niehaus, Daniela Pilz, Ivana Ricca, Fernando Santos Simarro, Evelin Schrock, Anja Marquardt, Manuel H. Taft, Kamer Tezcan, Sofia Thunström, Judith Verhagen, Alain Verloes, Bernd Wollnik, Peter Krawitz, Tzung-Chien Hsieh, Michael Seifert, Michael Heide, Catherine B. Lawrence, Neil A. Roberts, Dietmar J. Manstein, Adrian S. Woolf, and Siddharth Banka

Supplemental Materials

Contents

Note S1. Syndrome caused by *ACTB* pLoF variants ... 2
Note S2. Baraitser-Winter-Cerebrofrontofacial Syndrome ... 4
Note S3. *ACTB*:p.Arg183Trp-related dystonia-deafness syndrome ... 8
Note S4. *ACTG1*-associated isolated hearing loss (*ACTG1*-ADHL) ... 10
Note S5. Unspecified non-muscle actinopathies including *ACTG1*-associated isolated coloboma ... 11
Note S6. GestaltMatcher facial analysis ... 15
Note S7. Transcriptome sequencing ... 15
Supplemental Figures ... 17
Figure S1. Classification of the NMA patient cohort applying genomic and phenotypic-led approach. ... 17
Figure S2. Differences in population genetic variability of actin loci. ... 18
Figure S3. Compatible number of cancer-associated somatic variants in CYA genes ... 19
Figure S4. GestaltMatcher analysis of the NMA spectrum. ... 20
Figure S5. Size-matched inter-group separation after downsampling. ... 22
Figure S6. Immunoblot of Sf9 insect cell lysate revealed only small amounts of mutated actin-thymosin β4 fusion constructs in the cell lysate ... 23
Figure S7. Expression of CYA isoforms in patient-derived and control fibroblasts. ... 24
Figure S8. Western blots of ßCYA in patient-derived and control fibroblasts. ... 25
Figure S9. Western blots of γCYA in patient-derived and control fibroblasts. ... 26
Figure S10. Western blots of panactin in patient-derived and control fibroblasts. ... 27
Figure S11. Pyrene–based bulk-polymerization and depolymerization experiments of CYA isoforms (5% pyrene–labeled). ... 28
Figure S12. Expression profiles of the patient-derived and control fibroblasts. ... 29
Figure S13. Principle component analysis of the average expression profile per patient. . 30
Figure S14. MRI images with cortical malformations typical for BWCFF ... 31
Figure S15. Spatial enrichment of variants by phenotype across the actin structure. ... 32
Figure S16. Functional classification of non-muscle actinopathies. ... 34
Supplemental Tables ... 35
Table S4. List of antibodies ... 35
Table S5. GestaltMatcher analysis - positive predictive values for all pairwise contrasts presented in Figure S4. ... 36
NMA clinical consortium ... 37
References ... 45

Supplemental Notes

Note S1. Syndrome caused by *ACTB* pLoF variants

This note includes details about the syndrome cause by heterozygous germline predicted loss of function (pLOF) variants (nonsense and frameshift), and missense variants (MVs) resulting in instability of cytoplasmic β-actin (β-CYA).

0% Absent, <10% Rare, 10-25% Sometimes, 25-75% Frequent, >75% Very frequent 100% Always

Tabular summary of clinical features of individuals with *ACTB* pLOF variants (N=31*)

**Intellectual development and behaviour problems** (Frequent)	21 had intellectual difficulties (ID), mostly borderline/mild and moderate (in two). Where information was available, individuals without ID had either normal or low normal IQ (75-80). Generally, individuals had open and pleasant personalities. Behaviour anomalies reported in 14/31 (45%) individuals (with or without ID), and included attention deficit hyperactivity disorder (ADHD), temper tantrums, autism and difficulty in socialising.
**Craniofacial Anomalies** (Very frequent)	Recognisable facial features with long face, straight eyebrows, deep set eyes, epicanthus, narrow or flat nasal bridge with broad nasal tip, and large mouth. Microcephaly was present in 13.
**Eye coloboma** (Rare)	Iris coloboma and cataract in one individual (116-B).
**MRI anomalies** (Frequent)	Reported in 5/14 (36%) and included periventricular nodular heterotopia (PNVH) in three, hypoplastic corpus callosum (CC) and hypoplastic cerebellar vermis in one, and unspecific areas of high T2 signal in one. Pachygyria was not reported in anyone. MRI was not performed in 17/31.
**Growth problems** (Frequent)	Short stature was documented in 11 (up to -4 SD).
**Epilepsy** (Absent) **or Seizures** (Rare)	No individual was reported to have epilepsy. Seizures were reported in two and included as single episodes of seizures during the early childhood with spontaneous remission later (in individual 168-B with additional variant in *MID2* and individual 107-B).
**Dystonia** (Rare or absent)	Reported in one previously published individual (63-B, patient XXIV[1]) who was lost for follow-up. No neurological features noted in other individuals (included five older than 35y of age).
**Hearing loss** (Rare)	Reported in three and included bilateral sensorineural (173-B, XXVI[1]), bilateral mixed (153-B, XXII[1]), or conductive that resolved after the first few years of life (111-B).
**Skeletal anomalies** (Frequent)	Reported in nine and included pectus deformities, scapula winging, leg deformities, craniosynostosis, congenital parietal foramina, and scoliosis.
**Heart Defects** (Frequent)	Congenital heart defects reported in eight individuals and included atrial septal defect (ASD), ventricular septal defect (VSD), pulmonary stenosis, and patent ductus arteriosus. One individual reported with cardiomyopathy (remission at 10y). One individual reported with left ventricular dilatation at 2y without functional consequences (last assessment at 12y).

**Respiratory problems** (Sometimes)	Reported in five and included severe and prolonged respiratory infections and pneumonias in three and asthma in the remaining two
**Gastro-intestinal problems** (Frequent)	Reported in 12 and included feeding difficulties, failure to thrive in early childhood, constipation, gastro-oesophageal reflux, esophageal atresia with trachea-oesophageal fistula (in one) and gallstones (in one).
**Genito-urinary anomalies** (Sometimes)	Reported in five and included horseshoe kidney in three, hypospadias and renal cortical cysts.
**Skin and integument** (Rare)	Atopic reactions (in three), sparse scalp hair, generalised hirsutism, facial haemangioma, extra skin folds on abdomen and back.
**Repeated infections** (Frequent)	Reported in 8 and included recurrent respiratory infections including pneumonias, and multiple acute otitis media, chronic ear infections. Tests of immune function were performed in one and no abnormality was detected.
**Haematological anomalies** (Sometimes)	Thrombocytopenia was documented in 6 individuals from the original report of the *ACTB*-associated syndromic thrombocytopenia[2], one individual with *ACTB* gene deletion (111-B) and one patient with an *ACTB* MV (61-B). Blood counts were not available for 4/31.
**Healthy carriers** (Rare)	All carries demonstrated typical features that however might have been limited to mild craniofacial dysmorphism and learning difficulties mentioned only on enquiry after the molecular diagnosis

*Some features could not be assessed in all individuals.

Diagnostic and follow-up recommendations

- Individuals with larger deletions can have more severe presentation, perhaps due to loss of other genes.
- 6 out of 31 patients inherited the *ACTB* pLOF variant from a parent who was similarly affected, or mildly affected, or apparently unaffected. pLOF *ACTB* variants should, therefore, be considered as pathogenic variants even if inherited from apparently unaffected parent.
- Clinical follow-up should consider appropriate nutritional status in infancy and early childhood; screening for heart and renal defects; regular hearing test in early childhood in individuals with recurrent otitis; singular blood count with occasional follow-up if necessary (transitory thrombocytopenia with spontaneous remission during first decade was reported); GI function monitoring; developmental and behaviour assessment with appropriate intervention (individuals with severe behaviour anomalies benefit from symptomatic medication).

Note S2. Baraitser-Winter-Cerebrofrontofacial Syndrome

The first report[3] of the syndromic condition later named as Baraitser-Winter syndrome described three children with ID and a unique combination of clinical features including iris coloboma, bilateral ptosis, telecanthus, hypertelorism and short stature, the gestalt resembling Noonan syndrome[4]. The major clinical features were further extended with trigonocephaly and/or prominent metopica suture and lissencephaly mostly in form of frontal predominant pachygyria with or without posterior subcortical band heterotopia[5,6]. Delineation of the genetic cause[7] demonstrated that two other syndromes originally described as separate conditions (Fryns-Aftimos and cerebrofrontofacial syndromes) were part of the same spectrum with a unifying name of the Baraitser-Winter-Cerebrofrontofacial syndrome (BWCFFS) [8,9].

Tabular summary of clinical features in individuals with BWCFFS (N=113)

**Intellectual development and behaviour** (Nearly always)	101/104 individuals whose developmental level could be assessed, presented with developmental delay (DD) (N=25, age 9mo-3y) or ID, that was mild (in 24), moderate (in 33), severe (in 21) and profound in 6 individuals. For 17 individuals ID grade was not specified. Remaining 5 individuals were younger than 9 months; 4 fetal cases were also excluded from this evaluation. One of three individuals without ID had IQ>130. 14 individuals with ID showed behaviour anomalies that included ADHD, hyperactivity, temper tantrums with aggression and autism; however, majority of the individuals had open and pleasant personalities.
**Craniofacial Anomalies** (Nearly always)	Craniofacial anomalies were very consistent resulting in distinct and recognizable facial gestalt. Typical features included prominent metopic ridge, hypertelorism, high-arched eyebrows, ptosis, long palpebral fissures with everted lower lid, broad nasal tip, long smooth philtrum, large mouth with thin upper lip and everted lower lip, grooved chin, large vertically oriented ears, low posterior hair line. Face becomes coarser in the 2nd decade, so gestalt is easier to recognize. Microcephaly was reported in 52% (59/113) individuals with HC in range of -2 to -5.6 SD. Craniosynostosis requiring surgical correction was documented in 5 individuals.
**Eye coloboma** (Frequent)	Iris and/or chorioretinal colobomas were reported in 28. However, vision problems and other eye anomalies were seen in 43 individuals represented by reduced vision, refractive errors and in some individuals microphthalmia/microcornea, nystagmus, cataract and bilateral congenital fibrosis of the rectus medial and inferior extraocular muscles seen in one patient.
**MRI anomalies** (Very frequent)	MRI anomalies were documented in 81/97 (84%) individuals and were represented by lissencephaly/pachygyria in 52. Other cortical malformations included dysgyria/polymicrogyria in five, and periventricular nodular heterotopia (PNVH) in two individuals. Other anomalies included agenesis or hypoplastic CC, leukomalacia, Chiari I anomaly, hypoplastic cerebellum, and ventriculomegaly. MRI was not performed in 16 individuals.
**Growth** (Frequent)	Short stature was documented in 39 (height -6,4 to 3,4 SD) One individual had significantly delayed bone age and received growth hormone therapy with some catch up growth (23-B). Significant failure to thrive in early childhood was reported in at least 6 individuals

**Epilepsy** (Frequent)	Epilepsy was present in 41, with age of onset ranging from neonatal period to 24y. Cortical malformations were reported in 28 individuals with epilepsy, five were reported not to have structural brain anomaly, and in four individuals brain imaging was not performed.
**Dystonia** (Absent)	Not reported.
**Hearing loss** (Frequent)	Reported in 35 individuals and included bilateral sensorineural (in 19), conductive (in two) or unspecified in the remaining individuals.
**Skeletal anomalies** (Frequent)	Reported in 55 and included spine anomalies in 22, pectus anomalies, scapula winging, hip dysplasia, Polydactyly (in seven) and feet deformities.
**Heart Defects** (Frequent)	Reported in 39 and included ASD, VSD, pulmonary stenosis, patent ductus arteriosus, aortic coarctation, and valve anomalies.
**Respiratory problems** (Sometimes)	Reported in 14 and included severe and prolong respiratory infections and pneumonias in six individuals, sleep apnoea and asthma in two individuals as well as laryngomalacia and narrow nasal passage in one patient, respectively.
**GI problems** (Frequent)	Reported documented in 27 and included constipation, feeding difficulties (four required gastrostomy feeding), structural anomalies (in four individuals including duodenal atresia, jejunal atresia, intestinal malrotation and partial bowel obstruction). One individual had progressive liver cirrhosis, and another presented with chronic diarrhoea. Interestingly, GI complains were not documented in the first BWCFF cohort[8] and were often reported only on enquiry and no detailed information was available for 44 of 109 individuals (4 fetal cases excluded).
**Genito-urinary anomalies** (Sometimes)	Structural renal anomalies were reported in 23 individuals and included 10 with duplicated kidneys and/or collecting system, 7 with severe hydronephrosis, and two individuals each with ectopic kidneys, renal fusion or hypoplastic kidneys. Abnormal external genitalia were described predominantly in males including cryptorchidism, inguinal hernia and small penis. Hypoplastic external genitalia were also reported in one female individual.
**Skin and integument** (Sometimes)	Reported in 19 individuals, included dysplastic skin derivatives in 7 individuals (sparse hair, hypoplastic nails, hypodontia, small teeth and delayed tooth eruption), cutis hyperelastica, vascular anomalies (cutis marmorata, teleangiaectasia, hemangiomas) and skin hyperpigmentation (café au lait marks and Mongolian sacral spot), dermatitis, pterygia and interdigital webbing reported in one or two individuals each.
**Repeated infections** (Sometimes)	Repeated and/or excessive infections were documented in 13 individuals mostly as recurrent respiratory infections including pneumonias, multiple acute otitis media as well as chronic ear infections, urinary tract infections and necrotising enterocolitis.
**Haematological anomalies** (Absent)	Not reported (including thrombocytopenia).
**Other**	We could confirm previous observation about BWCFFs typical body posture than becomes apparent in the second or third life decade[8]. This includes anteverted shoulders with generally narrow shoulder gridle, scoliosis and semiflexed knees. Children are often

	present with excessive nuchal skinfolds or pterygium colli, low posterior hairline, pectus excavatum and mild diastasis recti resulting in prominent navel or umbilical hernia.
**Healthy carrier** (Almost absent)	All carries demonstrated typical features and vast majority of the individuals had *de novo* variants. Only one patient inherited the pathogenic variant from affected mother[9], who presented mild but typical gestalt and had low normal intelligence.

*Some features could not be assessed in all individuals.

BWCFF specific brain anomalies

BWCFF is associated with a specific MCD pattern: frontal-predominant pachygyria, frontal pachygyria accompanied with a thin occipital band heterotopia and PVNH (Figure S12). As bilateral PVNH were also observed in individuals with *ACTB* pLOF disorder, we did not consider the later MCD to be BWCFF specific. Enlarged (prominent) perivascular spaces in the centrum semiovale (but not in basal ganglia) were noted in several BWCFF individuals with and without cortical malformations (N=18). As this is a quite common non-specific finding, we suspect that enlargement of perivascular spaces might have been overlooked or not mentioned in the final radiological report in individuals where no MRI images were available for the evaluation.

Prenatal manifestation of BWCFFs

43 of 76 BWCFFs individuals with available pregnancy data had abnormal prenatal history. For the remaining 37 individuals, early clinical information was not available. 3 pregnancies were terminated between 26th and 35th gestational weeks. The most common manifestation was increased nuchal translucency (N=22) either transient or persisting and reaching the form of cystic hygroma (N=7). 14 individuals presented with hydrops fetalis. Other recurrent features were microcephaly, agenesis of the CC, ventriculomegaly or hydrocephalus, polyhydramnios, cleft lip/palate, cortical anomalies, structural renal and heart anomalies. Reduced foetal movements, oligohydramnios and duodenal atresia were reported in a single patient respectively. Although common, prenatal manifestation is not specific and does not allow for clinical suspicion of BWCFF in absence of family history. Prenatal diagnosis is only possible through exome/genome-wide genetic testing. We recommend a very careful consideration of the clinical diagnosis in every patient with a novel MV in *ACTB* and *ACTG1* as well as MVs that were previously observed in less than three individuals and/or MVs with insufficient clinical information.

Adult complications and reduced life expectancy in BWCFFs

Our BWCFFs cohort includes 19 individuals at the age 18-45 years. The oldest known patient was 62y old at the time of the last follow-up (personal experience of Allan Bayat, limited clinical data were available). 16/19 individuals had epilepsy with AO from 2-24y and all 19 individuals showed ID ranging from mild (2 individuals), moderate (N=7) to severe (N=10). 10/19 individuals demonstrated progressive spinal deformity, limited extension of large joints and slow decline in overall motor activity. Two previously reported individuals died at the age of 26y (P1[10]) and 30y (B34[8]) from complications of the acute ileus and progressive feeding difficulties resulting in recurrent respiratory pneumonias. Both individuals carried the same MV p.Thr120Ile in *ACTB*. The whole BWCFFs cohort encompasses two other deceased individuals with presumable cause of death being adverse reaction to codeine administration at the age of 20y[10] and progressive sepsis in a 8m boy with untreated decompensated heart defect[11]. The

current adult cohort might not be representative for milder affected individuals that were currently diagnosed via genotype-first approach.

Diagnostic and follow-up recommendations

- BWCFF diagnostic criteria delineated in this work - (1) specific facial dysmorphism, and/or (2) frontal predominant pachygyria in a patient with (3) (likely) pathogenic MV in *ACTB* or *ACTG1*.
- Clinical follow-up includes initial organ screening (brain MRI, EEG, heart ultrasound, abdominal and renal ultrasound, assess of the nutritional status, ophthalmologic evaluation including fundoscopy, audiologic evaluation, developmental assessment and genetic counseling) and annual surveillance (surveillance might be more frequent depending on the individual situation)[12]

Note S3. *ACTB*:p.Arg183Trp-related dystonia-deafness syndrome

Our cohort included 9 individuals with well documented progressive generalized dystonia; all of them carried an identical pathogenic variant in *ACTB*:p.Arg183Trp. All individuals had a history of the profound prelingual sensorineural hearing loss with significant improvement with the cochlear implants in individuals who received them. Four remaining individuals were ascertained following detection of *ACTB*:p.Arg183Trp and presence of the congenital deafness without BWCFF specific features. Dystonia was a fully penetrant feature in all adults. However, this might represent an ascertainment bias as all adult individuals underwent genetic testing because of dystonia, whereas younger individuals received exome sequencing because of congenital hearing loss. As the cohort remains small, the exact penetrance of dystonia cannot be estimated.

First individuals reported with *ACTB*-DDs were monozygotic twins[13,14] that were subsequently discussed as a part of BWCFF spectrum[8].

It remains currently unknown whether other MV within the same *ACTB* codon would also result in *ACTB*-DDs. In ClinVar we identified one individual with a de novo MV NM_001101.5(*ACTB*):c.547C>G (p.Arg183Gly). This variant was evaluated by a single submitter as a likely pathogenic for *ACTB*-related disorder. No clinical data could be provided after our active enquiry.

Tabular summary of clinical features in individuals with deafness-dystonia syndrome (N=13)

**Intellectual development and behaviour**	Borderline/mild ID was present in 5 out of 13 individuals, however two individuals with normal intelligence had delayed motor and/or speech development. Three individuals demonstrated abnormal behaviour with anxiety and insecurity in two individuals and psychotic episodes with paranoid delusions in another one.
**Craniofacial anomalies**	No specific gestalt has been documented, however 6 individuals had hypertelorism with arched eyebrows and/or mild ptosis. None of the individuals had BWCFF facial gestalt.
**Eye coloboma**	No coloboma was reported, one patient had cataract diagnosed at 3y[13]
**MRI anomalies**	MRI anomalies were present in two individuals and included arterial ischemic stroke at 5y in one subject (26-B) and bilateral symmetrical FLAIR T2 hyperintensity in the basal ganglia[15] in the other patient with manifest dystonia.
**Epilepsy**	Focal seizures with AO at 5y were reported in one patient[16].
**Dystonia**	Dystonia manifested in 9 of 13 individuals, AO varied from 11 till 24 years and the disease progression from focal into severe generalised dystonia, 5 individuals received deep brain stimulation with positive effect, 3 individuals died. Dystonia did not respond to conventional drug treatment such as LDopa, biperiden and clonazepam[16]. Three individuals without dystonia were 7months, 2y and 12y at the last follow-up.
**Hearing loss**	Profound hearing loss was documented in all individuals, 7 individuals received cochlear implant, one as early as 11months.
**Skeletal anomalies**	Skeletal anomalies (scoliosis) were documented in 3 individuals[13,16] and most probably represented secondary complications of progressive uncontrolled dystonia.
**Heart Defects**	Structural heart defects were not documented.
**Respiratory anomalies**	Respiratory features were present in 3 individuals and included aspiration pneumonia, asthma, and impeded breathing during

	cold most probably representing secondary complication of progressive uncontrolled dystonia.
**GI anomalies**	Gastro-intestinal concerns were documented in 5 individuals and included constipations reported in three individuals as well as achalasia reported in monozygotic twins[13].
**Genito-urinary anomalies**	No GU anomalies were documented.
**Skin and integument**	One patient presented with dermatitis.
**Repeated infections**	Repeated and/or excessive infections were not documented
**Thrombocytopenia and other haematological anomalies**	Thrombocytopenia was not observed.
**Healthy carrier**	All carries presented with congenital deafness; as dystonia is an age-related manifestation, its penetrance in the youngest individuals remains unknown.

Diagnostic and follow-up recommendations

- *ACTB*-DDs is diagnosed in individuals with early onset severe hearing loss carrying MV *ACTB*:p.Arg183Trp.
- Hearing loss is severe and rapidly progressive suggesting that early cochlear implants should be considered to maintain adequate language development.
- The incidence of dystonia remains unknown but current data suggests that it may be as high as 100%.
- Regular monitoring of motor and language development with appropriate early intervention program if necessary.
- Early connection to the neurologist specialized in movement disorders is highly recommended to facilitate future treatment.
- Bilateral globus pallidus interna deep brain stimulation currently represents the only treatment option resulting in substantial clinical improvement.
- Supportive therapy such as early initiation of physiotherapy and application of the adaptive aids after onset of dystonia.

Note S4. *ACTG1*-associated isolated hearing loss (*ACTG1*-ADHL)

Non-syndromic hearing loss was the first disorder associated with the cytoplasmic actin genes[17-19]. Heterozygous variants in *ACTG1* were reported segregating in six families with multiple affected individuals including a large Norwegian family with more than 40 affected family members presenting with post lingual progressive sensorineural hearing loss, clinically defined as DFNA20/26[19,20]. The typical characteristics include a bilateral slowly progressive sensorineural hearing loss with the age of onset between the first and the third decades of life. The hearing loss begins at the highest frequencies and steadily progresses into profound deafness across all frequencies. The majority of the affected individuals would demonstrate the sloping configuration audiogram at the early age while hearing threshold remains intact at the lower frequencies. The hearing loss is progressing into deafness by the 6th decade[21]. Tinnitus, vertigo, and other vestibular symptoms were occasionally reported in individuals with the *ACTG1*-associated hearing loss. The recent review summarized 36 *ACTG1* variants reported in individuals with hearing loss. However, several individuals presented with additional symptoms including other malformations and neurodevelopmental disorder[21] indicating that these individuals should be classified as unspecified non-muscle actinopathies. Considering the highly variable symptomatic even within the same family[22], we recommend careful consideration of the clinical assignment in individuals with the novel *ACTG1* variants especially when the molecular diagnosis was done early in life. Several missense variants such as T89I[17], K118M/N[17,23,24], K213R[25], E241K[23,24], T278I[18], and V370A[19] were recurrently observed in large well characterized families with non-syndromic hearing loss and some of these variants were also studied *in-vitro* and *in vivo*[23,26,27]. These variants can be reliably associated with the non-syndromic hearing loss.
The penetrance was reported as complete; however, the age of onset, progression and severity differ greatly even within the same family[19,21].

Tabular summary of clinical features in individuals with non-syndromic hearing loss (N=60)

**Intellectual development and behaviour**	Normal development.
**Craniofacial anomalies**	No specific gestalt has been documented.
**Hearing loss**	Bilateral progressive sensorineural hearing loss with the typical begin at the higher frequencies and characteristic audiogram with the sloping configuration that may maintain even at the advanced stage. The progression rate varies from 1 dB/year to 6 dB/year[24].
**Vestibular symptoms**	Vestibular dysfunction, manifested as some equilibristic instability, was claimed occasionally by some of the elderly, profoundly hearing-impaired individuals but was formally assessed[19]. Tinnitus is occasionally reported.
**Healthy carrier**	Not reported; hearing loss is an age-related phenotype with the variable onset within the same family.

Note S5. Unspecified non-muscle actinopathies including *ACTG1*-associated isolated coloboma

This cohort encompassed individuals with missense variants in either *ACTB* or *ACTG1* whose clinical features did not fit any of the disorders described above. It is possible that future work might define novel distinct entities within this group, one of which could be an *ACTG1*-associated isolated coloboma[28]. unNMA is diagnosed in a patient without BWCCF typical facial gestalt and/or brain malformation with a (likely) pathogenic missense variant in *ACTB* or *ACTG1* (except *ACTB* R183W) presenting with any phenotype other than post-lingual non-syndromic hearing loss.

In line with the previous section, we want to point out the high phenotypic heterogeneity in this group observed even within the same family. Although most of the individuals presented with the neurodevelopmental disorder, the severity of the intellectual impairment is usually mild with good developmental progress under intensive speech and occupational therapy. Speech was usually more severely impaired in comparison with motor skills. Speech delay was more prominent in children with the congenital or early onset hearing loss and remained a significant health issue even after the administration of the adequate hearing aids or cochlear implants.

The available data on adult individuals in unNMA (10 individuals older than 20y) indicates the stable course with no additional neurological or other health issues being developed. However, this statement would need to be confirmed in a larger patient cohort.

Tabular summary of clinical features in individuals with unspecified NMA (N=66)

	ACTB **N=36**	***ACTG1*** **N=30**
**Intellectual development and behaviour**	21 individuals, borderline/mild in 13 and moderate in 6, 7 individuals had normal mental development; 8 individuals presented prenatally or during neonatal period; 10 individuals with and without ID had behaviour anomalies, presented with ADHS, hyperactive and aggressive behaviour and temper tantrums; single individuals were reported to have sleep disorder, Tourette syndrome and auto aggression.	20 out of 30, mild in 7, moderate in 6 but also severe and profound in 3 individuals; 8 individuals had behaviour anomalies with ADHS and temper tantrums, as well as autism with obsessions described in 1 patient.
**Craniofacial anomalies**	Craniofacial anomalies were present in 27 individuals and were mild in the majority of the individuals. Microcephaly was documented in 11 individuals. Interestingly, microcephaly was a consistent feature in three individuals with MV within the codon 152.	Mild craniofacial anomalies were described in 16 individuals presented with an unspecific pattern. However, 4 individuals had ptosis accompanied by epicanthus in 2. Only two individuals presented with microcephaly.
**Eye coloboma**	Iris coloboma was reported in two individuals.	Iris coloboma was reported in three individuals.
**MRI anomalies**	MRI anomalies were documented in 15 individuals but nobody	MRI anomalies were present in 7 individuals, thereof 4

	presented with cortical malformations except one patient with single PVNH. Structural abnormalities included abnormal corpus callosum in 3, enlarged ventricles in 3 and hydrocephalus in 1, posterior fossa anomalies in 2, as well as Chiari I anomaly, multiple calcifications and abnormal white matter signal in one patient, respectively.	individuals had cortical malformations including PMG in 2, dysgyria in 1 and PVNH in 1; the remaining 3 individuals had either agenesis or hypoplastic corpus callosum.
**Epilepsy**	Epilepsy was present in 5 individuals, two of them had abnormal MRI such as Chiari I anomaly and multiple calcifications. Another patient was diagnosed with Doose syndrome.	Epilepsy manifested in 5 individuals, two of them had cortical malformations (PVNH and PMG).
**Dystonia**	Dystonia was not documented.	Dystonia was not documented.
**Hearing loss**	Hearing loss was documented in 6 individuals as bilateral sensorineural in three individuals, mixed in one patient and conductive in another two individuals.	Hearing loss was present in 21 individuals, all individuals had bilateral sensorineural hearing loss with AO from birth/first year till 3rd and 4th decades. However, adult onset was observed only in one multigenerational family with MV p.Ile85Leu. Other individuals had the onset in early childhood.
**Skeletal anomalies**	Skeletal anomalies were documented in 11 and included vertebral anomalies (N=4) as well as pectus deformity, joint hypermobility, feet deformities, brachydactyly and long and slender fingers described in individual individuals. Eight individuals had short stature (till -3,4 z).	Skeletal anomalies were present in 9 individuals, 5 had scoliosis, two had short stature (-4,7 z) and two bilateral feet deformities, respectively.
**Heart Defects**	Structural heart defects were present in 9 individuals and included ASD, VSD, aortic coarctation, and PFO. Two individuals had transposition of the great arteries and one had dextrocardia. Two individuals had mitral valve prolapse.	Heart anomalies were seen in 5 individuals as ASD/VSD, PDA, pulmonary stenosis and right descending aortic arch with aberrant left subclavian artery and diverticle of Kommerell, respectively.
**Respiratory anomalies**	Respiratory features were present in 4 individuals and included severe and prolonged respiratory infections and	One patient had asthma; another patient presented with laryngomalacia and two individuals had documented

	pneumonias in three and respiratory distress in the remaining patient.	tracheomalacia in early months.
**GI anomalies**	Gastro-intestinal concerns were documented in 12 individuals and required operative treatment in 4 individuals.<br>Incomplete data in 6 individuals and not assessed in 4 fetuses.	Gastro-intestinal concerns were documented in 4 individuals and presented as duodenal atresia in 1, intestinal pseudo-obstruction and TNT dependency in 1, and constipations in the other two individuals. In 8 individuals GI data was incomplete.
**Genito-urinary anomalies**	GU anomalies included renal anomalies in 5 (pyelectasis/hydronephrosis, cystic dysplasia, and pyelonephritis) and abnormal genitalia in other 7 individuals.	One patient presented with hydronephrosis, two with inguinal hernias and one with cryptorchidism.
**Skin and integument**	Diverse dermatological concerns were recorded in 5 individuals: skin laxity, mild angiomas, photosensitivity and cutaneous infections with impetigo.	CALFs were documented in a single patient.
**Repeated infections**	Repeated and/or excessive infections were documented in 7 individuals presented as recurrent respiratory infections including pneumonias in and multiple acute otitis media as well as chronic ear infections. However, 3 individuals demonstrated systemic disorder with recurrent abscesses and cutaneous infections (158-B, 62-B and 119-B). One of these individuals had thymus atrophy. One patient had periodic fever.	Repeated and/or excessive infections were documented in three individuals.
**Thrombocytopenia and other haematological anomalies**	Three individuals had thrombocytopenia presented as borderline or mildly diminished platelet count without manifesting bleeding disorder. All 3 individuals had MV in the last exon.	Thrombocytopenia was not documented.
**Healthy carrier**	All carries demonstrated either ID or structural/morphological anomalies.	All carries demonstrated either ID or structural/morphological anomalies.

Prenatal manifestation in unNMA

Abnormal prenatal history was documented in 15 individuals (N=9 with variants in *ACTB* and N=6 in *ACTG1*). Whereas increased nuchal translucency was the most common prenatal feature in individuals with BWCFF, it was reported in only two pregnancies in the unNMA cohort. Other features included ventriculomegaly, heart defects, cleft lip/palate, duodenal atresia, omphalocele, and fetal arrhythmia. Prenatal molecular diagnosis was made in four cases and led to the termination between 16 and 28 GWs. Three of four foetuses presented with ventriculomegaly or hydrocephalus, one had IUGR, transposition of the great arteries, renal cysts and omphalocele. Detailed neuropathological examination of the cerebral was available in two cases and reported normal cortical structure.

Diagnostic and follow-up recommendations

- Considering the high clinical heterogeneity within the unNMA patient cohort and still limited information about the natural history, developing general recommendations regarding the clinical management remains difficult.
- Referral to an early intervention program is strongly recommended for the detailed developmental and behaviour evaluation and intervention.
- Medical surveillance should be focused on individual presentation of the individuals and may include the control of the growth parameters, cardiac evaluation, hearing test, ophthalmological surveillance and other evaluations depending on individual concerns. Young individuals with uncertain clinical classification should have annual follow-up and their families should be informed that clinical diagnosis is ambiguous and so remains developmental and neurological long-term prognosis; families should be offered the maximal BWCFFs-oriented management that can become less intensive or lifted completely if BWCFFs can be prospectively excluded.

Note S6. GestaltMatcher facial analysis

Figure S4 and Table S5 summarize the quantitative evidence for cohort distinctiveness from the pairwise PPV analysis and the intra-group percentile results against the random baseline. The PPV values (probability that two cohorts are truly different within the decision interval, neutral prior) show strong separation between BWCFF and *ACTB* LoF (PPV ≈ 93%), indicating these cohorts are highly distinct. Comparisons against unNMA also support inter-group distinctiveness: *ACTB* LoF vs unNMA (PPV ≈ 76.5%) and BWCFF vs unNMA (PPV ≈ 63.9%) both favor difference. Within the unNMA framework, ACTB_unNMA vs ACTG1_unNMA yields a moderate signal (PPV ≈ 71.7%), suggesting gene-specific separation inside unNMA. By contrast, BWCFF vs BWCFF_unNMA (PPV ≈ 18.8%) and ACTB_BWCFF vs ACTG1_BWCFF (PPV ≈ 8.7%) show limited evidence of distinctiveness, consistent with substantial phenotypic overlap.

To make sample-size dependence explicit, Figure S5 presents a size-matched downsampling analysis. For each pair, both cohorts are repeatedly downsampled to the same size k (from 1 up to the smaller cohort), and the inter-group mean pairwise distance is summarized at each k relative to the threshold c. As expected, variability widens and apparent separation can attenuate at very small k, whereas robust pairings (e.g., BWCFF vs ACTB LoF) remain consistently above c across a broad range of k. This sensitivity analysis complements the PPV/percentile results and clarifies how limited n in recurrent-variant cohorts influences confidence in inter-group differences.

The intra-group percentile analysis (Figure 3D) against the resampled random baseline (random KDE figure) independently supports these conclusions. BWCFF and *ACTB* LoF have unusually low mean within-group distances—approximately the 1.6th and 4.4th percentiles of the random control distribution, respectively—indicating pronounced intra-group cohesion far beyond chance. By comparison, unNMA and its gene-specific subsets (ACTB_unNMA and ACTG1_unNMA) fall around the 16th–22nd percentiles, which does not indicate a recognizable, tight gestalt as a group. Together, the PPV results (between-group) and percentile findings (within-group) provide convergent, quantitative evidence that BWCFF and ACTB LoF are both internally cohesive and externally distinct from other cohorts, whereas unNMA lacks strong intra-group similarity yet can still be distinct from other cohorts in inter-group comparisons.

Note S7. Transcriptome sequencing

Missense variants in CYA genes do not have major impact on overall gene expression.

In line with the overlapping expression profiles, we observed only few differentially expressed genes between individuals derived and control cell cultures. Only one gene (*OLFM1*) was differentially expressed at an FDR-adjusted p-value cutoff of 0.01 in comparison with BWCFF patient cell cultures to control 2 cell cultures, whereas all other disease-specific cell cultures did not show any differentially expressed genes in comparison to control 2 cell cultures (Supplementary Table 3 Differential Gene Expression Analyses). Of note, seven fibroblast cultures of control 2 group were established under identical conditions like most of the patient derived cultures whereas control 1 consisted of three cultures acquired from Coriell. Some more genes were differentially expressed in comparison to control 1 cell cultures, but only for BWCFF vs. control 1 (90 genes) and *ACTB*-BWCFF vs. control 1 (43 genes).
Analysing the expression of the genes encoding for actin isoforms and actin-binding proteins(ABP) (as in Latham et al.[2]) we observed one cluster that mainly contained BWCFF samples (Figure S8) together with another larger cluster with three subclusters

including three control 2 samples in a subcluster, control 1 samples form a subcluster control samples, Dystonia Deafness, non-BWCFF and BWCFF samples that were more wide-spread across the subclusters. Therefore, analysed actin variants have only minor systemic impact even on the expression of ABP-encoding genes.

Supplemental Figures

Figure S1. Classification of the NMA patient cohort applying genomic and phenotypic-led approach.

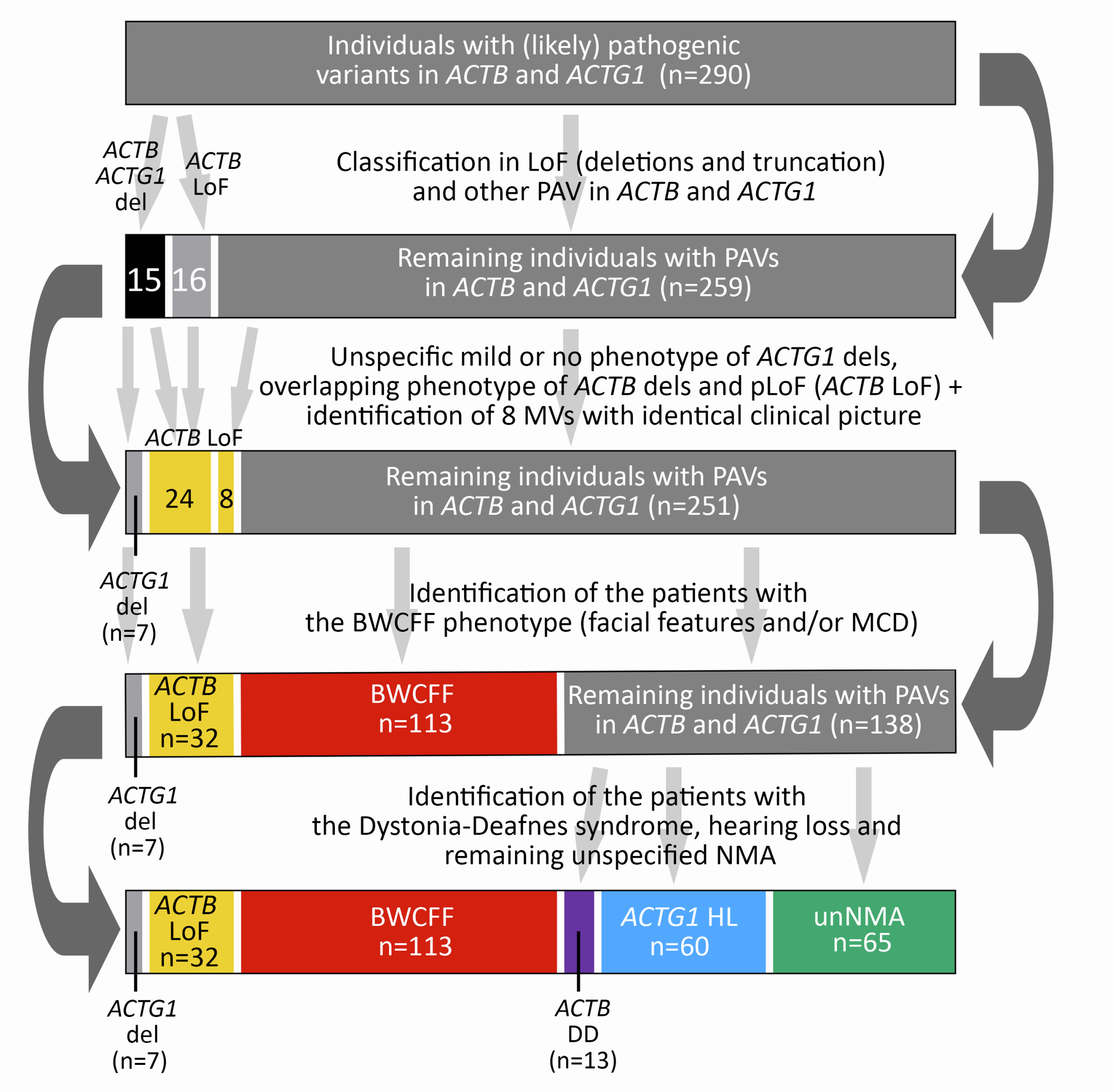

Figure S2. Differences in population genetic variability of actin loci.

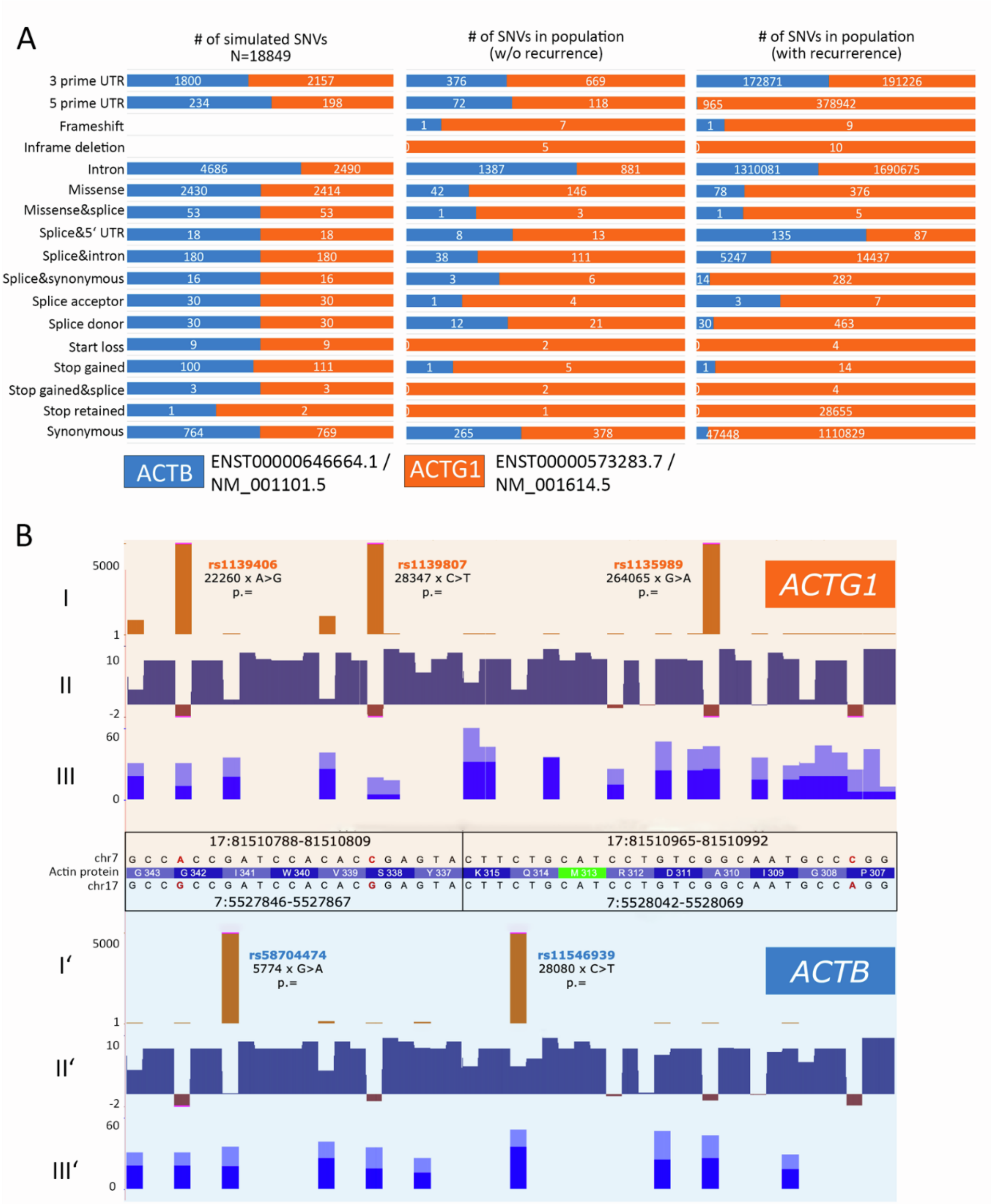

(A) Stacked column charts demonstrating functional consequences of all possible singe nucleotide substitutions simulated within the genomic regions of *ACTB* (NM_001101.5; hg38 7:5527148-5530601) and *ACTG1* (NM_001614.5; hg38 17:81509971-81512799) annotated with the Ensembl Variant Effect predictor (VEP) compared to the small nucleotide variants observed in public databases with (with recurrences) and without (w/o recurrence) population frequencies of the variants; variant types are listed in alphabetical order **(B)** integrated ucsc genome browser view demonstrating two paralogous regions within coding regions of *ACTG1* and *ACTB* (sequence differences are highlighted in red), I and I' relative usage of reference (dark blue) and alternative codon (light blue), II and II' PhyloP basewise conservation score (100 vertebrates), III and III' number of recurrent variants in public databases, pink top on bars indicates the data exceeding the display limit of 5,000.

Figure S3. Compatible number of cancer-associated somatic variants in CYA genes

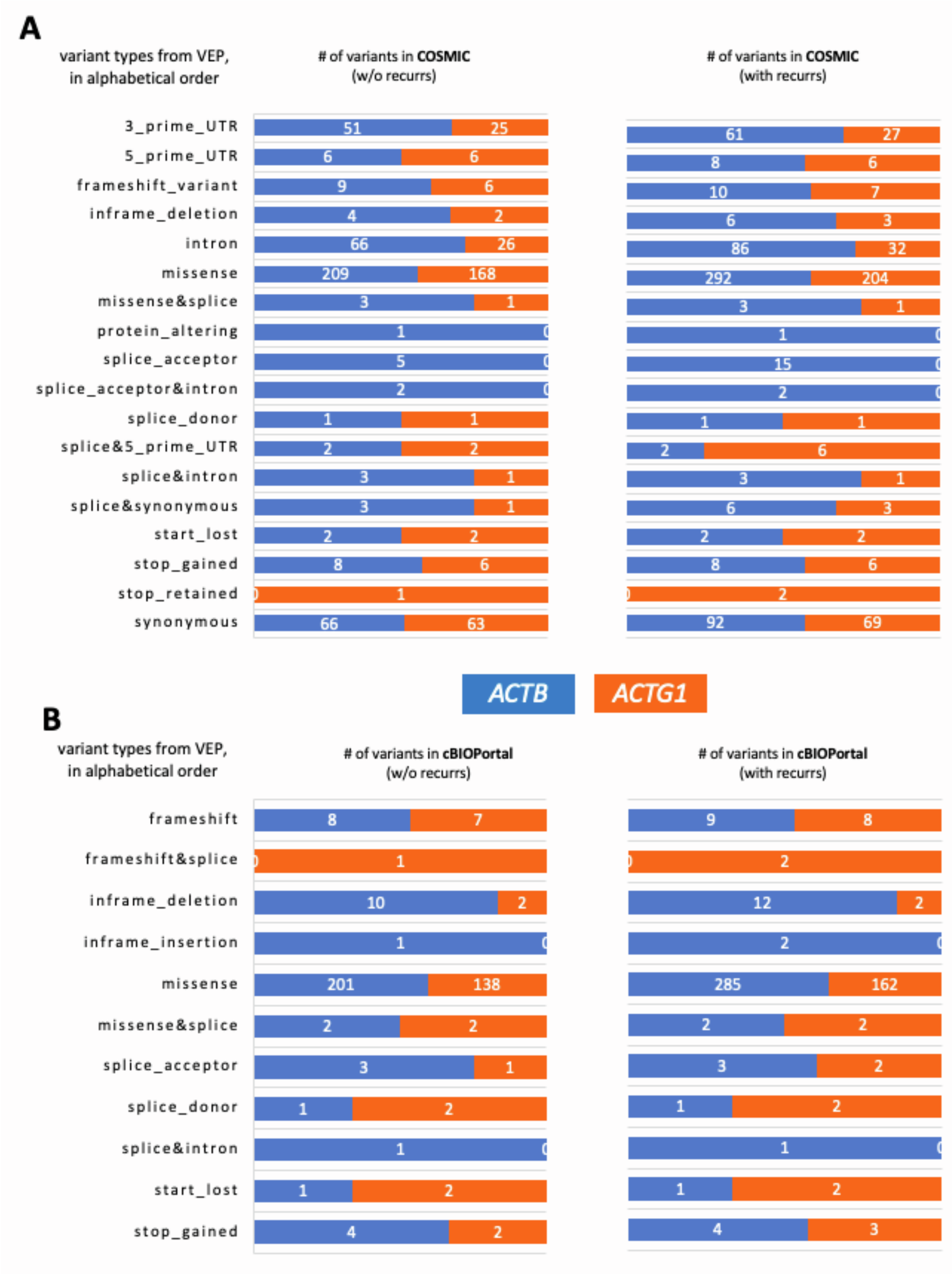

(A) Stacked column charts demonstrating the spectrum and frequencies of somatic small nucleotide variations in *ACTB* and *ACTG1* observed in COSMIC and **(B)** cBIOPortal databases; note that cBIOPortal supports only non-synonymous and coding region small nucleotide variants; colour code and annotations correspond to the Figure S2. Note that the data is included to illustrate the spectrum and relative frequencies of reported somatic variants with respect to the mutagenic potential of the affected regions; no functional or clinical conclusions were drawn.

Figure S4. GestaltMatcher analysis of the NMA spectrum.

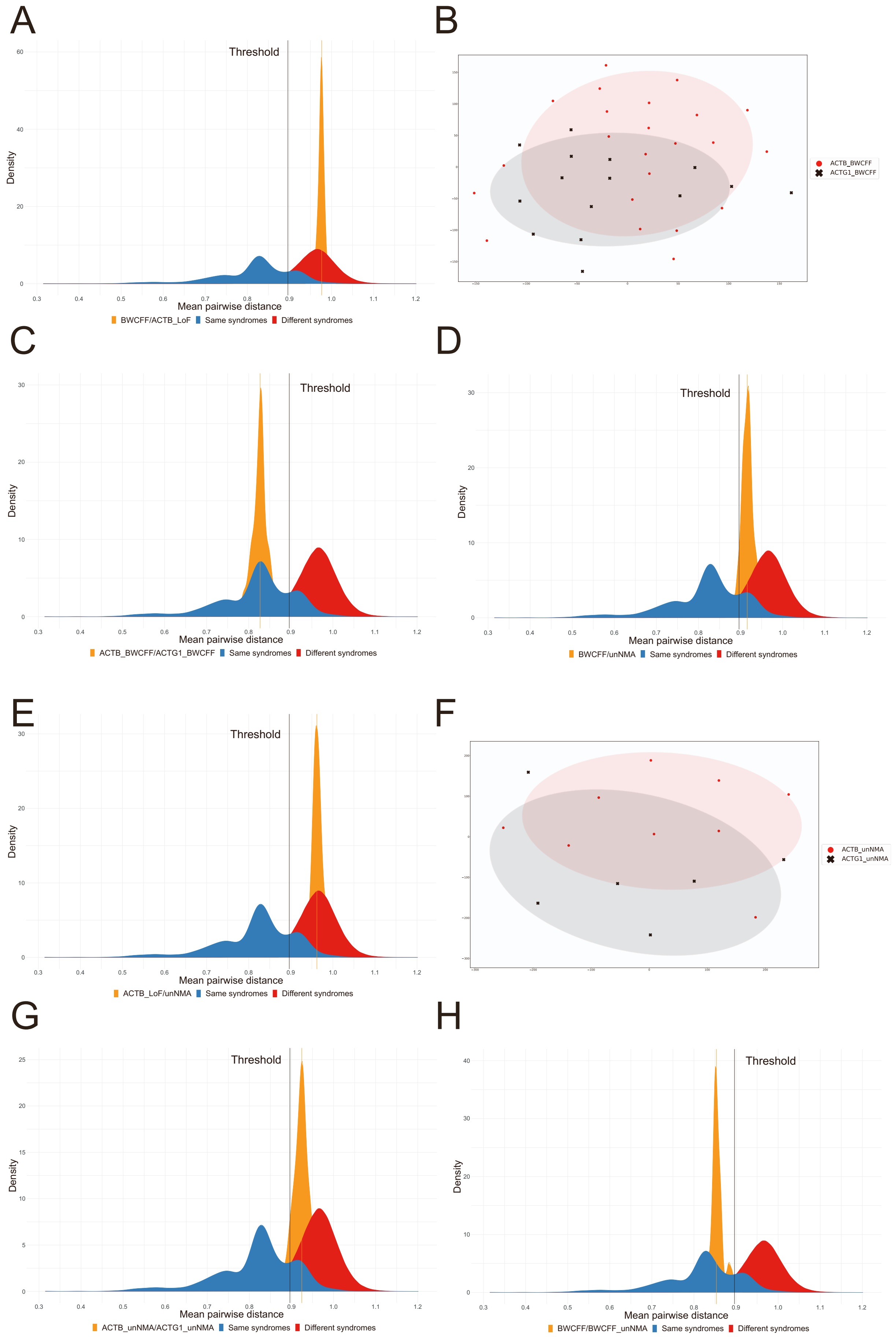

tSNE visualization of different groups in GestaltMatcher analysis and the mean pairwise distance distribution of cohorts sampled from (blue) same syndrome, (red)

different syndrome, and the orange distribution of the target comparisons. The threshold (c) is 0.896. When more than 50% of the orange region fall above the threshold, it indicates the two disorders are not similar. **(A)** The mean pairwise distance between BWCFF and *ACTB*_LoF individuals is 0.977, and 100% of the sampling is above the threshold (the region falling on the right of the threshold) PPV (positive predictive value) ≈ 93%, indicating strong distinctiveness. **(B)** The tSNE visualization between *ACTB*_BWCFF and *ACTG1*_BWCFF. **(C)** The mean pairwise distance between BWCFF_*ACTB* and BWCFF_*ACTG1* individuals is 0.827, and 0% of the sampling is above the threshold. **(D)** The mean pairwise distance between unNMA and BWCFF individuals is 0.916, and 92% of the sampling is above the threshold; PPV ≈ 63.9%, supporting inter-group difference with partial overlap; **(E)** The mean pairwise distance between unNMA and *ACTB*_LoF individuals is 0.962, and 98% of the sampling is above the threshold; PPV ≈ 76.5%, indicating separation. F) The tSNE visualization between *ACTB*_unNMA and *ACTG1*_unNMA. **(G)** The mean pairwise distance between *ACTB*_unNMA and *ACTG1*_unNMA individuals is 0.924, and 92% of the sampling is above the threshold; suggesting gene-specific separation within unNMA. **(H)** The mean pairwise distance between BWCFF and BWCFF_unNMA individuals is 0.853, and 1% of the sampling is above the threshold; PPV ≈ 18.8%, indicating considerable overlap. PPVs for all pairwise contrasts are summarized in Table S5.

Figure S5. Size-matched inter-group separation after downsampling.

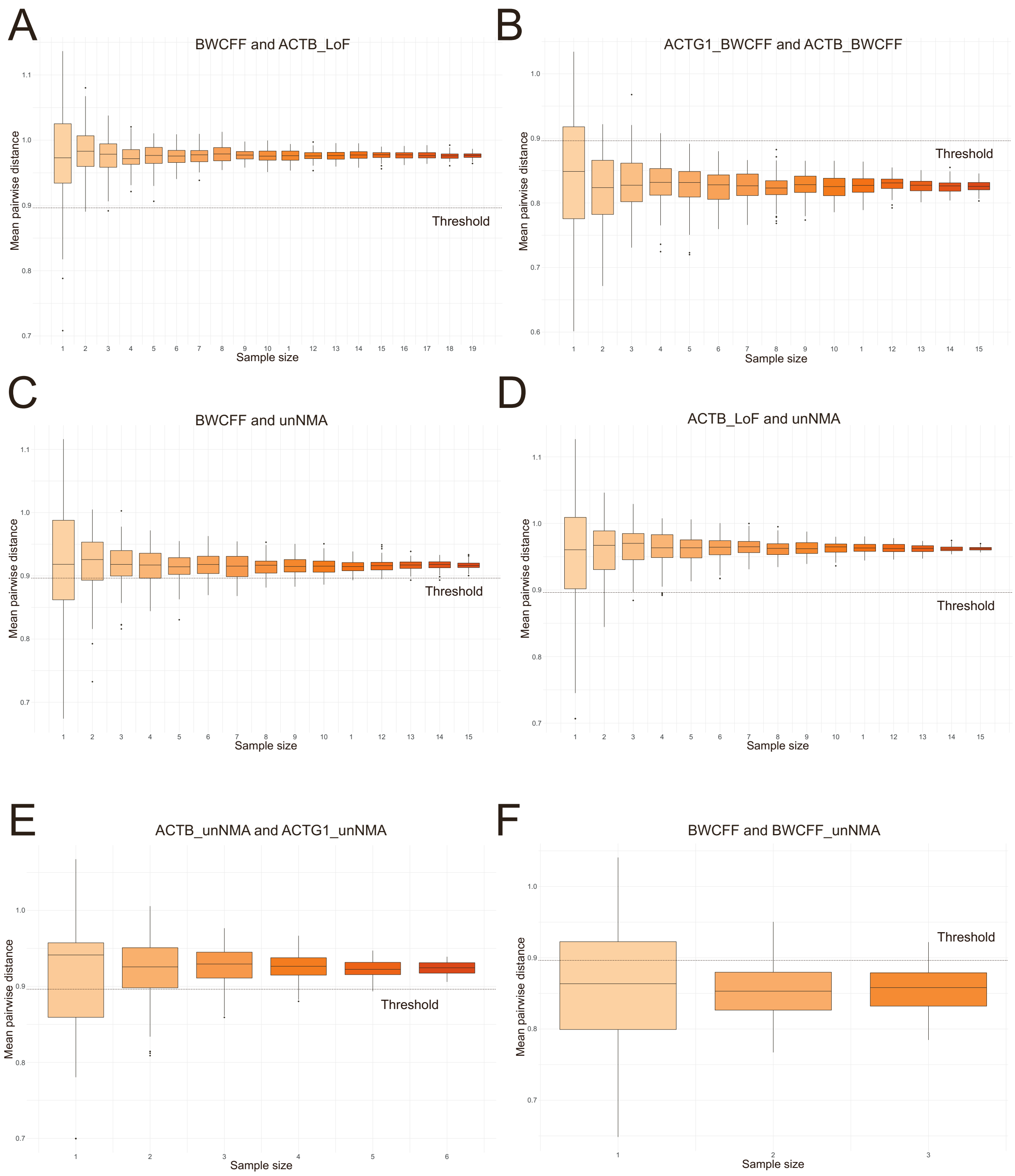

Panels A–F show pairwise cohort comparisons. For each panel, both cohorts are downsampled to the same size k (x-axis) and repeatedly resampled; the boxplots summarize the distribution of the mean pairwise distance between cohorts at that k. The dashed line marks the historical threshold $c$for "different." **A)** BWCFF vs ACTB LoF: distances remain stably above c from small $k$upward. **B)** ACTG1_BWCFF vs ACTB_BWCFF: distances are lower and closer to c, reflecting greater overlap. **C)** BWCFF vs unNMA and **D)** ACTB LoF vs unNMA: distances trend above c as k increases. **E)** ACTB_unNMA vs ACTG1_unNMA: moderate separation with wider dispersion at small k. **F)** BWCFF vs BWCFF_unNMA: small n limits precision; distances hover near c. Overall, smaller k yields wider variability due to limited sampling, while consistently separated pairs remain above c across k, illustrating how recurrent-variant cohorts with small $n$influence confidence in inter-group differences.

Figure S6. Immunoblot of Sf9 insect cell lysate revealed only small amounts of mutated actin-thymosin β4 fusion constructs in the cell lysate

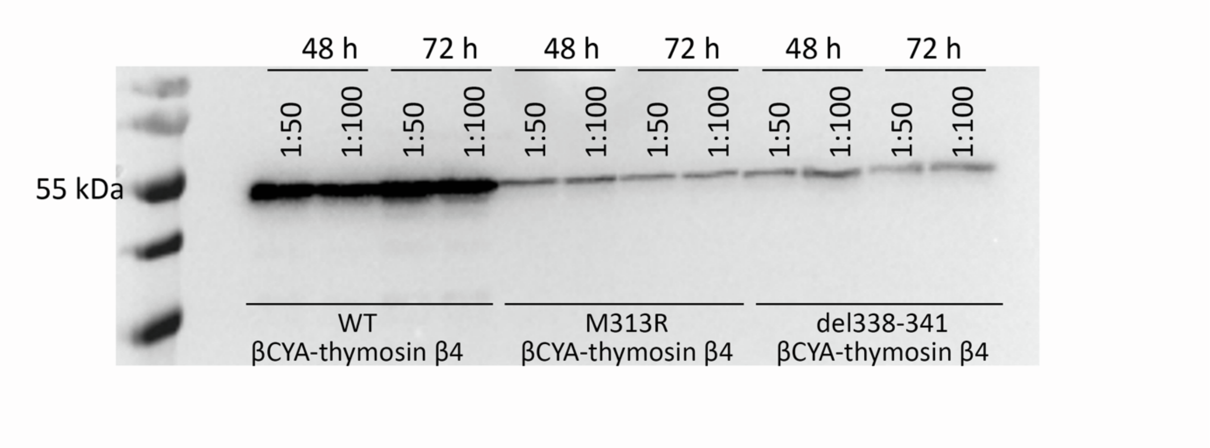

Immunoblot of Sf9 insect cell lysate revealing only small amounts of mutated actin-thymosin β4 fusion constructs in the cell lysate. This amount was not sufficient to purify mutant contracts as they could not be eluted of the NiNTA column. Cells were transfected with different titres of baculovirus encoding for the respective actin-thymosin β4 construct (1:50, 1:100). Samples were taken 48 hours and 72 hours after transfection. Blot was developed using the anti-Penta-His antibody (Qiagen, Hilden, Germany) and the goat anti-mouse IgG-HRP secondary antibody (Thermo, Waltham, USA)

Figure S7. Expression of CYA isoforms in patient-derived and control fibroblasts.

Analysis of bCYA, gCYA, and panactin protein abundance in patient-derived fibroblasts by western blot; data is presented using the box-and-whiskers plot where box contains the 25th to 75th percentiles of the data set and central line indicate the median signal intensity in immunoblots normalized to the total protein.

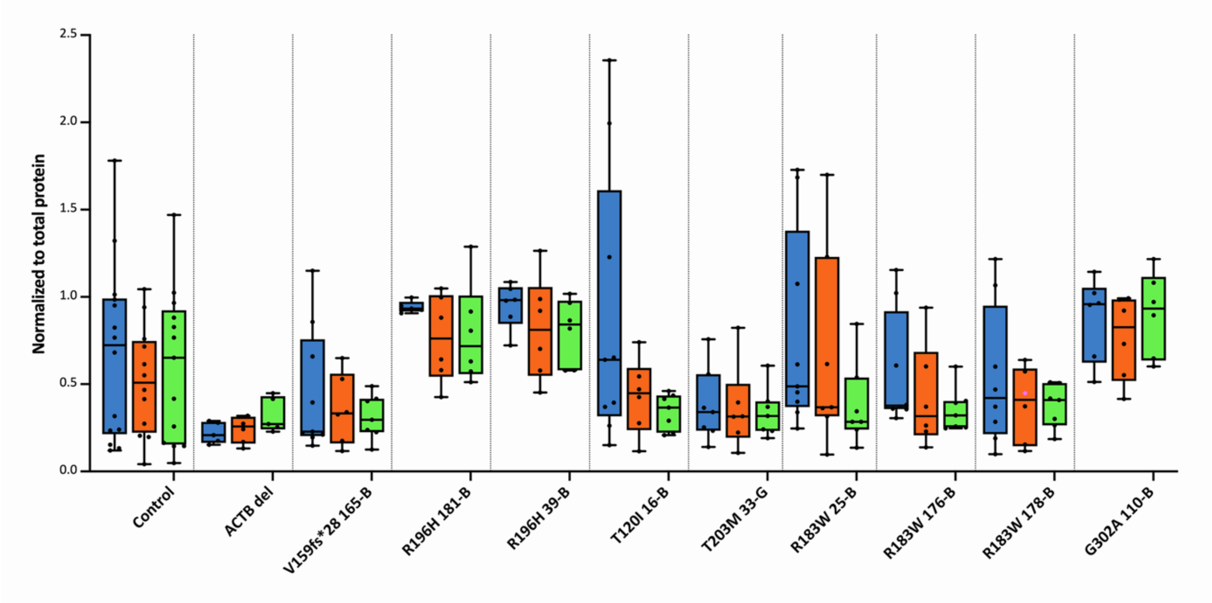

Figure S8. Western blots of ßCYA in patient-derived and control fibroblasts.

(A), (C) Fluorescent total protein membrane staining (Revert^{TM} 700 Total protein stain), gray scale image; samples are labeled corresponding to sample identifiers in Figure S6, R183W corresponds to 25-B and R183W' to 176-B, R196H corresponds to 181-B, R196H' to 39-B (**B), (D)** Fluorescent ßCYA protein detection using IRDye800CW.

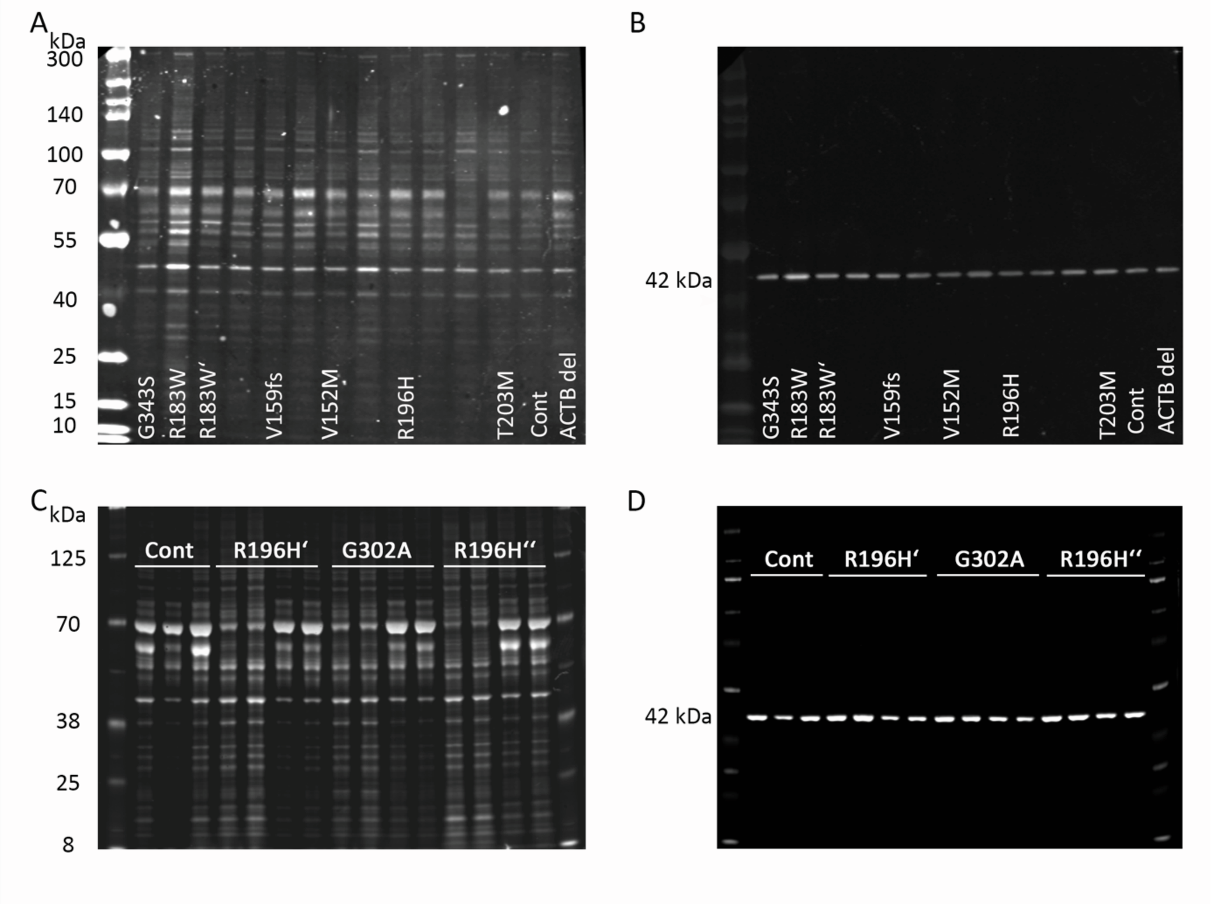

Figure S9. Western blots of γCYA in patient-derived and control fibroblasts.

(A), (C) Fluorescent total protein membrane staining (Revert^{TM} 700 Total protein stain), gray scale image; samples are labeled corresponding to sample identifiers in Figure S6, R183W corresponds to 25-B and R183W' to 176-B, R196H corresponds to 181-B, R196H' to 39-B **(B), (D)** Fluorescent ßCYA protein detection using IRDye800CW.

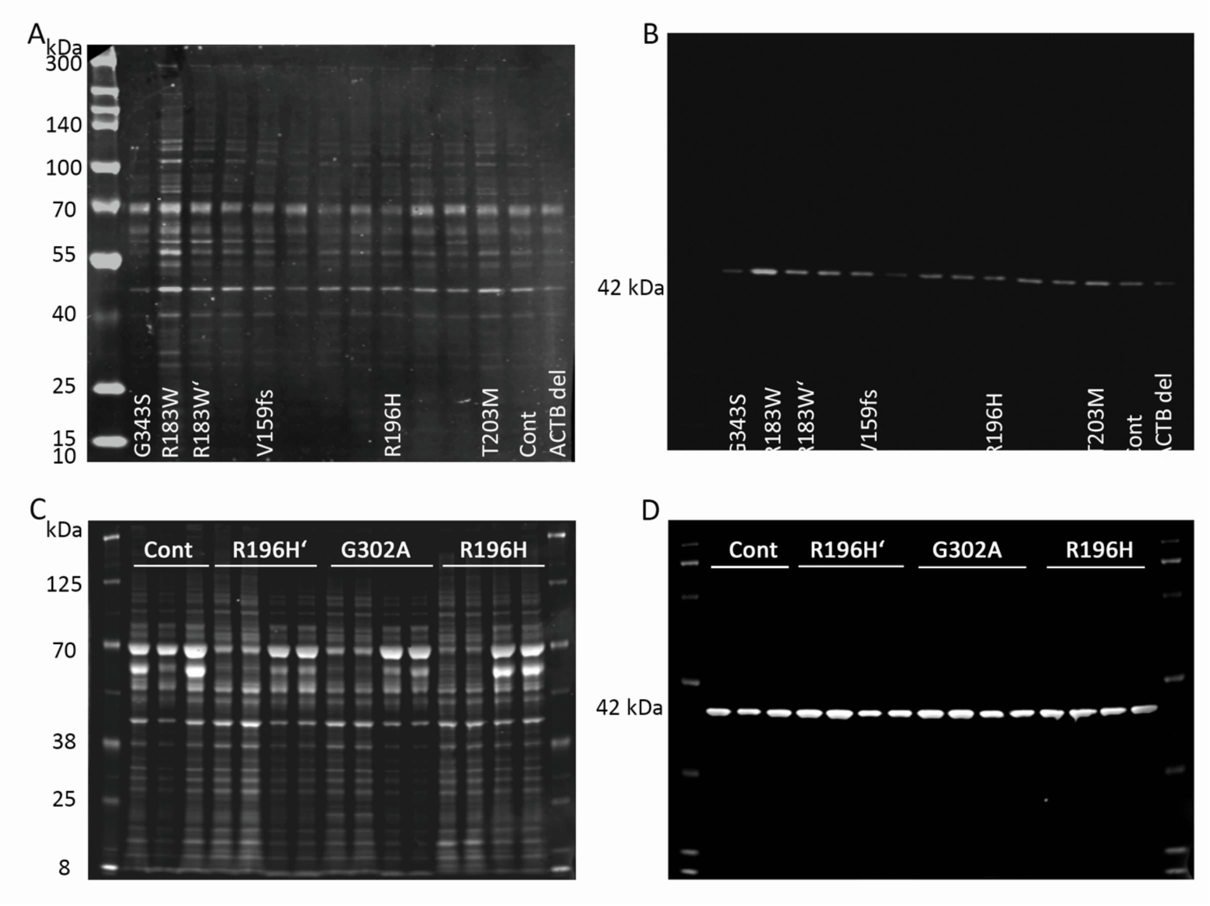

Figure S10. Western blots of panactin in patient-derived and control fibroblasts.

((A), (C) Fluorescent total protein membrane staining (Revert^{TM} 700 Total protein stain), gray scale image; samples are labeled corresponding to sample identifiers in Figure S6, R183W corresponds to 25-B and R183W' to 176-B, R196H corresponds to 181-B, R196H' to 39-B (**B), (D)** Fluorescent ßCYA protein detection using IRDye800CW.

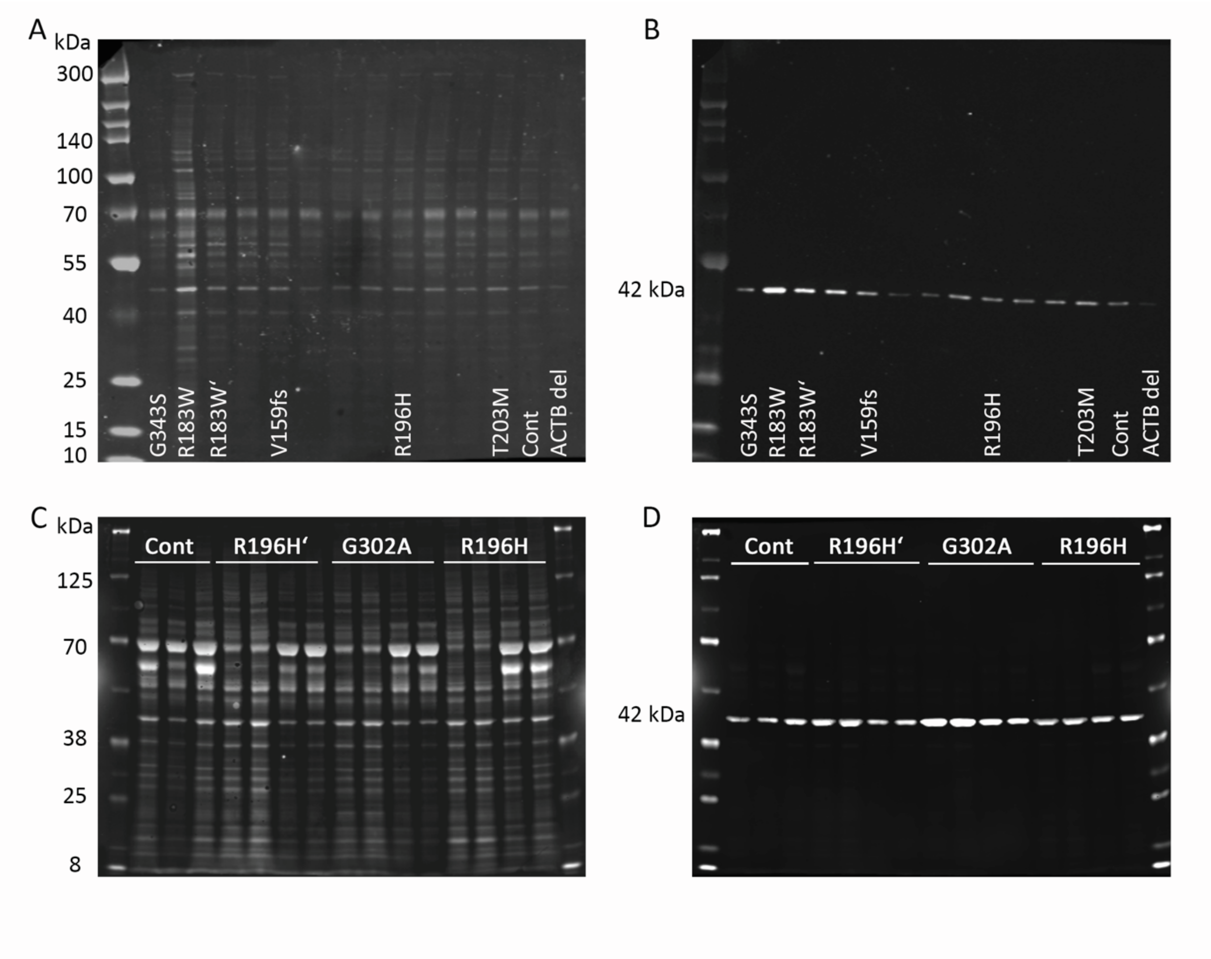

Figure S11. Pyrene–based bulk-polymerization and depolymerization experiments of CYA isoforms (5% pyrene–labeled).

(A, B) Representative traces of pyrene-polymerization experiments with wild type b-actin and mutants. Experiments were performed with pure actin mutants (A) or a 1:1 mixture of wild type and mutant actin (B) **(C)** Representative traces of pyrene-based dilution-induced depolymerization experiments performed with pure wild type b-actin and mutant proteins. (**D, E**) Representative traces of seeded pyrene-polymerization experiments with g-actin wild type and mutants. Experiments were performed with pure actin mutants (D) or a 1:1 mixture of wild type and mutant actin (E). **(F)** Representative traces of pyrene-based dilution-induced depolymerization experiments performed with pure wild type g-actin and mutant proteins.

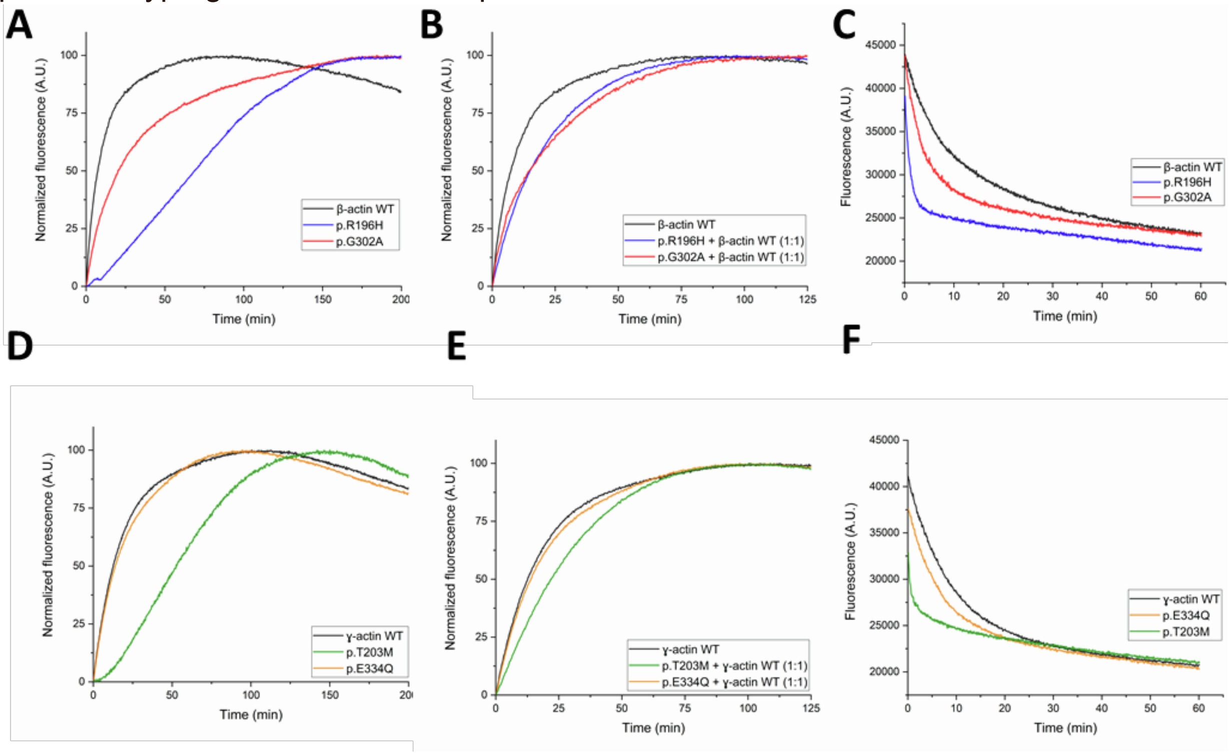

Figure S12. Expression profiles of the patient-derived and control fibroblasts.

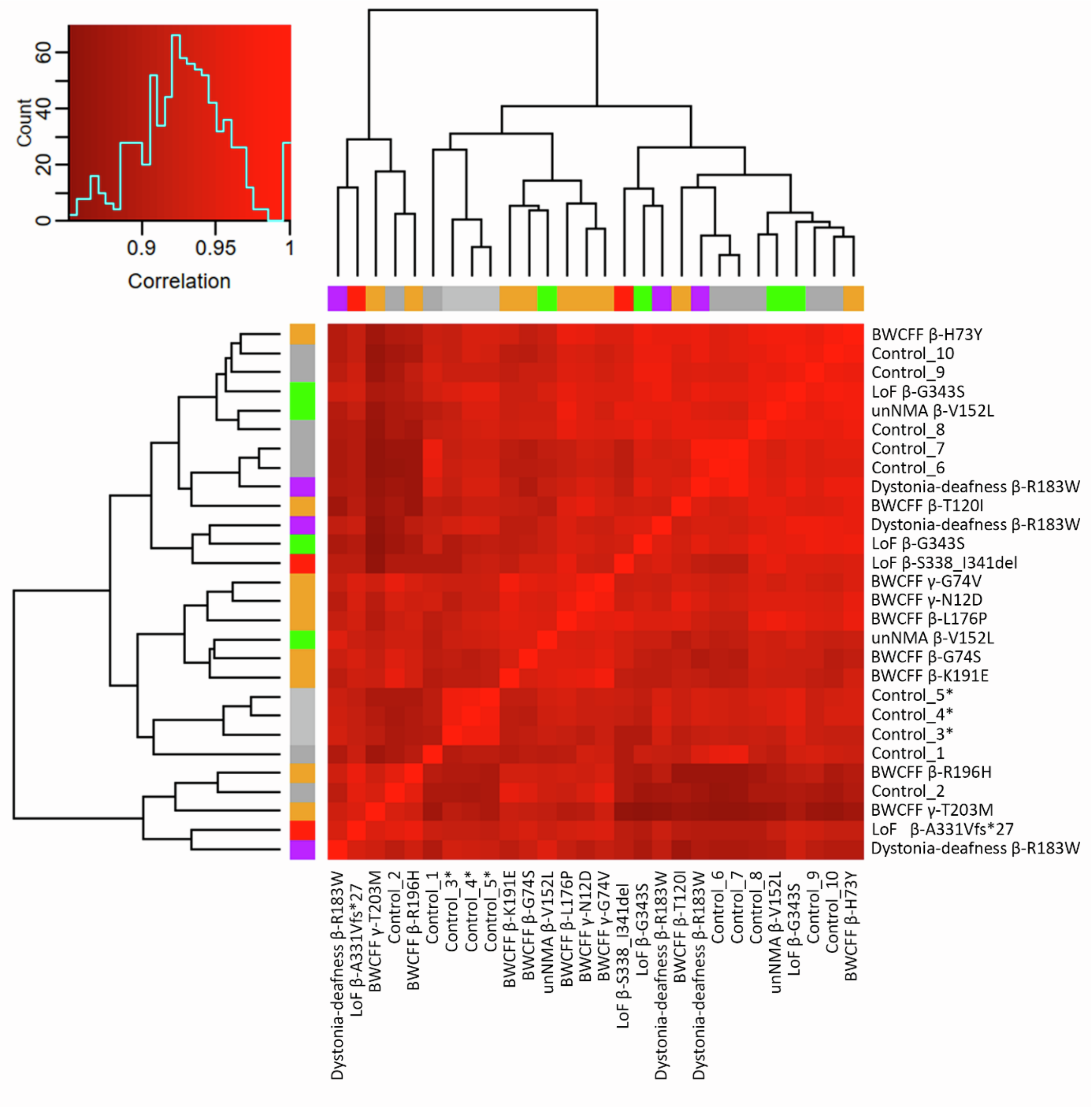

Figure S13. Principle component analysis of the average expression profile per patient.

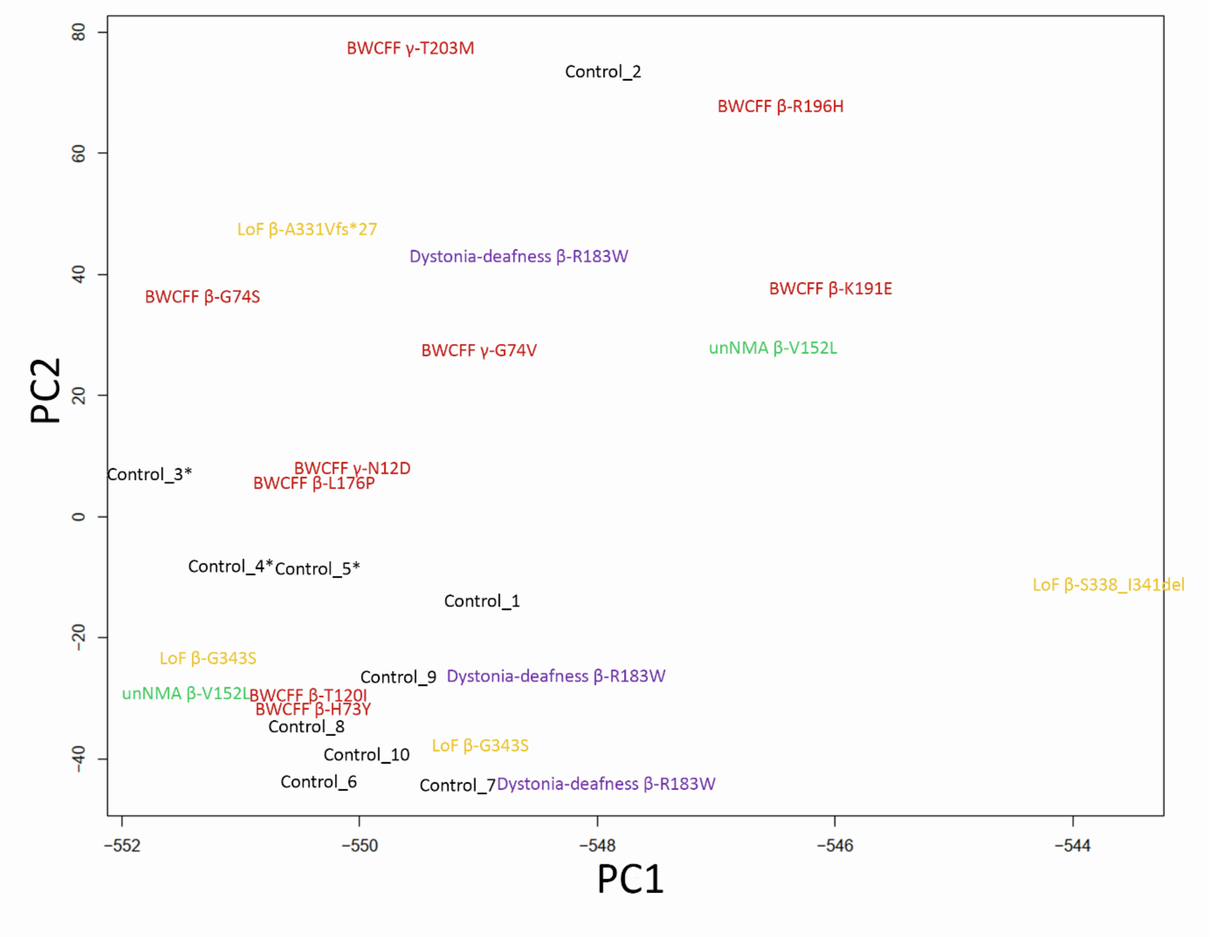

Figure S14. MRI images with cortical malformations typical for BWCFF

(A) T2 weighted axial image demonstrating fronto-temporal pachygyria and prominent perivascular spaces (age 10y); **(B)** T1 weighted axial image shows anterio predominant pachygyria and a thin band heterotopia in the occipital lobes with prominent perivascular spaces (age 1,5y); **(C)** T2 weighted coronal image with the bilateral single periventricular nodules (age 4m); scale bar 1cm.

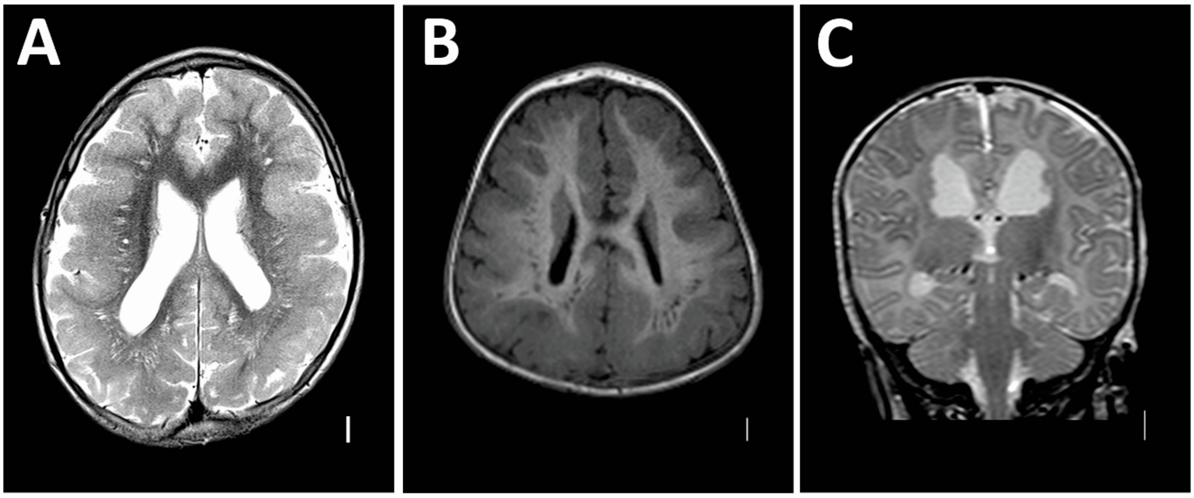

Figure S15. Spatial enrichment of variants by phenotype across the actin structure.

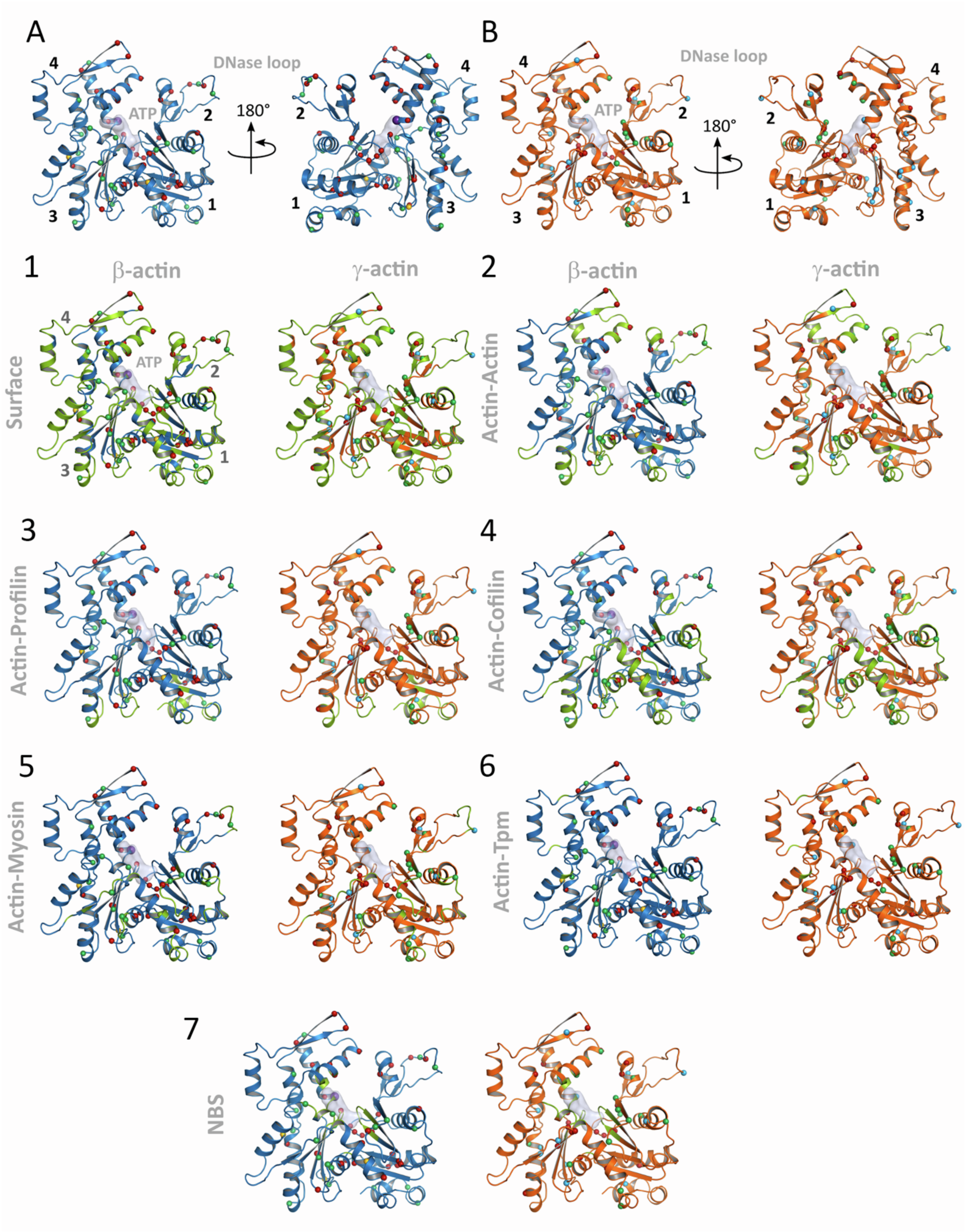

The structures of human βCYA **(A)** γCYA **(B)** were homology modeled using the Schrödinger Prime 4.0 and BioLuminate® applications (Schrödinger Inc., New York, NY). The sequences were retrieved from the Uniprot database (accession numbers: P60709 and P6326). The C-α atoms at mutation sites are shown as spheres. The different disease phenotypes associated with the site of mutation are indicated according to the color code used in Figure 2. Residues located on the surface are colored in

green (1); an interaction site is defined as the region within 5 Å of the binding partner and is colored in green: actin-actin interaction (2) profilin (3) cofilin (4), myosin (5), tropomyosin (6), phosphate and Mg^{2+}, as well as nucleotide coordination at the nucleotide binding site, NBS. (7)

Figure S16. Functional classification of non-muscle actinopathies.

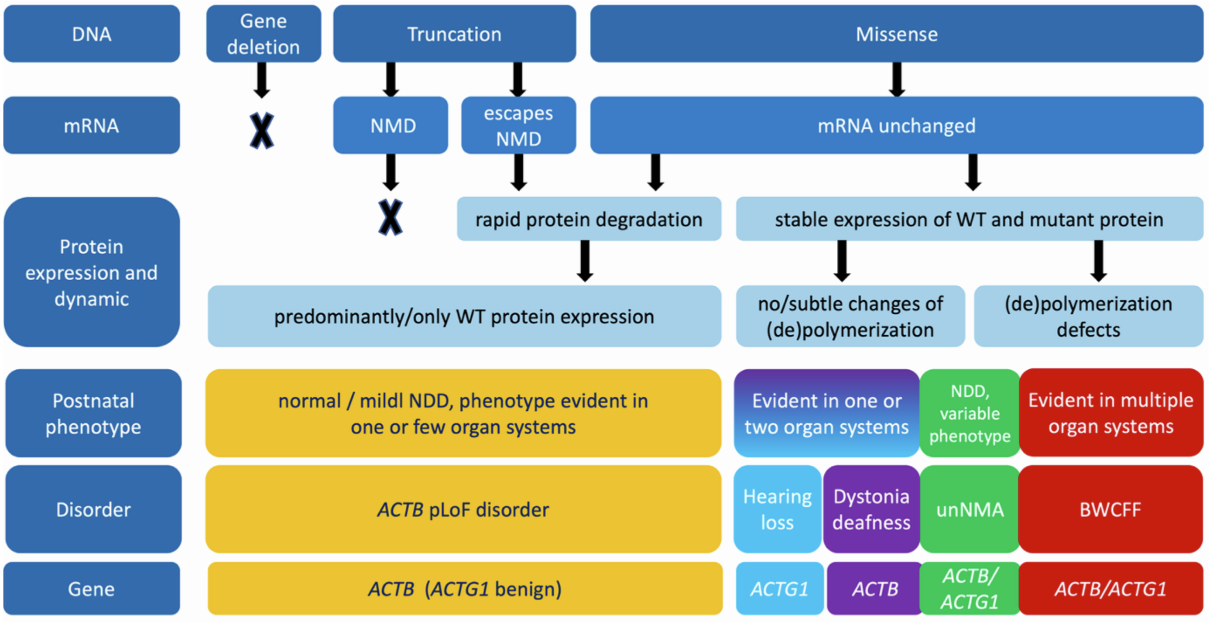

Based on our data, NMAs can be categorized into five clinical entities and three major functional groups First, genomic variants that result in decreased or absent expression of mRNA or production of unstable mutant actin. Such variants in *ACTB* are clinically associated with *ACTB* pLoF. ACTG1 variants of the first group are either benign or result with not-fully penetrant unNMA. Second, MVs that result in stable actin expression with severely impaired poly-/depolymerization dynamics. These variants in both *ACTB* and *ACTG1* are associated with BWCFF, pointing out the correlation with the abnormal postnatal presentation affecting in multiple organ systems. Third, MVs that result in stable expression of actin with normal or slightly abnormal polymerization dynamics. One of these variants in *ACTB* (R183W) leads to dystonia-deafness syndrome, and the others are associated with unNMA. Such variants in *ACTG1* can also cause unNMA. Still, several of these MVs result in progressive hearing loss, suggesting that these group three variants are likely associated with the limited expression of the phenotype referable to only one organ system.

Supplemental Tables

Table S4. List of antibodies

Primary antibodies

Target	Specificity	Company	Catalog no. #	RRID	Dilution factor
β-CYA	Mouse monoclonal IgG_1 clone 4C2	bio-rad	CMCA5775GA	AB_2571580	1:50 (IF)<br>1:7500 (WB)
g-CYA	Mouse monoclonal IgG_{2b} clone 2A3	bio-rad	MCA5776GA	AB_2571583	1:100 (IF)<br>1:7500 (WB)
Ki-67	Rabbit polyclonal	abcam	ab15580	AB_443209	1:300 (IF)
Tuj1	Mouse monoclonal	BioLegend	#801201	AB_2313773	1:300 (IF)
Pan-Actin	Mouse monoclonal	Novus Biologicals	NB600-535	AB_2222881	1:200 (IF)<br>1:2500 (WB)
SOX2	Goat polyclonal	R+D Systems	AF2018	AB_355110	1:300 (IF)
DAPI		Roche	10236276001		1:1000
IRDye® 800CW		Li-COR	926-32210		1:15000

Secondary antibodies

Host/ Target	Isotype	Conjugate	Company	Catalog no. #	RRID	Dilution factor
Goat anti Mouse	Mouse IgG, Fcg Subclass 1 Specific	AlexaFluor 488	Jackson Immunoresearch	115-545-205	AB_2338854	1:200
Goat anti Mouse	Mouse IgG, Fcg Subclass 2b Specific	CY5	Jackson Immunoresearch	115-175-207	AB_2338717	1:50
Donkey anti Mouse	Donkey IgG	AlexaFluor 488	Thermo Fisher	A-21202	AB_141607	1:500
Donkey anti Rabbit	Donkey IgG	AlexaFluor 555	Thermo Fisher	A-31572	AB_162543	1:500
Donkey anti Goat	Donkey IgG	AlexaFluor 647	Thermo Fisher	A-21447	AB_2535864	1:500
Donkey anti Rat	Donkey IgG	AlexaFluor 488	Thermo Fisher	A-21208	AB_2535794	1:500

Table S5. GestaltMatcher analysis - positive predictive values for all pairwise contrasts presented in Figure S4.

Group 1	Group 2	Mean pairwise distance	% above threshold	PPV in interval (%)
BWCFF	ACTB_LoF	0.977	100	92.99
ACTB_BWCFF	ACTG1_BWCFF	0.827	0	8.74
ACTB_unNMA	ACTG1_unNMA	0.924	92	71.68
BWCFF	BWCFF_unNMA	0.853	1.01	18.77
BWCFF	unNMA	0.916	92	63.91

NMA clinical consortium

Name	Affiliation 1	Affiliation 2
Andrea Accogli	Department of Specialized Medicine, Division of Medical Genetics, McGill University Health Centre, Montreal, Canada	Department of Human Genetics, McGill University, Montreal, Canada
Maria Albers	Department of Genetics, University Medical Center Utrecht, Utrecht, Netherlands	
Fowzan Alkuraya	Department of Genetics, King Faisal Specialist Hospital and Research Center, Riyadh, Saudi Arabia	
Neophytos Apeshiotis	Praxis für Genetik, Eckert-Str. 12, Braunschweig, Germany	
Diana Baralle	Faculty of Medicine, University of Southampton, University of Southampton, Southampton, United Kingdom	
Carmen Barba	Neuroscience Department, Meyer Children's Hospital IRCCS, viale Pieraccini 24, 50139, Florence, Italy	Department of NEUROFARBA, University of Florence, viale Pieraccini 6, 50139, Florence, Italy
Allan Bayat	Department of Epilepsy Genetics and Personalized Medicine, Danish Epilepsy Centre, Dianalund, Denmark	Department of Clinical Genetics, Copenhagen University Hospital, Rigshospitalet, Copenhagen, Denmark
Andreas Benneche	Department of Medical Genetics, Haukeland University Hospital, Bergen, Norway	
Laura Bernardini	Medical Genetics Unit, IRCCS Casa Sollievo della Sofferenza Foundation, San Giovanni Rotondo (FG), Italy	
Saskia Biskup	Zentrum für Humangenetik Tübingen, Tübingen, Germany	
Nina Bögershausen	Institute of Human Genetics, University Medical Center Göttingen, Göttingen, Germany	
Knut Brockmann	Department of Pediatrics and Adolescent Medicine, University Medical Center Göttingen, Göttingen, Germany	
Nicola Brunetti-Pierri	Telethon Institute of Genetics and Medicine (TIGEM), Pozzuoli, Naples, Italy	Department of Translational Medicine, Federico II University, Naples, Italy
Peter Burfeind	Institute of Human Genetics, University Medical Center Göttingen, Göttingen, Germany	

Ruben Cabanillas	Cabanillas Precision Consulting, Zurich, Switzerland	Translational Medicine, T-Therapeutics, Cambridge, United Kingdom
Patricia Corriols-Noval	Department of Otorhinolaryngology, Hospital Universitario Marqués de Valdecilla, Santander, Spain	
Elke de Boer	Department of Human Genetics, Radboudumc, 6500 HB, Nijmegen, Netherlands	
Iris de Lange	Department of Genetics, University Medical Center Utrecht, Utrecht, Netherlands	
Charulata Deshpande	Manchester Centre for Genomic Medicine, St Mary's Hospital, Manchester University NHS Foundation Trust, Manchester, United Kingdom	
Marta Diñeiro	Instituto de Medicina Oncológica y Molecular de Asturias (IMOMA), Oviedo, Spain	
Emily Doherty	Carilion Clinic Children's Hospital, Roanoke, United States	
Julia Doll	Institut für Humangenetik, Biozentrum, Universität Würzburg, Würzburg, Germany	
Sofia Douzgou	Department of Medical Genetics, Haukeland University Hospital, Bergen, Norway	
Tracy Dudding-Byth	University of Newcastle, The NSW Genetics of Learning Disability Newcastle, Newcastle, Australia	
Nadja Ehmke	Institute of Medical Genetics and Human Genetics, Charité-Universitätsmedizin Berlin, Corporate member of Freie Universität Berlin and Humboldt-Universität zu Berlin, Berlin, Germany	
Katherine Fawcett	MRC Computational Genomics Analysis and Training Programme (CGAT), MRC Centre for Computational Biology, MRC Weatherall Institute of Molecular Medicine, John Radcliffe Hospital, Oxford, United Kingdom	Department of Population Health Sciences, University of Leicester, LE1 7RH, Leicester, United Kingdom
Carlos R. Ferreira	National Human Genome Research Institute, National Institutes of Health, 20892, Bethesda, United States	
Jan Fischer	Institute for Clinical Genetics, Medical Faculty and University Hospital Carl Gustav Carus, TUD Dresden University of Technology, Fetscherstrabe 78, 01311, Dresden, Germany	

Joel Fluss	Pediatric Neurology Unit, Paediatrics Subspecialties Service, Geneva Children's Hospital, Geneva, Switzerland	
Rocío González-Aguado	Department of Otorhinolaryngology, Hospital Universitario Marqués de Valdecilla, Santander, Spain	
Luitgard Graul-Neumann	Institute of Medical Genetics and Human Genetics, Charité-Universitätsmedizin Berlin, Corporate member of Freie Universität Berlin and Humboldt-Universität zu Berlin, Berlin, Germany	
Andrew Green	UCD School of Medicine and Medical Science, Children’s Health Ireland (CHI) at Crumlin, Dublin, Ireland	
Renzo Guerrini	Neuroscience Department, Meyer Children's Hospital IRCCS, viale Pieraccini 24, 50139, Florence, Italy	Department of NEUROFARBA, University of Florence, viale Pieraccini 6, 50139, Florence, Italy
Asya Gusina	Laboratory of Cytogenetic, Molecular Genetic and Morphological Studies, National Research and Applied Medicine Centre 'Mother and Child", Minsk, Belarus	
Ute Hehr	Center for Human Genetics, Regensburg, Germany	
Maja Hempel	Institute of Human Genetics, Heidelberg University, Heidelberg, Germany	
Michaela AH Hofrichter	Institut für Humangenetik, Biozentrum, Universität Würzburg, Würzburg, Germany	
Ivan Ivanovski	Medical Genetics Unit, Azienda USL-IRCCS di Reggio Emilia, Reggio Emilia, Italy	Institute of Medical Genetics, University of Zurich, Zürich, Switzerland
Wibke G. Janzarik	Department of Neuropediatrics and Muscle Disorders, Center for Pediatrics and Adolescent Medicine, Medical Center, Faculty of Medicine, University of Freiburg, Freiburg, Germany	
Diana Johnson	Department of Medical Genetics, National Health Service, NHS, Leeds, United Kingdom	
Marieke Joosten	Department of Clinical Genetics, Erasmus MC, Rotterdam, Netherlands	
Silke Kaulfub	Institute of Human Genetics, University Medical Center Göttingen, Göttingen, Germany	
Hyun Jung Kim	Department of Pediatrics, Eulji General Hospital, College of Medicine, Eulji University, Seoul, Republic of Korea	

Tjitske Kleefstra	Department of Human Genetics, Radboudumc, 6500 HB, Nijmegen, Netherlands	Donders Institute for Brain, Cognition and Behaviour, Radboud University, 6500 GL, Nijmegen, Netherlands
Eva Klopocki	Institut für Humangenetik, Biozentrum, Universität Würzburg, Würzburg, Germany	
Karla Krause	Institute for Clinical Genetics, Medical Faculty and University Hospital Carl Gustav Carus, TUD Dresden University of Technology, Fetscherstrabe 77, 01310, Dresden, Germany	
Alma Kuechler	Institute of Human Genetics, University Hospital Essen, University Duisburg-Essen, 45122, Essen, Germany	
Maria Kuzyakova	Institute of Human Genetics, University Medical Center Göttingen, Göttingen, Germany	
Martin W. Laass	Department of Pediatrics, Medizinische Fakultät Carl Gustav Carus, TUD Dresden University of Technology, Dresden, Germany	
Augusta Lachmeijer	Department of Genetics, University Medical Center Utrecht, Utrecht, Netherlands	
Wayne Lam	South East of Scotland Clinical Genetics Service, Edinburgh, United Kingdom	
Cha Gon Lee	Department of Pediatrics, Nowon Eulji Medical Center, Eulji University School of Medicine, Seoul, Republic of Korea	
Yun Li	Institute of Human Genetics, University Medical Center Göttingen, Göttingen, Germany	
Vanesa López-González	Sección de Genética Médica, Servicio de Pediatría, Hospital Clínico Universitario Virgen de la Arrixaca, Murcia, Spain	
Karen Low	Department of Clinical Genetics, University Hospitals Bristol NHS Foundation Trust, Bristol, United Kingdom	Centre for Academic Child Health, Bristol Medical School, University of Bristol, Bristol, United Kingdom,
Michael Lyons	Greenwood Genetic Center, Greenwood, United States	
Carlo Marcelis	Department of Clinical Genetics, Radboud University Medical Center, Nijmegen, Netherlands	
Francisco Martinez-Castellano	Unit of Genetics, Hospital Universitari i Politècnic La Fe. Valencia, Valencia, Spain	Genomics Unit, Instituto de Investigación Sanitaria La Fe, 46026, Valencia, Spain

Maarten Massink	Department of Genetics, University Medical Center Utrecht, Utrecht, Netherlands	
Kay Metcalfe	Manchester Centre for Genomic Medicine, St Mary's Hospital, Manchester University NHS Foundation Trust, Manchester, United Kingdom	
Donatella Milani	Fondazione IRCCS Ca' Granda Ospedale Maggiore Policlinico, Milan, Italy	
Shahida Moosa	Division of Molecular Biology and Human Genetics, Faculty of Medicine and Health Sciences, Stellenbosch University, Tygerberg, South Africa	Medical Genetics, Tygerberg Hospital, South Africa
Manuela Morleo	Telethon Institute of Genetics and Medicine (TIGEM), Pozzuoli, Naples, Italy	Department of Precision Medicine, University of Campania “Luigi Vanvitelli”, Naples, Italy
Teresa Neuhann	Institute of Human Genetics, University Medical Center Göttingen, Göttingen, Germany	
Thomas Neumann	Mitteldeutscher Praxisverbund Humangenetik, Halle, Germany	
Huu Nguyen	Department of Human Genetics, Ruhr-University Bochum, Bochum, Germany	
Vincenzo Nigro	Telethon Institute of Genetics and Medicine (TIGEM), Pozzuoli, Naples, Italy	Department of Precision Medicine, University of Campania “Luigi Vanvitelli”, Naples, Italy
Nuha Nimeri	Women’s Wellness and Research Center, NICU, Hamad Medical Corporation, Doha, Qatar	
Ewa Obersztyn	Department of Medical Genetics, Institute of Mother and Child, Warsaw, Poland	
Anne O'Donnell	Division of Genetics and Genomics, Boston Children's Hospital, Boston, United States	
Carmen Orellana	Unit of Genetics, Hospital Universitari i Politècnic La Fe. Valencia, Valencia, Spain	
Estrella Pallas	Department of Otorhinolaryngology, Hospital Álvaro Cunqueiro, Vigo, Spain	
Hans-Jürgen Pander	Institute of Clinical Genetics, Klinikum Stuttgart, Stuttgart, Germany	
Elena Parrini	Neuroscience Department, Meyer Children's Hospital IRCCS, viale Pieraccini 24, 50139, Florence, Italy	
Silke Pauli	Institute of Human Genetics, University Medical Center Göttingen, Göttingen, Germany	

Michele Pinelli	Department of Molecular Medicine and Medical Biotechnologies, University Federico II, Naples, Italy	Telethon Institute of Genetics and Medicine (TIGEM), Pozzuoli, Naples, Italy
Lina Quteineh	Division of Genetic Medicine, Geneva University Hospitals, Geneva, Switzerland	
Julia Rankin	Peninsula Clinical Genetics Service, Royal Devon and Exeter NHS Trust, Exeter, United Kingdom	
Monica Rosello	Unit of Genetics, Hospital Universitari i Politècnic La Fe. Valencia, Valencia, Spain	
Tamanna Roshan Lal	Genetics and Metabolism, Children's National Hospital, Washington, United States	
Vincenzo Salpietro	Department of Neuromuscular Disorders, Queen Square Institute of Neurology, University College London, WC1N 3BG, London, United Kingdom	Department of Biotechnological and Applied Clinical Sciences, University of L'Aquila, 67100, L'Aquila, Italy
Jens Schallner	Department of Neuropediatrics, TUD Dresden University of Technology, Dresden, Germany	
Gregor Schlüter	PRAENATAL, Nürnberg, Germany	
Julia Schmidt	Institute of Human Genetics, University Medical Center Göttingen, Göttingen, Germany	
Mariasavina Severino	Neuroradiology Unit, IRCCS Istituto Giannina Gaslini, Genoa, Italy	
Vandana Shashi	Department of Pediatrics, Division of Medical Genetics, Duke University Medical Center, Durham, United States	
Corinna Siegel	Institute of Human Genetics, Klinikum rechts der Isar, Technical University of Munich, Munich, Germany	Department of Clinical Genetics, MVZ Martinsried, Munich,
Margie Sinnema	Department of Clinical Genetics, Maastricht University Medical Center, Maastricht, Netherlands	
Anne Slavotinek	Division of Genetics, Department of Pediatrics, University of California, San Francisco, United States	Division of Human Genetics, Cincinnati Children's Hospital, 3333 Burnet Ave, Cincinnati OH 45229, United States
Sarah Smithson	Department of Clinical Genetics, University Hospitals Bristol NHS Foundation Trust, Bristol, United Kingdom	
Siddharth Srivastava	Department of Neurology, Boston Children's Hospital, Boston, United States	
Maja Svrakic	Northwell Health Department of Otolaryngology, New York, United States	

Lindsay Swanson	Department of Neurology, Boston Children's Hospital, Boston, United States	
Hannah Thomson	Hunter Genetics, The NSW Genetics of Learning Disability Newcastle, Newcastle, Australia	
Eduardo Tizzano Ferrari	Àrea de Genètica Clínica i Molecular, Hospital Vall d'Hebrón, Barcelona, Spain	
Annalaura Torella	Telethon Institute of Genetics and Medicine (TIGEM), Pozzuoli, Naples, Italy	Department of Precision Medicine, University of Campania “Luigi Vanvitelli”, Naples, Italy
Undiagnosed Diseases Network		
Irene Valenzuela Palafoll	Àrea de Genètica Clínica i Molecular, Hospital Vall d'Hebrón, Barcelona, Spain	
Yolande van Bever	Department of Clinical Genetics, ErasmusMC University Medical Center Rotterdam, 3015 GD, Rotterdam, Netherlands	
Ellen van Binsbergen	Department of Genetics, University Medical Center Utrecht, Utrecht, Netherlands	
Marjon van Slegtenhorst	Department of Clinical Genetics, ErasmusMC University Medical Center Rotterdam, 3015 GD, Rotterdam, Netherlands	
Nienke Verbeek	Department of Genetics, University Medical Center Utrecht, Utrecht, Netherlands	
Virginie Verhoeven	Department of Clinical Genetics, ErasmusMC University Medical Center Rotterdam, Rotterdam, Netherlands	
Barbara Vona	Institute of Human Genetics, University Medical Center Göttingen, Heinrich-Düker-Weg 12, 37073, Göttingen, Germany	Institute for Auditory Neuroscience and InnerEarLab, University Medical Center Göttingen, Robert-Koch-Str. 40, 37075, Göttingen, Germany
Dagmar Wahl	Medical Practice for Genetic Counselling, Center for Human Genetics and Laboratory Diagnostics Martinsried, Augsburg, Germany	
Luisa Weiss	Center for Human Genetics, Regensburg, Germany	

Gökhan Yigit	Institute of Human Genetics, University Medical Center Göttingen, Göttingen, Germany	DZHK (German Center for Cardiovascular Research), partner site Göttingen, Göttingen, Germany
Maha Zaki	Clinical Genetics Department, Human Genetics and Genome Research Institute, National Research Centre, Cairo, Egypt	

References

1. Cuvertino, S., Stuart, H.M., Chandler, K.E., Roberts, N.A., Armstrong, R., Bernardini, L., Bhaskar, S., Callewaert, B., Clayton-Smith, J., Davalillo, C.H., et al. (2017). ACTB Loss-of-Function Mutations Result in a Pleiotropic Developmental Disorder. Am J Hum Genet *101*, 1021-1033. 10.1016/j.ajhg.2017.11.006.
2. Latham, S.L., Ehmke, N., Reinke, P.Y.A., Taft, M.H., Eicke, D., Reindl, T., Stenzel, W., Lyons, M.J., Friez, M.J., Lee, J.A., et al. (2018). Variants in exons 5 and 6 of ACTB cause syndromic thrombocytopenia. Nature communications *9*, 4250. 10.1038/s41467-018-06713-0.
3. Baraitser, M., and Winter, R.M. (1988). Iris coloboma, ptosis, hypertelorism, and mental retardation: a new syndrome. J Med Genet *25*, 41-43.
4. Verloes, A. (1993). Iris coloboma, ptosis, hypertelorism, and mental retardation: Baraitser-Winter syndrome or Noonan syndrome? J Med Genet *30*, 425-426.
5. Ramer, J.C., Lin, A.E., Dobyns, W.B., Winter, R., Ayme, S., Pallotta, R., and Ladda, R.L. (1995). Previously apparently undescribed syndrome: shallow orbits, ptosis, coloboma, trigonocephaly, gyral malformations, and mental and growth retardation. Am J Med Genet *57*, 403-409. 10.1002/ajmg.1320570308.
6. Rossi, M., Guerrini, R., Dobyns, W.B., Andria, G., and Winter, R.M. (2003). Characterization of brain malformations in the Baraitser-Winter syndrome and review of the literature. Neuropediatrics *34*, 287-292. 10.1055/s-2003-44666.
7. Riviere, J.B., van Bon, B.W., Hoischen, A., Kholmanskikh, S.S., O'Roak, B.J., Gilissen, C., Gijsen, S., Sullivan, C.T., Christian, S.L., Abdul-Rahman, O.A., et al. (2012). De novo mutations in the actin genes ACTB and ACTG1 cause Baraitser-Winter syndrome. Nat Genet *44*, 440-444, S441-442. 10.1038/ng.1091.
8. Verloes, A., Di Donato, N., Masliah-Planchon, J., Jongmans, M., Abdul-Raman, O.A., Albrecht, B., Allanson, J., Brunner, H., Bertola, D., Chassaing, N., et al. (2015). Baraitser-Winter cerebrofrontofacial syndrome: delineation of the spectrum in 42 cases. Eur J Hum Genet *23*, 292-301. 10.1038/ejhg.2014.95.
9. Yates, T.M., Turner, C.L., Firth, H.V., Berg, J., and Pilz, D.T. (2017). Baraitser-Winter cerebrofrontofacial syndrome. Clin Genet *92*, 3-9. 10.1111/cge.12864.
10. Di Donato, N., Rump, A., Koenig, R., Der Kaloustian, V.M., Halal, F., Sonntag, K., Krause, C., Hackmann, K., Hahn, G., Schrock, E., and Verloes, A. (2014). Severe forms of Baraitser-Winter syndrome are caused by ACTB mutations rather than ACTG1 mutations. Eur J Hum Genet *22*, 179-183. 10.1038/ejhg.2013.130.
11. Eker, H.K., Derinkuyu, B.E., Unal, S., Masliah-Planchon, J., Drunat, S., and Verloes, A. (2013). Cerebro-Fronto-Facial Syndrome Type 3 With Polymicrogyria: a Clinical Presentation of Baraitser-Winter Syndrome. European journal of medical genetics. 10.1016/j.ejmg.2013.10.005.
12. Verloes, A., Drunat, S., Pilz, D., and Di Donato, N. (1993). Baraitser-Winter Cerebrofrontofacial Syndrome. In GeneReviews((R)), M.P. Adam, H.H. Ardinger, R.A. Pagon, S.E. Wallace, L.J.H. Bean, K. Stephens, and A. Amemiya, eds.
13. Gearing, M., Juncos, J.L., Procaccio, V., Gutekunst, C.A., Marino-Rodriguez, E.M., Gyure, K.A., Ono, S., Santoianni, R., Krawiecki, N.S., Wallace, D.C., and Wainer, B.H. (2002). Aggregation of actin and cofilin in identical twins with juvenile-onset dystonia. Ann Neurol *52*, 465-476. 10.1002/ana.10319.
14. Procaccio, V., Salazar, G., Ono, S., Styers, M.L., Gearing, M., Davila, A., Jimenez, R., Juncos, J., Gutekunst, C.A., Meroni, G., et al. (2006). A mutation of beta -actin that alters depolymerization dynamics is associated with autosomal dominant developmental malformations, deafness, and dystonia. Am J Hum Genet *78*, 947-960. 10.1086/504271.
15. Conboy, E., Vairo, F., Waggoner, D., Ober, C., Das, S., Dhamija, R., Klee, E.W., and Pichurin, P. (2017). Pathogenic Variant in ACTB, p.Arg183Trp, Causes Juvenile-Onset Dystonia, Hearing Loss, and Developmental Delay without Midline Malformation. Case Rep Genet *2017*, 9184265. 10.1155/2017/9184265.
16. Freitas, J.L., Vale, T.C., Barsottini, O.G.P., and Pedroso, J.L. (2020). Expanding the Phenotype of Dystonia-Deafness Syndrome Caused by ACTB Gene Mutation. Mov Disord Clin Pract *7*, 86-87. 10.1002/mdc3.12854.

17. Zhu, M., Yang, T., Wei, S., DeWan, A.T., Morell, R.J., Elfenbein, J.L., Fisher, R.A., Leal, S.M., Smith, R.J., and Friderici, K.H. (2003). Mutations in the gamma-actin gene (ACTG1) are associated with dominant progressive deafness (DFNA20/26). Am J Hum Genet *73*, 1082-1091. S0002-9297(07)61971-510.1086/379286.

18. van Wijk, E., Krieger, E., Kemperman, M.H., De Leenheer, E.M., Huygen, P.L., Cremers, C.W., Cremers, F.P., and Kremer, H. (2003). A mutation in the gamma actin 1 (ACTG1) gene causes autosomal dominant hearing loss (DFNA20/26). J Med Genet *40*, 879-884.

19. Rendtorff, N.D., Zhu, M., Fagerheim, T., Antal, T.L., Jones, M., Teslovich, T.M., Gillanders, E.M., Barmada, M., Teig, E., Trent, J.M., et al. (2006). A novel missense mutation in ACTG1 causes dominant deafness in a Norwegian DFNA20/26 family, but ACTG1 mutations are not frequent among families with hereditary hearing impairment. Eur J Hum Genet *14*, 1097-1105. 10.1038/sj.ejhg.5201670.

20. Teig, E. (1968). Hereditary progressive perceptive deafness in a family of 72 patients. Acta Otolaryngol *65*, 365-372. 10.3109/00016486809120977.

21. Sorrentino, U., Piccolo, C., Rigon, C., Brasson, V., Trevisson, E., Boaretto, F., Martini, A., and Cassina, M. (2021). DFNA20/26 and Other ACTG1-Associated Phenotypes: A Case Report and Review of the Literature. Audiol Res *11*, 582-593. 10.3390/audiolres11040052.

22. Kemerley, A., Sloan, C., Pfeifer, W., Smith, R., and Drack, A. (2016). A novel mutation in ACTG1 causing Baraitser-Winter syndrome with extremely variable expressivity in three generations. Ophthalmic Genet, 1-5. 10.3109/13816810.2016.1164196.

23. Morin, M., Bryan, K.E., Mayo-Merino, F., Goodyear, R., Mencia, A., Modamio-Hoybjor, S., del Castillo, I., Cabalka, J.M., Richardson, G., Moreno, F., et al. (2009). In vivo and in vitro effects of two novel gamma-actin (ACTG1) mutations that cause DFNA20/26 hearing impairment. Hum Mol Genet *18*, 3075-3089. 10.1093/hmg/ddp249.

24. Miyagawa, M., Nishio, S.Y., Ichinose, A., Iwasaki, S., Murata, T., Kitajiri, S., and Usami, S. (2015). Mutational spectrum and clinical features of patients with ACTG1 mutations identified by massively parallel DNA sequencing. Ann Otol Rhinol Laryngol *124 Suppl 1*, 84S-93S. 10.1177/0003489415575057.

25. Yuan, Y., Gao, X., Huang, B., Lu, J., Wang, G., Lin, X., Qu, Y., and Dai, P. (2016). Phenotypic Heterogeneity in a DFNA20/26 family segregating a novel ACTG1 mutation. BMC Genet *17*, 33. 10.1186/s12863-016-0333-1.

26. Bryan, K.E., Wen, K.K., Zhu, M., Rendtorff, N.D., Feldkamp, M., Tranebjaerg, L., Friderici, K.H., and Rubenstein, P.A. (2006). Effects of human deafness gamma-actin mutations (DFNA20/26) on actin function. J Biol Chem *281*, 20129-20139. 10.1074/jbc.M601514200.

27. Drummond, M.C., Belyantseva, I.A., Friderici, K.H., and Friedman, T.B. (2012). Actin in hair cells and hearing loss. Hear Res *288*, 89-99. 10.1016/j.heares.2011.12.003.

28. Rainger, J., Williamson, K.A., Soares, D.C., Truch, J., Kurian, D., Gillessen-Kaesbach, G., Seawright, A., Prendergast, J., Halachev, M., Wheeler, A., et al. (2017). A recurrent de novo mutation in ACTG1 causes isolated ocular coloboma. Hum Mutat *38*, 942-946. 10.1002/humu.23246.
